# Msx1 loss suppresses formation of the ectopic crypts developed in the Apc-deficient small intestinal epithelium

**DOI:** 10.1038/s41598-018-38310-y

**Published:** 2019-02-07

**Authors:** Monika Horazna, Lucie Janeckova, Jiri Svec, Olga Babosova, Dusan Hrckulak, Martina Vojtechova, Katerina Galuskova, Eva Sloncova, Michal Kolar, Hynek Strnad, Vladimir Korinek

**Affiliations:** 1 Institute of Molecular Genetics of the Czech Academy of Sciences, Videnska 1083, 142 20 Prague 4, Czech Republic; 2 Faculty of Science, Charles University in Prague, Albertov 6, 128 43 Praha 2, Czech Republic; 3 Department of Radiotherapy and Oncology, Third Faculty of Medicine, Charles University, Prague, Srobarova 50, 100 34 Prague 10, Czech Republic

**Keywords:** Cancer models, Intestinal stem cells

## Abstract

The first step in the development of human colorectal cancer is aberrant activation of the Wnt signaling pathway. Wnt signaling hyperactivation is predominantly caused by loss-of-function mutations in the adenomatous polyposis coli (*APC*) gene that encodes the pathway negative regulator. In order to identify genes affected by the *Apc* loss, we performed expression profiling of intestinal epithelium isolated from mice harboring a conditional *Apc* allele. The gene encoding transcriptional factor msh homeobox 1 (*Msx*1) displayed robust upregulation upon *Apc* inactivation. Histological analysis of the Apc-deficient epithelium revealed that in the small intestine, the Msx1 protein was localized exclusively in ectopic crypts, i.e., in pockets of proliferating cells abnormally positioned on the villi. Ablation of the *Msx1* gene leads to the disappearance of ectopic crypts and loss of differentiated cells. Moreover, tumors arising from Msx1-deficient cells display altered morphology reminiscent of villous adenomas. In human tumor specimens, *MSX1* displayed significantly increased expression in colonic neoplasia with a descending tendency during the lesion progression towards colorectal carcinoma. In summary, the results indicate that Msx1 represents a novel marker of intestinal tumorigenesis. In addition, we described the previously unknown relationship between the Msx1-dependent formation of ectopic crypts and cell differentiation.

## Introduction

With a rate of entire renewal every 3–5 days, well-defined organization of the tissue compartments containing proliferating and differentiated cells, the epithelial lining of the gastrointestinal (GI) tract represents an attractive paradigm for tissue maintenance studies. The homeostasis of the tissue is sustained by multipotent intestinal stem cells (ISCs) that reside at the bottom of submucosal invaginations of the single-layer epithelium called the crypts of Lieberkühn. Intestinal stem cells divide approximately every 24 hours, generating a pool of transit-amplifying (TA) cells that are rapidly dividing progenitors located above ISCs. The TA cells migrate upwards and while exiting the crypt, they differentiate into several cell types that mainly include absorptive enterocytes, hormone-releasing enteroendocrine cells, and mucus-producing goblet cells. In the small intestine, the differentiated cells cover fingerlike microscopic projections called villi; the surface of the large intestine is flat. The differentiated cells are short-lived and after several days are extruded from the epithelium into the gut lumen. The only exception are Paneth cells. These bactericidal post-mitotic cells present in the small intestine do not migrate from the crypts but stay at the crypt base, where they function for 6–8 weeks (reviewed in^1^). The Wnt signaling pathway is activated in the cells present in the lower part of the intestinal crypts. The pathway drives proliferation and pluripotency of ISCs and contributes to differentiation of the Paneth cells. Additionally, aberrant activation of the Wnt pathway increases the stem cell numbers and initiates tumorigenesis of the GI tract (reviewed in^2^). In the absence of Wnt stimulus β-catenin, the key molecule of the best defined so-called canonical branch of the pathway, is phosphorylated at its N-terminus, subsequently ubiquitinated, and degraded by the proteasome. Binding of the Wnt molecules to the transmembrane complex composed of Frizzled (Fzd) and low-density lipoprotein receptor 5/6 (LRP5/6) induces a cascade of events that leads to β-catenin stabilization. A portion of the cytoplasmic β-catenin pool translocates to the cell nucleus, where it associates with transcription factors of the T-cell-specific transcription factor (TCF)/lymphoid enhancer binding factor (LEF) family and activates expression of the Wnt target genes (reviewed in)^3,4^.

Basic information about the genetic program controlled by the Wnt/β-catenin pathway in the intestine was obtained by studying tumor cells derived from cancer affecting the colon and rectum. Colorectal carcinoma (CRC) constitutes one of the most commonly diagnosed neoplasia in developed countries^5^. Intriguingly, the majority (>80%) of sporadic colorectal tumors contain mutations in the tumor suppressor adenomatous polyposis coli (*APC*) gene, which encodes the negative regulator of canonical Wnt signaling^6^. The APC protein together with another tumor suppressor, axis inhibition protein 1/2 (Axin 1/2), function as scaffolding components of the β-catenin degradation complex. The complex also includes casein kinase 1 alpha (CK1α) and glycogen synthase kinase 3 (GSK-3), which phosphorylate β-catenin, marking it for subsequent degradation. Aberrant (hyper)activation of the Wnt pathway in the mouse intestinal epithelium using homozygous deletion of the *Apc* gene or β-catenin stabilization instantly promotes cellular proliferation while impairing differentiation^7–9^. In 2002, van de Wetering and colleagues identified leucine-rich repeat-containing G-protein-coupled receptor 5 (LGR5) as a gene upregulated by aberrant Wnt signaling in human colon cancer cells. Subsequent lineage tracing experiments performed in genetically modified mice revealed that Lgr5 is specifically produced in ISCs^10^.

To characterize the changes induced by Apc loss we performed expression profiling of the intestinal epithelium isolated from mice harboring the conditional allele of the *Apc* gene. We identified msh homeobox 1 (*Msx1*) as a gene prominently upregulated in Apc-deficient tissue. Msx1 (also known as Hox7) belongs to the muscle segment homeobox (msh) family that includes one of the most evolutionarily conserved homeobox transcription factors found in animals (reviewed in)^11,12^. Msx1 may act as a transcriptional activator and/or repressor, and its function depends on the cellular context and interacting partners. We used several mouse models of intestinal cancer to demonstrate that Msx1 represents a robust marker of intestinal tumorigenesis induced by aberrant Wnt signaling. However, in contrast to the other intestinal genes regulated by the Wnt pathway, Msx1 was exclusively expressed in ectopic crypts, abnormally positioned crypts formed on the villi in the orthogonal orientation to the crypt-villus axis. Intriguingly, simultaneous deletion of *Apc* and *Msx1* suppressed ectopic crypt formation and converted the epithelium to a highly proliferative compartment with reduced cell differentiation. Furthermore, analysis of human tumor specimens showed that *MSX1* is upregulated in various progression stages of intestinal neoplasia. In summary, our data clearly demonstrate that in transformed Apc-deficient cells, β-catenin-dependent transcription is influenced by the cell position in the epithelium. Additionally, our results revealed the previously unknown relationship between the Msx1-dependent formation of ectopic crypts and cell differentiation.

## Results

### *Msx1* expression is upregulated in the mouse intestine and human cells upon Wnt/β-catenin pathway hyperactivation

**Figure 1:**
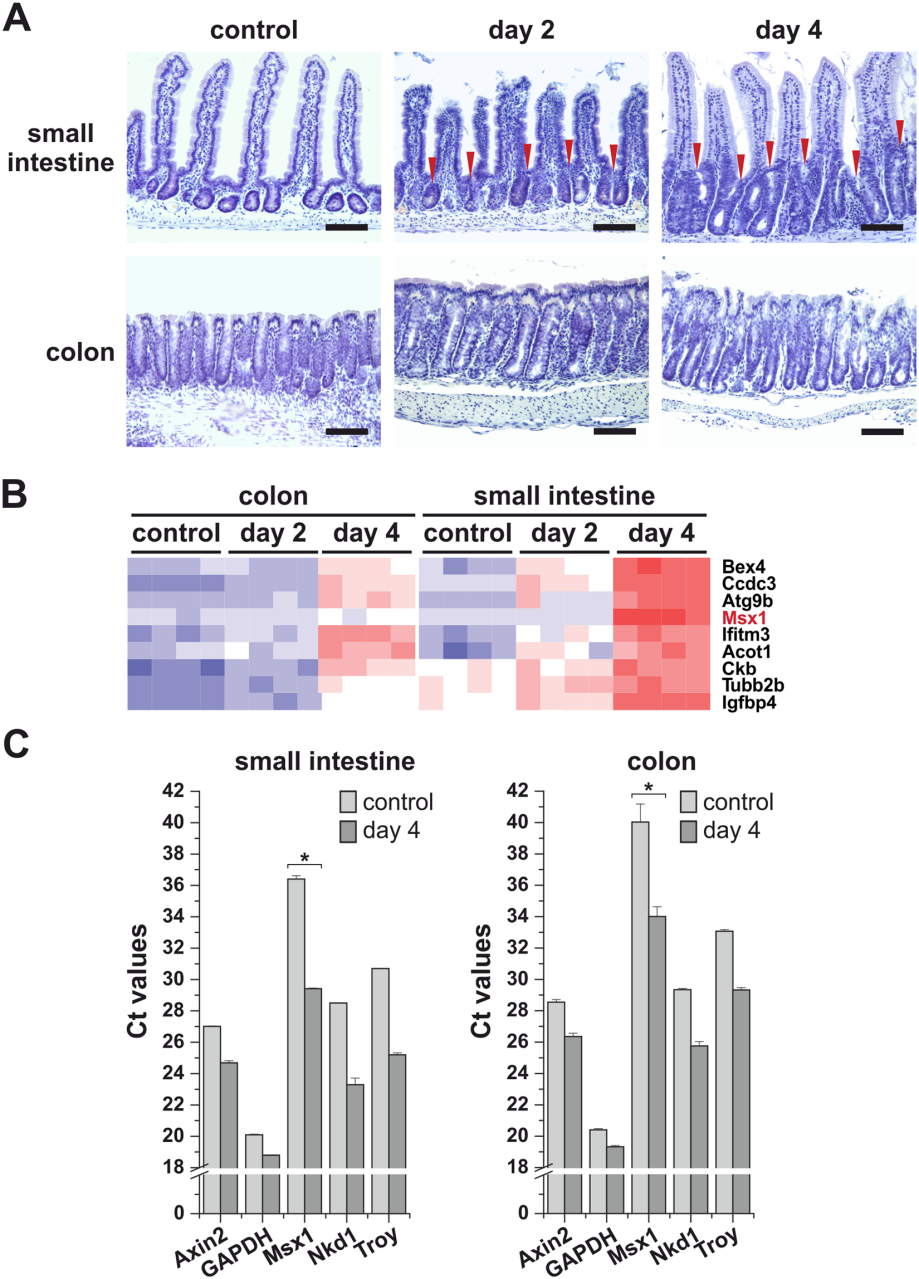
*Msx1* expression is upregulated upon *Apc* gene inactivation in the mouse intestine. (**A**) Crypt hyperplasia arising in *Apc*^*cKO/cKO*^ *Villin-CreERT2* small intestine 2 and 4 days after tamoxifen administration. Hematoxylin-stained (blue nuclei) paraffin sections of the small intestine (jejunum) and colon at the indicated time points upon tamoxifen administration are shown. Control tissues were obtained from mice of the same genetic background prior to tamoxifen treatment. Red arrowheads indicate hyperproliferative crypt compartments. Scale bar: 0.15 mm. (**B**) Expression profiling of *Apc*^*cKO/cKO*^ *Villin-CreERT2* small intestinal and colonic crypt cells 2 and 4 days after tamoxifen administration. Control RNA was isolated from crypt cells with intact *Apc*. A part of the heatmap is presented showing robust upregulation of the *Msx1* gene upon *Apc* inactivation in both tissues. For a complete list of differentially expressed genes, see supplementary Table S1 (small intestine) and Supplementary Table S2 (colon). (**C**) Quantitative RT-PCR analysis confirms a significant increase in the *Msx1* expression levels upon Apc loss. Total RNA was isolated from *Apc*^*cKO/cKO*^ *Villin-CreERT2* small intestinal and colonic crypts 4 days after tamoxifen administration. Control RNA samples were obtained from the animals treated with the solvent only. The diagrams show threshold cycle (Ct) values normalized to *β-actin* gene expression (the *β-actin* gene Ct value in this and other diagrams was arbitrarily set to 17). *Axin2*, *Nkd1*, and *Troy* represent the Wnt/β-catenin-responsive genes. *GAPDH* was – next to *β-actin* – used as an additional housekeeping gene. RNA samples obtained from four tamoxifen-treated and four control animals were analyzed; qRT-PCR reactions were run in technical triplicates. The diagrams show representative results obtained from one animal; error bars indicate standard deviations (SDs); *p < *0.05*; **p < *0.01*.

To analyze the changes in intestinal epithelial cells upon the loss of the *Apc* gene we performed expression profiling of small intestinal and colonic crypts isolated from *Apc*^*cKO/cKO*^ *Villin-CreERT2* mice. Mice of the *Apc*^*cKO/cKO*^ strain are homozygous for a conditional knock-out (cKO) allele of the *Apc* gene. The allele was generated by flanking exon 14 with loxP site sequences. The Cre-mediated excision of the exon changes the reading frame of the sequence downstream of the deletion. This results in production of a truncated (nonfunctional) Apc polypeptide^13^. Transgenic *Villin-CreERT2* mice express CreERT2 recombinase driven from the murine *villin* gene promoter allowing tamoxifen-inducible inactivation of Apc in the entire adult intestinal epithelium^14^. Progressive crypt extension was observed in the small intestine as early as two days upon Apc loss; the colon was seemingly less affected (Fig. 1A). Subsequently, the expression profile of the intestinal genes influenced by Apc deficiency was analyzed by DNA microarray hybridization. The analysis was performed using total RNA isolated from fresh epithelial crypts of the small intestine and colon prior to and at days 2 and 4 after tamoxifen injection. In the Apc-deficient small intestine, increased expression of the Wnt target gene and ISC marker tumor necrosis factor receptor superfamily, member 19 (*Tnfrsf19*; alternative name *Troy*) was detected already at day 2. At day 4, robust upregulation of additional crypt-specific Wnt-responsive genes *Lgr5*, achaete-scute complex homolog 2 (*Ascl2*), *Axin2*, and Sp5 transcription factor (*Sp5*) was observed. In agreement with previously published data, increased expression of Paneth cell-specific markers lysozyme 1 (*Lyz1*) and defensins (*Defa6*, *Defa26*) was found in the colon at day 2 after tamoxifen administration^8^. Similarly as in the small intestine, *Lgr5*, *Ascl2*, *Axin2*, and *Sp5* genes were upregulated in the Apc-deficient colon at day 4. The gene encoding transcription factor *Msx1* displayed significantly increased expression in the small intestine four days after Apc inactivation. In the colon, the expression change was less pronounced [the binary logarithm of fold change (logFC) 0.77 vs. 3.53; Fig. 1B]. A complete list of differentially expressed genes with |logFC| ≥ 1 and q-value < 0.05 is given in Supplementary Table S1 (small intestine) and Supplementary Table S2 (colon). Reverse-transcription quantitative polymerase chain reaction (qRT-PCR) analysis confirmed the result of the expression profiling; the analysis included additional Wnt target gene naked cuticle homolog 1 (*Nkd1*; Fig. 1C).

**Figure 2:**
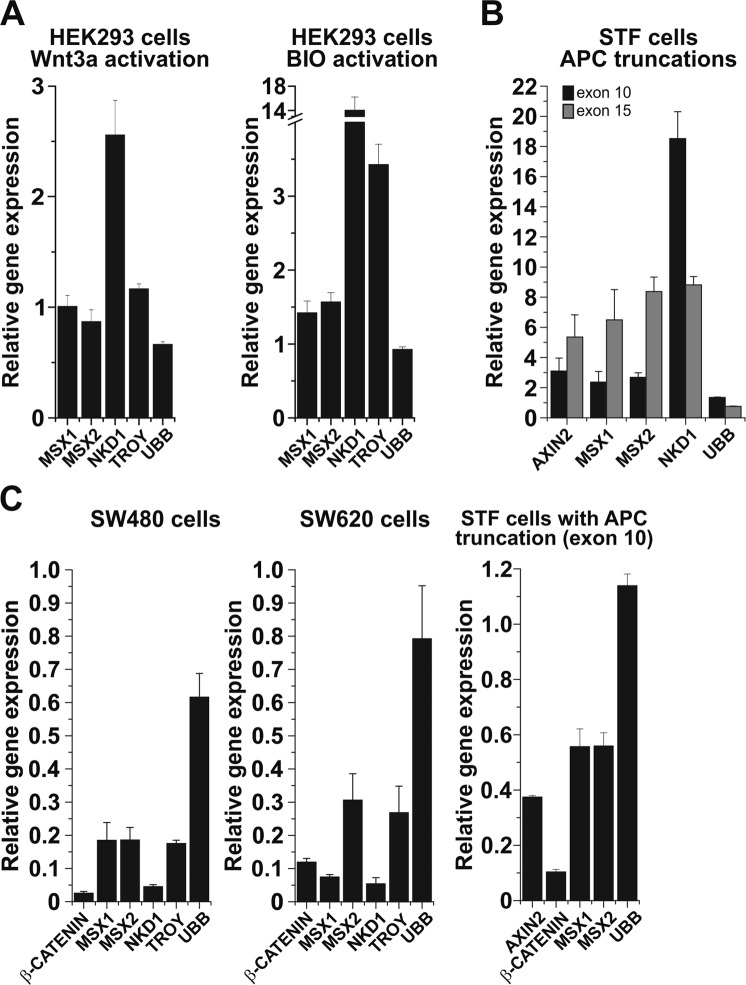
Aberrant Wnt/β-catenin signaling activates *MSX1* expression in human cells. (**A**) Quantitative RT-PCR analysis of the *MSX1* mRNA expression level in HEK293 cells upon treatment with Wnt3a-conditioned medium (left diagram) or with GSK3 inhibitor BIO (right diagram). The diagrams show expression levels of the indicated gene in Wnt3a- or BIO-treated cells relative to the levels determined in control cells without treatment. (**B**) *MSX1* is upregulated in STF cells expressing truncated APC. Quantitative RT-PCR analysis of STF cells producing wild-type or truncated APC proteins. The mutant *APC* gene was generated by targeting exon 10 or exon 15 using TALENs or the CRISPR/Cas9 system, respectively. The diagram shows corresponding Ct values upon normalization to *β-actin* mRNA levels. (**C**) Decreased expression of *MSX1* upon siRNA-mediated knock-down of β*-catenin* mRNA. Quantitative RT-PCR analysis of SW480 and SW620 CRC cells and STF cells expressing a truncated APC variant generated by exon 10 targeting transfected with β-catenin-specific siRNA. The gene expression level in cells transfected with non-silencing siRNA was set to 1. Each analysis depicted in panels A, B, and C was performed at least twice; results of one representative experiment for the given experimental setup are shown. PCR reactions were run in triplicates; error bars indicate SDs. The amounts of total RNA in individual samples were normalized to β-actin expression, the results for additional housekeeping gene ubiquitin B (*UBB*) are shown. Corresponding Ct values are given in Supplementary Table S3.

Next, we tested the responsiveness of the *MSX1* gene to different stimuli activating (or inhibiting) the Wnt pathway in cultured human cells. In human embryonic kidney (HEK) 293 cells, the pathway was activated by conditioned medium containing Wnt3a ligand or GSK3β inhibitor (2′Z,3′E)-6-bromoindirubin-3′-oxime (BIO). The latter treatment activates Wnt signaling at the cytoplasmic level by preventing β-catenin phosphorylation and degradation. Quantitative RT-PCR analysis revealed increased expression of all putative Wnt target genes including *MSX1* and its paralog *MSX2* in both Wnt3a- and BIO-treated cells. Nevertheless, in comparison to other tested Wnt signaling target genes, *MSX1* (and *MSX2*) expression was not increased (Wnt3a stimulation) or upregulated only moderately (BIO treatment; Fig. 2A). Alternatively, Wnt signaling was – similarly to the experiment performed in genetically modified mice – activated by disruption of the *APC* gene. For the assay, HEK293 derivative SuperTOPFLASH (STF) cells containing a genome-integrated Wnt luciferase reporter SuperTOPFLASH were used. The cells enable easy monitoring of the Wnt pathway status by luciferase activity quantification^15^. Two variants of STF cells expressing different truncated forms of APC were utilized. STF cells containing frame-shift mutations in exon 10 of the *Apc* gene were generated previously by transcription activator-like effector nucleases (TALENs)-mediated gene targeting^16^. In addition, using the clustered regularly interspaced short palindromic repeats (CRISPR)/Cas9 system we targeted exon 15 that contains the mutational hotspot in the *APC* gene^17^ and we generated STF cells producing a longer version of mutated APC polypeptide (Supplementary Fig. S1A–C). In both STF cell variants we observed increased production of all tested Wnt signaling target genes (compared to parental STF cells with intact APC). The *MSX1* and *MSX2* genes were upregulated up to six and eight times, respectively (Fig. 2B). Conversely, depletion of β-catenin mRNA using small interfering RNAs (siRNAs) in human APC-deficient CRC cells SW480 and SW620^18^ or in STF cells producing truncated APC protein resulted in a substantial decrease in mRNA levels encoding *MSX1* and *MSX2* (Fig. 2C).

### Msx1 marks ectopic crypts formed in the Apc-deficient small intestine

**Figure 3:**
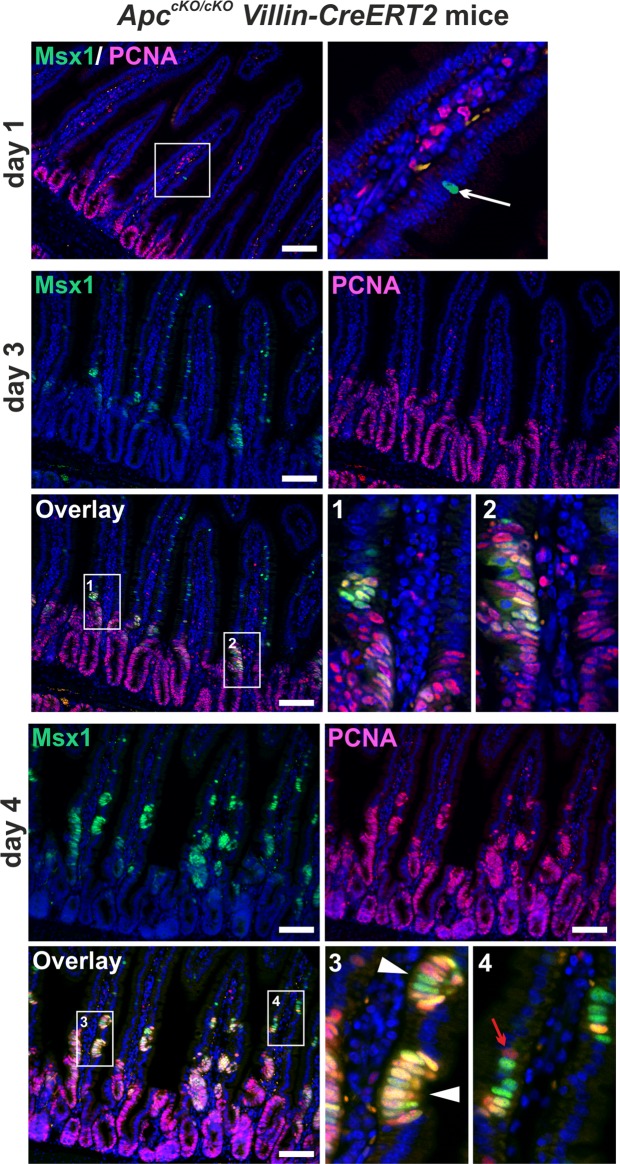
Msx1 marks ectopic crypts formed on the small intestinal villi upon Apc loss. Fluorescent microscopy images of Msx1 (green fluorescent signal) and PCNA (red florescent signal) protein localization in *Apc*^*cKO/cKO*^ *Villin-CreERT2* small intestine 2, 3, and 4 days after tamoxifen administration. Rare Msx1-positive cells (white arrow) in PCNA-negative areas are observed at day 2. At day 3, groups of cells expressing Msx1 are detected on the villi (see insets Nos 1 and 2). At day 4, ectopic crypts containing Msx1- and PCNA-positive cells are formed on the villi (white arrowheads in inset No. 3). Some of these cells co-express Msx1 and PCNA (yellow fluorescence). Occasionally, villus cells produce PCNA, but they are Msx1-negative (red arrow in inset No. 4). Specimens were counterstained with 4′,6-diamidine-2′-phenylindole dihydrochloride (DAPI; nuclear blue florescent signal). Notice that the purple color results from the coalescence of the blue and red fluorescent signal. Boxed areas are magnified in the insets. Scale bar: 0.15 mm.

Subsequently, we used immunofluorescent staining to localize the Msx1 protein in the intestinal epithelium of *Apc*^*cKO/cKO*^ *Villin-CreERT2* mice 2, 3, 4, and 7 days after *Apc* inactivation. To prevent premature death of the experimental animals, we lowered the dose of tamoxifen to 1 mg/per animal, i.e., to 20% of the amount used for the gene expression profiling experiment. Interestingly, at day 2, Msx1-positive nuclei were observed in rare cells localized mainly on the small intestinal villi. The cells were seemingly non-dividing, since proliferating cell nuclear antigen (PCNA) staining did not reveal any obvious colocalization of the PCNA and Msx1 signal. At day 3, cells expressing Msx1 were more abundant and started to form clusters. Some of these clusters, especially those localized to the enlarged (hyperplastic) crypts, contained proliferating cells. At days 4 and 7, Msx1 marked proliferating cells abnormally covering the villi (Fig. 3 and Supplementary Fig. S2). Interestingly, co-staining revealed that not all proliferating, i.e. PCNA-positive cells, expressed Msx1 (Fig. 3).

**Figure 4:**
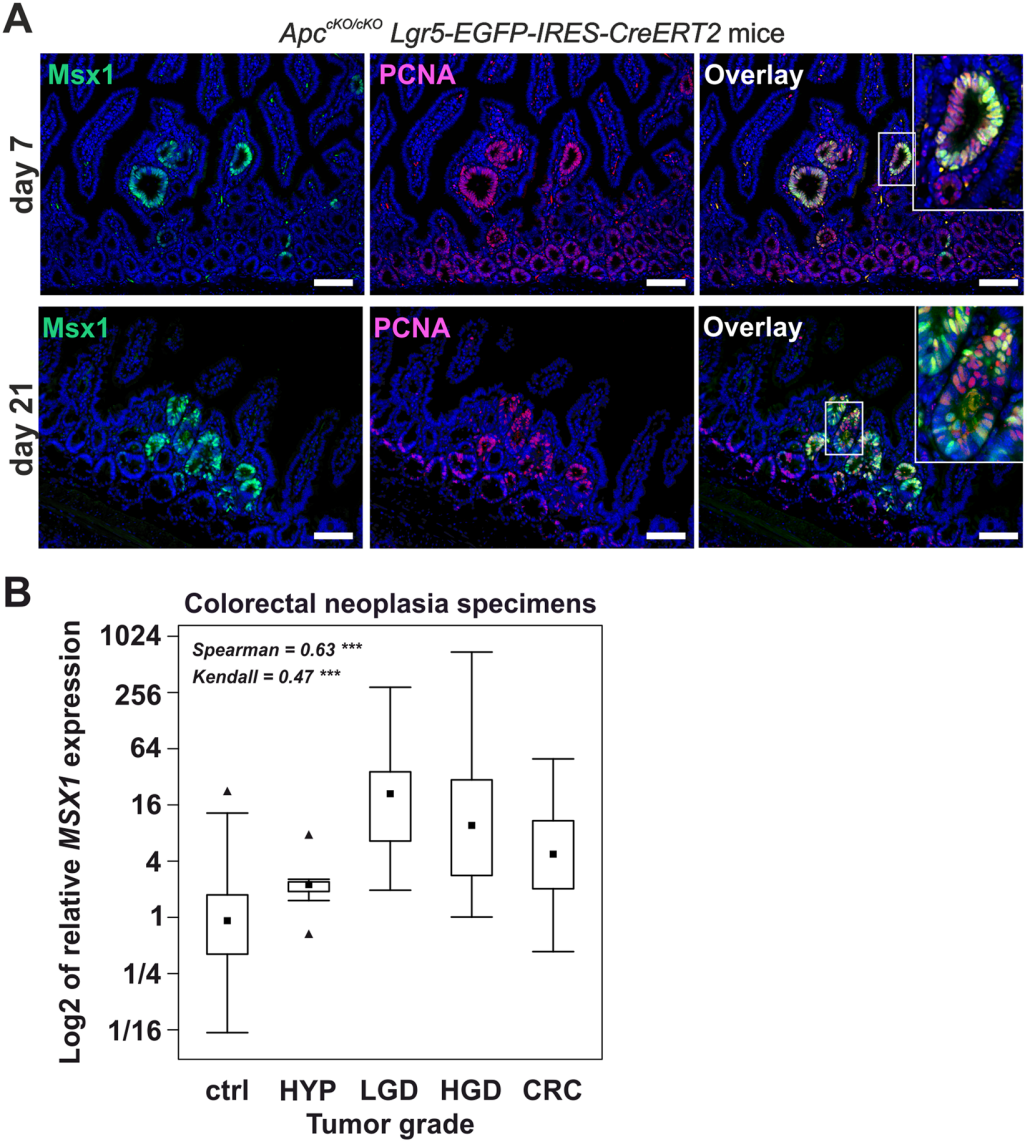
Tumor cells are Msx1-positive. (**A**) Immunofluorescent localization of Msx1 (green fluorescent signal) and PCNA (red florescent signal) in (micro)adenomas formed in the small intestine of *Apc*^*cKO/cKO*^ *Lgr5-EGFP-IRES-CreERT2* mice. The tissue was analyzed 7 and 21 days after tamoxifen administration. Specimens were counterstained with DAPI; boxed areas are magnified in the insets. Scale bar: 0.15 mm. (**B**) Analysis of *MSX1* expression changes during colorectal neoplasia progression. Quantitative RT-PCR analysis of the *MSX1* mRNA levels in healthy tissue (ctrl), hyperplastic adenomas (HYP; n = 9), adenomas displaying low-grade (LGD; n = 27) or high-grade (HGD; n = 24) dysplasia, and CRC (n = 12). The boxed areas correspond to the second and third quartiles; the median of ΔCt values for each category is indicated as the black dot. The range of the values is given by “whiskers” above and below each box. “Outliers” are indicated by black triangles. The RNA content in individual isolates was normalized to the geometric average of Ct values of housekeeping genes *UBB* and *β2-microglobulin*. The relation between the *MSX1* expression profile and neoplasia progression is significant as evidenced by the Spearman (ρ = 0.63) and Kendall (0.47) coefficient values; ***p < *0.001*.

Next, we analyzed Msx1 expression in intestinal lesions developed in *Apc*^*cKO/cKO*^ *Lgr5-EGFP-IRES-CreERT2* mice. The mice enable tamoxifen-induced ISC-specific inactivation of *Apc*. The animals were sacrificed at several time points (after *Apc* inactivation) and analyzed by immunohistochemistry. Msx1-positive cells were visible at day 4 in proliferating enlarged small intestinal crypts (Supplementary Fig. S3). At days 7 and 21, cells producing Msx1 were present in (micro)adenomas. Similarly to *Apc*^*cKO/cKO*^ *Villin-CreERT2* mice, Msx1 and PCNA co-staining revealed that not all proliferating cells are Msx1-positive (Fig. 4A and Supplementary Fig. S3). A similar staining pattern was observed in tumors formed in the small intestine of *Apc*^+/*Min*^ mice. The mice carry a nonsense mutation in one allele of the *Apc* gene and as the result of random inactivation of the second “healthy” allele develop numerous predominantly small intestinal tumors in adulthood^19^. In *Apc*^+/*Min*^ mice, Msx1 was detected in the upper portions of the small intestinal adenomas or in the colonic aberrant crypt foci (ACF), but not in the crypts (Supplementary Fig. S4A). This Msx1 localization was confirmed by *in situ* hybridization (ISH) using an Msx1-specific antisense probe (Supplementary Fig. S4B). Increased Msx1 expression in different Apc-deficient tumors developed in *Apc*^+/*Min*^ mice was subsequently verified by qRT-PCR. In addition, analysis of RNA isolated from multiple tumors growing in several mice did not show any correlation between *Msx1* expression levels and tumor size or position along the rostro-caudal axis of the small intestine (Supplementary Fig. S1C).

Finally, we examined the expression pattern of human *MSX1* in a collection of colonic tumors. The *MSX1* mRNA level was increased in all types of human intestinal neoplasia tested. Nevertheless, the most robust upregulation of *MSX1* mRNA was detected in adenomas with low-grade dysplasia. Moreover, *MSX1* mRNA abundance showed a descending tendency as the lesions progressed towards more progressed phenotypes (Fig. 4B).

### Msx1 deficiency changes morphology of small intestinal tumors

**Figure 5:**
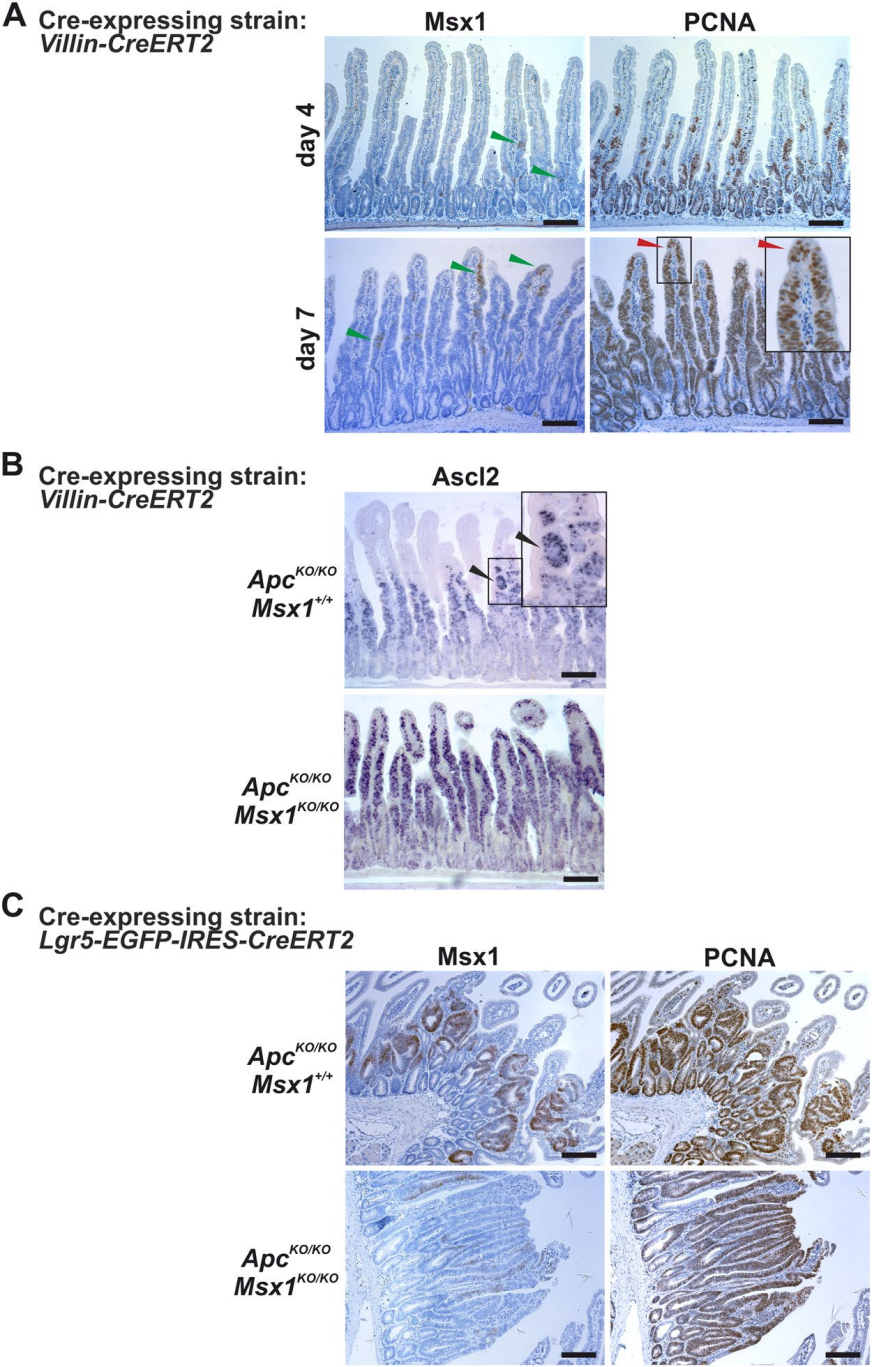
Msx1 is essential for tubular morphology of early small intestinal adenomas. (**A**) Immunodetection of Msx1 and PCNA in *Apc*^*cKO/cKO*^ *Msx1*^*cKO/cKO*^ *Villin-CreERT2* mice at indicated time points after tamoxifen administration. At day 7, Msx1 depletion results in expansion of the proliferating cell compartments, and PCNA-positive cells reach tips of the villi (red arrowhead). Notice that the gene recombination is not complete and groups of Msx1-positive proliferating cells are occasionally detected on the villi (green arrowhead). (**B**) Detection of mRNA encoding stem cell marker *Ascl2* in *Apc*^*cKO/cKO*^ *Villin-CreERT2* (*Apc*^*KO/KO*^ *Msx1*^+/+^) and *Apc*^*cKO/cKO*^ *Msx1*^*cKO/cKO*^ *Villin-CreERT2* (*Apc*^*KO/KO*^ *Msx1*^*KO/KO*^) using ISH. The slides were processed 7 days after tamoxifen administration. Notice that an anti-sense *Ascl2* probe robustly stains the hyperplastic and ectopic crypts developed on the villi (black arrowheads). (**C**) Msx1 loss alters morphology of Apc-deficient adenomas. Msx1 and PCNA immunohistochemical staining in neoplastic lesions developed in the small intestine of *Apc*^*cKO/cKO*^ *Lgr5-EGFP-IRES-CreERT2* (*Apc*^*KO/KO*^ *Msx1*^+/+^) and *Apc*^*cKO/cKO*^ *Msx1*^*cKO/cKO*^ *Lgr5-EGFP-IRES-CreERT2* (*Apc*^*KO/KO*^ *Msx1*^*KO/KO*^) mice 21 days after tamoxifen administration. In contrast to the tubular adenoma developed in the mouse intestine with the intact *Msx1* gene, the Msx1 deficiency results in formation of the villous type lesions. Sections in A and C were counterstained with hematoxylin. Boxed areas are magnified in the insets. The histological analysis was performed using samples obtained from ten animals (five for each genotype); representative images are shown. Additional sections are shown in Supplementary Fig. S6. The employed “Cre-deletor” mouse strains are indicated next to the corresponding images. Scale bar: 0.15 mm.

Since the whole body inactivation of the *Msx1* gene leads to neonatal death, we further employed the conditional allele of the gene. *Msx1*^*cKO/cKO*^ mice were intercrossed with *Villin-Cre* mice. The transgenic mice express constitutively active Cre enzyme in all intestinal epithelial cells starting at embryonic day (E) 12.5 to adulthood^20^. Nevertheless, no pathological changes were observed in the small or large intestine upon continuous inactivation of the *Msx1* gene. Additionally, Msx1 loss did not affect the growth rate and morphology of intestinal organoids (Supplementary Fig. S5). In *Msx1*^*cKO/cKO*^ *Apc*^*cKO/cKO*^ *Villin-CreERT2* mice 4 days after tamoxifen administration, we did not notice any remarkable differences except for the absence of Msx1 staining. However, at day 7, the absence of Msx1 changed the morphological features of hyperplastic epithelium. In contrast to Msx1-proficient epithelium, the epithelial layer was mainly composed of PCNA-positive cells (Fig. 5A and Supplementary Fig. S6). Intriguingly, *Ascl2* expression was not localized only to the crypts (ectopic or hyperplastic); however, the Ascl2-positive areas were substantially enlarged and sometimes reached tips of the villi (Fig. 5B). The increase in proliferation was accompanied by loss of cell differentiation, as evidenced by the absence of histone 3 trimethylation on lysine 27 (H3K27me3) that is specific for differentiated epithelial cells^21^ (Supplementary Fig. S7). In addition, co-deletion of Msx1 and Apc in ISCs of *Msx1*^*cKO/cKO*^ *Apc*^*cKO/cKO*^ *Lgr5-EGFP-IRES-CreERT2* mice altered the appearance of intestinal tumors. Whereas adenomas with intact Msx1 displayed a “typical” tubular shape, the Msx1-deficient tumors were transformed to adenomas with villus-like morphology (Fig. 5C). To gain a mechanistic explanation for the observed morphological change, we performed expression profiling of small intestinal epithelial cells isolated from *Msx1*^*cKO/cKO*^ *Apc*^*cKO/cKO*^ *Villin-CreERT2* and *Apc*^*cKO/cKO*^ *Villin-CreERT2* mice. Although the difference in gene expression between Msx1 wt and Msx1-deficient tumor cells was rather negligible (one of the significance criteria, i.e. q-value < 0.05, was never reached), a set of differentially expressed genes (significance criterion: |logFC| ≥ 1 and p-value ≤ 0.05) was identified (the gene set is given in Supplementary Table S4) and analyzed using the online tool Enrichr^22,23^. However, the analysis did not reveal any signaling pathway, biological process, or molecular function significantly altered by the Msx1 absence. In addition, we performed qRT-PCR analysis for selected Wnt target genes and intestinal cell population markers. The analysis confirmed a (moderate) increase in the expression of *Ascl2* and other Wnt target genes *Axin2*, *Lgr5*, and *SP5* in *Apc/Msx*^*−*^double-deficient tumor cells when compared to the cells with intact *Msx1* gene. In the same comparison, sucrose isomaltase (*SI*) and chromogranin A (*CHGA*) mRNA encoding markers of enterocytes or enteroendocrine cells, respectively, were downregulated, confirming (further) reduction in cell differentiation upon Msx1 loss. As expected, downregulation of *Msx1* mRNA upon Cre enzyme activation was also observed (Supplementary Fig. S8).

**Figure 6:**
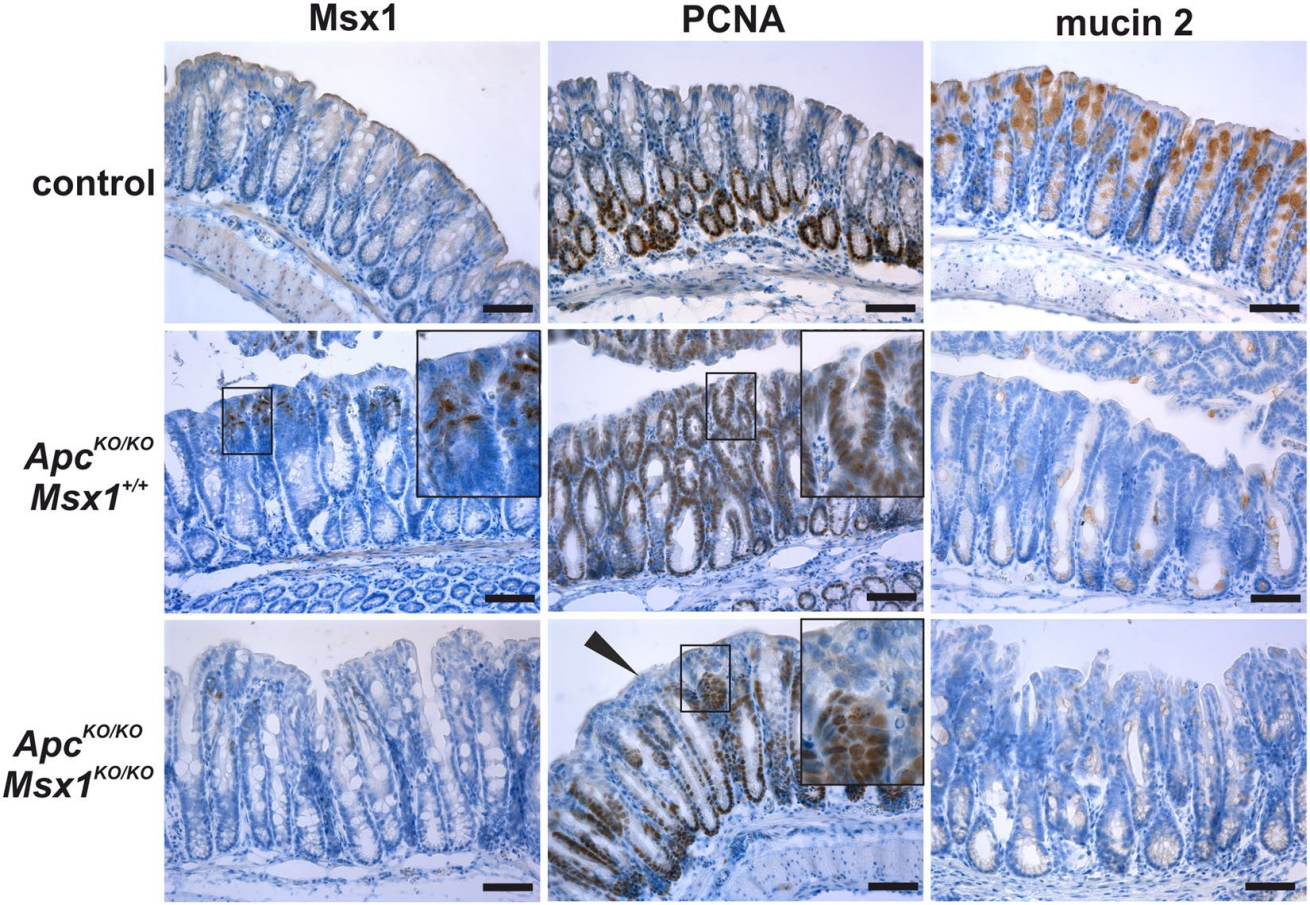
De novo expression of Msx1 in the Apc-deficient colon. Immunohistochemical staining of Msx1, PCNA, and mucin 2 in wt (control), *Apc*^*cKO/cKO*^ *Villin-CreERT2* (*Apc*^*KO/KO*^ *Msx1*^+/+^), and *Apc*^*cKO/cKO*^ *Msx1*^*cKO/cKO*^ *Villin-CreERT2* (*Apc*^*KO/KO*^ *Msx1*^*KO/KO*^) mice 7 days after tamoxifen administration. Notice the absence of PCNA staining in the crypts close to the colonic surface upon Msx1 inactivation (black arrowhead). In contrast, production of goblet cells marker mucin 2 is impaired in the hyperplastic epithelium irrespective of the *Msx1* gene status. The histological analysis was performed using samples obtained from nine animals (three for each genotype); representative images are shown. Sections were counterstained with hematoxylin. Boxed areas are magnified in the insets. Scale bar: 0.15 mm.

Next, we analyzed Msx1 expression and function in the colon. Similarly as in the small intestine, *Msx1* mRNA and protein was absent in the colon at homeostatic conditions. However, after Apc inactivation (in tamoxifen-treated *Apc*^*cKO/cKO*^ *Villin-CreERT2* mice), nuclear Msx1 protein was detected in the upper portion of the hyperplastic crypts. The staining was less prominent than in the small intestine, although recombination efficiency of the floxed *Apc* alleles (judged from the extent of the crypt hyperplasia) was comparable between these two organs (Supplementary Fig. S9). Additionally, Msx1 expression was mainly observed in the proximal part of the colon. Concomitant inactivation of Apc and Msx1 in *Msx1*^*cKO/cKO*^ *Apc*^*cKO/cKO*^ *Villin-CreERT2* mice seemingly reduced formation of proliferating crypts adjacent to the colon lumen. Nevertheless, the absence of staining for goblet cell marker mucin 2 indicated loss of cell differentiation in both Msx1-proficient or Msx1-deficient epithelia (Fig. 6). To identify possible changes induced by Msx1 we performed expression profiling of the control (the *Apc* and *Msx1* genes intact), Apc-deficient, and Apc/Msx1 double-deficient colonic epithelium obtained from the proximal third of the organ. However, expression of only one gene, encoding serine/threonine kinase 32B (Stk32b), differed significantly between Msx1 wt and Msx1-deficient tumor tissue (Supplementary Table S6). Subsequent qRT-PCR analysis confirmed that except for *Stk32b* and *Msx1*, the expression levels of none of the tested genes changed significantly between the analyzed samples (Supplementary Fig. S10 and Supplementary Table S7).

### Expression profiling of MSX1-deficient CRC cells

**Figure 7:**
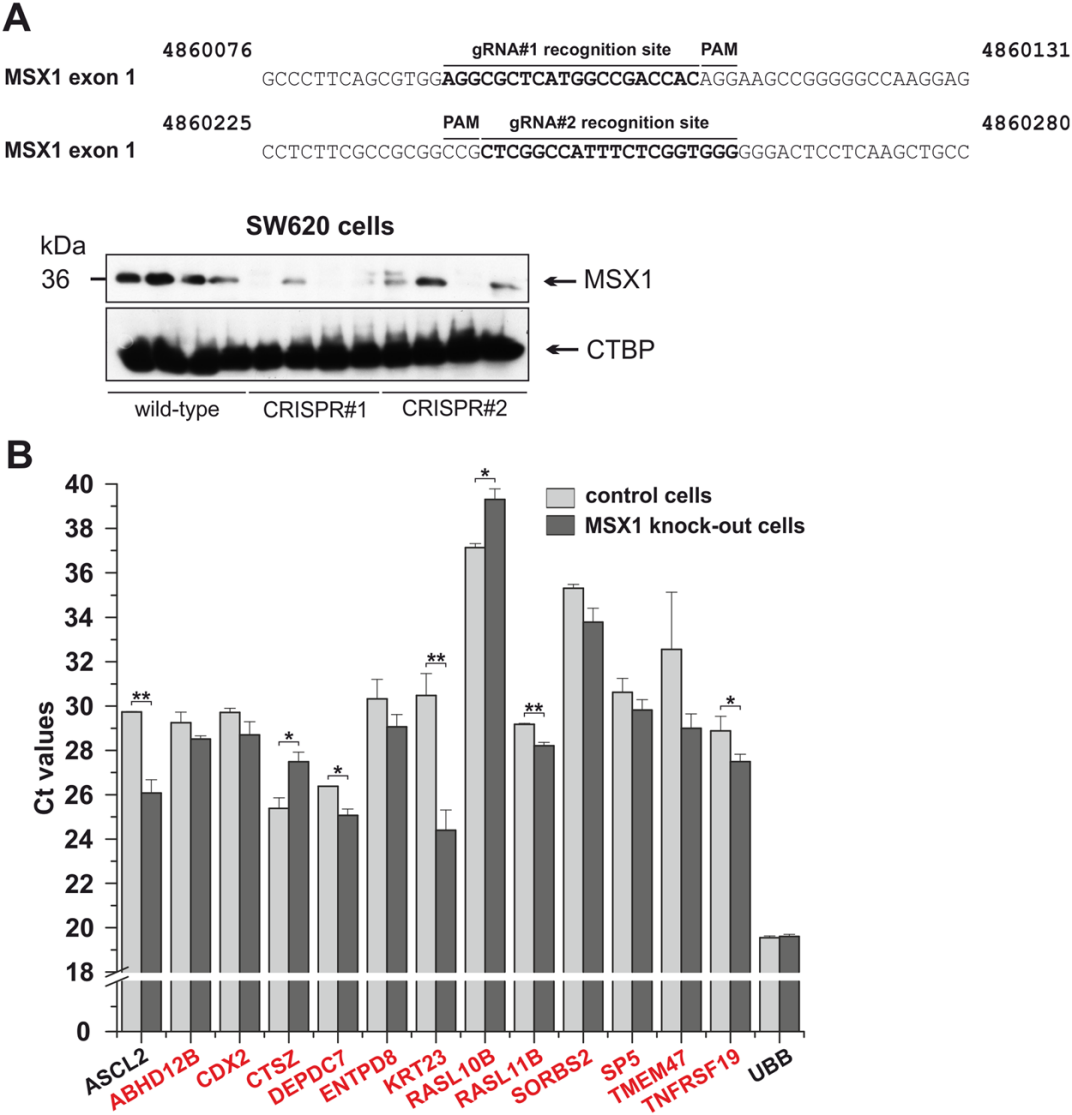
Generation and analysis of human CRC SW620 cells harboring truncations in the *MSX1* gene. (**A**) Top, DNA sequences showing guide RNA (gRNA) recognition sites (in bold) of CRISPR/Cas9-targeted regions in the first *MSX1* exon. The numbers above the sequence indicate nucleotide positions in the human genome assembly GRCh38:CM000666.2. Bottom, Western blotting of SW620 cell lysates confirmed reduction of MSX1 protein in cells harboring the modified *MSX1* alleles; four different cell clones are shown for both MSX1-specific gRNAs (indicated CRISPR#1 and CRISPR#2). Wild-type refers to control cells transduced with the lentiCRISPR vector encoding no gRNA. (**B**) Quantitative RT-PCR analysis of expression levels of the indicated genes in MSX1 knock-out and MSX1 wt SW620 cells. Genes identified in ref.^24^ are in red. RNA samples isolated from four MSX1-deficient and four MSX1-proficient cells clones was analyzed (each in technical triplicate). The diagram shows Ct values normalized to *β-actin* gene expression (Ct value arbitrary set to 17). Error bars indicate standard error of mean (SEM); *p < *0.05*; **p < *0.01*. *ABHD12B, Abhydrolase Domain Containing 12B; CDX2, Caudal Type Homeobox 2; CTSZ, Cathepsin Z; DEPDC7, DEP Domain Containing 7; ENTPD8, Ectonucleoside Triphosphate Diphosphohydrolase 8; KRT23, Keratin 23; RASL10B, RAS Like Family 10 Member B; RASL11B, RAS Like Family 11 Member B; SORBS2, Sorbin And SH3 Domain Containing 2; SP5, Sp5 Transcription Factor; TMEM47, Transmembrane Protein 47*.

To examine genes regulated by MSX1 in CRC cells, we disrupted *MSX1* in SW620 cells using the CRISPR/Cas9 system (Fig. 7A). SW620 cells were selected because they produce high levels of MSX1 protein and production of the MSX2 protein in these cells is negligible. The MSX1-deficient clones were viable and did not change their proliferation rate when compared to the control cells that were transfected with the “empty” targeting vector (Supplementary Fig. S11A). Moreover, cells with disrupted MSX1 formed tumors with the same efficiency as control cells when xenografted into immunodeficient mice (Supplementary Fig. S11B). Next, we performed expression profiling of MSX1-deficient SW620 cells; control total RNA samples were obtained from cells with intact *MSX1*. The profiling yielded 202 genes (including *ASCL2*) whose expression differed significantly. Enrichr-based analysis showed an overlap between the obtained gene set and a group of β-catenin-activated genes in SW480 cells identified by anti-β-CATENIN chromatin immunoprecipitation (ChIP)-sequencing (ChIP-seq)^24^ (Supplementary Table S8). Notice that SW480 and SW620 cells were derived from the primary tumor and lymph node metastasis, respectively, of a single CRC patient^25^. Interestingly, all overlapping genes were upregulated after MSX1 disruption (Fig. 7B and Supplementary Table S8). In the mouse intestine, transcription factor Ascl2 synergizes with β-catenin/Tcf complexes to activate expression of genes essential for ISC identity^26^. To test whether *ASCL2* gene regulatory regions are directly bound by MSX1, we performed ChIP using chromatin isolated from SW620 cells. As none of commercially available antibodies precipitated endogenous MSX1, we used the TALEN-mediated homology repair procedure to insert the EGFP sequence into the 5′ end of the *MSX1* locus (Supplementary Fig. S12A–D). In the targeted cells, the MSX1 protein N-terminally tagged with EGFP was produced. Subsequently, EGFP-specific antibodies were employed to immuneprecipitate EGFP-MSX1 fusion protein crosslinked to the chromatin. Nevertheless, the ChIP method did not indicate direct binding of MSX1 to the regulatory regions of the *ASCL2* gene (Supplementary Fig. S13). A similar approach was used to test MSX1 interaction with the *SP5* gene. Previous reporter gene, electromobility-shift, and ChIP-seq assays indicated that the *SP5* promoter region contains the functional motives regulated by the β-catenin/TCF4 complex in CRC cells^24,27^. Interestingly, we identified multiple MSX1 binding sites in the *SP5* promoter. Nevertheless, ChIP and reporter gene assay did not show direct binding (or regulation) of the *SP5* gene by MSX1 (Supplementary Fig. S13).

## Discussion

In the present study, we aimed to identify and characterize the genetic program related to cell transformation induced by the loss of the *Apc* tumor suppressor gene. For the experimental design, we used the conditional *Apc* allele and expression profiling of mouse intestinal epithelial cells obtained from the intestine before and after *Apc* gene inactivation, i.e., upon Wnt pathway hyperactivation.

One of the genes whose expression was robustly elevated in epithelial cells was Msx1, a nuclear protein belonging to a superfamily of the homeobox transcription factors. In the mouse, the *Msx1* gene was studied especially in the context of embryonic development, particularly in the development of teeth, brain and limbs^28–30^. Although the MSX1 role in human CRC has not yet been described, several studies identified *MSX1* promoter hypermethylation in CRC, suggesting MSX1 downregulation in tumor tissue^31,32^. Nevertheless, other studies showed data more consistent with our results, indicating that *MSX1* represents a marker of tumor tissue^33^. In addition, brief inspection of the Biogps portal, which offers a comprehensive analysis of gene expression data, showed that human *MSX1* gene expression is strongly upregulated in CRC (http://biogps.org/#goto=genereport&id=4487). Moreover, according to the data available in the COSMIC database^34^, *MSX1* was rarely mutated in human colon tumors. Therefore, it is tempting to speculate that in CRC, *MSX1* represents a tumor-promoting gene with some essential function. In a collection of human intestinal neoplasia, the *MSX1* mRNA levels were upregulated in nearly all of the tumors (Fig. 4B). This was seemingly surprising, since in the mouse *M*sx*1* overexpression was directly linked to the *Apc* loss. Nevertheless, recent studies employing massive parallel sequencing of tumor DNA indicated that genetic alterations resulting in aberrant Wnt signaling were found in more than 90% of CRC specimens^6^. This finding is in concordance with the observation that *MSX1* is overproduced in the majority of tumor specimens.

It was assumed that the expansion of the crypts observed upon Apc loss is associated with dysregulation of the Wnt-dependent cellular program for *stemness*^7^. Intriguingly, our data shows that the apparently continuous hyperplastic crypt compartment (in the Apc-deficient small intestine) is divided in the lower “standard” ISC part that flows into the Msx1-expressing ectopic crypt region located on the villi. The tissue organization of the intestinal epithelium arises from branching of the crypts that divide by fission during postnatal growth^35^. The program of crypt fission is (re)activated during tumorigenesis and drives expansion of the tumor tissue^36^. In addition, several laboratories reported that under some specific (pathological) situations, ectopic crypts are developed. In the mouse intestine, ectopic crypt formation was induced by transgenic production of secreted inhibitors of the BMP pathway noggin or gremlin 1. However, it happened without apparent crypt compartment expansion^37–40^. In addition, Madison and colleagues reported that inhibition of sonic hedgehog signaling perturbed the villus architecture and was accompanied by generation of ectopic crypts^38^. The ectopic crypts were also observed after simultaneous activation of the Wnt and NF-κB pathways. It was proposed that proliferating aberrant foci on the villus were induced by cell dedifferentiation^41^. We cannot rule out this possibility; however, following development of these structures at different time intervals (after Apc loss) rather suggests that ectopic crypts were produced from transformed cells exiting the hyperplastic crypt compartment (Fig. 3). An alternative explanation might be that the extracellular environment at the crypt-villus border is permissive for cell dedifferentiation. In human CRC, ectopic crypts represent a typical feature of so-called traditional serrated adenomas that comprise only a small fraction of colorectal malignancies (reviewed in)^42^. In contrast, we observed increased expression levels of *MSX1* in the majority of intestinal neoplasia, implicating a broader role of MSX1 in tumor initiation and/or progression.

Although Msx1 activation is clearly related to the loss of Apc, cells positive for Msx1 occur outside the stem cell compartment, implicating an additional regulatory mechanism involved in Msx1 gene expression. A study by Wallmen and colleagues suggested that TCF/β-catenin complexes are incapable to invade silent chromatin and, consequently, fail to initiate “de novo” transcription^43^. Interestingly, during the tooth, facial, limb, and neuronal development, or in human carcinoma cells, Msx1 expression is regulated by bone morphogenetic protein (BMP) signaling^44,45^. In the intestine, the BMP pathway is restricted to epithelial cells present on the villi, as the signaling in the crypt is locally inhibited by production of the BMP antagonists (reviewed in)^46^. Since some BMP signaling nuclear mediators can induce chromatin opening (reviewed in)^47^, we suggest that the combined activity of both Wnt and BMP pathways might trigger expression of a specific set of genes that includes Msx1.

How the loss of Msx1 affects formation of the ectopic crypt is not clear. The Msx1-deficient epithelium does not invaginate into the underlying connective tissue, but forms a continuous sheet of proliferating cells instead. This implies that ectopic crypt formation is (inter)connected with differentiation of intestinal epithelial cells, and the loss of Msx1 obviously disrupts this process (Figs 3 and 5A). Moreover, in the mouse small intestine, Msx1 loss caused morphological changes reminding conversion from tubular to villous-like adenomas. In humans, the villous adenomas represent a more progressed stage along the path to fully developed CRC. However, we did not observe any correlation between the adenoma type and *MSX1* expression (Supplementary Fig. S14).

*Msx1* gene inactivation in the mouse is embryonic lethal, and studies employing a conditional *Msx1* allele never focused on gut tissue. However, our results showed that Msx1 inactivation in healthy embryonic or adult intestinal epithelium exhibited no remarkable histological or anatomical changes. The absence of any (observable) phenotype might be attributed to the fact that the Msx1 absence is compensated for in the gut tissue by the related *Msx2* gene. The mouse *Msx* gene family consists of three members: *Msx1*, *Msx2*, and *Msx3* (reviewed in)^48^. Whereas *Msx3* expression is restricted to the dorsal neural tube^49^, *Msx1* and *Msx2* exhibit partially overlapping expression during embryonic development at diverse sites of epithelial-mesenchymal interactions such as in the limb buds, teeth buds, craniofacial bones, and heart^50^. *Msx2*^−/−^ mice are viable, although they display defects in the skin, teeth, skull, and mammary gland development and also impaired chondrogenesis and osteogenesis^51,52^. *Msx1*^−/−^ *Msx2*^−/−^ mice exhibit enhanced phenotype of the single mutants, indicating functional redundancy of the genes^29,53^. Additionally, MSX2 has been described as the target of Wnt/β-catenin signaling^54^ and in our experiments, especially those performed in human cells, we frequently observed virtually identical trends in *MSX1* and *MSX2* expression, suggesting their regulation by similar mechanisms (Fig. 2A–C). The *Msx1* gene expression level is low in the healthy intestine (Fig. 1C). Thus, the second explanation for the absence of any phenotypic changes after Msx1 loss is that the Msx1 function is not essential at homeostatic conditions. To test this possibility, we used two models of intestinal tissue damage, namely irradiation of experimental mice by sublethal X-ray doses^55^, and chemical damage to the epithelial layer by dextran sulfate sodium (DSS) administration in drinking water^56^. However, these tests did not show any difference in the extent of tissue damage or epithelial regeneration dynamics between the control (wt) and Msx1-deficient epithelium (Supplementary Fig. S15A,B).

Identifying genes that are regulated by Msx1 in the mouse Apc-deficient intestinal epithelia did not provide a “clear picture”. In fact, the significance criteria (|logFC| ≥ 1 and q-value ≤ 0.05) was reached only for the *Stk32b* gene in the Apc/Msx1 double-deficient colonic epithelium. We noticed that in *Apc*^*KO/KO*^ *Msx1*^*KO/KO*^ small intestine, *Msx1* expression was still substantially elevated in comparison to healthy tissue. This was not surprising since *Msx1* mRNA was virtually “not expressed” in the healthy intestine (normalized Ct values < 42) and residual Msx1 production (probably caused by incomplete *Msx1* gene recombination) was still detected in the small intestine of mice harboring the floxed *Msx1* alleles (Fig. 5A and Supplementary Fig. S6). Nevertheless, the levels of *Msx1* were reduced after Cre-mediated excision by more than four cycles, indicating that insufficient Msx1 inactivation was not the cause of the insignificant results of expression profiling (Supplementary Table S5). We assume that the expression profiling of the entire epithelia did not have sufficient resolution to encompass differences between Apc-deficient and Apc/Msxl double-deficient tissue. In humans, single-nucleotide polymorphisms (SNPs) in the *STK32B* gene were associated with non-syndromic oral cleft, indicating involvement of the gene in craniofacial development^57^. Strikingly, in both mice and humans, *Stk32b* and *Msx1* loci are located in close vicinity. This would imply that rather than direct regulation of *Stk32b* transcription by Msx1, the genetic manipulation within the *Msx1* locus might cause (aberrant) expression of *Stk32b*. However, since we did not observe any interconnection between *Msx1* gene manipulation and *Stk32b* expression levels in the mouse small intestine, the relation of the two genes remains unclear. Expression profiling of human CRC SW620 cells yielded more than 200 genes whose expression differed significantly after *MSX1* gene disruption. A subset of the genes were identified previously as β-catenin-regulated genes in related SW480 cells^24^. Since expression of all the genes was relieved in MSX1-deficient cells, we assumed that MSX1 might function as a repressor of (some) genes activated by Wnt/β-catenin signaling. However, we failed to prove the assumption experimentally. We anticipate that without unbiased ChIP-seq analysis, the identification of functional MSX1-binding sites in the genome is difficult to achieve.

It is evident that to identify the relationship between the results obtained in the mouse model and the tumorigenesis process in humans will require further experiments. Nevertheless, our data clearly demonstrate that some components of the transcriptional program triggered by the Apc loss are influenced by Msx1 and the program is related to the position of transformed cells in the affected tissue.

## Materials and Methods

### Experimental mice

Housing of mice and *in vivo* experiments were performed in compliance with the European Communities Council Directive of 24 November 1986 (86/609/EEC) and national and institutional guidelines. Animal care and experimental procedures were approved by the Animal Care Committee of the Institute of Molecular Genetics (no. 71/2014). *Apc*^*cKO/cKO*^ mice were obtained from the Mouse Repository (National Cancer Institute, Frederick, MD, US); *Villin-CreERT2* and *Villin-Cre* mice^14^ were kindly provided by S. Robine (Institut Curie, Centre de Recherche, Paris, France); *Apc*^*+/Min*^, *Lgr5-EGFP-IRES-CreERT2*, *Msx1*^*cKO/cKO*^, and immonodeficient NSG™ mice were purchased from the Jackson Laboratory (Bar Harbor, ME, US). Animals were housed in specific pathogen-free conditions.

### Cre-mediated gene recombination

For expression profiling, 6 weeks old mice were gavaged with 5 mg of tamoxifen (Sigma-Aldrich); 1 mg of tamoxifen was used in all other experiments. Tamoxifen was dissolved in ethanol (100 mg/ml) and prior to gavage combined with mineral oil. Mice were sacrificed by cervical dislocation at various time points after a single dose (100 μl) of tamoxifen solution. Intestines were dissected, washed in phosphate-buffered saline (PBS), fixed in 4% (v/v) formaldehyde (Sigma-Aldrich) in PBS overnight, embedded in paraffin, sectioned, and stained.

### Microarray analysis

Total RNA was isolated from *Apc*^*cKO/cKO*^ *VillinCreERT2* intestinal epithelium 2 and 4 days after administration of 5 mg of tamoxifen by gavage; control mice were administered with the solvent only (ethanol and mineral oil mixture). Four biological replicates were used for each time point. The RNA samples were analyzed using MouseRef-8 v2.0 Expression BeadChip (Illumina). Raw data were processed using the beadarray package of Bioconductor and analyzed as described previously^58,59^. Gene set enrichment analysis (GSEA) was performed using the Enrichr gene analysis tool^22,23^. Alternatively, total RNA was isolated from *Apc*^*cKO/cKO*^ and *Apc*^*cKO/cKO*^*Msx1*^*cKO/cKO*^ mouse small intestinal or colonic epithelium 7 days after administration of 1 mg of tamoxifen by gavage; mice administered with the solvent were used as controls. Four biological replicates were used for both mouse strains. Samples obtained from the small intestine were processed and analyzed as described above. RNA samples obtained from the colon were amplified and labeled using GeneChip WT PLUS Reagent Kit (Applied Biosystems) following the supplier’s protocol and starting with 250 ng of total RNA. Labeled single-stranded DNA was hybridized onto GeneChip Mouse Gene 2.0 ST arrays using GeneChip Hybridization, Wash, and Stain Kit (Applied Biosystems) following the supplier’s protocol. Arrays were scanned using GeneChip 3000 7 G Scanner (Affymetrix). Total RNA isolated from SW620 cell clones with the *MSX1* gene disrupted (n = 8) or intact (n = 4) was utilized. RNA samples were analyzed using Human HT expression BeadChip V4 (Illumina). Raw data were processed and analyzed as described above. The quality of all isolated RNA was checked using Agilent Bioanalyzer 2100; RNAs with RNA integrity number (RIN) above 8 were further processed.

### Human specimens

All methods used to collect the human specimens were performed in accordance with the relevant national and EU guidelines and regulations. The study was approved by the Ethics Committee of the Third Faculty of Medicine, Charles University in Prague. Informed consent have been obtained from all patients participating in the study. Paired samples of normal and neoplastic colonic tissue were obtained from patients undergoing either polypectomy of colonic adenomas or surgical resection of sporadic CRC (patient data are summarized in Supplementary Table S9). The tumor and corresponding normal colonic mucosa samples were immediately frozen and stored in liquid nitrogen. None of the patients underwent radiotherapy or chemotherapy before operation. Samples were processed as described in^60^. Briefly, frozen specimens were disrupted in 600 µl of lysis buffer by green ceramics beads and MagNA Lyser Instrument (Roche Life Science), and total RNA was extracted using RNeasy Mini kit (Qiagen) according to the manufacturer’s instructions. cDNA synthesis was performed in 20-µl reaction using 1 µg of total RNA, random hexamers and RevertAid reverse transcriptase (Thermo Fisher Scientific) according to the manufacturer’s protocol. PCR reactions were run in triplicates using LightCycler 480 Probes Master and Universal Probe Library (UPL) hydrolysis probes and LightCycler 480 Instrument (Roche Life Sciences). The primer pairs and corresponding UPL probes are listed in Supplementary Table S5. Threshold cycle (Ct) values for each triplicate were normalized by geometric average of housekeeping genes *UBB* and β*2-microglobulin*. The resulting values were averaged to obtain ΔCt values for biological replicates. Relative mRNA abundance (ΔCt in healthy tissue − ΔCt in neoplastic tissue) was correlated with the histological grade of tumor samples using the rank-order Spearman’s (*ρ*) and Kendall’s (*τ*) coefficient.

### Cell and organoid culture, 4-hydroxytamoxifen (4-OHT) treatment

HEK293, SW480, and SW620 cell lines were purchased from the American Type Culture Collection (Cat. Nos.: CRL-1573, CCL-228, and CCL-227). STF cells^15^ were kindly provided by Q. Xu and J. Nathans (Johns Hopkins University, Baltimore, MD). HEK293 and STF cells were maintained in Dulbecco’s Modified Eagle’s Medium (DMEM) supplemented with 10% fetal bovine serum (FBS; Gibco), penicillin, streptomycin, and gentamicin (all antibiotics were purchased from Invitrogen). SW480 and SW620 cells were maintained in Iscove’s Modified Dulbecco’s Medium (IMDM; Sigma-Aldrich) supplemented with 10% FBS, penicillin, streptomycin, gentamicin, NEA (Gibco), and Glutamax (Gibco). For Wnt pathway activation, HEK293 cells were treated with GSK3 inhibitor BIO (Sigma-Aldrich; final concentration 1 µM; the stock solution was prepared in DMSO; control cells were treated with solvent only) or by conditioned media (CM) obtained from cells producing the mouse Wnt3a ligand (dilution 1:1; cells were kindly donated by M. Maurice, University Medical Center Utrecht, Utrecht, The Netherlands); control cells were treated with the same dilution of CM obtained from cells non-producing the Wnt3a ligand. Both treatments were performed overnight. Small intestinal and colonic crypts obtained from *Msx1*^*cKO/cKO*^ *Villin-CreERT2* mice were isolated and cultured as described previously^61,62^. In culture media for colon organoids, recombinant Wnt3a was replaced by Wnt3a CM (dilution 1:1). For Cre-mediated recombination, organoids were treated with 1 µM 4-hydroxytamoxifen (Sigma-Aldrich). Control organoids were treated with the same volume of ethanol.

### RNA purification, cDNA synthesis, qRT-PCR

Total RNA from cell lines and mouse tumors was isolated using the TRI Reagent (Sigma-Aldrich), total RNA from epithelial cells was isolated by RNeasy Micro Kit or RNeasy Mini Kit (both kits were purchased from Qiagen); cDNA synthesis was performed in 20-µl reaction using random hexamers and 1 µg of total RNA (or the whole eluate when the RNeasy Micro Kit was used). RNA was reverse transcribed using RevertAid Reverse Transcriptase or MAXIMA Reverse Transcriptase (both were purchased from Thermo Fisher Scientific) following the manufacturer’s protocol. Quantitative RT-PCR was performed in triplicates using SYBR Green I Master Mix (Roche Life Science) and LightCycler 480 apparatus. For list of primers, see Supplementary Table S10.

### Immunoblotting, immunohistochemistry, immunocytochemistry, and antibodies

A detailed protocol of the immunoblotting procedure was described previously^63^. Full-length blots were included in Supplementary Fig. S16. Antigen retrieval of paraffin-embedded tissue was performed in 10 mM citrate buffer (pH 6.0) in a steam bath for 20 min. Primary antibodies were: anti-α-tubulin (rabbit polyclonal, kindly provided by L. Anděra), anti-β-actin (rabbit polyclonal, A2066, Sigma-Aldrich), anti-APC (mouse monoclonal, clone FE-9, Calbiochem), anti-GFP (mouse monoclonal, JL-8 632381, Clontech), anti-H3K27me3 (mouse monoclonal, #9733, Cell Signaling), anti-mucin 2 (rabbit polyclonal, sc-15334, Santa Cruz), anti-MSX1 (goat polyclonal, AF5045, R&D Systems), anti-PCNA (rabbit polyclonal, ab18197, Abcam). Peroxidase-conjugated anti-goat, anti-mouse, and anti-rabbit secondary antibodies were purchased from Sigma-Aldrich. Biotin-conjugated anti-goat, anti-mouse, and anti-rabbit secondary antibodies were purchased from Thermo Fisher Scientific. Peroxidase signal was enhanced by 30 min incubation with Vectastain ABC kit (Vector Laboratories) and developed in DAB solution (Vector Laboratories). For fluorescent microscopy, rabbit-anti-goat IgG (H + L) Alexa Fluor 488- and goat-anti-rabbit IgG (H + L) Alexa Fluor 594-conjugated secondary antibody (Thermo Fischer Scientific) were used. SW620 EGFP-MSX1 cells were stained as described previously^64^. Cells were counterstained with hematoxylin (Vector Laboratories) or DAPI nuclear stain (Sigma-Aldrich).

### ISH

ISH was performed with 8-µm paraffin sections according to the procedure described previously^65^. Mouse *Msx1* cDNA was purchased from Addgene (#21024). *Msx1* cDNA was directly cut out by restriction enzymes from the donor vector and ligated into pBluescript KS II (Stratagene); mouse *Ascl2* was PCR amplified from cDNA reverse transcribed from total RNA isolated from mouse small intestinal epithelial cells and cloned into pBluescript KS II (primers are listed in Supplementary Table S10). Plasmids were linearized and digoxigenin (DIG)-labeled RNA probes were synthesized using DIG RNA Labeling Mix (Roche Life Science) and T7 or T3 RNA polymerase (Thermo Fischer Scientific) for sense and antisense probes, respectively. Probes were purified using mini Quick Spin RNA Columns (ROCHE). Hybridized slides were developed using alkaline phosphatase-conjugated sheep anti-digoxigenin Fab antibody (Roche Life Science) in MATB buffer (100 mM maleic acid pH 7.5/150 mM NaCl/0.1% Tween) with 0.5% Blocking Reagent at 4 °C overnight (O/N). Signal was detected using a mixture of nitro-blue tetrazolium chloride [NBT; 100 mg/ml in 70% dimethyl formamide (DMF)] and 5-bromo-4-chloro-3′-indolyphosphate p-toluidine salt (BCIP; 50 mg/ml in 100% DMF). Sections were mounted in Mowiol (Sigma-Aldrich).

### RNAi

Cells were transfected with 10 nM small interfering RNAs (siRNAs) targeting the *CTNNB1* (β-catenin) gene (s437; Ambion), or control siRNAs (D001206-13-20; Dharmacon) using Lipofectamine RNAiMax (Invitrogen) according to the manufacturer’s protocol. Cells were re-transfected 2 days after the first transfection to increase the effect of RNA interference and harvested 2 days after the second transfection.

### Transfection, lentivirus production and purification

To produce lentiviral particles, one 10-cm Petri dish with HEK293FT cells (Invitrogen) was seeded at ~30% confluency one day before transfection in IMDM media. The transfection was performed using Lipofectamine^®^ 2000 (Thermo Fischer Scientific) in serum-free OptiMEM medium (Thermo Fischer Scientific); 48 hours after the transfection the culture medium was centrifuged at 3000 × g at 4 °C for 15 min to remove the cell debris. The lentiviral particles were precipitated from the supernatant using PEGit Virus Precipitation Solution (System Biosciences).

### Disruption of the human *APC* gene

Exon 15 of the *APC* gene was targeted in STF cells using the CRISPR/Cas9 system. Three different guide RNAs (gRNAs) were cloned into the lentiCRISPRv2 vector (Addgene, #52961) as described in the protocol published by Zhang and colleagues^66,67^. Guide RNAs were designed using the CRISPR Design Tool available at crispr.mit.edu; the list of the gRNA sequences is provided in Supplementary Table S10. Cells were co-transfected with lentiCRISPRv2 plasmid and pARv-RFP reporter^68^ containing the appropriate gDNA sequence recognized by gRNA. RFP^+^ cells were sorted into 96-well plates and expanded as single cell clones. Control cells were transfected with the empty (BsmBI digested and self-ligated) lentiCRISPRv2 vector and processed in an analogous way. Generation of STF cells harboring truncation in exon 10 of the *APC* gene was described previously^16^.

### Statistical analysis of data

The results of the qRT-PCR analysis were evaluated by Student’s *t* test. The relative mRNA abundance (ΔCt in healthy tissue - ΔCt in neoplastic tissue) was correlated with the histological grade of tumor samples using the rank-order Spearman’s (ρ) and Kendall’s (τ) coefficient. Datasets obtained using RNA microarrays were analyzed within the oligo and limma packages of Bioconductor^69–71^. Moderated *t*-test was used to detect differentially expressed genes (DEGs) between experimental groups: at least two-fold change difference in gene expression and Storey’s q-value^58^ less than 0.05 were considered significant.

### Raw expression data repository

Minimum Information About a Microarray Experiment (MIAME) compliant data were deposited to the ArrayExpress database (E-MTAB-6915, E-MTAB-6930, E-MTAB-6928, and E-MTAB-6909).

Additional Materials and Methods are given in Supplementary Information.

## Supplementary information

Supplementary Materials_Methods_Figure and Table Legends_ Figures

Supplementary Tables
