# Author Correction: Msx1 loss suppresses formation of the ectopic crypts developed in the Apc-deficient small intestinal epithelium

**DOI:** 10.1038/s41598-019-55963-5

**Published:** 2019-12-24

**Authors:** Monika Horazna, Lucie Janeckova, Jiri Svec, Olga Babosova, Dusan Hrckulak, Martina Vojtechova, Katerina Galuskova, Eva Sloncova, Michal Kolar, Hynek Strnad, Vladimir Korinek

**Affiliations:** 1 Institute of Molecular Genetics of the Czech Academy of Sciences, Videnska 1083, 142 20, Prague, 4 Czech Republic; 2 Faculty of Science, Charles University in Prague, Albertov 6, 128 43, Praha, 2 Czech Republic; 3 Department of Radiotherapy and Oncology, Third Faculty of Medicine, Charles University, Prague, Srobarova 50, 100 34, Prague, 10 Czech Republic

Correction to: *Scientific Reports* 10.1038/s41598-018-38310-y, published online 07 February 2019

The original version of this Article contained an error in Affiliation 1, which was incorrectly given as ‘Institute of Molecular Genetics of the ASCR, v. v. i., Videnska 1083, 142 20 Prague 4, Czech Republic’.

The correct affiliation is listed below:

Institute of Molecular Genetics of the Czech Academy of Sciences, Videnska 1083, 142 20 Prague 4, Czech Republic

This error has now been corrected in the HTML and PDF versions of the Article.

