# Supplementary material for: Msx1 loss suppresses formation of the ectopic crypts developed in the Apc-deficient small intestinal epithelium
Source: Sci Rep. 2019 Feb 7;9:1629. doi: 10.1038/s41598-018-38310-y (PMC6367488; doi:10.1038/s41598-018-38310-y)

# Supplementary information

**Title: Msx1 loss suppresses formation of the ectopic crypts developed in the Apc-deficient small intestinal epithelium**

**Authors:** Monika Horazna, Lucie Janeckova, Jiri Svec, Olga Babosova, Dusan Hrckulak, Martina Vojtechova, Katerina Galuskova, Eva Sloncova, Michal Kolar, Hynek Strnad, and Vladimir Korinek

## Supplementary Materials and Methods

### Tagging and disruption of the *MSX1* gene in human cells

To generate cells producing MSX1 fused at its N-terminus with EGFP (EGFP-MSX1), SW620 cells were co-transfected with the lentiCRISPRv2 vector containing gRNA targeting exon 1 of the *MSX1* gene together with a synthetic template (purchased from GenScript) for homology-directed repair of the *MSX1* locus and corresponding pARv-RFP reporter vector (Addgene #60021). RFP$^+$ cells were sorted into 96-well plates and expanded as single cell clones. Individual clones were PCR screened for the presence of correct insertion of the exogenous template, genotyping primers are provided in supplementary Table S10. The scheme of the targeted locus can be found in supplementary Fig. S8. Full sequences of the construct used are available upon request. To disrupt the *MSX1* gene, first SW620 cells stably producing the Cas9 enzyme were generated by retroviral transduction using the lentiCas9-Blast vector (#52962, Addgene) and batch-selected using 10 μg/ml blasticidin (Gibco). The resulting SW620/Cas9-Blast cells were transduced with lentiGuide-Puro (#52963, Addgene) viruses targeting the first exon of the *MSX1* gene and selected using 6 μg/ml puromycin (Thermo Fischer Scientific). The transduction was performed in four technical replicates using two lentiGuide-Puro plasmids targeting different sites in the *MSX1* first exon; cells transduced with empty (BsmBI digested and selfligated) lentiGuide-Puro plasmid were used as a control. Correct targeting was verified by PCR amplification and sequencing. Selected polyclonal cultures were analyzed by Western blotting.

### Luciferase reporter assay

The assay was performed using the Dual-Glo Luciferase Assay System and GloMax® 20/20 Luminometer (all from Promega). To test the *SP5* promoter activity, a region of the promoter containing TCF/β-catenin and MSX1 binding sites was PCR-amplified from human genomic DNA and cloned into the pTA-Luc vector (Invitrogen); primers are listed in supplementary Table S10. The luciferase assay was performed in SW620 cells (wild-type or *MSX1*-defficient) and in HEK293 cells co-transfected with a DNA mixture containing increasing amounts of the expression vector encoding FLAG-tagged mouse Msx1, the SP5 reporter, and Renilla expression plasmid (pRL-TK; Promega). In addition, to activate Wnt signaling in HEK293 cells, transfection mixtures also included a plasmid encoding the stabilized form of β-catenin protein mutated at its serine 33 residue{Valenta, 2011 #8457}. All luciferase assays were performed in triplicates; three MSX1-deficient and three control clones of SW620 cells were used. Luciferase activity was normalized to Renilla.

**DSS treatment, total body irradiation**

*Msx1$^{cKO/cKO}$ Villin-Cre* mice were used for both experiments. In the colon, intestinal damage was inflicted by 2% (w/v) DSS (MP Biomedicals; MW36–50 kDa;) in drinking water for 5 days. Colons were collected upon DSS withdrawal at day 2 (acute colitis), day 5 (beginning of the regenerative phase), and day 9 (late regenerative phase). For the total body irradiation experiment, mice were exposed to single total body irradiation of 5 Gy during 5 minutes. The X-RAD 225 XL instrument (PRECISION X-Ray Inc.) with 0.5 mm Cu (F5) filter was used; voltage and current of the X-ray tube was set to 225 kV/13.3 mA. Mice were sacrificed 2, 4, and 6 days after irradiation and small intestines were collected for analysis. For each time point (for both DSS or irradiation experiment), two Cre$^{+}$ and two Cre$^{-}$ littermates were used; untreated mice of the same genotype were used as a control.

**ChIP**

SW620 cells (10 million cells per ChIP) producing EGFP-MSX1 fusion protein were crosslinked by 1% formaldehyde (Sigma-Aldrich), lysed using HighCell ChIP kit (C01010062, Diagenode), and the isolated chromatin was sheared by sonication with three runs of 10 cycles (30 s ON/30 s OFF) at high

power setting using Bioruptor sonicator (Diagenode). The sheared chromatin from SW620 EGFP-MSX1 or parental cells was incubated with GFP-Trap®_MA (Chromotek) magnetic beads and further processed as described in the protocol provided by the manufacturer. Antibodies against H3K4me3 (C15410003) and negative control rabbit IgG were purchased from Diagenode. After decrosslinking, the DNA was isolated using QIAquick PCR purification kit (Qiagen) and the quantitative PCR reactions were performed on a LightCycler 480 Instrument

After decrosslinking, DNA was isolated using QIAquick PCR purification kit (Qiagen). Occupancy of the *ASCL2* and *SP5* gene regulatory regions by MSX1 was assayed by PCR; primers are listed in supplementary Table S10. The recovery of the particular promoter locus was calculated as the relative amount of immunoprecipitated DNA compared to input DNA (percentage of input).

**Cell viability test**

Cells were seeded to 96-well Assay Plates (Corning) at approximately 12.5% confluency in 100 µl of IMDM. Six hours after seeding, 10 µl of alamarBlue reagent (Thermo Fisher Scientific) was added to each well. After 60 min, the fluorescence intensity was measured in the culture medium using EnVision® Multilabel Reader (PerkinElmer). The measurement was repeated every 24 hours for 5 days.

**Xenotransplantation**

SW620 single cell clones with the *MSX1* gene knock-out (n=3) and control cells with intact *MSX1* (n=3) were cultured to 90-100% confluency, harvested and resuspended in PBS. NSG™ mice were injected with 1 million cells (in 100 µl PBS) into the lumbar back area. Mice were sacrificed 28 days after injection, tumors were resected and weighed.

**Supplementary supplementary Table legends**

**Table S1. List of differentially expressed genes in the small intestinal epithelium of *Apc*$^{cKO/cKO}$ *Villin-CreERT2* mice 2 or 4 days after tamoxifen administration.**

**Table S2. List of differentially expressed genes in the colonic epithelium of *Apc*$^{cKO/cKO}$ *Villin-CreERT2* mice 2 or 4 days after tamoxifen administration.**

**Table S3. Quantitative RT-PCR analysis of *MSX1* expressions in human cells upon Wnt pathway activation or in APC-deficient cells.** Corresponding diagrams and additional details are given in Fig. 2.

**Table S4. List of differentially expressed genes in the small intestinal epithelium of *Msx1*$^{cKO/cKO}$ *Apc*$^{cKO/cKO}$ *Villin-CreERT2* and *Apc*$^{cKO/cKO}$ *Villin-CreERT2* mice 7 days after tamoxifen administration.**

**Table S5. Quantitative RT-PCR analysis of gene expression in the small intestinal epithelium of *Msx1*$^{cKO/cKO}$ *Apc*$^{cKO/cKO}$ *Villin-CreERT2* and *Apc*$^{cKO/cKO}$ *Villin-CreERT2* mice 7 days after tamoxifen administration.**

**Table S6. List of differentially expressed genes in the proximal colon of *Msx1*$^{cKO/cKO}$ *Apc*$^{cKO/cKO}$ *Villin-CreERT2* and *Apc*$^{cKO/cKO}$ *Villin-CreERT2* mice 7 days after tamoxifen administration.**

**Table S7. Quantitative RT-PCR analysis of gene expression in the proximal colonic epithelium of *Msx1*$^{cKO/cKO}$ *Apc*$^{cKO/cKO}$ *Villin-CreERT2* and *Apc*$^{cKO/cKO}$ *Villin-CreERT2* mice 7 days after tamoxifen administration.**

**Table S8. List of differentially expressed genes in the MSX1-deficient SW620 cells.**

**Table S9. Patient summary and histopathological grade of colorectal neoplasia specimens.** The *MSX1* gene expression level was analyzed in the total amount of 72 neoplasia specimens and compared to the gene expression level in the matching healthy mucosa.

**Table S10. List of DNA oligonucleotides used in the study.**

**Supplementary Figure legends**

**Figure S1. Generation of STF cells producing truncated APC.** (**A**) DNA (top) and protein (bottom) sequences showing the CRISPR/Cas9-targeted region in exon 15 of the *APC* gene. Numbers indicate the positions in the translated portion of *APC* cDNA or protein. The guide RNA recognition sequence (gRNA) and protospacer adjacent motif (PAM) are in bold and overlined. A CRISPR/Cas9-mediated cut and subsequent repair of genomic DNA by non-homologous end joining generated 4- and 5-nucleotide deletions (depicted in "targeted allele 1" and "targeted allele 2", respectively) in the coding sequence of the *APC* alleles. Both DNA alterations resulted in a premature stop of translation. Amino acid residues translated upon the frameshift are shown in red; asterisks indicate premature termini of the protein. (**B**) Western blotting of STF cell lysates with anti-APC antibody confirmed production of truncated APC protein in STF cells harboring truncated alleles of the *APC* gene. An anti-HSP90 antibody was used as a loading control. (**C**) Luciferase reporter assay in STF cells harboring inactivating mutations in exons 10 and 15 of the *APC* gene. Relative luciferase units (RLU) indicate the level of luciferase activity normalized to protein concentration in cells lysed used for luciferase activity measurement. Samples were measured in technical duplicates; the experiment was performed in two replicates, representative results are shown. Error bars indicate SDs.

**Figure S2. Immunohistochemical staining of Msx1 and PCNA in *Apc$^{cKO/cKO}$ Villin-CreERT2* and *Apc$^{cKO/cKO}$ Msx1$^{cKO/cKO}$ Villin-CreERT2* small intestine 7 days after tamoxifen administration.** In *Apc$^{cKO/cKO}$ Villin-CreERT2* mice, ectopic crypts containing Msx1- and PCNA-positive cells are positioned orthogonally to the crypt axis (red arrowheads). In contrast, PCNA-positive proliferating cells reach tips of the villi and ectopic crypts diminish in Msx1-deficient epithelium (green arrowheads). For each mouse strain, sections from four different animals were used for staining with both antibodies. Sections were stained using 3,3′-diaminobenzidine (DAB) stain (brownish nuclear precipitate); they were counterstained with hematoxylin (blue nuclear stain). Boxed areas are magnified at the right of the corresponding image. Scale bar: 0.3 mm.

**Figure S3. Proliferating tumor cells are Msx1-positive.** Immunohistochemical colocalization of Msx1 and PCNA in (micro)adenomas (red arrowheads) formed in the small intestine of *Apc*$^{cKO/cKO}$ *Lgr5-EGFP-IRES-CreERT2* mice. The tissue was analyzed 4, 7, and 21 days after tamoxifen administration using DAB staining. Scale bar: 0.3 mm.

**Figure S4. Msx1 mRNA is enriched in tumors developed in *Apc*$^{+/Min}$ mice.** (**A**) Immunohistochemical detection of Msx1 protein in two small intestinal and colonic neoplastic lesions (red arrowheads) of an *Apc*$^{+/Min}$ mouse. (**B**) *In situ* hybridization of *Msx1* mRNA in the small intestinal and colonic tumor developed in an *Apc*$^{+/Min}$ mouse. Msx1-positive tissue (violet signal) is indicated by red arrowheads. The sections in A were counterstained with hematoxylin; scale bar: 0.15 mm (A), 0.3 mm (B). (**C**) Quantitative RT-PCR analysis of total RNA isolated from tumors developed in three 20 weeks old *Apc*$^{+/Min}$ mice. The *Msx1* Ct values were normalized to *β-actin* gene expression (the *β-actin* gene Ct value was arbitrarily set to 17). The diagrams show relative *Msx1* expression in tumor samples compared to *Msx1* expression in control healthy mucosa (arbitrarily set to 1). The qRT-PCR analysis was performed in technical triplicates; error bars indicate SDs. In animal #3, no colonic tumor was detected.

**Figure S5. Msx1 loss does not affect growth or morphology of intestinal organoids.** Stereomicroscopic images of organoids derived from the small intestine of *Msx1*$^{cKO/cKO}$ *Villin-Cre* (*Msx1*$^{KO/KO}$) and *Villin-Cre* (*Msx1*$^{+/+}$) mice. The images were taken at indicated days after crypt isolation (A) or after the first passage (B). Scale bar: 0.150 mm.

**Figure S6. The absence of Msx1 changed the morphological features of hyperplastic epithelium.** Immunodetection of PCNA in *Apc*$^{cKO/cKO}$ *Villin-CreERT2* (*Apc*$^{KO/KO}$ *Msx1*$^{+/+}$) and *Apc*$^{cKO/cKO}$ *Msx1*$^{cKO/cKO}$ *Villin-CreERT2* (*Apc*$^{KO/KO}$ *Msx1*$^{KO/KO}$) mice seven days after tamoxifen administration. Three representative images of the middle part of the small intestine for each genotype are shown. Notice that in *Apc*$^{KO/KO}$ *Msx1*$^{KO/KO}$ epithelium, PCNA-positive proliferative cells reach tips of the villi (green arrowheads in insets). In contrast, ectopic crypts containing Msx1-positive cells are formed on

the villi of *Apc*$^{KO/KO}$ *Msx1*$^{+/+}$ animals (red arrowheads in insets). Notice that the gene recombination is not complete and Msx1-positive cells are occasionally detected on the villi of *Apc*$^{KO/KO}$ *Msx1*$^{KO/KO}$ mice. Sections were counterstained with hematoxylin; scale bar: 0.150 mm.

**Figure S7. Immunohistochemical staining of histone 3 trimethylation on lysine 27 (H3K27me3) shows a decrease in numbers of differentiated cells in Apc/Msx1-double-deficient epithelium.** *Apc*$^{cKO/cKO}$ *Villin-CreERT2* and *Apc*$^{cKO/cKO}$ *Msx1*$^{cKO/cKO}$ *Villin-CreERT2* mice were sacrificed 7 days after a single intraoral dose of tamoxifen (the specimens are indicated as *Apc*$^{KO/KO}$ *Msx1*$^{+/+}$ and *Apc*$^{KO/KO}$ *Msx1*$^{-/-,}$ respectively); control mice (genotype: *Apc*$^{cKO/cKO}$ *Msx1*$^{cKO/cKO}$ *Villin-CreERT2*) were gavaged with the solvent only. Differentiated cells were visualized in the small intestine by anti-H3K27me3 staining (green fluorescence signal). Notice that in *Apc*$^{cKO/cKO}$ *Msx1*$^{cKO/cKO}$ *Villin-CreERT2* animals, the differentiated cells (red arrowheads) are positioned at the very end of the villi. The sections were counterstained with DAPI nuclear stain (blue fluorescence); scale bar: 0.15 mm (control) and 0.3 mm (*Apc*$^{KO/KO}$ *Msx1*$^{+/+}$ and *Apc*$^{KO/KO}$ *Msx1*$^{-/-}$).

**Figure S8. Decreased expression of cell differentiation markers in the Apc/Msx1-deficient small intestine.** Quantitative RT-PCR analysis of *Msx1*$^{cKO/cKO}$ *Apc*$^{cKO/cKO}$ *Villin-CreERT2* (*Apc*$^{KO/KO}$ *Msx1*$^{KO/KO}$) and *Apc*$^{cKO/cKO}$ *Villin-CreERT2* (*Apc*$^{KO/KO}$ *Msx1*$^{+/+}$) small intestinal epithelium 7 days after tamoxifen administration; control samples were obtained from *Msx1*$^{cKO/cKO}$ *Apc*$^{cKO/cKO}$ *Villin-CreERT2* mice gavaged with the solvent only. RNA samples obtained from three tamoxifen-treated mice of each strain and four control animals were analyzed; qRT-PCR reactions were run in technical triplicates. The obtained Ct values were normalized to *β-actin* gene expression. The gene expression level in control mice was set to 1; error bars indicate SDs. *CHGA*, chromogranin A; *SI*, sucrose isomaltase. Error bars indicate SDs; significance level ($p < 0.05$) was not reached for any of the indicated genes. The fold change of the *Msx1* mRNA levels in *Apc*$^{KO/KO}$ *Msx1*$^{+/+}$ and *Apc*$^{KO/KO}$ *Msx1*$^{KO/KO}$ mice (when compared to control mice) was 12337.82 and 1114.82, respectively. In the same comparison, the fold change of

the *Msx2* mRNA levels was 1523.82 and 1543.41, respectively. Due to the high values, these results were not included in the diagrams. All numeric values are given in supplementary Table S5.

**Figure S9. Immunohistochemical detection of PCNA-positive cells indicates efficient Cre-mediated recombination.** PCNA was visualized in the middle part of the small intestine and colon of $Apc^{cKO/cKO}$ *Villin-CreERT2* mice 7 days after tamoxifen injection. Scale bar: 0.3 mm.

**Figure S10. Expression profiling of the Apc- and Apc/Msx1-deficient colon.** Total RNA samples were isolated from the mucosa obtained from the proximal colon of $Msx1^{cKO/cKO}$ $Apc^{cKO/cKO}$ *Villin-CreERT2* ($Apc^{KO/KO}$ $Msx1^{KO/KO}$) and $Apc^{cKO/cKO}$ *Villin-CreERT2* ($Apc^{KO/KO}$ $Msx1^{+/+}$) mice 7 days after tamoxifen administration; control samples were obtained from $Msx1^{cKO/cKO}$ $Apc^{cKO/cKO}$ *Villin-CreERT2* mice gavaged with the solvent only. Top, results of DNA microarray analysis for the *Msx1* gene. The boxed areas correspond to the second and third quartiles; the median is indicated by the crossline. Bottom, qRT-PCR analysis of selected genes identified by DNA microarray-based expression profiling. The gene expression level in control mucosa was set to 1. PCR reactions were run in triplicates; error bars indicate SDs. The fold change of *Msx1* mRNA in $Apc^{KO/KO}$ $Msx1^{+/+}$ and $Apc^{KO/KO}$ $Msx1^{KO/KO}$ mice (when compared to control mice) was 862.66 and 76.74, respectively. In the same comparison, the fold change of mRNA encoding Msx2 was 840.45 and 1344.64, respectively. Due to the high values, these results were not included in the diagram. All numeric values are given in supplementary Table S7; ***, $p < 0.001$. *Bves, Blood Vessel Epicardial Substance; Mdga1, MAM Domain Containing Glycosylphosphatidylinositol Anchor 1; Mtus2, Microtubule Associated Scaffold Protein 2; Mylk3, Myosin Light Chain Kinase 3; Slc5a6, solute carrier family 5 member 6; Stk32b, Serine/Threonine Kinase 32B; Trpm5, transient receptor potential cation channel, subfamily M, member 5; Ttn, titin.*

**Figure S11.** (**A**) Cell viability assay of four MSX1-deficient and two control (Ctrl#1 and Ctrl#2) clones of SW620 cells. The relative fluorescence intensity of the alamarBlue metabolic product is indicated; the intensity at day 0 was set to 1. The experiment was performed in technical triplicates; average values of the relative fluorescence intensity are plotted. (**B**) Tumor formation after xenotransplantation of

MSX1 wt and MSX1-deficient SW620 cells into immunodeficient (NSG$^{TM}$) mice. The animals were injected into the lumbal back area using $1 \times 10^6$ cells. Mice were sacrificed 28 days later, growing tumors were resected and weighed. The experiment was repeated twice using technical triplicates for each cell clone. The diagram shows weights of individual tumors combined from both experiments. The boxed areas correspond to the second and third quartiles; the median and mean value is indicated by crossline or empty square, respectively. The range of the values is given by "whiskers" above and below each box.

**Figure S12. Generation of SW620 cells harboring a modified *MSX1* allele producing EGFP-MSX1 fusion protein.** (**A**) The diagram shows CRISPR/Cas9-based genome editing of the *MSX1* locus; exons are depicted by black boxes, untranslated regions at the 5' and 3' end of exons 1 and 2 are indicated as white boxes. CRISPR/Cas9 recognizes and cleaves a sequence in the first exon of the gene (pink asterisk). The affected locus is repaired by homologous recombination using an exogenous template carrying a portion of the *MSX1* gene including the EGFP sequence (green box). The CRISPR/Cas9 recognition site in the exogenous template was wobbled (empty pink asterisk) to prevent its cleavage. Primary screen of clones for the presence of the EGFP sequence was done by PCR analysis of genomic DNA using internal EGFP primers originally designed for qRT-PCR (not shown in the diagram). Correct targeting (at both ends of the template integration site) was verified by sequencing of PCR products amplified from genomic DNA using two primer pairs: P1 and P2, P3 and P4. Primer positions are depicted by black arrows; one primer from each set (P1 and P4) primes in a sequence that is not present in the exogenous template. (**B**) Fluorescent immunocytochemical staining with an anti-GFP antibody (green) visualized nuclear localization of endogenously produced EGFP-MSX1 fusion protein in SW620 cells. Cells were counterstained with DAPI nuclear stain (blue). Magnification: 1000 ×. (**C**) Western blotting of SW620 and SW620/EGFP-MSX1 cell lysates using an anti-GFP antibody confirmed endogenous production of EGFP-MSX1 fusion protein in SW620 cells. (**D**) Western blotting of cell lysate (input), supernatant, and precipitate (beads) retained on GPF-Trap beads. Full-length blots were included in supplementary Fig. S10C.

**Figure S13. Analyzed regulatory regions of the *ASCL* and *SP5* genes.** (**A**) Top, position of potential MSX1 binding sites (pink boxes) in the *ASCL2* locus; untranslated regions and exons are depicted by empty or filled boxes, respectively. The nucleotides surrounding the consensus MSX1 binding sequence are in lowercase letters. ChIP analysis was performed using indicated primer pairs (P1-P6). Bottom, the *SP5* gene regulatory sequence tested in luciferase reporter and ChIP assays. The sequence indicating position of the peak obtained upon anti-β-CATENIN ChIP in SW480 cells (ref. No. 24) is in bold. TCF/β-catenin binding sites are depicted in magenta, MSX1 binding sites are in blue. The sequence of primers used for ChIP is overlined. Transcription start site is indicated by a red arrow; translation initiation codon is highlighted. The sequence maps to nucleotides 170,714,781-170,715,617 on chromosome 4 in GRCh38 human genome assembly. (**B**) ChIP of putative MSX1-binding sites in the *ASCL2* and *SP5* promoter as depicted in (A). Chromatin obtained from parental SW620 cells or SW620 cells producing EGFP-MSX1 fusion protein was precipitated using indicated antibodies; IgG, negative control antibody. ChIP analysis of housekeeping genes β-actin (*ACTB*) and glyceraldehyde-3-phosphate dehydrogenase (*GAPDH*) using the H3K4me3-specific antibody (recognizing chromatin of transcribed genes) was employed as a positive control. The recovery of the particular promoter region was calculated as the relative amount of immunoprecipitated DNA compared to input DNA (percentage of input).

**Figure S14. MSX1 expression in tubular (n = 20), tubulovillous (n = 31), and villous (n = 3) adenomas.** Threshold cycle values for each technical triplicate were normalized by geometric average of ubiquitin B (*UBB*) and β2-microglobulin reference genes. The resulting values were averaged to obtain ΔCt values for biological replicates. The relative mRNA abundance/expression 2^(mean ΔCt in healthy tissues − ΔCt of the adenoma) was correlated with the adenoma structure/appearance. Welch's two sample t-test was used to compare means of the gene expression among all groups; however, statistical significance was not reached. The boxes correspond to the first and third quartiles; the median of relative expression values for each category is indicated as the black square. The range of the values is given by "whiskers" above and below each box. Outliers are indicated by black triangles.

**Figure S15. DSS treatment and total body irradiation of *Villin-Cre* (*Msx1*$^{+/+}$) and *Msx1*$^{cKO/cKO}$ *Villin-Cre* (*Msx1*$^{KO/KO}$) mice.** (**A**) Acute colitis and regeneration after DSS treatment was monitored by immunohistochemical staining of PCNA-positive cells. Representative images obtained 2, 5, and 9 days after DSS withdrawal are shown. (**B**) Mice of the indicated genotype were exposed to a single dose of irradiation (5 Gy) and PCNA-positive cells in the small intestine were detected immunohistochemically at the indicated time point after irradiation. Notice the decreased number of proliferating cells in the crypts two days after irradiation (red arrowheads). The proliferating compartment in the crypts is restored six days after irradiation. Scale bar: 0.15 mm.

**Figure S16. Full-length immunoblots.** (**A**) Detection of wt and truncated APC in STF cells. The blot was co-stained with an APC- and HSP90-specific (loading control) antibody. Regions shown in supplementary Fig. S1B are indicated by dotted rectangles. **(B**) MSX1 and CTBP1 (loading control) detection in SW620 cells. Dotted rectangles indicate regions shown in Fig. 7A. The blot was cut into two parts (the position of the cut is indicated by red arrow) and stained with indicated antibodies. (**C**) Detection of EGFP-MSX1 fusion protein in SW620 cells. Areas indicated by dotted rectangles are shown in supplementary Figs. S12C and S12D. Azure c280 (Azure Biosystems) was used for digital chemiluminescent detection in (A). Other images were obtained using exposition on films.

# Supplementary Figure S1

**A**

| | | PAM / gRNA recognition site | |
|---|---|---|---|
| **wt APC cDNA** | 3941 | GGTCAGCTGAAGAT**CCTGTGAGCGAAGTTCCAGCAGT**GTCACAGCACCCTAGAAC | 3995 |
| **targeted allele 1** | | GGTCAGCTGAAGATCCTGCGA----AGTTCCAGCAGTGTCACAGCACCCTAGAAC | 3991 |
| **targeted allele 2** | | GGTCAGCTGAAGATC-----AGCGAAGTTCCAGCAGTGTCACAGCACCCTAGAAC | 3990 |

| | | | |
|---|---|---|---|
| **wt APC protein** | 1371 | EDPVSEVPAVSQHPRTKSSRLQGSSLSSESARHKAVEFSSGAKSPSKSGAQTPKS<br>PPEHYVQETPLMFSRCTSVSSLDSFESRSIASSVQSEPCSGMVSG | 1471 |
| **truncated protein 1** | | EDPAKFQQCHSTLEPNPADCRVLVYLQNQPGTKLLNFLQERNLPPKVVLRHPKVH<br>LNTMFRRPHSCLADVLLSVHLIVLRVVRLPAPFRVNHAVEW* | 1467 |
| **truncated protein 2** | | EDQRSSSSVTAP* | 1382 |

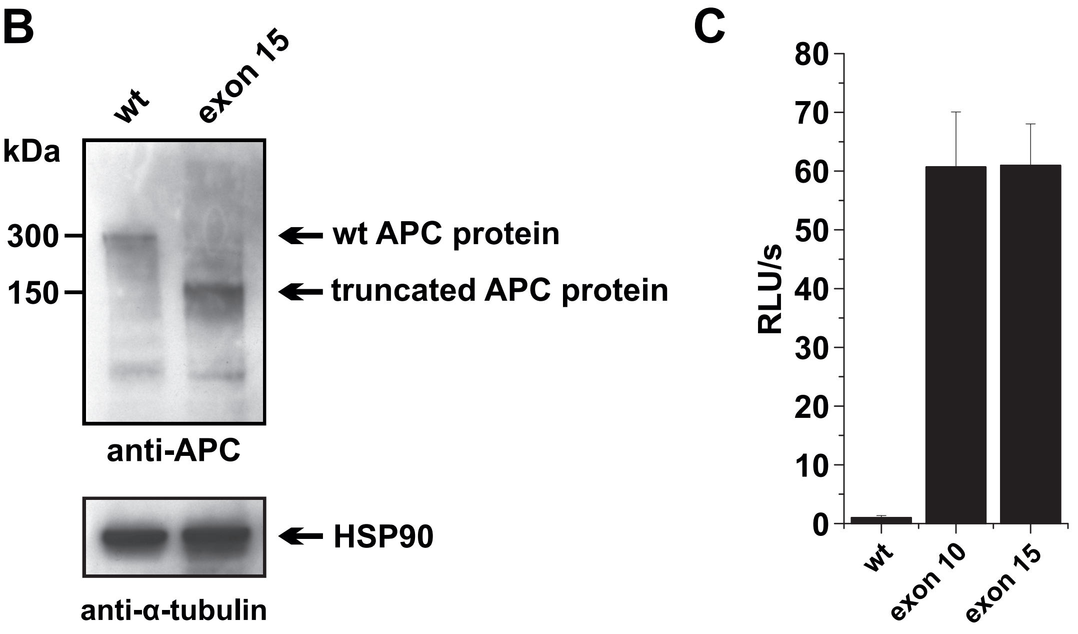

# Supplementary Figure S2

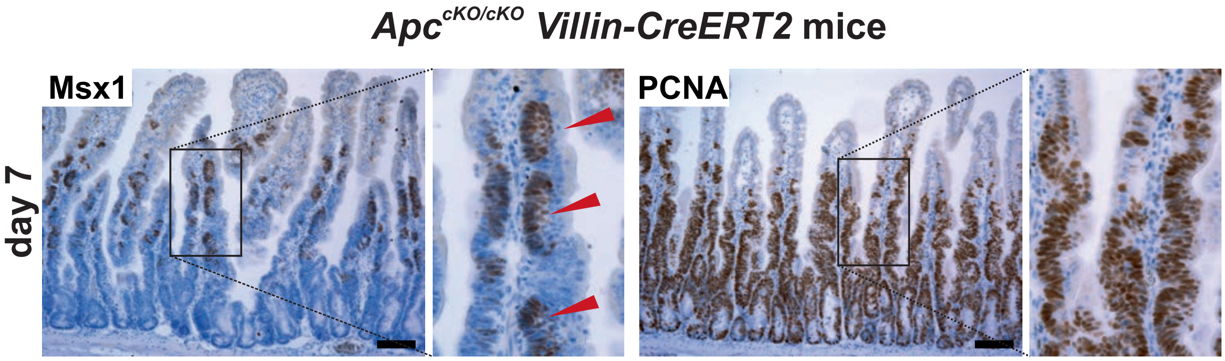

# Supplementary Figure S3

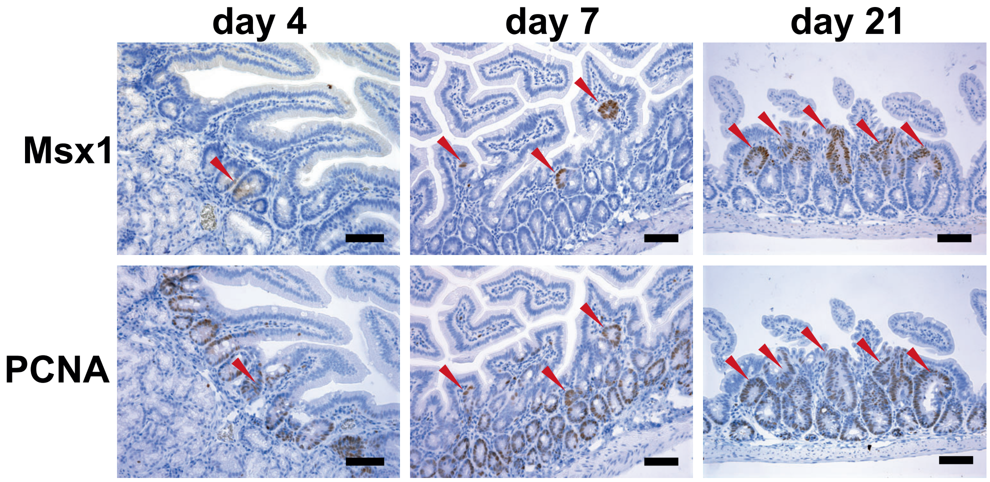

# Supplementary Figure S4

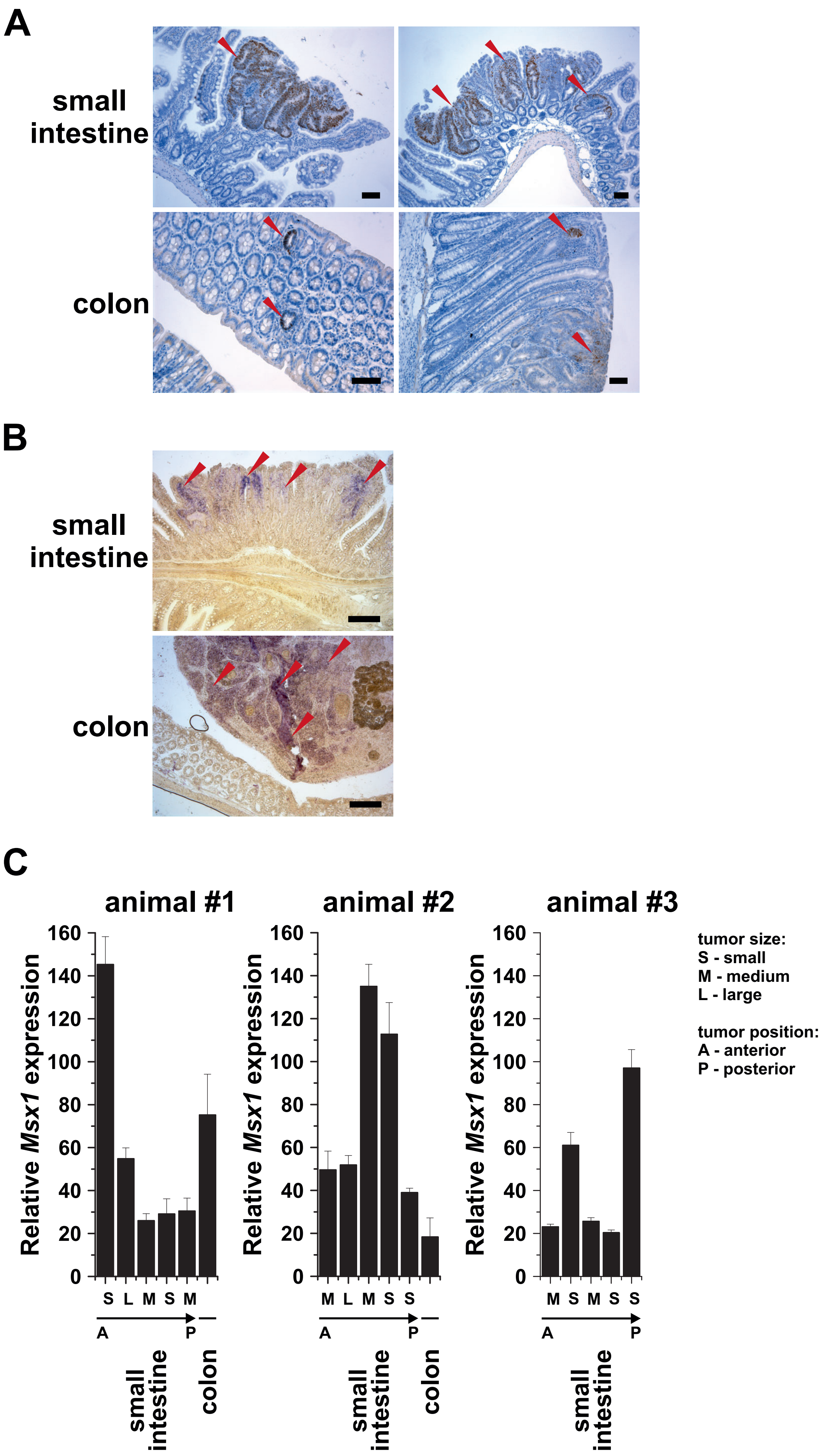

## Supplementary Figure S5

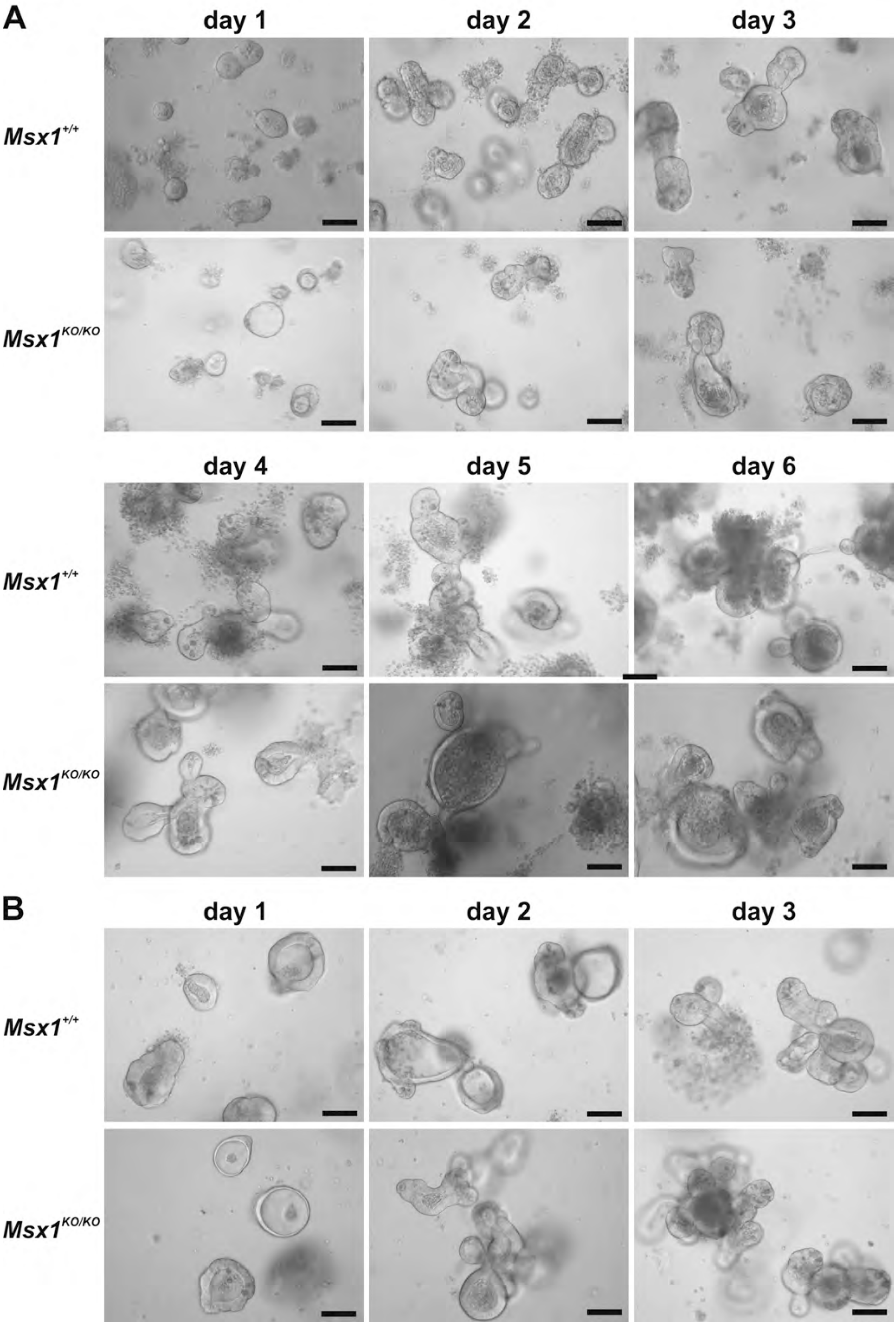

# Supplementary Figure S6

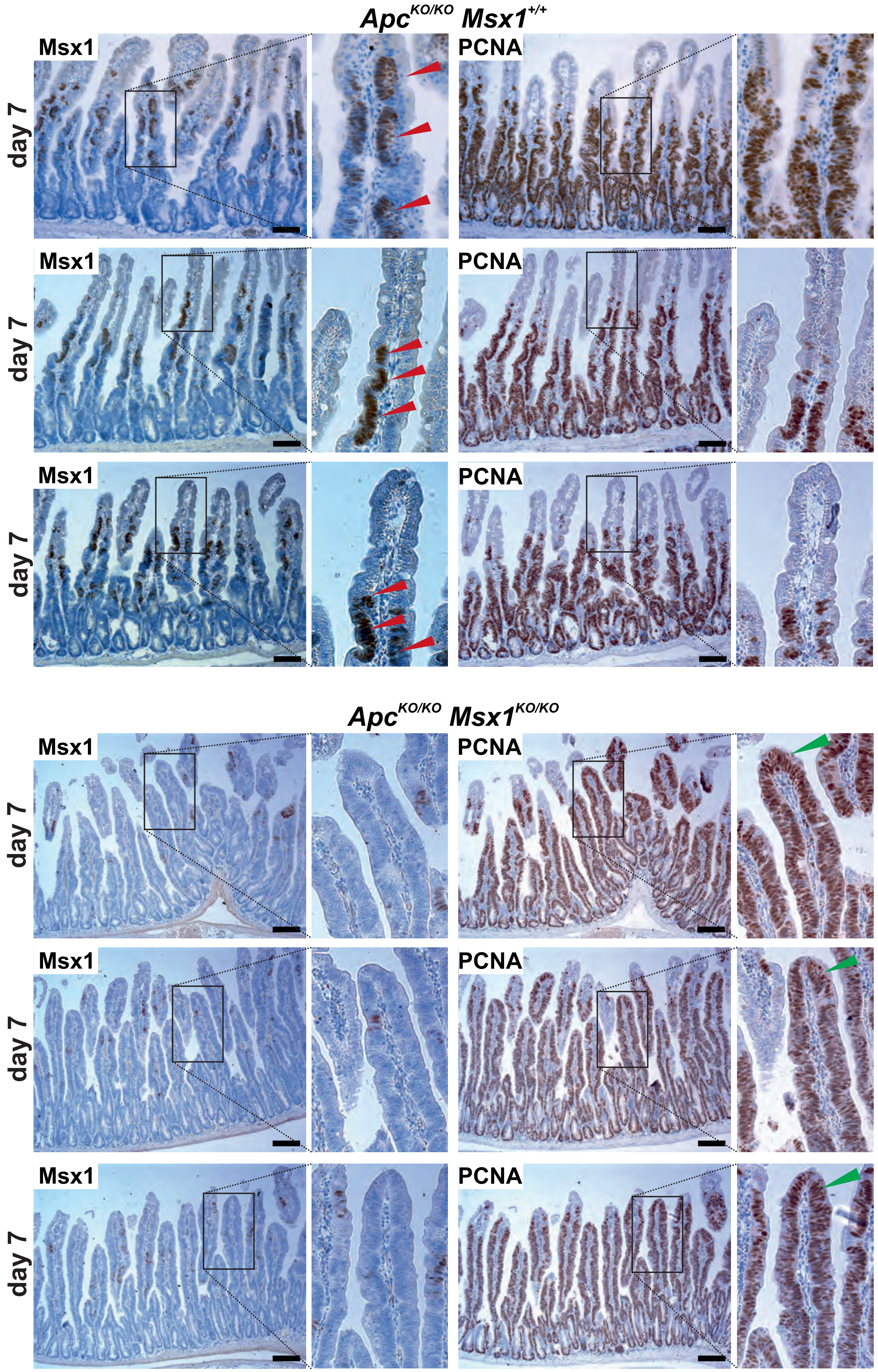

# Supplementary Figure S7

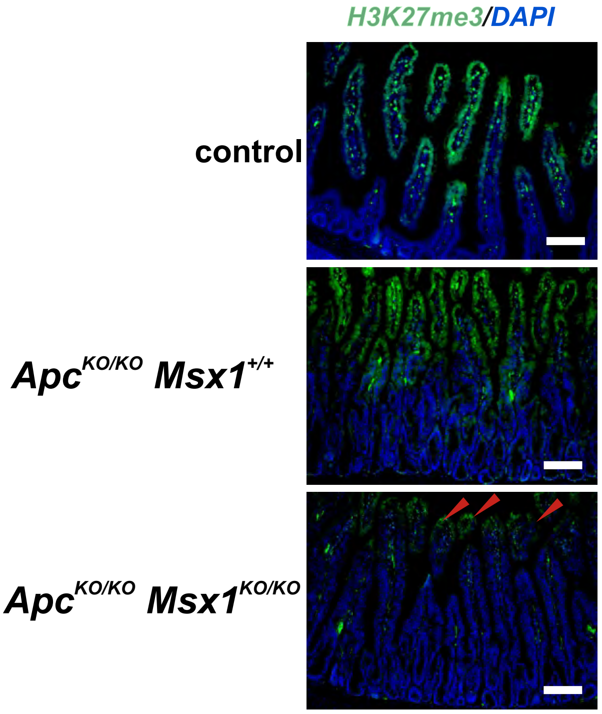

# Supplementary Figure S8

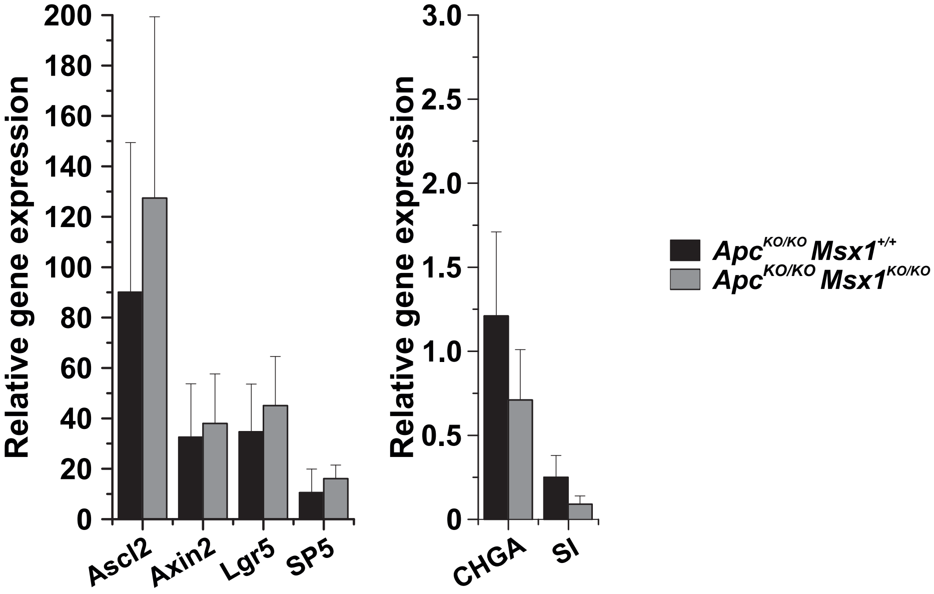

# Supplementary Figure S9

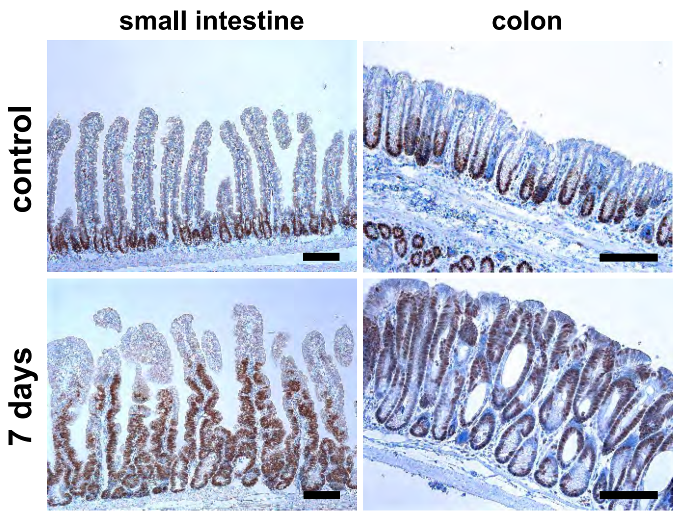

# Supplementary Figure S10

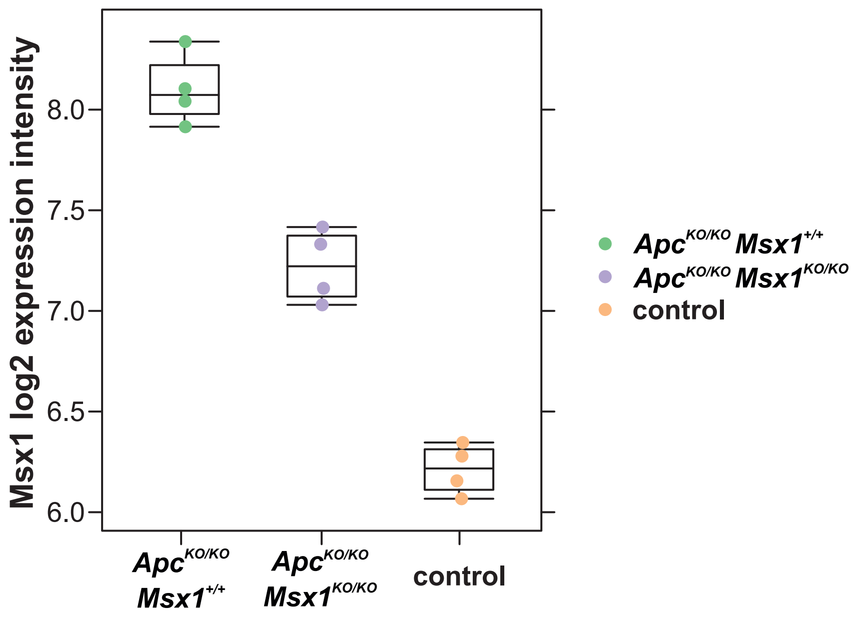


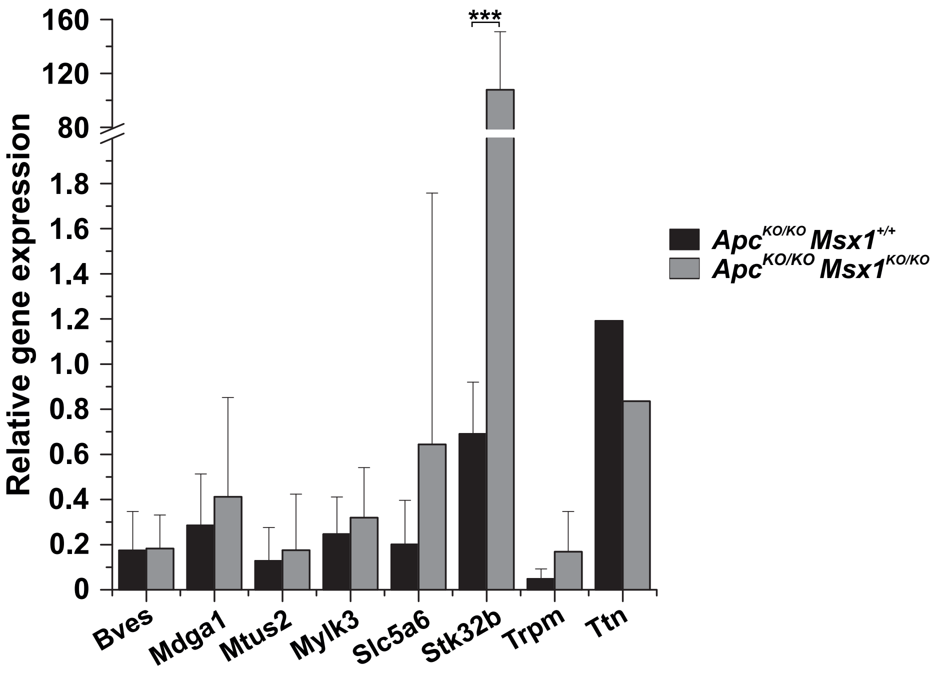

# Supplementary Figure S11

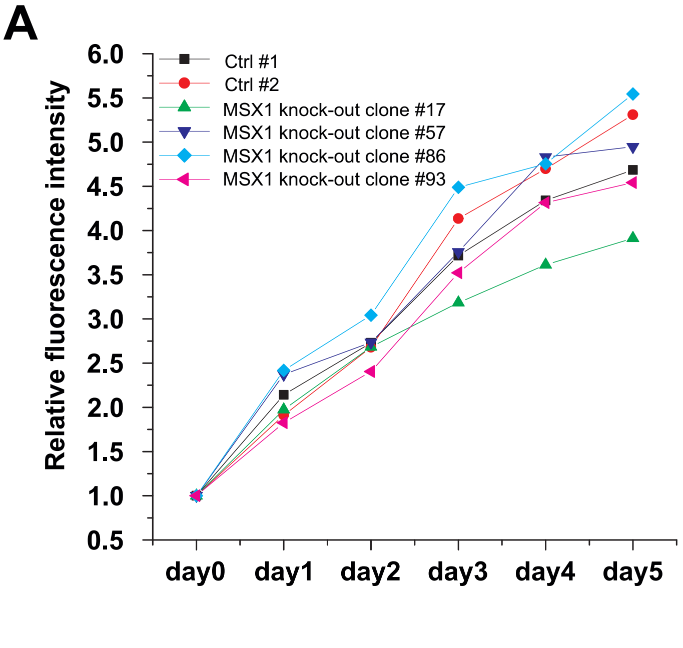

# Supplementary Figure S12

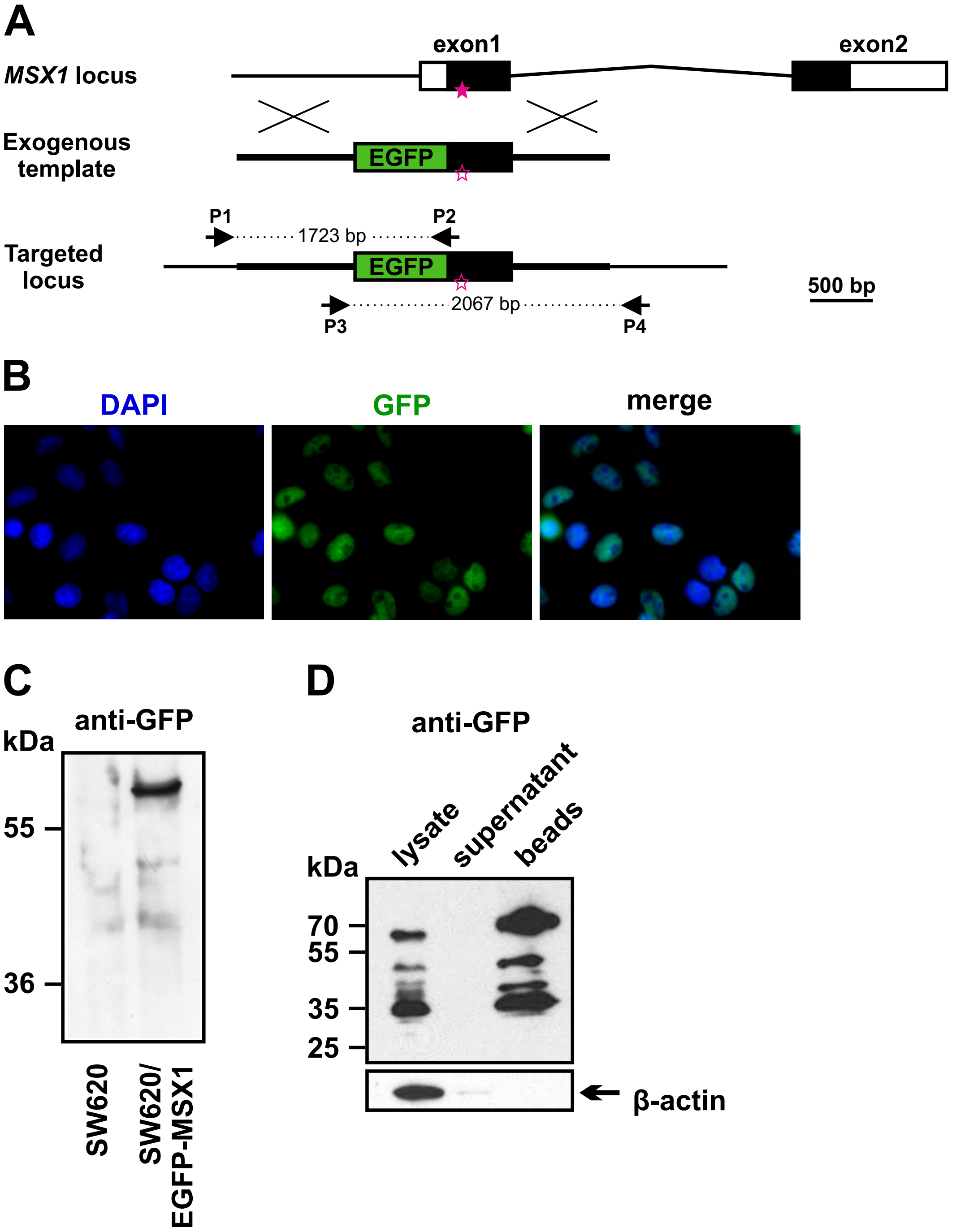

# Supplementary Figure S13

## A

*ASCL2* locus

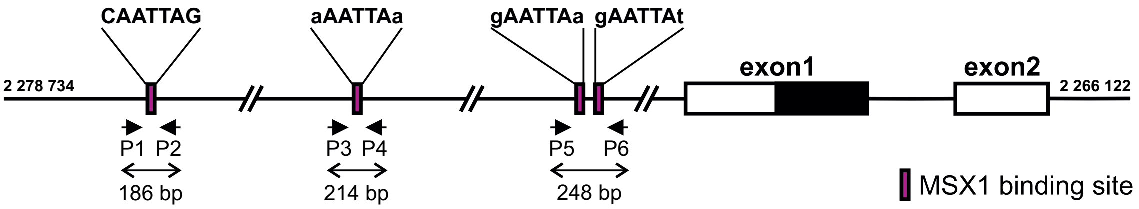


*SP5* promoter region

GCGGGTACCGCGAGGGTGCAGGGTGTGCAAGTAAATACAGGGGAGAGTTACACTCTTTGCCG
TCTCAAAGTCCGGCGCCTGGAAGCCGAAGGCAGATTTTTCCAGAGATAACAAAGACACTTTG
TCACTTTGGGCGCTGCGTCGGTGCAAATCTTTAATTGCAAGTGTGTGTGTGTGTGTGTGTGT
GTATGTTGGGGGGGGCGGGAAGAGTGTCTCGAGCAACCCAAGCGCGGGTCTCCAGGCGGCAA
GGCCCCCTTTGATCAGGAAAATCCAATTATTTGTGTATA**TACATTTCCCACATAGTGATTAC**
**TTCATTAATTGTCCCGCCTTTATCTCCTCCCTTCCCATCCCCCCTAATAATCAGTTCTTTTA**
**TCCAGACCAACAAACACACCATAGGAGCTTTGTGGATTCAAAGGATTTGCTTTCGCTTCTGA**
**AAGAGCCGCTATTCTTTGATGATTGGGTAGCGGCAAACTTCAAAGCCATAAATCTTCCCTCT**
**GACTGGCTGGCGGCCCAGCAAAGTCCTTATCAAATTCTTGGAGGTGATCCTGAAGCGCTCAG**
**ACCGCGCGCGGGGCGAGCGAGCGGGGCGCGGCGAGGGGCAAGGGCGGGGAGGGCCCCGGCGC**
**TCAGAGCAGGCGCCAGGGAGGCAGGCTGGGCGGCCCTTCGTCCTCGCCTTCGGGTGTCCATG**
**CCTCGGCGGCGGCGTCCCGCTCCGCAGCCAGGGGCCTGCAAGCCGTAGCCATGGCCGCGGTG**
**GCCGTCCTCCGGAACGACTCGCTGCAGGCCTTTCTCC**AGGTCAGGGCCGAGCCCGGAGGGGG

## B

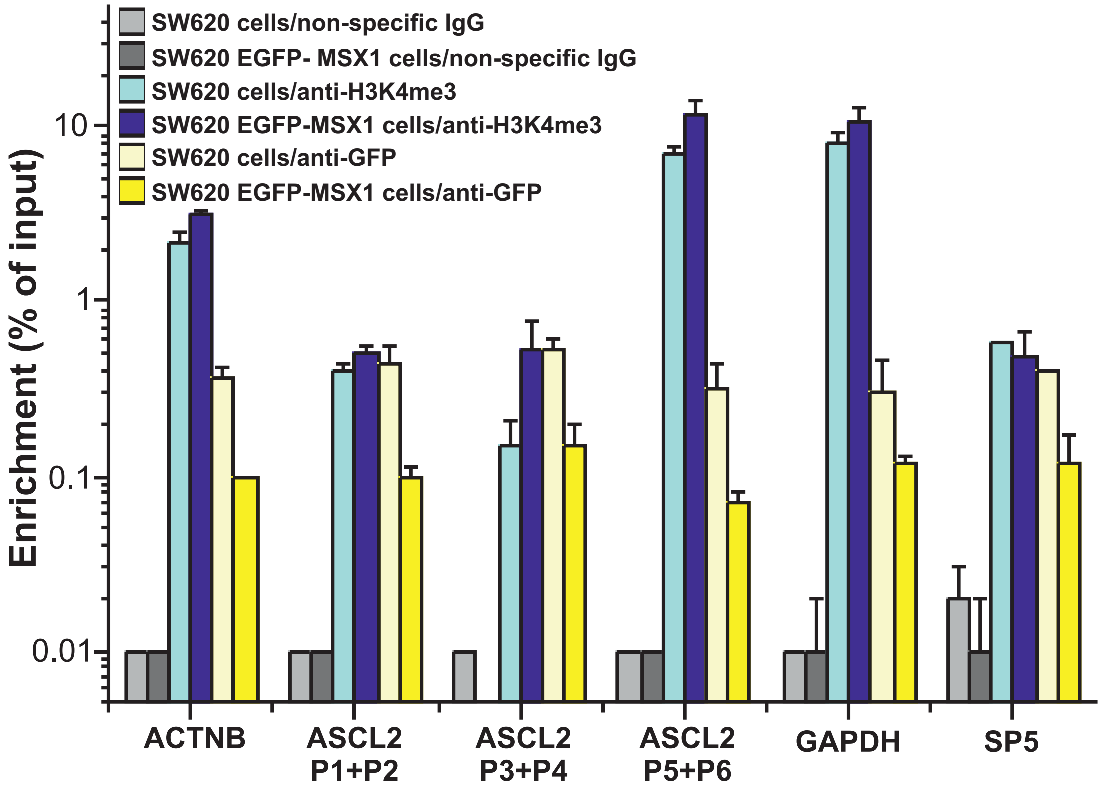

# Supplementary Figure S14

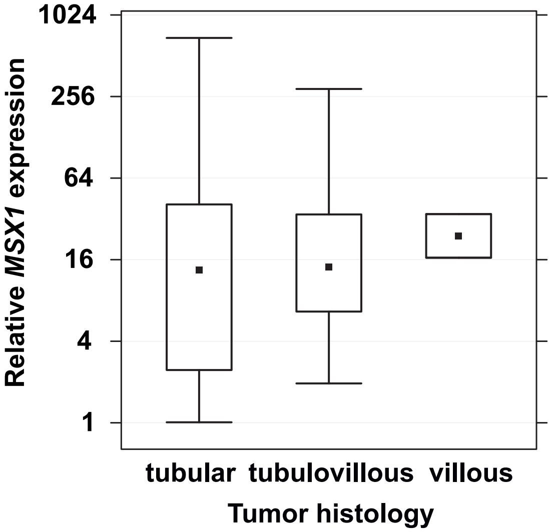

# Supplementary Figure S15

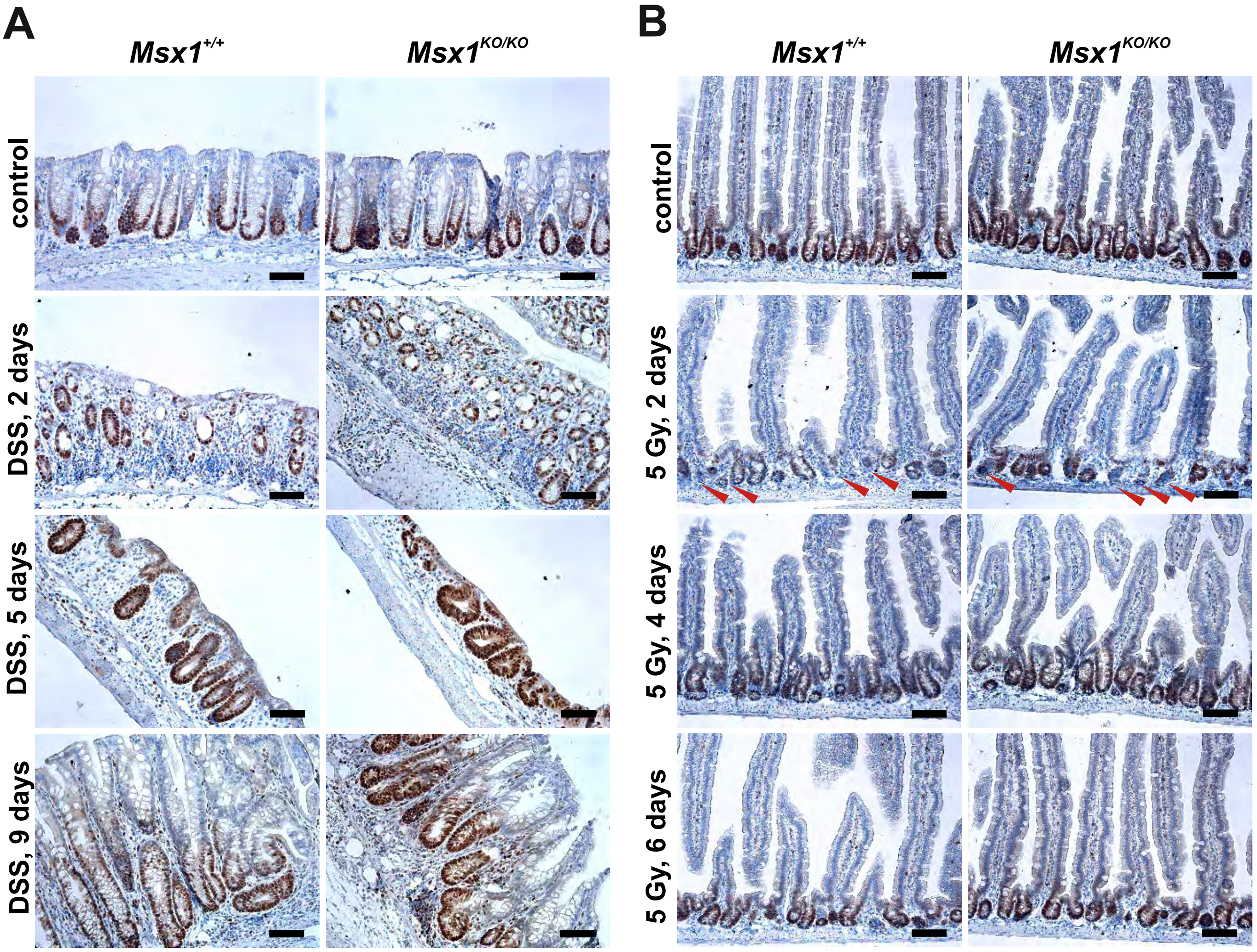

# Supplementary Figure S16

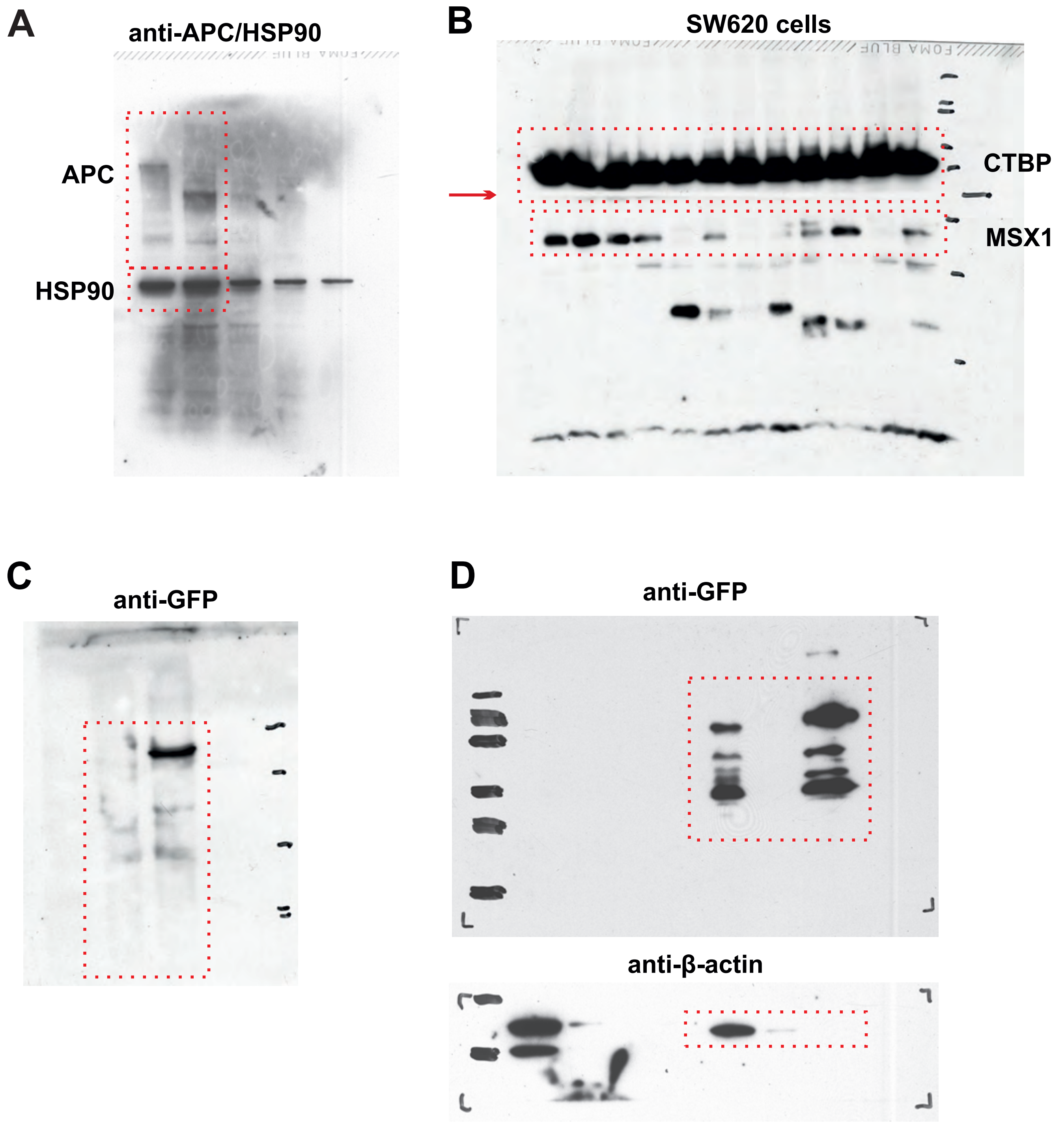

Supplement: Supplementary file 1 — Supplementary Materials_Methods_Figure and Table Legends_ Figures [file 41598_2018_38310_MOESM1_ESM.pdf]
