# Supplementary material for: Msx1 loss suppresses formation of the ectopic crypts developed in the Apc-deficient small intestinal epithelium
Source: Sci Rep. 2019 Feb 7;9:1629. doi: 10.1038/s41598-018-38310-y (PMC6367488; doi:10.1038/s41598-018-38310-y)
Supplement: Supplementary file 2 — Supplementary Tables [file 41598_2018_38310_MOESM2_ESM.pdf]

Supplementary Tables

Article: Msx1 loss suppresses formation of the ectopic crypts developed in the Apc-deficient small intestinal epithelium

Authors: Monika Horazna, Lucie Janeckova, Jiri Svec, Olga Babosova, Dusan Hrckulak, Martina Vojtechova, Katerina Galuskova, Eva Sloncova, Michal Kolar, Hynek Strnad, and Vladimir Korinek

Supplementary Table S1

Differentialy expressed genes (|logFC|≥ 1, q-value < 0.05 and) in the small intestinal epithelium 2 days upon Apc depletion compared to tissue with intact Apc

ENTREZ	SYMBOL	GENENAME	logFC	q-value
66214	1190002H23Rik	RIKEN cDNA 1190002H23 gene	2.09	0.0044
237038	Nox1	NADPH oxidase 1	1.76	0.0071
193740	Hspa1a	heat shock protein 1A	1.61	0.045
110454	Ly6a	lymphocyte antigen 6 complex, locus A	1.51	0.012
26897	ILMN_223756	Mus musculus acyl-CoA thioesterase 1 (Acot1), mRNA.	1.49	0.0055
29820	Tnfrsf19	tumor necrosis factor receptor superfamily, member 19	1.45	0.00033
74186	Ccdc3	coiled-coil domain containing 3	1.43	2.00E-04
11459	Acta1	actin, alpha 1, skeletal muscle	1.42	0.01
11839	Areg	amphiregulin	1.4	0.012
66141	Ifitm3	interferon induced transmembrane protein 3	1.3	0.0038
406217	ILMN_231589	Mus musculus brain expressed gene 4 (Bex4), mRNA.	1.28	7.00E-04
226419	Dyrk3	dual-specificity tyrosine-(Y)-phosphorylation regulated kinase 3	1.2	0.014
406217	Bex4	brain expressed gene 4	1.2	0.028
15985	Cd79b	CD79B antigen	1.18	0.031
72821	Scn2b	sodium channel, voltage-gated, type II, beta	1.17	0.0052
26897	Acot1	acyl-CoA thioesterase 1	1.17	0.0044
73710	Tubb2b	tubulin, beta 2B	1.15	0.0017
53605	ILMN_214137	Mus musculus nucleosome assembly protein 1-like 1 (Nap1l1), mRNA.	1.14	0.0013
244886	AI118078	expressed sequence AI118078	1.14	0.0043
19703	Renbp	renin binding protein	1.12	0.021
231327	Ppat	phosphoribosyl pyrophosphate amidotransferase	1.08	0.0019
14859	Gsta3	glutathione S-transferase, alpha 3	1.08	0.018
68026	2810417H13Rik	RIKEN cDNA 2810417H13 gene	1.06	0.018
71805	Nup93	nucleoporin 93	1.05	0.0038
320685	Dctd	dCMP deaminase	1.02	0.036
100047619	ILMN_219663	PREDICTED: Mus musculus similar to solute carrier family 7 (cationic amino acid transporter, y+ system), member 5 (LOC100047619), misc RNA.	1.01	0.011
26362	Axl	AXL receptor tyrosine kinase	1	0.046
77125	Il33	interleukin 33	-1	0.031
212070	Clrn3	clarin 3	-1	0.0096
11832	ILMN_210666	Mus musculus aquaporin 7 (Aqp7), mRNA.	-1	7.00E-04
74018	ILMN_211844	Mus musculus amyotrophic lateral sclerosis 2 (juvenile) homolog (human) (Als2), mRNA.	-1	0.0088
71670	Acy3	aspartoacylase (aminoacylase) 3	-1	0.0038
328365	Zmiz1	zinc finger, MIZ-type containing 1	-1.01	0.033
104086	Cyp27a1	cytochrome P450, family 27, subfamily a, polypeptide 1	-1.01	0.0032
209387	Trim30d	tripartite motif-containing 30D	-1.01	0.017
104086	Cyp27a1	cytochrome P450, family 27, subfamily a, polypeptide 1	-1.03	0.007
12351	Car4	carbonic anhydrase 4	-1.03	0.028
71733	Susd2	sushi domain containing 2	-1.04	0.045
15567	Slc6a4	solute carrier family 6 (neurotransmitter transporter, serotonin), member 4	-1.04	0.0052
68846	Rnf208	ring finger protein 208	-1.04	0.043
16548	Khk	ketohexokinase	-1.04	0.015
11803	Aplp1	amyloid beta (A4) precursor-like protein 1	-1.04	0.02
21929	Tnfaip3	tumor necrosis factor, alpha-induced protein 3	-1.05	0.029
71648	ILMN_216896	Mus musculus optineurin (Optn), mRNA.	-1.05	0.0075
69123	ILMN_210310	Mus musculus RIKEN cDNA 1810022C23 gene (1810022C23Rik), mRNA.	-1.06	0.0025
19017	Ppargc1a	peroxisome proliferative activated receptor, gamma, coactivator 1 alpha	-1.07	0.019
29813	Zfp385a	zinc finger protein 385A	-1.08	0.014
13026	Pcyt1a	phosphate cytidylyltransferase 1, choline, alpha isoform	-1.08	0.0074
54200	Sult2b1	sulfotransferase family, cytosolic, 2B, member 1	-1.09	0.004
215113	Slc43a2	solute carrier family 43, member 2	-1.09	0.0044
213019	Pdlim2	PDZ and LIM domain 2	-1.09	0.046
13120	Cyp4b1	cytochrome P450, family 4, subfamily b, polypeptide 1	-1.09	0.0062
17684	Cited2	Cbp/p300-interacting transactivator, with Glu/Asp-rich carboxy-terminal domain, 2	-1.1	0.003
67131	Acbd4	acyl-Coenzyme A binding domain containing 4	-1.1	0.014
20604	Sst	somatostatin	-1.11	0.042
104601	Mycbpap	MYCBP associated protein	-1.11	0.0038
100044177	ILMN_185055	PREDICTED: Mus musculus hypothetical protein LOC100044177 (LOC100044177), mRNA.	-1.11	0.0058
433470	AA467197	expressed sequence AA467197	-1.11	0.038
12182	ILMN_214474	Mus musculus bone marrow stromal cell antigen 1 (Bst1), mRNA.	-1.12	0.0038
54200	Sult2b1	sulfotransferase family, cytosolic, 2B, member 1	-1.13	0.0038
233836	Slc5a11	solute carrier family 5 (sodium/glucose cotransporter), member 11	-1.13	0.012
213522	Plekhg6	pleckstrin homology domain containing, family G (with RhoGef domain) member 6	-1.13	0.0077
12962	Crybb3	crystallin, beta B3	-1.13	0.003
54447	Asah2	N-acylsphingosine amidohydrolase 2	-1.13	0.043
11832	Aqp7	aquaporin 7	-1.13	0.026
67484	Eepd1	endonuclease/exonuclease/phosphatase family domain containing 1	-1.13	0.0038
545902	Ptprh	protein tyrosine phosphatase, receptor type, H	-1.15	0.006
213019	Pdlim2	PDZ and LIM domain 2	-1.15	0.017
56226	Espn	espin	-1.15	0.0088
13850	Ephx2	epoxide hydrolase 2, cytoplasmic	-1.15	0.039
258458	Olfr165	olfactory receptor 165	-1.16	0.0044
432516	Myo1a	myosin IA	-1.16	0.0017
20526	Slc2a2	solute carrier family 2 (facilitated glucose transporter), member 2	-1.17	0.0038
19660	Rbp2	retinol binding protein 2, cellular	-1.17	0.046
14963	ILMN_208890	Mus musculus histocompatibility 2, blastocyst (H2-Bl), mRNA.	-1.17	0.026
93694	Clec2d	C-type lectin domain family 2, member d	-1.17	0.0058
50934	Slc7a8	solute carrier family 7 (cationic amino acid transporter, y+ system), member 8	-1.18	0.0038
13370	Dio1	deiodinase, iodothyronine, type I	-1.18	0.0075
208677	Creb3l3	cAMP responsive element binding protein 3-like 3	-1.18	0.039
217166	Nr1d1	nuclear receptor subfamily 1, group D, member 1	-1.2	0.007
68396	Nat8	N-acetyltransferase 8 (GCN5-related, putative)	-1.2	0.028
20698	Sphk1	sphingosine kinase 1	-1.22	0.0052
11421	Ace	angiotensin I converting enzyme (peptidyl-dipeptidase A) 1	-1.22	0.013
54200	Sult2b1	sulfotransferase family, cytosolic, 2B, member 1	-1.23	0.00085
20537	Slc5a1	solute carrier family 5 (sodium/glucose cotransporter), member 1	-1.23	0.0044
11421	Ace	angiotensin I converting enzyme (peptidyl-dipeptidase A) 1	-1.26	0.0044
215113	Slc43a2	solute carrier family 43, member 2	-1.28	0.00048
19694	Reg3a	regenerating islet-derived 3 alpha	-1.29	0.049
67473	Slc47a1	solute carrier family 47, member 1	-1.32	0.039
67405	Nts	neurotensin	-1.33	0.01
232409	Clec2e	C-type lectin domain family 2, member e	-1.33	0.0044
20526	Slc2a2	solute carrier family 2 (facilitated glucose transporter), member 2	-1.34	0.0058
109731	Maob	monoamine oxidase B	-1.34	0.01

12013	Bach1	BTB and CNC homology 1	-1.34	0.031
228775	Trib3	tribbles homolog 3 (Drosophila)	-1.37	0.0019
13370	Dio1	deiodinase, iodothyronine, type I	-1.37	0.0042
56643	Slc15a1	solute carrier family 15 (oligopeptide transporter), member 1	-1.4	0.029
19692	Reg1	regenerating islet-derived 1	-1.41	0.0058
67082	1700011H14Rik	RIKEN cDNA 1700011H14 gene	-1.41	0.0058
70261	2010110P09Rik	RIKEN cDNA 2010110P09 gene	-1.45	0.0058
17380	ILMN_220122	Mus musculus membrane metallo endopeptidase (Mme), mRNA.	-1.5	0.012
18604	Pdk2	pyruvate dehydrogenase kinase, isoenzyme 2	-1.51	0.00019
230163	Aldob	aldolase B, fructose-bisphosphate	-1.52	0.022
64452	Slc5a4a	solute carrier family 5, member 4a	-1.54	0.0016
17380	Mme	membrane metallo endopeptidase	-1.62	0.0055
68947	Chst8	carbohydrate (N-acetylgalactosamine 4-0) sulfotransferase 8	-1.66	0.0033
54150	Rdh7	retinol dehydrogenase 7	-1.68	2.00E-04
71584	Gdpd2	glycerophosphodiester phosphodiesterase domain containing 2	-1.71	2.10E-06
13419	Dnase1	deoxyribonuclease I	-1.75	0.028
20526	Slc2a2	solute carrier family 2 (facilitated glucose transporter), member 2	-1.83	0.013
64454	Slc5a4b	solute carrier family 5 (neutral amino acid transporters, system A), member 4b	-1.89	3.50E-05
54150	Rdh7	retinol dehydrogenase 7	-1.94	2.00E-04
545156	Kalrn	kalirin, RhoGEF kinase	-1.94	0.00084
434203	ILMN_244381	Mus musculus solute carrier family 28 (sodium-coupled nucleoside transporter), member 1 (Slc28a1), mRNA.	-2.14	0.00046

Supplementary Table S1

Differentialy expressed genes (|logFC| ≥1, q-value < 0.05) in the small intestinal epithelium 4 days upon Apc depletion compared to tissue with intact Apc

ENTREZ	SYMBOL	GENENAME	logFC	q-value
213948	Atg9b	ATG9 autophagy related 9 homolog B (S. cerevisiae)	4.12	9.00E-08
74186	Ccdc3	coiled-coil domain containing 3	3.91	3.80E-11
66214	1190002H23Rik	RIKEN cDNA 1190002H23 gene	3.69	4.90E-07
17701	Msx1	homeobox, msh-like 1	3.53	4.10E-11
110454	Ly6a	lymphocyte antigen 6 complex, locus A	3.52	1.90E-07
16010	Igfbp4	insulin-like growth factor binding protein 4	3.48	4.80E-09
406217	ILMN_231589	Mus musculus brain expressed gene 4 (Bex4), mRNA.	3.4	4.30E-10
66141	Ifitm3	interferon induced transmembrane protein 3	3.1	1.40E-08
18612	Etv4	ets variant gene 4 (E1A enhancer binding protein, E1AF)	3.1	8.90E-07
16010	Igfbp4	insulin-like growth factor binding protein 4	3.09	4.40E-07
237038	Nox1	NADPH oxidase 1	3.06	1.80E-06
26897	ILMN_223756	Mus musculus acyl-CoA thioesterase 1 (Acot1), mRNA.	3.06	1.50E-07
29820	Tnfrsf19	tumor necrosis factor receptor superfamily, member 19	2.95	2.10E-09
406217	Bex4	brain expressed gene 4	2.81	1.20E-06
73710	Tubb2b	tubulin, beta 2B	2.66	6.30E-09
18383	Tnfrsf11b	tumor necrosis factor receptor superfamily, member 11b (osteoprotegerin)	2.63	6.90E-08
26897	Acot1	acyl-CoA thioesterase 1	2.61	4.30E-08
94179	Krt23	keratin 23	2.53	3.50E-07
14859	Gsta3	glutathione S-transferase, alpha 3	2.51	4.90E-07
103551	E130012A19Rik	RIKEN cDNA E130012A19 gene	2.5	1.00E-06
12505	Cd44	CD44 antigen	2.49	4.10E-08
27279	Tnfrsf12a	tumor necrosis factor receptor superfamily, member 12a	2.47	2.50E-05
16918	Mycl1	v-myc myelocytomatosis viral oncogene homolog 1, lung carcinoma derived (avian)	2.39	3.10E-07
13401	Dmwd	dystrophia myotonica-containing WD repeat motif	2.37	5.00E-09
55963	Slc1a4	solute carrier family 1 (glutamate/neutral amino acid transporter), member 4	2.33	3.90E-07
100102	Pcsk9	proprotein convertase subtilisin/kexin type 9	2.33	2.80E-07
56016	Hebp2	heme binding protein 2	2.33	1.10E-07
18432	Mybbp1a	MYB binding protein (P160) 1a	2.31	2.60E-07
320027	Fstl4	follistatin-like 4	2.31	9.60E-08
11459	Acta1	actin, alpha 1, skeletal muscle	2.31	7.30E-06
19703	Renbp	renin binding protein	2.3	2.70E-06
17357	Marcksl1	MARCKS-like 1	2.26	4.20E-07
100047619	ILMN_219663	PREDICTED: Mus musculus similar to solute carrier family 7 (cationic amino acid transporter, y+ system), member 5 (LOC100047619), misc RNA.	2.23	2.90E-07
107581	Col16a1	collagen, type XVI, alpha 1	2.23	8.40E-06
226419	Dyrk3	dual-specificity tyrosine-(Y)-phosphorylation regulated kinase 3	2.22	3.20E-06
19385	Ranbp1	RAN binding protein 1	2.17	3.70E-09
19277	Ptpro	protein tyrosine phosphatase, receptor type, O	2.16	1.40E-06
15360	Hmgcs2	3-hydroxy-3-methylglutaryl-Coenzyme A synthase 2	2.16	0.0056
12709	ILMN_193661	Mus musculus creatine kinase, brain (Ckb), mRNA.	2.16	4.10E-06
20810	Srm	spermidine synthase	2.15	1.10E-06
72821	Scn2b	sodium channel, voltage-gated, type II, beta	2.15	4.40E-07
11839	Areg	amphiregulin	2.15	1.80E-05
215690	Nav1	neuron navigator 1	2.14	4.20E-07
13401	Dmwd	dystrophia myotonica-containing WD repeat motif	2.14	6.90E-09
21677	Tead2	TEA domain family member 2	2.09	1.70E-07
53605	ILMN_214137	Mus musculus nucleosome assembly protein 1-like 1 (Nap1l1), mRNA.	2.09	4.00E-08
14862	Gstm1	glutathione S-transferase, mu 1	2.09	3.90E-09
15469	Prmt1	protein arginine N-methyltransferase 1	2.08	3.40E-08
231327	Ppat	phosphoribosyl pyrophosphate amidotransferase	2.06	5.00E-08
244886	AI118078	expressed sequence AI118078	2.06	3.10E-07
15220	Foxq1	forkhead box Q1	2.05	1.80E-05
11925	Neurog3	neurogenin 3	2.04	2.40E-06
14570	Arhgdig	Rho GDP dissociation inhibitor (GDI) gamma	2.03	6.90E-07
72462	Rrp1b	ribosomal RNA processing 1 homolog B (S. cerevisiae)	2.01	2.00E-07
353156	Egfl7	EGF-like domain 7	2	9.80E-08
100046120	ILMN_220805	PREDICTED: Mus musculus similar to clusterin (LOC100046120), mRNA.	1.99	0.00013
215690	Nav1	neuron navigator 1	1.98	1.20E-06
12505	Cd44	CD44 antigen	1.97	4.90E-07
100044103	ILMN_210607	PREDICTED: Mus musculus similar to mKIAA1645 protein (LOC100044103), mRNA.	1.96	1.00E-06
19752	Rnase1	ribonuclease, RNase A family, 1 (pancreatic)	1.95	6.20E-05
66350	Pla2g12a	phospholipase A2, group XIIA	1.95	2.60E-07
102657	Cd276	CD276 antigen	1.95	3.20E-07
223227	ILMN_220261	Mus musculus SRY-box containing gene 21 (Sox21), mRNA.	1.94	3.20E-09
19280	Ptprs	protein tyrosine phosphatase, receptor type, S	1.94	1.90E-08
320685	Dctd	dCMP deaminase	1.94	2.20E-05
69524	Esam	endothelial cell-specific adhesion molecule	1.93	1.30E-07
20652	Soat1	sterol O-acyltransferase 1	1.92	1.30E-05
109857	Cbr3	carbonyl reductase 3	1.9	0.00038
68147	Gar1	GAR1 ribonucleoprotein homolog (yeast)	1.89	1.80E-07
15114	Hap1	huntingtin-associated protein 1	1.88	8.80E-07
107221	Gpr120	G protein-coupled receptor 120	1.88	9.70E-08
70082	Lysmd2	LysM, putative peptidoglycan-binding, domain containing 2	1.87	8.10E-06
53356	Eif3g	eukaryotic translation initiation factor 3, subunit G	1.86	7.00E-06
98170	Tmem132a	transmembrane protein 132A	1.85	3.40E-08
50492	Thop1	thimet oligopeptidase 1	1.84	4.90E-07
20810	Srm	spermidine synthase	1.84	1.40E-06
26564	Ror2	receptor tyrosine kinase-like orphan receptor 2	1.84	3.20E-06
77583	Notum	notum pectinacetylesterase homolog (Drosophila)	1.84	1.30E-08
110006	ILMN_221841	Mus musculus glucuronidase, beta (Gusb), mRNA.	1.84	2.90E-07
27280	Phlda3	pleckstrin homology-like domain, family A, member 3	1.83	7.00E-04
107272	Psat1	phosphoserine aminotransferase 1	1.82	8.70E-06
57257	Vav3	vav 3 oncogene	1.81	1.40E-08
21664	Phlda1	pleckstrin homology-like domain, family A, member 1	1.81	0.00048
434632	BC085271	cDNA sequence BC085271	1.81	5.00E-06
53356	Eif3g	eukaryotic translation initiation factor 3, subunit G	1.8	7.30E-07
12162	Bmp7	bone morphogenetic protein 7	1.8	1.80E-06
268396	Sh3pxd2b	SH3 and PX domains 2B	1.79	3.10E-07
240334	Pcyox1l	prenylcysteine oxidase 1 like	1.79	2.50E-06
22021	Tpst1	protein-tyrosine sulfotransferase 1	1.78	2.70E-07
18710	Pik3r3	phosphatidylinositol 3 kinase, regulatory subunit, polypeptide 3 (p55)	1.78	2.00E-07
19130	Prox1	prospero-related homeobox 1	1.77	2.00E-08
72500	ILMN_225633	Mus musculus immediate early response 5-like (Ier5l), mRNA.	1.77	1.90E-08
76267	Fads1	fatty acid desaturase 1	1.77	2.70E-07
93721	Cpn1	carboxypeptidase N, polypeptide 1	1.77	1.20E-05
66953	Cdca7	cell division cycle associated 7	1.77	1.20E-06
11898	Ass1	argininosuccinate synthetase 1	1.77	1.20E-05
116972	Fam57a	family with sequence similarity 57, member A	1.77	1.50E-07
102614	Rpp25	ribonuclease P 25 subunit (human)	1.76	2.70E-07
319520	Dusp4	dual specificity phosphatase 4	1.76	4.30E-07
270152	Amica1	adhesion molecule, interacts with CXADR antigen 1	1.76	4.40E-06
20682	Sox9	SRY-box containing gene 9	1.75	1.60E-05
109820	Pgc	progastricsin (pepsinogen C)	1.75	2.70E-06
68915	Vars2	valyl-tRNA synthetase 2, mitochondrial (putative)	1.74	7.80E-08
20810	Srm	spermidine synthase	1.74	2.90E-05
100047856	ILMN_208813	PREDICTED: Mus musculus similar to calponin 3, acidic (LOC100047856), mRNA.	1.74	2.00E-06
116972	Fam57a	family with sequence similarity 57, member A	1.74	7.50E-09
52530	Nhp2	NHP2 ribonucleoprotein homolog (yeast)	1.73	9.50E-07
72640	Mex3a	mex3 homolog A (C. elegans)	1.73	4.30E-08
19183	Psmc3ip	proteasome (prosome, macropain) 26S subunit, ATPase 3, interacting protein	1.72	1.50E-05
328949	ILMN_247906	Mus musculus mutated in colorectal cancers (Mcc), mRNA.	1.71	1.20E-06
242785	Klhl21	kelch-like 21 (Drosophila)	1.71	1.20E-05
12505	Cd44	CD44 antigen	1.71	7.80E-06

229474	Fhdc1	FH2 domain containing 1	1.71	2.50E-06
93840	Vangl2	vang-like 2 (van gogh, Drosophila)	1.7	2.00E-06
56505	Ruvbl1	RuvB-like protein 1	1.7	4.30E-08
19280	Ptprs	protein tyrosine phosphatase, receptor type, S	1.7	4.90E-10
69902	Mrto4	MRT4, mRNA turnover 4, homolog (S. cerevisiae)	1.69	3.60E-07
17083	Tmed1	transmembrane emp24 domain containing 1	1.69	3.80E-07
15361	Hmga1	high mobility group AT-hook 1	1.69	2.80E-05
30877	Gnl3	guanine nucleotide binding protein-like 3 (nucleolar)	1.69	6.90E-06
22154	Tubb5	tubulin, beta 5	1.68	2.70E-07
108037	Shmt2	serine hydroxymethyltransferase 2 (mitochondrial)	1.68	2.30E-07
71805	Nup93	nucleoporin 93	1.68	8.50E-07
71242	Spata24	spermatogenesis associated 24	1.68	3.20E-06
209354	Eif2b1	eukaryotic translation initiation factor 2B, subunit 1 (alpha)	1.67	2.10E-08
110749	ILMN_215876	Mus musculus chromatin assembly factor 1, subunit B (p60) (Chaf1b), mRNA.	1.67	2.80E-07
94275	ILMN_215570	Mus musculus melanoma antigen, family D, 1 (Maged1), mRNA.	1.66	4.60E-07
51797	Ctps	cytidine 5'-triphosphate synthase	1.66	2.50E-06
27373	Csnk1e	casein kinase 1, epsilon	1.65	1.50E-08
100046741	ILMN_190874	PREDICTED: Mus musculus similar to red-1 (LOC100046741), mRNA.	1.64	1.60E-07
110006	Gusb	glucuronidase, beta	1.64	2.30E-06
13731	Emp2	epithelial membrane protein 2	1.64	4.40E-06
63959	Slc29a1	solute carrier family 29 (nucleoside transporters), member 1	1.63	1.20E-05
19227	Pthlh	parathyroid hormone-like peptide	1.63	3.20E-08
18391	Sigmar1	sigma non-opioid intracellular receptor 1	1.63	2.60E-07
272359	Irf2bp1	interferon regulatory factor 2 binding protein 1	1.63	3.10E-07
67951	Tubb6	tubulin, beta 6	1.62	8.70E-06
20019	Polr1a	polymerase (RNA) I polypeptide A	1.62	9.00E-08
22113	Phlda2	pleckstrin homology-like domain, family A, member 2	1.62	0.00012
57028	Pdxp	pyridoxal (pyridoxine, vitamin B6) phosphatase	1.62	1.10E-07
55927	Hes6	hairy and enhancer of split 6 (Drosophila)	1.62	1.00E-07
11792	Apex1	apurinic/apyrimidinic endonuclease 1	1.62	8.60E-05
67824	Nmral1	NmrA-like family domain containing 1	1.61	1.80E-07
16668	Krt18	keratin 18	1.61	1.80E-06
66902	Mtap	methylthioadenosine phosphorylase	1.6	4.50E-07
55927	Hes6	hairy and enhancer of split 6 (Drosophila)	1.6	5.70E-07
116701	ILMN_211983	Mus musculus fibroblast growth factor receptor-like 1 (Fgfrl1), mRNA.	1.6	7.30E-07
227358	Fam132b	family with sequence similarity 132, member B	1.6	8.90E-07
14160	Lgr5	leucine rich repeat containing G protein coupled receptor 5	1.59	1.90E-06
72017	Cyb5r1	cytochrome b5 reductase 1	1.59	0.00015
17173	Ascl2	achaete-scute complex homolog 2 (Drosophila)	1.59	0.00012
27966	Rrp9	RRP9, small subunit (SSU) processome component, homolog (yeast)	1.58	4.40E-06
74747	Ddit4	DNA-damage-inducible transcript 4	1.58	1.50E-05
12124	Bik	BCL2-interacting killer	1.58	2.20E-06
66976	ILMN_212730	Mus musculus RIKEN cDNA 2410002F23 gene (2410002F23Rik), mRNA.	1.58	3.90E-06
30927	Snai3	snail homolog 3 (Drosophila)	1.57	4.00E-08
20509	Slc19a1	solute carrier family 19 (sodium/hydrogen exchanger), member 1	1.57	6.30E-07
108014	Srsf9	serine/arginine-rich splicing factor 9	1.57	3.20E-07
17357	Marcksl1	MARCKS-like 1	1.57	1.30E-06
51797	Ctps	cytidine 5'-triphosphate synthase	1.57	2.50E-07
76459	Car12	carbonic anyhydrase 12	1.57	1.00E-05
12006	Axin2	axin2	1.57	6.60E-05
73710	2410129E14Rik	Mus musculus tubulin, beta 2b (Tubb2b), mRNA.	1.56	3.00E-05
20361	Sema7a	sema domain, immunoglobulin domain (Ig), and GPI membrane anchor, (semaphorin) 7A	1.56	4.80E-05
216456	Gls2	glutaminase 2 (liver, mitochondrial)	1.56	4.20E-06
13639	Efna4	ephrin A4	1.56	1.60E-05
104001	Rtn1	reticulon 1	1.55	0.00075
100608	ILMN_209940	Mus musculus nucleolar complex associated 4 homolog (S. cerevisiae) (Noc4l), mRNA.	1.55	4.10E-06
16206	Lrig1	leucine-rich repeats and immunoglobulin-like domains 1	1.55	3.90E-08
71242	Spata24	spermatogenesis associated 24	1.55	2.70E-07
13384	Mpp3	membrane protein, palmitoylated 3 (MAGUK p55 subfamily member 3)	1.54	5.00E-06
100019	Mdn1	midasin homolog (yeast)	1.54	1.10E-05
72393	Faim2	Fas apoptotic inhibitory molecule 2	1.54	3.60E-06
64406	Sp5	trans-acting transcription factor 5	1.53	1.20E-06
81840	Sorcs2	sortilin-related VPS10 domain containing receptor 2	1.53	1.40E-06
66870	Serbp1	serpine1 mRNA binding protein 1	1.53	8.80E-07
17393	Mmp7	matrix metallopeptidase 7	1.53	7.00E-06
66617	Mettl11a	methyltransferase like 11A	1.53	1.10E-05
216456	Gls2	glutaminase 2 (liver, mitochondrial)	1.53	4.20E-07
13436	Dnmt3b	DNA methyltransferase 3B	1.53	1.30E-07
11792	Apex1	apurinic/apyrimidinic endonuclease 1	1.52	2.20E-06
21813	Tgfbr2	transforming growth factor, beta receptor II	1.52	6.50E-06
17216	Mcm2	minichromosome maintenance deficient 2 mitotin (S. cerevisiae)	1.52	1.20E-07
22321	Vars	valyl-tRNA synthetase	1.51	9.70E-08
21844	Tiam1	T-cell lymphoma invasion and metastasis 1	1.51	6.00E-07
70556	Slc25a33	solute carrier family 25, member 33	1.51	4.10E-07
59028	Rcl1	RNA terminal phosphate cyclase-like 1	1.51	1.20E-05
29871	Scmh1	sex comb on midleg homolog 1	1.5	1.30E-06
67223	Rrp15	ribosomal RNA processing 15 homolog (S. cerevisiae)	1.5	4.90E-06
16907	Lmnb2	lamin B2	1.5	6.40E-05
57773	Wdr4	WD repeat domain 4	1.49	1.80E-06
18140	Uhrf1	ubiquitin-like, containing PHD and RING finger domains, 1	1.49	9.20E-07
21815	Tgif1	TGFB-induced factor homeobox 1	1.49	8.70E-06
53605	ILMN_214137	Mus musculus nucleosome assembly protein 1-like 1 (Nap1l1), mRNA.	1.49	2.10E-06
17218	Mcm5	minichromosome maintenance deficient 5, cell division cycle 46 (S. cerevisiae)	1.49	2.10E-07
15312	Hmgn1	high mobility group nucleosomal binding domain 1	1.49	5.70E-07
14450	Gart	phosphoribosylglycinamide formyltransferase	1.49	8.80E-06
13433	Dnmt1	DNA methyltransferase (cytosine-5) 1	1.49	6.00E-06
98170	Tmem132a	transmembrane protein 132A	1.48	1.90E-05
20133	ILMN_231868	Mus musculus ribonucleotide reductase M1 (Rrm1), mRNA.	1.48	7.00E-06
18784	Pla2g5	phospholipase A2, group V	1.48	0.00091
17319	Mif	macrophage migration inhibitory factor	1.48	3.10E-08
14187	ILMN_218920	Mus musculus aldo-keto reductase family 1, member B8 (Akr1b8), mRNA.	1.48	0.002
11564	Adsl	adenylosuccinate lyase	1.48	2.00E-06
215193	Diexf	digestive organ expansion factor homolog (zebrafish)	1.48	8.90E-07
328162	Trmt61a	tRNA methyltransferase 61 homolog A (S. cerevisiae)	1.48	2.50E-05
71242	Spata24	spermatogenesis associated 24	1.48	2.30E-07
103733	ILMN_184611	Mus musculus tubulin, gamma 1 (Tubg1), mRNA.	1.47	6.90E-05
654467	ILMN_234090	Mus musculus heterogeneous nuclear ribonucleoprotein A1 pseudogene (LOC654467) on chromosome 9.	1.47	3.40E-05
66637	Tsen15	tRNA splicing endonuclease 15 homolog (S. cerevisiae)	1.47	3.20E-07
57785	Rangrf	RAN guanine nucleotide release factor	1.46	4.70E-06
67177	Cdt1	chromatin licensing and DNA replication factor 1	1.46	2.80E-06
12469	Cct8	chaperonin containing Tcp1, subunit 8 (theta)	1.46	3.20E-06
27407	Abcf2	ATP-binding cassette, sub-family F (GCN20), member 2	1.46	3.10E-07
71981	Tdrd12	tudor domain containing 12	1.45	1.70E-05
17304	Mfge8	milk fat globule-EGF factor 8 protein	1.45	5.80E-05
101612	Grwd1	glutamate-rich WD repeat containing 1	1.45	2.00E-06
110956	D17H6S56E-5	DNA segment, Chr 17, human D6S56E 5	1.45	1.60E-05
66523	2810004N23Rik	RIKEN cDNA 2810004N23 gene	1.45	4.40E-07
17912	Myo1b	myosin IB	1.44	5.40E-05
17215	ILMN_213080	Mus musculus minichromosome maintenance deficient 3 (S. cerevisiae) (Mcm3), mRNA.	1.44	6.90E-08
55927	Hes6	hairy and enhancer of split 6 (Drosophila)	1.44	2.70E-06
80914	Uck2	uridine-cytidine kinase 2	1.43	2.80E-06
236539	ILMN_226456	Mus musculus 3-phosphoglycerate dehydrogenase (Phgdh), mRNA.	1.43	1.00E-04
75273	Pelp1	proline, glutamic acid and leucine rich protein 1	1.43	1.60E-05
110310	Krt7	keratin 7	1.43	8.30E-06
217995	ILMN_214317	Mus musculus HEAT repeat containing 1 (Heatr1), mRNA.	1.43	1.00E-06
170942	ILMN_221931	Mus musculus erythroid differentiation regulator 1 (Erdr1), mRNA.	1.43	0.0033
12505	Cd44	CD44 antigen	1.43	9.40E-05

209588	Sectm1a	secreted and transmembrane 1A	1.43	4.20E-07
66241	Tmem9	transmembrane protein 9	1.42	1.50E-06
78294	Rps27a	ribosomal protein S27A	1.42	4.60E-06
13650	Rhbdf1	rhomboid family 1 (Drosophila)	1.42	3.80E-06
12070	Ngfrap1	nerve growth factor receptor (TNFRSF16) associated protein 1	1.42	2.60E-07
13877	ILMN_212781	Mus musculus enhancer of rudimentary homolog (Drosophila) (Erh), mRNA.	1.42	5.10E-07
75430	ILMN_184438	Mus musculus RIKEN cDNA 3200002M19 gene (3200002M19Rik), mRNA.	1.42	0.00018
72061	2010111I01Rik	RIKEN cDNA 2010111I01 gene	1.42	2.30E-07
68948	1500011H22Rik	RIKEN cDNA 1500011H22 gene	1.42	3.40E-06
22247	Umps	uridine monophosphate synthetase	1.41	3.00E-06
56390	Sssca1	Sjogren's syndrome/scleroderma autoantigen 1 homolog (human)	1.41	1.10E-05
18391	Sigmar1	sigma non-opioid intracellular receptor 1	1.41	2.20E-06
17427	Mns1	meiosis-specific nuclear structural protein 1	1.41	8.20E-07
17217	Mcm4	minichromosome maintenance deficient 4 homolog (S. cerevisiae)	1.41	1.50E-06
100042777	ILMN_212781	PREDICTED: Mus musculus similar to human protein homologous to DROER protein (LOC100042777), mRNA.	1.41	1.30E-07
13844	Ephb2	Eph receptor B2	1.41	8.90E-07
69534	Avpi1	arginine vasopressin-induced 1	1.41	5.90E-05
107503	Atf5	activating transcription factor 5	1.41	6.30E-05
27407	Abcf2	ATP-binding cassette, sub-family F (GCN20), member 2	1.41	9.30E-06
27081	Zfp275	zinc finger protein 275	1.4	1.30E-05
22321	Vars	valyl-tRNA synthetase	1.4	5.70E-06
70356	St13	suppression of tumorigenicity 13	1.4	0.00054
20595	Smn1	survival motor neuron 1	1.4	1.00E-06
20563	Slit2	slit homolog 2 (Drosophila)	1.4	4.60E-06
27401	Skp2	S-phase kinase-associated protein 2 (p45)	1.4	5.60E-06
435684	ILMN_245068	Mus musculus Src homology 2 domain containing F (Shf), mRNA.	1.4	0.00017
27374	ILMN_257867	Mus musculus protein arginine N-methyltransferase 5 (Prmt5), mRNA.	1.4	2.30E-05
217011	ILMN_239279	Mus musculus notchless homolog 1 (Drosophila) (Nle1), mRNA.	1.4	7.10E-05
	ILMN_211691		1.4	2.40E-06
18432	Mybbp1a	MYB binding protein (P160) 1a	1.4	0.00015
270058	Mtap1s	microtubule-associated protein 1S	1.4	1.00E-05
66902	Mtap	methylthioadenosine phosphorylase	1.4	1.20E-05
17220	Mcm7	minichromosome maintenance deficient 7 (S. cerevisiae)	1.4	0.00029
14450	Gart	phosphoribosylglycinamide formyltransferase	1.4	2.80E-05
27407	Abcf2	ATP-binding cassette, sub-family F (GCN20), member 2	1.4	1.00E-05
11906	ILMN_192544	Mus musculus zinc finger homeobox 3 (Zfhx3), mRNA.	1.39	6.60E-07
319757	Smo	smoothened homolog (Drosophila)	1.39	4.20E-07
19891	Rpa2	replication protein A2	1.39	3.60E-05
29870	Gtse1	G two S phase expressed protein 1	1.39	3.10E-05
56505	Ruvbl1	RuvB-like protein 1	1.38	2.70E-07
106344	Rfc4	replication factor C (activator 1) 4	1.38	0.00015
78929	ILMN_209245	Mus musculus polymerase (RNA) III (DNA directed) polypeptide H (Polr3h), mRNA.	1.38	8.20E-07
18008	Nes	nestin	1.38	1.10E-06
17975	Ncl	nucleolin	1.38	2.40E-06
70083	Metrn	meteorin, glial cell differentiation regulator	1.38	1.80E-05
15516	Hsp90ab1	heat shock protein 90 alpha (cytosolic), class B member 1	1.38	1.70E-05
22059	Trp53	transformation related protein 53	1.37	7.50E-06
66131	Tipin	timeless interacting protein	1.37	6.50E-06
56390	Sssca1	Sjogren's syndrome/scleroderma autoantigen 1 homolog (human)	1.37	5.40E-07
73296	Rhobtb3	Rho-related BTB domain containing 3	1.37	5.10E-07
245688	ILMN_215499	Mus musculus retinoblastoma binding protein 7 (Rbbp7), mRNA.	1.37	0.00014
14208	Ppm1g	protein phosphatase 1G (formerly 2C), magnesium-dependent, gamma isoform	1.37	3.90E-06
17768	Mthfd2	methylenetetrahydrofolate dehydrogenase (NAD+ dependent), methenyltetrahydrofolate cyclohydrolase	1.37	1.30E-05
17089	Lyar	Ly1 antibody reactive clone	1.37	9.70E-06
70465	ILMN_214857	Mus musculus WD repeat domain 77 (Wdr77), mRNA.	1.36	2.00E-07
66409	Rsl1d1	ribosomal L1 domain containing 1	1.36	1.60E-06
20088	Rps24	ribosomal protein S24	1.36	7.20E-05
20019	Polr1a	polymerase (RNA) I polypeptide A	1.36	1.70E-06
100046883	ILMN_217094	PREDICTED: Mus musculus similar to CKLF-like MARVEL transmembrane domain containing 3 (LOC100046883), mRNA.	1.36	5.00E-05
27041	G3bp1	Ras-GTPase-activating protein SH3-domain binding protein 1	1.36	2.30E-07
13681	Eif4a1	eukaryotic translation initiation factor 4A1	1.36	1.20E-06
12738	Cldn2	claudin 2	1.36	9.70E-06
56222	Cited4	Cbp/p300-interacting transactivator, with Glu/Asp-rich carboxy-terminal domain, 4	1.36	4.00E-07
67236	Cinp	cyclin-dependent kinase 2 interacting protein	1.36	2.00E-06
21414	Tcf7	transcription factor 7, T-cell specific	1.35	2.10E-07
110816	ILMN_212496	Mus musculus PWP2 periodic tryptophan protein homolog (yeast) (Pwp2), mRNA.	1.35	3.20E-05
64424	ILMN_189434	Mus musculus polymerase (RNA) I polypeptide E (Polr1e), mRNA.	1.35	3.10E-05
26425	Nubp1	nucleotide binding protein 1	1.35	4.60E-07
100608	Noc4l	nucleolar complex associated 4 homolog (S. cerevisiae)	1.35	9.40E-05
27756	Lsm2	LSM2 homolog, U6 small nuclear RNA associated (S. cerevisiae)	1.35	1.00E-06
217995	ILMN_214317	Mus musculus HEAT repeat containing 1 (Heatr1), mRNA.	1.35	6.80E-05
13555	E2f1	E2F transcription factor 1	1.35	1.70E-07
21915	Dtymk	deoxythymidylate kinase	1.35	0.00016
66976	ILMN_212730	Mus musculus RIKEN cDNA 2410002F23 gene (2410002F23Rik), mRNA.	1.35	2.80E-05
80914	Uck2	uridine-cytidine kinase 2	1.34	2.70E-06
68926	Ubap2	ubiquitin-associated protein 2	1.34	5.40E-07
213391	Rassf4	Ras association (RalGDS/AF-6) domain family member 4	1.34	3.30E-05
18391	Sigmar1	sigma non-opioid intracellular receptor 1	1.34	1.50E-06
18221	Nudc	nuclear distribution gene C homolog (Aspergillus)	1.34	2.70E-05
15516	Hsp90ab1	heat shock protein 90 alpha (cytosolic), class B member 1	1.34	9.40E-05
101612	Grwd1	glutamate-rich WD repeat containing 1	1.34	7.00E-04
56149	Grasp	GRP1 (general receptor for phosphoinositides 1)-associated scaffold protein	1.34	7.70E-05
13007	Csrp1	cysteine and glycine-rich protein 1	1.34	1.20E-05
56279	Fam69b	family with sequence similarity 69, member B	1.34	3.00E-06
223626	ILMN_219117	Mus musculus RIKEN cDNA 4930572J05 gene (4930572J05Rik), mRNA.	1.34	0.0016
66497	2610528E23Rik	RIKEN cDNA 2610528E23 gene	1.34	2.60E-05
56520	Nme4	non-metastatic cells 4, protein expressed in	1.33	1.50E-05
17304	Mfge8	milk fat globule-EGF factor 8 protein	1.33	1.80E-05
100087	Kti12	KTI12 homolog, chromatin associated (S. cerevisiae)	1.33	5.30E-05
14534	Kat2a	K(lysine) acetyltransferase 2A	1.33	5.90E-06
71780	Isyna1	myo-inositol 1-phosphate synthase A1	1.33	4.10E-05
14635	Galk1	galactokinase 1	1.33	2.50E-07
14229	Fkbp5	FK506 binding protein 5	1.33	0.0067
207521	Dtx4	deltex 4 homolog (Drosophila)	1.33	4.10E-07
12223	Btc	betacellulin, epidermal growth factor family member	1.33	0.00043
56369	Apip	APAF1 interacting protein	1.33	5.50E-06
233066	AI428936	expressed sequence AI428936	1.33	4.20E-06
27407	Abcf2	ATP-binding cassette, sub-family F (GCN20), member 2	1.33	1.60E-07
70435	ILMN_222437	Mus musculus RIKEN cDNA 2610204M08 gene (2610204M08Rik), mRNA.	1.33	0.00025
20621	Snn	stannin	1.32	0.00022
56190	Rbm38	RNA binding motif protein 38	1.32	0.00013
100044829	ILMN_209238	PREDICTED: Mus musculus similar to Fibrillarin, transcript variant 1 (LOC100044829), mRNA.	1.32	1.10E-06
70572	Ipo5	importin 5	1.32	4.40E-07
	ILMN_208668		1.32	3.00E-06
80876	Ifitm2	interferon induced transmembrane protein 2	1.32	7.90E-05
233876	Hirip3	HIRA interacting protein 3	1.32	1.70E-07
14287	Fpgs	folylpolyglutamyl synthetase	1.32	3.90E-06
75458	Cklf	chemokine-like factor	1.32	4.90E-06
108673	Ccdc86	coiled-coil domain containing 86	1.32	0.00016
77045	Bcl7a	B-cell CLL/lymphoma 7A	1.32	3.00E-05
78354	2210407C18Rik	RIKEN cDNA 2210407C18 gene	1.32	0.002
72322	Xpo5	exportin 5	1.31	1.10E-05
71354	Wdr31	WD repeat domain 31	1.31	3.50E-05
74196	Ttc27	tetratricopeptide repeat domain 27	1.31	1.20E-07
18817	Plk1	polo-like kinase 1 (Drosophila)	1.31	0.00024
69912	Nup43	nucleoporin 43	1.31	1.70E-07
100047155	ILMN_221233	PREDICTED: Mus musculus similar to Small nuclear ribonucleoprotein polypeptide A (LOC100047155), mRNA.	1.31	8.80E-07

29876	Clic4	chloride intracellular channel 4 (mitochondrial)	1.31	8.70E-06
11837	Rplp0	ribosomal protein, large, P0	1.3	9.10E-06
56190	Rbm38	RNA binding motif protein 38	1.3	0.00015
	ILMN_222473		1.3	1.30E-05
67619	Nob1	NIN1/RPN12 binding protein 1 homolog (S. cerevisiae)	1.3	2.50E-05
234549	ILMN_208905	Mus musculus HEAT repeat containing 3 (Heatr3), mRNA.	1.3	0.00015
207521	Dtx4	deltex 4 homolog (Drosophila)	1.3	1.80E-05
229473	D930015E06Rik	RIKEN cDNA D930015E06 gene	1.3	6.50E-06
12236	Bub1b	budding uninhibited by benzimidazoles 1 homolog, beta (S. cerevisiae)	1.3	3.40E-05
72657	2700094K13Rik	RIKEN cDNA 2700094K13 gene	1.3	4.90E-06
66356	2310008H09Rik	RIKEN cDNA 2310008H09 gene	1.3	2.70E-07
107071	Wdr74	WD repeat domain 74	1.29	1.10E-05
73710	Tubb2b	tubulin, beta 2B	1.29	1.20E-06
72960	Top1mt	DNA topoisomerase 1, mitochondrial	1.29	3.80E-06
104886	ILMN_218127	Mus musculus RAB15, member RAS oncogene family (Rab15), mRNA.	1.29	5.20E-06
27374	Prmt5	protein arginine N-methyltransferase 5	1.29	6.60E-06
68106	Nt5c3l	5'-nucleotidase, cytosolic III-like	1.29	7.50E-06
67134	Nop56	NOP56 ribonucleoprotein homolog (yeast)	1.29	0.00017
17865	ILMN_212854	Mus musculus myeloblastosis oncogene-like 2 (Mybl2), mRNA.	1.29	7.50E-09
434858	ILMN_233946	Mus musculus predicted gene, EG434858 (EG434858), non-coding RNA.	1.29	0.002
66143	Eef1e1	eukaryotic translation elongation factor 1 epsilon 1	1.29	3.20E-05
21771	Cirh1a	cirrhosis, autosomal recessive 1A (human)	1.29	1.30E-05
26886	Cenph	centromere protein H	1.29	0.00033
74254	Gpn1	GPN-loop GTPase 1	1.28	1.80E-05
56753	Tacstd2	tumor-associated calcium signal transducer 2	1.28	2.60E-07
72462	Rrp1b	ribosomal RNA processing 1 homolog B (S. cerevisiae)	1.28	0.01
107094	Rrp12	ribosomal RNA processing 12 homolog (S. cerevisiae)	1.28	1.10E-05
245688	ILMN_215499	Mus musculus retinoblastoma binding protein 7 (Rbbp7), mRNA.	1.28	0.00024
27280	Phlda3	pleckstrin homology-like domain, family A, member 3	1.28	0.0041
230721	Pabpc4	poly(A) binding protein, cytoplasmic 4	1.28	9.40E-05
97961	Nol12	nucleolar protein 12	1.28	3.40E-06
66164	Nip7	nuclear import 7 homolog (S. cerevisiae)	1.28	2.50E-06
17748	Mt1	metallothionein 1	1.28	0.00052
98386	Lbr	lamin B receptor	1.28	4.40E-05
15510	Hspd1	heat shock protein 1 (chaperonin)	1.28	1.00E-06
30877	Gnl3	guanine nucleotide binding protein-like 3 (nucleolar)	1.28	2.90E-05
75458	Cklf	chemokine-like factor	1.28	5.70E-06
69928	Apitd1	apoptosis-inducing, TAF9-like domain 1	1.28	0.00019
66409	Rsl1d1	ribosomal L1 domain containing 1	1.27	0.00028
245688	ILMN_215499	Mus musculus retinoblastoma binding protein 7 (Rbbp7), mRNA.	1.27	0.0011
214572	Prmt7	protein arginine N-methyltransferase 7	1.27	6.50E-06
29858	Pmm1	phosphomannomutase 1	1.27	0.00013
384793	ILMN_246137	Mus musculus nuclear distribution gene C homolog (Aspergillus), pseudogene 1 (Nudc-ps1), non-coding RNA.	1.27	3.40E-05
217431	Nol10	nucleolar protein 10	1.27	2.00E-05
110109	Nop2	NOP2 nucleolar protein homolog (yeast)	1.27	7.10E-05
100045484	ILMN_211590	PREDICTED: Mus musculus similar to GFRalpha-3 (LOC100045484), mRNA.	1.27	2.70E-05
226519	ILMN_232528	Mus musculus laminin, gamma 1 (Lamc1), mRNA.	1.27	0.00017
23872	Ets2	E26 avian leukemia oncogene 2, 3' domain	1.27	8.20E-06
20624	Eftud2	elongation factor Tu GTP binding domain containing 2	1.27	6.90E-07
52874	D19Bwg1357e	DNA segment, Chr 19, Brigham & Women's Genetics 1357 expressed	1.27	1.30E-05
59053	Fam203a	family with sequence similarity 203, member A	1.27	4.40E-06
12144	Blm	Bloom syndrome, RecQ helicase-like	1.27	9.20E-06
494504	Apcdd1	adenomatosis polyposis coli down-regulated 1	1.27	3.00E-06
17025	ILMN_215056	Mus musculus aminolevulinate, delta-, dehydratase (Alad), mRNA.	1.27	6.70E-06
22419	Wnt5b	wingless-related MMTV integration site 5B	1.26	1.90E-07
	ILMN_213714		1.26	0.00011
93737	Pard6g	par-6 partitioning defective 6 homolog gamma (C. elegans)	1.26	1.70E-06
18221	Nudc	nuclear distribution gene C homolog (Aspergillus)	1.26	5.10E-07
17219	Mcm6	minichromosome maintenance deficient 6 (MIS5 homolog, S. pombe) (S. cerevisiae)	1.26	0.00012
107351	Kank1	KN motif and ankyrin repeat domains 1	1.26	4.20E-06
23917	Impdh1	inosine 5'-phosphate dehydrogenase 1	1.26	0.0063
69639	Exosc8	exosome component 8	1.26	8.20E-06
14026	ILMN_221136	Mus musculus Ena-vasodilator stimulated phosphoprotein (Evl), mRNA. XM_923143	1.26	2.50E-05
74107	Cep55	centrosomal protein 55	1.26	3.10E-05
	ILMN_212926		1.26	0.00075
319278	A230050P20Rik	RIKEN cDNA A230050P20 gene	1.26	0.00015
19027	Sypl	synaptophysin-like protein	1.25	1.30E-06
66406	Sac3d1	SAC3 domain containing 1	1.25	0.00017
58887	ILMN_210453	Mus musculus replication initiator 1 (Repin1), transcript variant 5, mRNA.	1.25	8.70E-06
213391	Rassf4	Ras association (RalGDS/AF-6) domain family member 4	1.25	4.10E-05
214572	Prmt7	protein arginine N-methyltransferase 7	1.25	1.20E-06
52033	Pbk	PDZ binding kinase	1.25	8.90E-06
100046953	ILMN_213254	PREDICTED: Mus musculus similar to Rab6 interacting protein 1 (LOC100046953), mRNA.	1.25	1.90E-07
231670	Fbxo21	F-box protein 21	1.25	3.60E-05
211651	Fancd2	Fanconi anemia, complementation group D2	1.25	7.90E-06
12144	Blm	Bloom syndrome, RecQ helicase-like	1.25	5.00E-06
76895	Bicd2	bicaudal D homolog 2 (Drosophila)	1.25	5.40E-05
66637	Tsen15	tRNA splicing endonuclease 15 homolog (S. cerevisiae)	1.25	4.10E-06
67671	Rpl38	ribosomal protein L38	1.24	5.00E-04
19366	Rad54l	RAD54 like (S. cerevisiae)	1.24	5.10E-07
52683	Ncaph2	non-SMC condensin II complex, subunit H2	1.24	6.60E-07
100047490	ILMN_221509	PREDICTED: Mus musculus similar to putative oral cancer suppressor (LOC100047490), mRNA.	1.24	5.40E-07
246293	Klhl8	kelch-like 8 (Drosophila)	1.24	1.90E-05
	ILMN_186180		1.24	5.70E-07
14252	Flot2	flotillin 2	1.24	1.70E-05
20624	Eftud2	elongation factor Tu GTP binding domain containing 2	1.24	3.90E-06
66336	Cenpp	centromere protein P	1.24	1.50E-05
12462	Cct3	chaperonin containing Tcp1, subunit 3 (gamma)	1.24	1.20E-05
20308	Ccl9	chemokine (C-C motif) ligand 9	1.24	0.00024
66912	Bzw2	basic leucine zipper and W2 domains 2	1.24	4.00E-05
22340	Vegfb	vascular endothelial growth factor B	1.23	1.00E-04
30954	Siva1	SIVA1, apoptosis-inducing factor	1.23	3.80E-05
20425	Shmt1	serine hydroxymethyltransferase 1 (soluble)	1.23	0.00015
75416	Nop14	NOP14 nucleolar protein homolog (yeast)	1.23	3.20E-05
68092	Ncbp2	nuclear cap binding protein subunit 2	1.23	2.70E-06
677205	ILMN_212422	PREDICTED: Mus musculus similar to DEAD (Asp-Glu-Ala-Asp) box polypeptide 18 (LOC677205), misc RNA.	1.23	1.60E-06
15505	Hsph1	heat shock 105kDa/110kDa protein 1	1.23	1.00E-04
73284	Ddit4l	DNA-damage-inducible transcript 4-like	1.23	0.0036
68183	Bcas2	breast carcinoma amplified sequence 2	1.23	1.20E-05
71449	ILMN_221329	Mus musculus RIKEN cDNA 5630401D24 gene (5630401D24Rik), mRNA.	1.23	0.00034
74237	Tubgcp2	tubulin, gamma complex associated protein 2	1.22	1.00E-06
66637	Tsen15	tRNA splicing endonuclease 15 homolog (S. cerevisiae)	1.22	2.00E-04
101739	Psip1	PC4 and SFRS1 interacting protein 1	1.22	1.40E-05
54563	Nup210	nucleoporin 210	1.22	1.70E-05
66225	ILMN_209045	Mus musculus LLP homolog, long-term synaptic facilitation (Aplysia) (Llph), mRNA.	1.22	2.70E-06
69237	Gtpbp4	GTP binding protein 4	1.22	2.40E-06
14719	Got2	glutamate oxaloacetate transaminase 2, mitochondrial	1.22	6.30E-07
56095	Ftsj3	FtsJ homolog 3 (E. coli)	1.22	6.60E-05
71963	Cdca4	cell division cycle associated 4	1.22	6.00E-07
381101	BC048355	cDNA sequence BC048355	1.22	5.10E-05
230234	BC026590	cDNA sequence BC026590	1.22	3.00E-04
213539	Bag2	BCL2-associated athanogene 2	1.22	1.20E-07
104732	4930427A07Rik	RIKEN cDNA 4930427A07 gene	1.22	8.70E-05
56412	2610024G14Rik	RIKEN cDNA 2610024G14 gene	1.22	1.30E-05
230734	Yrdc	yrdC domain containing (E.coli)	1.21	6.20E-05
28035	Usp39	ubiquitin specific peptidase 39	1.21	0.00022
20907	Stx1a	syntaxin 1A (brain)	1.21	1.80E-06

71729	Rgs12	regulator of G-protein signaling 12	1.21	0.00015
19072	Prep	prolyl endopeptidase	1.21	6.90E-08
68606	Ppm1f	protein phosphatase 1F (PP2C domain containing)	1.21	2.20E-06
11545	Parp1	poly (ADP-ribose) polymerase family, member 1	1.21	1.50E-06
70024	Mcm10	minichromosome maintenance deficient 10 (S. cerevisiae)	1.21	4.30E-07
16881	Lig1	ligase I, DNA, ATP-dependent	1.21	2.00E-05
15186	Hdc	histidine decarboxylase	1.21	0.039
50496	ILMN_220573	Mus musculus E2F transcription factor 6 (E2f6), mRNA.	1.21	0.00033
12411	Cbs	cystathionine beta-synthase	1.21	3.50E-07
69719	Cad	carbamoyl-phosphate synthetase 2, aspartate transcarbamylase, and dihydroorotase	1.21	0.00027
230376	Haus6	HAUS augmin-like complex, subunit 6	1.21	0.00032
104367	Snora65	small nucleolar RNA, H/ACA box 65	1.2	4.40E-05
227059	Slc39a10	solute carrier family 39 (zinc transporter), member 10	1.2	6.40E-06
217847	Serpina10	serine (or cysteine) peptidase inhibitor, clade A (alpha-1 antiproteinase, antitrypsin), member 10	1.2	0.00037
100088	Rcc1	regulator of chromosome condensation 1	1.2	1.20E-05
445007	Nup85	nucleoporin 85	1.2	0.0014
57753	Noc3l	nucleolar complex associated 3 homolog (S. cerevisiae)	1.2	1.50E-07
17219	Mcm6	minichromosome maintenance deficient 6 (MIS5 homolog, S. pombe) (S. cerevisiae)	1.2	3.00E-07
230233	ILMN_194092	Mus musculus inhibitor of kappa light polypeptide enhancer in B-cells, kinase complex-associated protein (Ikbkap), mRNA.	1.2	3.00E-06
15937	Ier3	immediate early response 3	1.2	4.40E-05
228366	Gyltl1b	glycosyltransferase-like 1B	1.2	5.60E-06
27979	Eif3b	eukaryotic translation initiation factor 3, subunit B	1.2	3.40E-05
13106	Cyp2e1	cytochrome P450, family 2, subfamily e, polypeptide 1	1.2	5.60E-06
12028	Bax	BCL2-associated X protein	1.2	0.00027
214579	Aldh5a1	aldhehyde dehydrogenase family 5, subfamily A1	1.2	3.40E-08
68026	2810417H13Rik	RIKEN cDNA 2810417H13 gene	1.2	0.00086
73635	1700113I22Rik	RIKEN cDNA 1700113I22 gene	1.2	1.80E-06
83669	Wdr6	WD repeat domain 6	1.19	0.00028
20931	Surf2	surfeit gene 2	1.19	1.50E-06
70358	Steap1	six transmembrane epithelial antigen of the prostate 1	1.19	2.50E-06
20496	ILMN_211631	Mus musculus solute carrier family 12, member 2 (Slc12a2), mRNA.	1.19	3.10E-05
22121	Rpl13a	ribosomal protein L13A	1.19	4.80E-05
69241	Polr2d	polymerase (RNA) II (DNA directed) polypeptide D	1.19	4.10E-08
56520	Nme4	non-metastatic cells 4, protein expressed in	1.19	5.50E-05
17938	Naca	nascent polypeptide-associated complex alpha polypeptide	1.19	6.60E-07
107435	Hat1	histone aminotransferase 1	1.19	3.40E-06
228366	Gyltl1b	glycosyltransferase-like 1B	1.19	2.30E-05
14776	Gpx2	glutathione peroxidase 2	1.19	4.20E-06
50496	ILMN_220573	Mus musculus E2F transcription factor 6 (E2f6), mRNA.	1.19	5.50E-06
228889	Ddx27	DEAD (Asp-Glu-Ala-Asp) box polypeptide 27	1.19	4.20E-06
68087	ILMN_218673	Mus musculus dephospho-CoA kinase domain containing (Dcakd), mRNA.	1.19	3.80E-07
51797	Ctps	cytidine 5'-triphosphate synthase	1.19	0.00013
207785	Csrnp2	cysteine-serine-rich nuclear protein 2	1.19	7.30E-07
72155	Cenpn	centromere protein N	1.19	6.80E-05
212547	ILMN_244646	Mus musculus cDNA sequence BC027231 (BC027231), mRNA.	1.19	1.00E-05
72635	Lins	lines homolog (Drosophila)	1.18	2.10E-06
67534	Ttll4	tubulin tyrosine ligase-like family, member 4	1.18	2.50E-05
330836	Slc7a6	solute carrier family 7 (cationic amino acid transporter, y+ system), member 6	1.18	1.80E-06
60507	ILMN_219623	Mus musculus queuine tRNA-ribosyltransferase 1 (Qtrt1), mRNA.	1.18	1.50E-07
107607	Nod1	nucleotide-binding oligomerization domain containing 1	1.18	1.40E-05
17535	Mre11a	meiotic recombination 11 homolog A (S. cerevisiae)	1.18	3.80E-06
57905	Isy1	ISY1 splicing factor homolog (S. cerevisiae)	1.18	2.80E-06
209357	ILMN_185469	Mus musculus general transcription factor IIH, polypeptide 3 (Gtf2h3), mRNA.	1.18	3.20E-06
14866	Gstm5	glutathione S-transferase, mu 5	1.18	2.00E-05
268527	ILMN_242588	Mus musculus gene regulated by estrogen in breast cancer protein (Greb1), mRNA.	1.18	0.00024
23886	Gdf15	growth differentiation factor 15	1.18	0.037
233908	Fus	fusion, derived from t(12;16) malignant liposarcoma (human)	1.18	0.0031
192176	Flna	filamin, alpha	1.18	0.00024
50496	E2f6	E2F transcription factor 6	1.18	5.70E-05
66570	Cenpm	centromere protein M	1.18	3.20E-07
330050	Fam185a	family with sequence similarity 185, member A	1.18	2.00E-06
11906	Zfhx3	zinc finger homeobox 3	1.17	4.20E-06
71446	Wrb	tryptophan rich basic protein	1.17	5.30E-06
73674	Wdr75	WD repeat domain 75	1.17	2.00E-05
27366	Txnl4a	thioredoxin-like 4A	1.17	7.30E-06
326618	Tpm4	tropomyosin 4	1.17	8.70E-05
209588	Sectm1a	secreted and transmembrane 1A	1.17	0.00032
80837	Rhoj	ras homolog gene family, member J	1.17	0.0012
18971	Pold1	polymerase (DNA directed), delta 1, catalytic subunit	1.17	9.00E-05
18969	Pola2	polymerase (DNA directed), alpha 2	1.17	8.90E-06
234865	Nup133	nucleoporin 133	1.17	0.00086
68106	Nt5c3l	5'-nucleotidase, cytosolic III-like	1.17	1.70E-05
404710	ILMN_250781	Mus musculus IQ motif containing GTPase activating protein 3 (Iqgap3), mRNA. XM_980709 XM_987883 XM_987929 XM_987972 XM_988004 XM_988041 XM_988104	1.17	0.0012
15375	ILMN_221873	Mus musculus forkhead box A1 (Foxa1), mRNA.	1.17	3.40E-06
72544	Exosc6	exosome component 6	1.17	1.70E-05
232813	Shisa7	shisa homolog 7 (Xenopus laevis)	1.17	8.90E-06
56693	Crtap	cartilage associated protein	1.17	1.10E-05
67291	Ccdc137	coiled-coil domain containing 137	1.17	3.30E-05
217737	Ahsa1	AHA1, activator of heat shock protein ATPase homolog 1 (yeast)	1.17	1.10E-06
215193	Diexf	digestive organ expansion factor homolog (zebrafish)	1.17	8.90E-06
19387	ILMN_258002	Mus musculus RAN GTPase activating protein 1 (Rangap1), mRNA.	1.16	2.30E-07
110639	Prps2	phosphoribosyl pyrophosphate synthetase 2	1.16	0.00091
18780	Pla2g2a	phospholipase A2, group IIA (platelets, synovial fluid)	1.16	0.0045
18538	ILMN_223289	Mus musculus proliferating cell nuclear antigen (Pcna), mRNA.	1.16	0.00065
67619	Nob1	NIN1/RPN12 binding protein 1 homolog (S. cerevisiae)	1.16	0.00045
77286	Nkrf	NF-kappaB repressing factor	1.16	5.00E-06
17975	Ncl	nucleolin	1.16	4.60E-06
50927	ILMN_211691	Mus musculus nuclear autoantigenic sperm protein (histone-binding) (Nasp), transcript variant 2, mRNA.	1.16	3.30E-05
59024	Med12	mediator of RNA polymerase II transcription, subunit 12 homolog (yeast)	1.16	6.20E-06
75751	Ipo4	importin 4	1.16	2.70E-05
77065	Ints7	integrator complex subunit 7	1.16	2.70E-05
15516	Hsp90ab1	heat shock protein 90 alpha (cytosolic), class B member 1	1.16	6.50E-05
72042	Cotl1	coactosin-like 1 (Dictyostelium)	1.16	1.70E-05
74268	Aven	apoptosis, caspase activation inhibitor	1.16	9.10E-05
106877	Afap1l1	actin filament associated protein 1-like 1	1.16	1.20E-05
72155	Cenpn	centromere protein N	1.16	3.40E-06
232679	Zc3hc1	zinc finger, C3HC type 1	1.15	2.80E-05
109161	Ube2q2	ubiquitin-conjugating enzyme E2Q (putative) 2	1.15	2.80E-06
21853	ILMN_246642	Mus musculus timeless homolog (Drosophila) (Timeless), transcript variant 2, mRNA.	1.15	0.00012
20641	Snrpd1	small nuclear ribonucleoprotein D1	1.15	1.80E-05
332397	Nanos1	nanos homolog 1 (Drosophila)	1.15	2.30E-05
76890	ILMN_211072	Mus musculus mediator of cell motility 1 (Memo1), mRNA.	1.15	0.006
100043257	ILMN_215552	PREDICTED: Mus musculus similar to RNA binding motif protein 3 (LOC100043257), mRNA.	1.15	1.80E-06
16881	Lig1	ligase I, DNA, ATP-dependent	1.15	0.00032
60530	Fignl1	fidgetin-like 1	1.15	2.00E-05
56200	Ddx21	DEAD (Asp-Glu-Ala-Asp) box polypeptide 21	1.15	0.0015
66570	Cenpm	centromere protein M	1.15	1.90E-06
12527	Cd9	CD9 antigen	1.15	4.80E-05
12462	Cct3	chaperonin containing Tcp1, subunit 3 (gamma)	1.15	2.70E-05
381903	Alg8	asparagine-linked glycosylation 8 homolog (yeast, alpha-1,3-glucosyltransferase)	1.15	0.00018
240675	Vwa2	von Willebrand factor A domain containing 2	1.14	5.50E-05
80914	Uck2	uridine-cytidine kinase 2	1.14	1.70E-05
22142	Tuba1a	tubulin, alpha 1A	1.14	6.20E-05
21815	Tgif1	TGFB-induced factor homeobox 1	1.14	0.00015
19027	Sypl	synaptophysin-like protein	1.14	1.90E-05
20638	Snrpb	small nuclear ribonucleoprotein B	1.14	2.40E-05
20638	Snrpb	small nuclear ribonucleoprotein B	1.14	5.30E-06

98365	Slamf9	SLAM family member 9	1.14	0.0016
20969	Sdc1	syndecan 1	1.14	1.50E-05
59028	Rcl1	RNA terminal phosphate cyclase-like 1	1.14	0.00075
11545	Parp1	poly (ADP-ribose) polymerase family, member 1	1.14	3.10E-05
234865	Nup133	nucleoporin 133	1.14	2.80E-05
18029	Nfic	nuclear factor I/C	1.14	0.011
17068	Ly6d	lymphocyte antigen 6 complex, locus D	1.14	0.02
66667	Hspbap1	Hspb associated protein 1	1.14	2.80E-05
14756	Gpld1	glycosylphosphatidylinositol specific phospholipase D1	1.14	1.10E-05
14457	Gas7	growth arrest specific 7	1.14	1.00E-05
66583	Exosc1	exosome component 1	1.14	1.60E-05
276770	Eif5a	eukaryotic translation initiation factor 5A	1.14	4.10E-06
71919	Rpap3	RNA polymerase II associated protein 3	1.14	9.30E-05
71963	Cdca4	cell division cycle associated 4	1.14	3.00E-06
21939	ILMN_242411	Mus musculus CD40 antigen (Cd40), transcript variant 5, mRNA.	1.14	3.00E-04
22381	Wbp5	WW domain binding protein 5	1.13	2.30E-05
74044	Ttf2	transcription termination factor, RNA polymerase II	1.13	2.10E-05
21983	Tpbg	trophoblast glycoprotein	1.13	5.70E-06
56459	Sae1	SUMO1 activating enzyme subunit 1	1.13	1.80E-05
100609	Nsun5	NOL1/NOP2/Sun domain family, member 5	1.13	0.00011
20826	ILMN_210334	Mus musculus NHP2 non-histone chromosome protein 2-like 1 (S. cerevisiae) (Nhp2l1), mRNA.	1.13	3.00E-05
17535	Mre11a	meiotic recombination 11 homolog A (S. cerevisiae)	1.13	3.20E-07
70024	Mcm10	minichromosome maintenance deficient 10 (S. cerevisiae)	1.13	0.00012
546015	3010033P07Rik	PREDICTED: Mus musculus similar to ribosomal protein S9 (LOC546015), misc RNA.	1.13	5.50E-06
209737	ILMN_212864	Mus musculus kinesin family member 15 (Kif15), mRNA.	1.13	7.30E-06
69692	Hddc2	HD domain containing 2	1.13	4.00E-04
110006	Gusb	glucuronidase, beta	1.13	0.00065
381633	Gm1673	predicted gene 1673	1.13	0.00029
23834	Cdc6	cell division cycle 6 homolog (S. cerevisiae)	1.13	4.90E-06
76080	Ttpal	tocopherol (alpha) transfer protein-like	1.12	0.00015
20195	S100a11	S100 calcium binding protein A11 (calgizzarin)	1.12	2.00E-05
17463	Psmd7	proteasome (prosome, macropain) 26S subunit, non-ATPase, 7	1.12	0.00012
100609	Nsun5	NOL1/NOP2/Sun domain family, member 5	1.12	7.90E-06
218121	Mboat1	membrane bound O-acyltransferase domain containing 1	1.12	3.00E-04
16897	Llgl1	lethal giant larvae homolog 1 (Drosophila)	1.12	9.10E-08
171463	Il17rd	interleukin 17 receptor D	1.12	8.10E-05
55944	Eif3d	eukaryotic translation initiation factor 3, subunit D	1.12	9.40E-05
20624	Eftud2	elongation factor Tu GTP binding domain containing 2	1.12	2.20E-06
12615	Cenpa	centromere protein A	1.12	1.90E-06
52276	Cdca8	cell division cycle associated 8	1.12	2.10E-05
67832	Brix1	BRX1, biogenesis of ribosomes, homolog (S. cerevisiae)	1.12	3.10E-05
51800	Bok	BCL2-related ovarian killer protein	1.12	0.00035
67211	Armc10	armadillo repeat containing 10	1.12	4.80E-05
66966	Trit1	tRNA isopentenyltransferase 1	1.11	6.70E-05
21849	Trim28	tripartite motif-containing 28	1.11	3.00E-05
72787	Tmem48	transmembrane protein 48	1.11	2.20E-05
20637	Snrnp70	small nuclear ribonucleoprotein 70 (U1)	1.11	0.00021
56459	Sae1	SUMO1 activating enzyme subunit 1	1.11	4.90E-07
69263	Rfc3	replication factor C (activator 1) 3	1.11	3.80E-05
69833	Polr2f	polymerase (RNA) II (DNA directed) polypeptide F	1.11	1.10E-06
212503	Paox	polyamine oxidase (exo-N4-amino)	1.11	2.50E-05
68051	Nutf2	nuclear transport factor 2	1.11	2.80E-06
28114	Nsun2	NOL1/NOP2/Sun domain family member 2	1.11	2.30E-05
70603	Mutyh	mutY homolog (E. coli)	1.11	1.70E-05
69692	Hddc2	HD domain containing 2	1.11	1.30E-05
67553	Gstcd	glutathione S-transferase, C-terminal domain containing	1.11	1.40E-05
17178	Fxyd3	FXYD domain-containing ion transport regulator 3	1.11	0.00026
68730	Dus1l	dihydrouridine synthase 1-like (S. cerevisiae)	1.11	9.50E-07
11933	Atp1b3	ATPase, Na+/K+ transporting, beta 3 polypeptide	1.11	5.30E-06
77254	Yif1b	Yip1 interacting factor homolog B (S. cerevisiae)	1.1	2.90E-05
20588	Smarcc1	SWI/SNF related, matrix associated, actin dependent regulator of chromatin, subfamily c, member 1	1.1	0.00013
108116	Slco3a1	solute carrier organic anion transporter family, member 3a1	1.1	2.70E-05
68275	Rpa1	replication protein A1	1.1	7.60E-07
	ILMN_218045		1.1	2.40E-05
68799	Rgmb	RGM domain family, member B	1.1	0.0013
72151	Rfc5	replication factor C (activator 1) 5	1.1	9.80E-07
26433	Plod3	procollagen-lysine, 2-oxoglutarate 5-dioxygenase 3	1.1	4.40E-05
18641	Pfkl	phosphofructokinase, liver, B-type	1.1	4.90E-06
71041	Pcgf6	polycomb group ring finger 6	1.1	1.20E-05
23943	Esyt1	extended synaptotagmin-like protein 1	1.1	1.70E-05
16319	Incenp	inner centromere protein	1.1	0.00011
15528	ILMN_213386	Mus musculus heat shock protein 1 (chaperonin 10) (Hspe1), mRNA.	1.1	0.00022
209354	Eif2b1	eukaryotic translation initiation factor 2B, subunit 1 (alpha)	1.1	0.0015
13494	Drg1	developmentally regulated GTP binding protein 1	1.1	5.70E-05
216877	Dhx33	DEAH (Asp-Glu-Ala-His) box polypeptide 33	1.1	4.90E-07
70333	Cd3eap	CD3E antigen, epsilon polypeptide associated protein	1.1	0.00075
66912	Bzw2	basic leucine zipper and W2 domains 2	1.1	2.20E-06
51800	Bok	BCL2-related ovarian killer protein	1.1	2.30E-07
330671	B4galnt4	beta-1,4-N-acetyl-galactosaminyl transferase 4	1.1	1.00E-04
74205	Acsl3	acyl-CoA synthetase long-chain family member 3	1.1	8.40E-06
66320	Tmem208	transmembrane protein 208	1.09	9.20E-07
54141	Spag5	sperm associated antigen 5	1.09	2.50E-06
19173	Psmb5	proteasome (prosome, macropain) subunit, beta type 5	1.09	0.00056
71974	Prmt3	protein arginine N-methyltransferase 3	1.09	1.80E-05
53324	ILMN_211885	Mus musculus neuronal pentraxin 2 (Nptx2), mRNA.	1.09	2.70E-06
116871	Mta3	metastasis associated 3	1.09	4.00E-05
229524	Msto1	misato homolog 1 (Drosophila)	1.09	0.00025
17688	Msh6	mutS homolog 6 (E. coli)	1.09	4.20E-07
16201	Ilf3	interleukin enhancer binding factor 3	1.09	3.20E-06
14297	Fxn	frataxin	1.09	9.30E-06
23873	Faim	Fas apoptotic inhibitory molecule	1.09	9.60E-06
105298	ILMN_214840	Mus musculus ependymin related protein 1 (zebrafish) (Epdr1), mRNA.	1.09	1.10E-05
66757	Adat2	adenosine deaminase, tRNA-specific 2, TAD2 homolog (S. cerevisiae)	1.09	2.30E-07
217364	Engase	endo-beta-N-acetylglucosaminidase	1.09	0.00091
232680	Cpa2	carboxypeptidase A2, pancreatic	1.09	3.10E-05
66570	Cenpm	centromere protein M	1.09	5.10E-07
67239	Rpf2	ribosome production factor 2 homolog (S. cerevisiae)	1.09	2.40E-06
56321	Aatf	apoptosis antagonizing transcription factor	1.09	1.10E-05
56412	2610024G14Rik	RIKEN cDNA 2610024G14 gene	1.09	9.30E-06
66965	ILMN_198892	Mus musculus RIKEN cDNA 2310061F22 gene (2310061F22Rik), mRNA.	1.09	1.80E-06
22147	Tuba3b	tubulin, alpha 3B	1.08	0.00039
22099	Tsn	translin	1.08	0.0012
71982	Snx10	sorting nexin 10	1.08	6.60E-05
226830	Smyd2	SET and MYND domain containing 2	1.08	5.90E-06
107701	Sf3b4	splicing factor 3b, subunit 4	1.08	0.0012
108943	ILMN_214434	Mus musculus RNA (guanine-9-) methyltransferase domain containing 2 (Rg9mtd2), mRNA.	1.08	1.60E-05
18637	Pfdn2	prefoldin 2	1.08	7.00E-06
67399	Pdlim7	PDZ and LIM domain 7	1.08	6.50E-06
65102	Nif3l1	Ngg1 interacting factor 3-like 1 (S. pombe)	1.08	0.0012
215387	Ncaph	non-SMC condensin I complex, subunit H	1.08	0.00075
17869	Myc	myelocytomatosis oncogene	1.08	0.00038
50918	ILMN_221407	Mus musculus myeloid-associated differentiation marker (Myadm), mRNA.	1.08	4.60E-06
17299	Mettl1	methyltransferase like 1	1.08	5.30E-05
100045019	ILMN_195453	PREDICTED: Mus musculus similar to Tubulin, gamma 2 (LOC100045019), mRNA.	1.08	0.00015
107045	ILMN_234717	Mus musculus leucyl-tRNA synthetase (Lars), mRNA. XM_901187 XM_913429 XM_922755 XM_922767 XM_922771 XM_922775 XM_922782 XM_922785 XM_989215	1.08	2.40E-05
15382	ILMN_257803	Mus musculus heterogeneous nuclear ribonucleoprotein A1 (Hnrpa1), transcript variant 2, mRNA.	1.08	0.00096
14156	Fen1	flap structure specific endonuclease 1	1.08	2.80E-05

105988	Espl1	extra spindle poles-like 1 (S. cerevisiae)	1.08	2.20E-05
13494	Drg1	developmentally regulated GTP binding protein 1	1.08	5.00E-06
240514	Ccdc85b	coiled-coil domain containing 85B	1.08	0.00013
192120	Bspry	B-box and SPRY domain containing	1.08	2.00E-06
12033	Bcap29	B-cell receptor-associated protein 29	1.08	0.00047
27078	B9d1	B9 protein domain 1	1.08	2.50E-05
11601	Angpt2	angiopoietin 2	1.08	1.00E-04
59008	Anapc5	anaphase-promoting complex subunit 5	1.08	0.0018
68099	Fam92a	family with sequence similarity 92, member A	1.08	3.00E-06
68964	1500010J02Rik	RIKEN cDNA 1500010J02 gene	1.08	3.10E-07
71735	Lrwd1	leucine-rich repeats and WD repeat domain containing 1	1.08	5.60E-06
28035	Usp39	ubiquitin specific peptidase 39	1.07	0.00075
20867	Stip1	stress-induced phosphoprotein 1	1.07	1.20E-05
	ILMN_212426		1.07	4.40E-05
19367	Rad9	RAD9 homolog (S. pombe)	1.07	8.20E-06
214572	Prmt7	protein arginine N-methyltransferase 7	1.07	3.40E-05
67037	Pmf1	polyamine-modulated factor 1	1.07	2.40E-05
212503	ILMN_212962	Mus musculus polyamine oxidase (exo-N4-amino) (Paox), mRNA.	1.07	2.70E-05
110109	Nop2	NOP2 nucleolar protein homolog (yeast)	1.07	6.20E-05
68298	Ncapd2	non-SMC condensin I complex, subunit D2	1.07	0.00047
17938	Naca	nascent polypeptide-associated complex alpha polypeptide	1.07	8.20E-05
110033	Kif22	kinesin family member 22	1.07	4.30E-05
229504	ILMN_211620	Mus musculus interferon stimulated exonuclease gene 20-like 2 (Isg20l2), mRNA.	1.07	0.00065
105148	Iars	isoleucine-tRNA synthetase	1.07	5.80E-05
15481	Hspa8	heat shock protein 8	1.07	0.00015
381633	Gm1673	predicted gene 1673	1.07	0.0019
104346	Gas8	growth arrest specific 8	1.07	2.80E-05
15377	Foxa3	forkhead box A3	1.07	9.40E-05
22791	Dnajc2	DnaJ (Hsp40) homolog, subfamily C, member 2	1.07	0.00015
27214	Dbf4	DBF4 homolog (S. cerevisiae)	1.07	1.60E-05
27373	Csnk1e	casein kinase 1, epsilon	1.07	0.0063
12630	ILMN_212927	Mus musculus complement component factor i (Cfi), mRNA.	1.07	0.00026
12520	Cd81	CD81 antigen	1.07	4.90E-07
14025	Bcl11a	B-cell CLL/lymphoma 11A (zinc finger protein)	1.07	8.10E-05
212547	ILMN_218852	Mus musculus cDNA sequence BC027231 (BC027231), mRNA.	1.07	0.0036
225215	Rsl24d1	ribosomal L24 domain containing 1	1.07	3.80E-06
72141	Adpgk	ADP-dependent glucokinase	1.07	1.90E-05
109711	ILMN_242046	Mus musculus actinin, alpha 1 (Actn1), mRNA.	1.07	6.90E-05
242864	Napepld	N-acyl phosphatidylethanolamine phospholipase D	1.07	3.60E-06
77254	Yif1b	Yip1 interacting factor homolog B (S. cerevisiae)	1.07	0.00043
27366	Txnl4a	thioredoxin-like 4A	1.06	2.20E-06
56403	Syncrip	synaptotagmin binding, cytoplasmic RNA interacting protein	1.06	2.20E-05
77634	Snapc3	small nuclear RNA activating complex, polypeptide 3	1.06	3.30E-05
232187	Smyd5	SET and MYND domain containing 5	1.06	0.00051
50721	Sirt6	sirtuin 6 (silent mating type information regulation 2, homolog) 6 (S. cerevisiae)	1.06	8.60E-06
102866	Pls3	plastin 3 (T-isoform)	1.06	0.00091
18148	ILMN_252426	Mus musculus nucleophosmin 1 (Npm1), mRNA.	1.06	1.30E-05
228869	Ncoa5	nuclear receptor coactivator 5	1.06	2.10E-05
105837	Mtbp	Mdm2, transformed 3T3 cell double minute p53 binding protein	1.06	1.10E-05
56280	Mrpl37	mitochondrial ribosomal protein L37	1.06	1.10E-05
56307	Metap2	methionine aminopeptidase 2	1.06	2.00E-04
15510	ILMN_213620	Mus musculus heat shock protein 1 (chaperonin) (Hspd1), mRNA.	1.06	0.015
78455	Helz	helicase with zinc finger domain	1.06	0.003
30877	Gnl3	guanine nucleotide binding protein-like 3 (nucleolar)	1.06	0.0067
57436	Gabarapl1	gamma-aminobutyric acid (GABA) A receptor-associated protein-like 1	1.06	7.00E-04
212377	Mms22l	MMS22-like, DNA repair protein	1.06	0.00096
66656	ILMN_255308	Mus musculus eukaryotic translation elongation factor 1 delta (guanine nucleotide exchange protein) (Eef1d), transcript variant 1, mRNA.	1.06	5.80E-05
56455	ILMN_233335	Mus musculus dynein light chain LC8-type 1 (Dynll1), mRNA.	1.06	3.60E-06
67728	Dph2	DPH2 homolog (S. cerevisiae)	1.06	0.00064
12566	Cdk2	cyclin-dependent kinase 2	1.06	3.50E-05
268880	ILMN_219150	Mus musculus expressed sequence AI480653 (AI480653), mRNA.	1.06	4.30E-05
71952	2410016O06Rik	RIKEN cDNA 2410016O06 gene	1.06	0.00011
22687	Zfp259	zinc finger protein 259	1.05	5.00E-04
57773	Wdr4	WD repeat domain 4	1.05	0.00086
216156	Wdr18	WD repeat domain 18	1.05	2.40E-05
28035	Usp39	ubiquitin specific peptidase 39	1.05	1.90E-05
22256	Ung	uracil DNA glycosylase	1.05	0.00026
66125	Sf3b5	splicing factor 3b, subunit 5	1.05	0.002
60406	Sap30	sin3 associated polypeptide	1.05	1.80E-05
20174	Ruvbl2	RuvB-like protein 2	1.05	9.10E-06
19679	Pitpnm2	phosphatidylinositol transfer protein, membrane-associated 2	1.05	0.00015
66181	Nop10	NOP10 ribonucleoprotein homolog (yeast)	1.05	0.00051
68294	Mfsd10	major facilitator superfamily domain containing 10	1.05	6.50E-05
66978	Luc7l	Luc7 homolog (S. cerevisiae)-like	1.05	8.20E-05
67978	Tctn2	tectonic family member 2	1.05	0.00041
100047009	ILMN_210896	PREDICTED: Mus musculus hypothetical protein LOC100047009 (LOC100047009), misc RNA.	1.05	0.0012
105148	Iars	isoleucine-tRNA synthetase	1.05	5.30E-05
18605	Enpp1	ectonucleotide pyrophosphatase/phosphodiesterase 1	1.05	9.30E-05
27221	Chaf1a	chromatin assembly factor 1, subunit A (p150)	1.05	4.40E-06
102920	Cenpi	centromere protein I	1.05	0.0014
12466	ILMN_224361	Mus musculus chaperonin containing Tcp1, subunit 6a (zeta) (Cct6a), mRNA.	1.05	1.90E-06
17060	Blnk	B-cell linker	1.05	8.70E-05
230917	Tmem201	transmembrane protein 201	1.05	7.00E-04
11677	ILMN_191705	Mus musculus aldo-keto reductase family 1, member B3 (aldose reductase) (Akr1b3), mRNA.	1.05	3.90E-05
330173	ILMN_215581	Mus musculus RIKEN cDNA 2610524H06 gene (2610524H06Rik), mRNA.	1.05	2.60E-05
73710	Tubb2b	tubulin, beta 2B	1.04	1.50E-05
22051	Trip6	thyroid hormone receptor interactor 6	1.04	0.012
68539	Tmem109	transmembrane protein 109	1.04	1.60E-06
106957	Slc39a6	solute carrier family 39 (metal ion transporter), member 6	1.04	4.60E-06
67390	Rnmtl1	RNA methyltransferase like 1	1.04	0.00013
56361	Pus1	pseudouridine synthase 1	1.04	1.60E-05
67203	Nde1	nuclear distribution gene E homolog 1 (A nidulans)	1.04	8.70E-05
67219	ILMN_208709	Mus musculus mediator of RNA polymerase II transcription, subunit 18 homolog (yeast) (Med18), mRNA.	1.04	2.70E-06
17192	Mbd3	methyl-CpG binding domain protein 3	1.04	1.00E-04
99010	Lpcat4	lysophosphatidylcholine acyltransferase 4	1.04	0.0016
16211	Kpnb1	karyopherin (importin) beta 1	1.04	6.30E-05
16525	Kcnk1	potassium channel, subfamily K, member 1	1.04	1.50E-07
320299	Iqcb1	IQ calmodulin-binding motif containing 1	1.04	1.10E-05
114663	Impa2	inositol (myo)-1(or 4)-monophosphatase 2	1.04	6.50E-06
269252	Gtf3c4	general transcription factor IIIC, polypeptide 4	1.04	4.10E-06
223666	Arhgap39	Rho GTPase activating protein 39	1.04	2.00E-04
71963	Cdca4	cell division cycle associated 4	1.04	1.70E-05
75565	ILMN_215478	Mus musculus coiled-coil domain containing 101 (Ccdc101), mRNA.	1.04	0.00018
56412	2610024G14Rik	RIKEN cDNA 2610024G14 gene	1.04	3.30E-05
230917	Tmem201	transmembrane protein 201	1.03	1.80E-05
21454	Tcp1	t-complex protein 1	1.03	0.0027
226830	Smyd2	SET and MYND domain containing 2	1.03	0.00028
116914	Slc19a2	solute carrier family 19 (thiamine transporter), member 2	1.03	0.0031
66083	Setd6	SET domain containing 6	1.03	7.10E-05
78895	Pus7l	pseudouridylate synthase 7 homolog (S. cerevisiae)-like	1.03	1.70E-05
107939	Pom121	nuclear pore membrane protein 121	1.03	0.00029
108083	Pip4k2b	phosphatidylinositol-5-phosphate 4-kinase, type II, beta	1.03	8.70E-06
56698	Phax	phosphorylated adaptor for RNA export	1.03	0.0023
17688	Msh6	mutS homolog 6 (E. coli)	1.03	9.10E-06
72050	Kdelc1	KDEL (Lys-Asp-Glu-Leu) containing 1	1.03	2.70E-05
27998	Exosc5	exosome component 5	1.03	3.40E-05
12729	Clns1a	chloride channel, nucleotide-sensitive, 1A	1.03	0.00018

214901	Chtf18	CTF18, chromosome transmission fidelity factor 18 homolog (S. cerevisiae)	1.03	4.90E-05
12400	Cbfb	core binding factor beta	1.03	5.10E-05
12033	Bcap29	B-cell receptor-associated protein 29	1.03	0.00021
328099	ILMN_241519	Mus musculus expressed sequence AU021838 (AU021838), mRNA.	1.03	6.60E-06
27053	Asns	asparagine synthetase	1.03	7.00E-04
57315	Wdr46	WD repeat domain 46	1.02	3.00E-04
28035	Usp39	ubiquitin specific peptidase 39	1.02	0.00015
50995	Uba2	ubiquitin-like modifier activating enzyme 2	1.02	3.00E-05
22143	Tuba1b	tubulin, alpha 1B	1.02	7.30E-05
69071	Tmem97	transmembrane protein 97	1.02	0.00015
20901	Strap	serine/threonine kinase receptor associated protein	1.02	3.10E-05
26570	Slc7a11	solute carrier family 7 (cationic amino acid transporter, y+ system), member 11	1.02	0.024
76560	Prss8	protease, serine, 8 (prostasin)	1.02	0.0013
69241	ILMN_212219	Mus musculus polymerase (RNA) II (DNA directed) polypeptide D (Polr2d), transcript variant 2, mRNA.	1.02	1.40E-05
66249	Pno1	partner of NOB1 homolog (S. cerevisiae)	1.02	0.00011
18769	Pkig	protein kinase inhibitor, gamma	1.02	0.00065
13619	Phc1	polyhomeotic-like 1 (Drosophila)	1.02	0.00011
212503	ILMN_212962	Mus musculus polyamine oxidase (exo-N4-amino) (Paox), mRNA.	1.02	1.10E-05
170762	ILMN_196165	Mus musculus nucleoporin 155 (Nup155), mRNA.	1.02	3.60E-06
241275	Noxa1	NADPH oxidase activator 1	1.02	0.0049
50529	Mrps7	mitchondrial ribosomal protein S7	1.02	9.60E-08
17279	Melk	maternal embryonic leucine zipper kinase	1.02	1.50E-05
73804	Kif2c	kinesin family member 2C	1.02	0.0016
67781	Ilf2	interleukin enhancer binding factor 2	1.02	5.60E-06
15331	ILMN_213625	Mus musculus high mobility group nucleosomal binding domain 2 (Hmgn2), mRNA.	1.02	8.70E-06
51886	Fubp1	far upstream element (FUSE) binding protein 1	1.02	7.00E-05
101358	Fbxl14	F-box and leucine-rich repeat protein 14	1.02	1.10E-06
23874	Farsb	phenylalanyl-tRNA synthetase, beta subunit	1.02	8.20E-07
19347	ILMN_213254	Mus musculus DENN/MADD domain containing 5A (Dennd5a), mRNA.	1.02	4.30E-05
76863	Dcun1d5	DCN1, defective in cullin neddylation 1, domain containing 5 (S. cerevisiae)	1.02	2.70E-05
235386	ILMN_259576	Mus musculus RIKEN cDNA C630028N24 gene (C630028N24Rik), mRNA.	1.02	0.0024
100710	Pds5b	PDS5, regulator of cohesion maintenance, homolog B (S. cerevisiae)	1.02	5.40E-07
208624	Alg3	asparagine-linked glycosylation 3 homolog (yeast, alpha-1,3-mannosyltransferase)	1.02	0.00019
67371	ILMN_209771	Mus musculus RIKEN cDNA 2410016F19 gene (2410016F19Rik), mRNA.	1.02	0.00096
22171	Tyms	thymidylate synthase	1.01	9.00E-05
21781	Tfdp1	transcription factor Dp 1	1.01	4.60E-05
217031	Tada2a	transcriptional adaptor 2A	1.01	4.80E-05
170460	Stard5	StAR-related lipid transfer (START) domain containing 5	1.01	0.00026
84505	Setdb1	SET domain, bifurcated 1	1.01	3.80E-05
	ILMN_187520		1.01	0.0013
20103	Rps5	ribosomal protein S5	1.01	0.0024
27176	ILMN_234979	Mus musculus ribosomal protein L7a (Rpl7a), mRNA.	1.01	0.0012
72486	Rnf219	ring finger protein 219	1.01	4.30E-05
56452	Orc6	origin recognition complex, subunit 6	1.01	5.00E-06
68043	N6amt2	N-6 adenine-specific DNA methyltransferase 2 (putative)	1.01	0.00017
68114	Mum1	melanoma associated antigen (mutated) 1	1.01	4.60E-05
546015	ILMN_213995	PREDICTED: Mus musculus similar to ribosomal protein S9 (LOC546015), misc RNA.	1.01	0.00014
27993	Imp4	IMP4, U3 small nucleolar ribonucleoprotein, homolog (yeast)	1.01	1.00E-05
14853	Gspt2	G1 to S phase transition 2	1.01	7.70E-05
66120	Fkbp11	FK506 binding protein 11	1.01	0.0024
276770	Eif5a	eukaryotic translation initiation factor 5A	1.01	3.20E-05
67160	Eef1g	eukaryotic translation elongation factor 1 gamma	1.01	5.70E-07
72831	Dhx30	DEAH (Asp-Glu-Ala-His) box polypeptide 30	1.01	2.30E-05
14793	Cdca3	cell division cycle associated 3	1.01	5.00E-04
71492	Bbs7	Bardet-Biedl syndrome 7 (human)	1.01	2.10E-05
23825	Banf1	barrier to autointegration factor 1	1.01	0.00027
11745	ILMN_221274	Mus musculus annexin A3 (Anxa3), mRNA.	1.01	0.015
381903	Alg8	asparagine-linked glycosylation 8 homolog (yeast, alpha-1,3-glucosyltransferase)	1.01	0.00056
223921	Aaas	achalasia, adrenocortical insufficiency, alacrimia	1.01	0.00027
237880	1700071K01Rik	RIKEN cDNA 1700071K01 gene	1.01	0.00051
240675	Vwa2	von Willebrand factor A domain containing 2	1	0.00015
22247	Umps	uridine monophosphate synthetase	1	0.00042
56085	Ubqln1	ubiquilin 1	1	8.70E-06
22051	Trip6	thyroid hormone receptor interactor 6	1	0.001
21973	Top2a	topoisomerase (DNA) II alpha	1	0.002
19240	ILMN_243396	Mus musculus thymosin, beta 10 (Tmsb10), mRNA.	1	0.00053
66628	Thg1l	tRNA-histidine guanylyltransferase 1-like (S. cerevisiae)	1	0.00022
53416	Stk39	serine/threonine kinase 39, STE20/SPS1 homolog (yeast)	1	2.70E-05
53607	Snrpa	small nuclear ribonucleoprotein polypeptide A	1	1.40E-06
74777	Sepn1	selenoprotein N, 1	1	9.00E-05
54364	Rpp30	ribonuclease P/MRP 30 subunit (human)	1	8.70E-05
19707	Reps1	RalBP1 associated Eps domain containing protein	1	4.10E-06
75292	Prkd3	protein kinase D3	1	1.90E-05
18618	Pemt	phosphatidylethanolamine N-methyltransferase	1	6.60E-05
56488	Nxt1	NTF2-related export protein 1	1	1.30E-05
68611	Mrpl28	mitochondrial ribosomal protein L28	1	3.00E-05
67903	Gipc1	GIPC PDZ domain containing family, member 1	1	0.00029
319710	Frmd6	FERM domain containing 6	1	0.00025
14252	Flot2	flotillin 2	1	9.10E-05
23873	Faim	Fas apoptotic inhibitory molecule	1	0.00091
227715	Exosc2	exosome component 2	1	1.00E-05
227715	Exosc2	exosome component 2	1	0.00027
67728	Dph2	DPH2 homolog (S. cerevisiae)	1	2.70E-07
225995	D030056L22Rik	RIKEN cDNA D030056L22 gene	1	0.00013
108912	Cdca2	cell division cycle associated 2	1	7.30E-05
54219	Cd320	CD320 antigen	1	0.0022
12181	Bop1	block of proliferation 1	1	0.00047
239368	ILMN_209600	Mus musculus cDNA sequence BC030476 (BC030476), mRNA.	1	0.017
208624	Alg3	asparagine-linked glycosylation 3 homolog (yeast, alpha-1,3-mannosyltransferase)	1	6.60E-06
66422	Dctpp1	dCTP pyrophosphatase 1	1	4.00E-04
22320	Vamp8	vesicle-associated membrane protein 8	-1	4.60E-06
67473	Slc47a1	solute carrier family 47, member 1	-1	1.10E-05
81910	Rrbp1	ribosome binding protein 1	-1	0.0037
224938	Pja2	praja 2, RING-H2 motif containing	-1	7.10E-05
52793	Fam3b	family with sequence similarity 3, member B	-1	0.0025
109305	Orai1	ORAI calcium release-activated calcium modulator 1	-1	0.00015
14792	ILMN_210660	Mus musculus lysophosphatidylcholine acyltransferase 3 (Lpcat3), mRNA.	-1	0.00025
381924	Itgad	integrin, alpha D	-1	0.006
14674	Gna13	guanine nucleotide binding protein, alpha 13	-1	0.0045
74256	Cyld	cylindromatosis (turban tumor syndrome)	-1	0.00086
54151	Cyhr1	cysteine and histidine rich 1	-1	2.70E-05
12653	Chgb	chromogranin B	-1	8.70E-05
218294	Cdc14b	CDC14 cell division cycle 14 homolog B (S. cerevisiae)	-1	0.00029
12153	Bmp1	bone morphogenetic protein 1	-1	7.20E-06
12010	B2m	beta-2 microglobulin	-1	0.0021
11370	Acadvl	acyl-Coenzyme A dehydrogenase, very long chain	-1	0.00052
76408	Abcc3	ATP-binding cassette, sub-family C (CFTR/MRP), member 3	-1	0.00021
71774	Shroom1	shroom family member 1	-1	0.0011
22217	Usp12	ubiquitin specific peptidase 12	-1.01	1.90E-05
381058	ILMN_190827	Mus musculus unc-93 homolog A (C. elegans) (Unc93a), mRNA.	-1.01	0.0021
66664	Tmem41a	transmembrane protein 41a	-1.01	9.40E-05
26944	Tinag	tubulointerstitial nephritis antigen	-1.01	0.00017
98267	Stk17b	serine/threonine kinase 17b (apoptosis-inducing)	-1.01	0.0012
20849	Stat4	signal transducer and activator of transcription 4	-1.01	0.0017
67474	Snap29	synaptosomal-associated protein 29	-1.01	7.80E-06
19334	Rab22a	RAB22A, member RAS oncogene family	-1.01	0.00091
18631	Pex11a	peroxisomal biogenesis factor 11 alpha	-1.01	0.0022

17916	Myo1f	myosin IF	-1.01	7.70E-05
16890	Lipe	lipase, hormone sensitive	-1.01	0.00055
16561	Kif1b	kinesin family member 1B	-1.01	1.70E-08
16362	Irf1	interferon regulatory factor 1	-1.01	0.0085
57890	Il17re	interleukin 17 receptor E	-1.01	0.0024
56489	Ikbke	inhibitor of kappaB kinase epsilon	-1.01	2.80E-05
68177	Ebpl	emopamil binding protein-like	-1.01	0.00023
11475	Acta2	actin, alpha 2, smooth muscle, aorta	-1.01	0.0098
381560	Xkr8	X Kell blood group precursor related family member 8 homolog	-1.02	1.00E-05
68667	ILMN_195068	Mus musculus transient receptor potential cation channel, subfamily M, member 4 (Trpm4), mRNA.	-1.02	0.00015
60455	Tmem8	transmembrane protein 8 (five membrane-spanning domains)	-1.02	4.60E-05
209760	Tmc7	transmembrane channel-like gene family 7	-1.02	0.0021
67528	Nudt7	nudix (nucleoside diphosphate linked moiety X)-type motif 7	-1.02	8.20E-06
259277	Klk8	kallikrein related-peptidase 8	-1.02	0.00053
13716	Ell	elongation factor RNA polymerase II	-1.02	2.10E-06
260409	Cdc42ep3	CDC42 effector protein (Rho GTPase binding) 3	-1.02	0.0081
12516	Cd7	CD7 antigen	-1.02	0.0026
73102	Slc22a23	solute carrier family 22, member 23	-1.02	2.50E-06
16912	Psmb9	proteasome (prosome, macropain) subunit, beta type 9 (large multifunctional peptidase 2)	-1.03	0.034
56428	Mtch2	mitochondrial carrier homolog 2 (C. elegans)	-1.03	5.90E-05
16561	Kif1b	kinesin family member 1B	-1.03	9.30E-05
216136	Ilvbl	ilvB (bacterial acetolactate synthase)-like	-1.03	0.00032
66847	Hint3	histidine triad nucleotide binding protein 3	-1.03	6.70E-06
14042	Ext1	exostoses (multiple) 1	-1.03	0.00026
53310	Dlg3	discs, large homolog 3 (Drosophila)	-1.03	1.20E-05
12511	Cd6	CD6 antigen	-1.03	0.00091
11671	ILMN_251976	Mus musculus aldehyde dehydrogenase family 3, subfamily A2 (Aldh3a2), mRNA.	-1.03	2.50E-06
269997	Zfp747	zinc finger protein 747	-1.04	0.00034
22337	Vdr	vitamin D receptor	-1.04	0.00062
70599	Ssfa2	sperm specific antigen 2	-1.04	0.0018
217430	Pqlc3	PQ loop repeat containing	-1.04	9.40E-05
16362	Irf1	interferon regulatory factor 1	-1.04	0.0021
277463	Gpr107	G protein-coupled receptor 107	-1.04	5.70E-07
12359	ILMN_254459	Mus musculus catalase (Cat), mRNA.	-1.04	7.40E-05
26363	Btd	biotinidase	-1.04	1.00E-05
66090	Ypel3	yippee-like 3 (Drosophila)	-1.05	0.00034
233405	Vps33b	vacuolar protein sorting 33B (yeast)	-1.05	0.00013
235135	Tmem45b	transmembrane protein 45b	-1.05	4.70E-06
21787	Tfg	Trk-fused gene	-1.05	6.20E-08
263876	Spata2	spermatogenesis associated 2	-1.05	1.50E-05
107723	Slc12a6	solute carrier family 12, member 6	-1.05	2.10E-05
12258	Serping1	serine (or cysteine) peptidase inhibitor, clade G, member 1	-1.05	0.01
101476	Plekha1	pleckstrin homology domain containing, family A (phosphoinositide binding specific) member 1	-1.05	1.30E-05
18633	Pex16	peroxisomal biogenesis factor 16	-1.05	8.50E-07
23959	Nt5e	5' nucleotidase, ecto	-1.05	0.0081
17130	Smad6	MAD homolog 6 (Drosophila)	-1.05	1.60E-05
114255	Dok4	docking protein 4	-1.05	0.014
109672	Cyb5	cytochrome b-5	-1.05	7.10E-05
68631	Cryl1	crystallin, lambda 1	-1.05	3.80E-07
12575	ILMN_209664	Mus musculus cyclin-dependent kinase inhibitor 1A (P21) (Cdkn1a), mRNA.	-1.05	0.00034
74018	Als2	amyotrophic lateral sclerosis 2 (juvenile) homolog (human)	-1.05	9.70E-06
69596	2310035K24Rik	RIKEN cDNA 2310035K24 gene	-1.05	6.00E-05
22376	Was	Wiskott-Aldrich syndrome homolog (human)	-1.06	6.40E-05
227659	Slc2a6	solute carrier family 2 (facilitated glucose transporter), member 6	-1.06	0.0034
215474	Sec22c	SEC22 vesicle trafficking protein homolog C (S. cerevisiae)	-1.06	3.80E-05
56367	Scoc	short coiled-coil protein	-1.06	2.50E-05
78388	Mvp	major vault protein	-1.06	5.30E-06
16452	Jak2	Janus kinase 2	-1.06	0.00013
140579	Elmo2	engulfment and cell motility 2, ced-12 homolog (C. elegans)	-1.06	0.00027
83429	Ctns	cystinosis, nephropathic	-1.06	0.00047
12833	Col6a1	collagen, type VI, alpha 1	-1.06	0.01
213649	Arhgef19	Rho guanine nucleotide exchange factor (GEF) 19	-1.06	0.00011
216991	Adap2	ArfGAP with dual PH domains 2	-1.06	8.70E-06
11461	Actb	actin, beta	-1.06	7.00E-04
11364	Acadm	acyl-Coenzyme A dehydrogenase, medium chain	-1.06	3.70E-07
24001	Tiam2	T-cell lymphoma invasion and metastasis 2	-1.07	7.00E-04
21355	ILMN_217715	Mus musculus transporter 2, ATP-binding cassette, sub-family B (MDR/TAP) (Tap2), mRNA.	-1.07	0.018
70510	Rnf167	ring finger protein 167	-1.07	1.40E-05
110095	Pygl	liver glycogen phosphorylase	-1.07	0.00032
78004	ILMN_214576	Mus musculus proline rich 15 (Prr15), mRNA.	-1.07	0.00075
17940	Naip1	NLR family, apoptosis inhibitory protein 1	-1.07	0.0012
194126	Mtmr11	myotubularin related protein 11	-1.07	7.70E-05
56428	Mtch2	mitochondrial carrier homolog 2 (C. elegans)	-1.07	3.20E-06
266632	ILMN_216529	Mus musculus interleukin-1 receptor-associated kinase 4 (Irak4), mRNA.	-1.07	4.00E-05
12499	Entpd5	ectonucleoside triphosphate diphosphohydrolase 5	-1.07	7.60E-06
243881	Cyp2b23	cytochrome P450, family 2, subfamily b, polypeptide 23	-1.07	0.0026
109754	Cyb5r3	cytochrome b5 reductase 3	-1.07	7.80E-05
12515	Cd69	CD69 antigen	-1.07	0.0013
	ILMN_223067		-1.07	3.80E-06
244882	Tnfaip8l3	tumor necrosis factor, alpha-induced protein 8-like 3	-1.08	0.00012
67043	Syap1	synapse associated protein 1	-1.08	1.70E-05
56632	ILMN_218883	Mus musculus sphingosine kinase 2 (Sphk2), transcript variant 2, mRNA.	-1.08	2.90E-05
20715	Serpina3g	serine (or cysteine) peptidase inhibitor, clade A, member 3G	-1.08	0.0014
72112	Ppp1r14d	protein phosphatase 1, regulatory (inhibitor) subunit 14D	-1.08	1.60E-06
102693	Phldb1	pleckstrin homology-like domain, family B, member 1	-1.08	0.0018
68671	Pcyt2	phosphate cytidylyltransferase 2, ethanolamine	-1.08	8.90E-07
107587	Osr2	odd-skipped related 2 (Drosophila)	-1.08	0.015
18105	Nqo2	NAD(P)H dehydrogenase, quinone 2	-1.08	3.90E-06
269593	Luzp1	leucine zipper protein 1	-1.08	0.00032
15040	ILMN_196750	Mus musculus histocompatibility 2, T region locus 23 (H2-T23), mRNA.	-1.08	0.016
14281	Fos	FBJ osteosarcoma oncogene	-1.08	0.00065
218294	ILMN_251331	Mus musculus CDC14 cell division cycle 14 homolog B (S. cerevisiae) (Cdc14b), mRNA.	-1.08	0.00011
226777	C130074G19Rik	RIKEN cDNA C130074G19 gene	-1.08	0.00013
74442	Sgms2	sphingomyelin synthase 2	-1.09	2.10E-05
19698	Relb	avian reticuloendotheliosis viral (v-rel) oncogene related B	-1.09	6.40E-05
67245	Peli1	pellino 1	-1.09	4.40E-05
360013	Myo18a	myosin XVIIIA	-1.09	0.00032
64898	Lpin2	lipin 2	-1.09	0.00047
214854	Neurl3	neuralized homolog 3 homolog (Drosophila)	-1.09	0.00013
16477	Junb	Jun-B oncogene	-1.09	1.00E-05
77125	Il33	interleukin 33	-1.09	0.003
13175	Dclk1	doublecortin-like kinase 1	-1.09	0.00026
211535	Ccdc114	coiled-coil domain containing 114	-1.09	2.60E-05
223642	Zc3h3	zinc finger CCCH type containing 3	-1.1	3.80E-07
22337	Vdr	vitamin D receptor	-1.1	2.60E-05
72094	Ugt2a3	UDP glucuronosyltransferase 2 family, polypeptide A3	-1.1	1.20E-05
17919	Myo5b	myosin VB	-1.1	0.00081
243374	Gimap8	GTPase, IMAP family member 8	-1.1	0.00081
72972	Gcap14	granule cell antiserum positive 14	-1.1	4.70E-06
231717	ILMN_193500	Mus musculus family with sequence similarity 109, member A (Fam109a), mRNA.	-1.1	0.00057
630499	ILMN_244752	Mus musculus predicted gene, EG630499 (EG630499), mRNA.	-1.1	0.0021
27883	D16H22S680E	DNA segment, Chr 16, human D22S680E, expressed	-1.1	1.20E-07
72747	Ttc39c	tetratricopeptide repeat domain 39C	-1.1	5.40E-05
625286	Fam23a	family with sequence similarity 23, member A	-1.1	2.70E-06
68520	Zfyve21	zinc finger, FYVE domain containing 21	-1.11	5.70E-07
27371	Sh2d2a	SH2 domain protein 2A	-1.11	0.0081
20181	ILMN_221874	Mus musculus retinoid X receptor alpha (Rxra), mRNA.	-1.11	2.00E-04

19268	Ptprf	protein tyrosine phosphatase, receptor type, F	-1.11	3.90E-06
76787	Ppfia3	protein tyrosine phosphatase, receptor type, f polypeptide (PTPRF), interacting protein (liprin), alpha 3	-1.11	0.0013
171580	Mical1	microtubule associated monoxygenase, calponin and LIM domain containing 1	-1.11	0.0039
232431	ILMN_239682	Mus musculus G protein-coupled receptor, family C, group 5, member A (Gprc5a), mRNA.	-1.11	0.011
12499	Entpd5	ectonucleoside triphosphate diphosphohydrolase 5	-1.11	1.90E-06
12833	Col6a1	collagen, type VI, alpha 1	-1.11	0.049
12651	ILMN_212086	Mus musculus choline kinase beta (Chkb), mRNA.	-1.11	1.60E-06
12362	ILMN_211039	Mus musculus caspase 1 (Casp1), mRNA.	-1.11	0.00038
74156	ILMN_220246	Mus musculus acyl-CoA thioesterase 12 (Acot12), mRNA.	-1.11	0.00096
66938	1700029G01Rik	RIKEN cDNA 1700029G01 gene	-1.11	2.00E-04
67226	Tmem19	transmembrane protein 19	-1.12	0.00035
71699	Slc41a3	solute carrier family 41, member 3	-1.12	0.00091
67712	Slc25a37	solute carrier family 25, member 37	-1.12	2.70E-05
224024	Scarf2	scavenger receptor class F, member 2	-1.12	0.00017
67991	Nacc2	nucleus accumbens associated 2, BEN and BTB (POZ) domain containing	-1.12	8.80E-06
70337	Iyd	iodotyrosine deiodinase	-1.12	3.20E-05
70110	Ifi35	interferon-induced protein 35	-1.12	3.20E-05
15488	Hsd17b4	hydroxysteroid (17-beta) dehydrogenase 4	-1.12	1.40E-06
330914	Arhgap32	Rho GTPase activating protein 32	-1.12	0.00017
14600	Ghr	growth hormone receptor	-1.12	0.0016
13047	Cux1	cut-like homeobox 1	-1.12	0.00014
223754	Tbc1d22a	TBC1 domain family, member 22a	-1.13	4.00E-07
22782	Slc30a1	solute carrier family 30 (zinc transporter), member 1	-1.13	0.00032
16889	Lipa	lysosomal acid lipase A	-1.13	7.90E-05
16664	Krt14	keratin 14	-1.13	0.01
71597	Isx	intestine specific homeobox	-1.13	0.00035
109754	Cyb5r3	cytochrome b5 reductase 3	-1.13	1.40E-07
21940	Cd27	CD27 antigen	-1.13	0.00032
66259	Camk2n1	calcium/calmodulin-dependent protein kinase II inhibitor 1	-1.13	1.70E-05
269423	3110057O12Rik	RIKEN cDNA 3110057O12 gene	-1.13	2.10E-05
69064	1810014F10Rik	RIKEN cDNA 1810014F10 gene	-1.13	6.10E-05
227620	Uap1l1	UDP-N-acteylglucosamine pyrophosphorylase 1-like 1	-1.14	5.30E-05
77975	Tmem50b	transmembrane protein 50B	-1.14	0.0011
17063	Muc13	mucin 13, epithelial transmembrane	-1.14	0.004
100045403	ILMN_221330	PREDICTED: Mus musculus similar to orthologue of H. sapiens chromosome 21 open reading frame 102 (C20orf102) (LOC100045403), misc RNA.	-1.14	0.001
74096	Hvcn1	hydrogen voltage-gated channel 1	-1.14	3.00E-04
71908	Cldn23	claudin 23	-1.14	1.50E-05
66433	Chchd7	coiled-coil-helix-coiled-coil-helix domain containing 7	-1.14	0.00024
23827	Bpnt1	bisphosphate 3'-nucleotidase 1	-1.14	2.50E-06
11975	Atp6v0a1	ATPase, H+ transporting, lysosomal V0 subunit A1	-1.14	6.00E-06
71704	Arhgef3	Rho guanine nucleotide exchange factor (GEF) 3	-1.14	5.30E-05
20910	Stxbp1	syntaxin binding protein 1	-1.15	0.00035
70510	Rnf167	ring finger protein 167	-1.15	7.60E-06
71801	Plekhf2	pleckstrin homology domain containing, family F (with FYVE domain) member 2	-1.15	0.0071
18631	Pex11a	peroxisomal biogenesis factor 11 alpha	-1.15	0.00055
641240	ILMN_210638	PREDICTED: Mus musculus similar to MHC class II antigen beta chain (LOC641240), mRNA.	-1.15	0.042
16452	Jak2	Janus kinase 2	-1.15	0.00022
67603	Dusp6	dual specificity phosphatase 6	-1.15	0.0021
27999	Fam3c	family with sequence similarity 3, member C	-1.15	5.70E-07
12511	Cd6	CD6 antigen	-1.15	0.0045
432442	Akap7	A kinase (PRKA) anchor protein 7	-1.15	4.50E-07
109652	Acy1	aminoacylase 1	-1.15	1.60E-05
56791	Ube2l6	ubiquitin-conjugating enzyme E2L 6	-1.16	0.0025
56786	Tmem9b	TMEM9 domain family, member B	-1.16	2.70E-06
76281	ILMN_189706	Mus musculus Tax1 (human T-cell leukemia virus type I) binding protein 3 (Tax1bp3), mRNA.	-1.16	3.20E-06
76650	Srxn1	sulfiredoxin 1 homolog (S. cerevisiae)	-1.16	0.00032
72022	ILMN_235723	Mus musculus solute carrier family 35, member F2 (Slc35f2), mRNA.	-1.16	0.00096
140742	Sesn1	sestrin 1	-1.16	2.70E-05
235442	Rab8b	RAB8B, member RAS oncogene family	-1.16	0.0067
18762	Prkcz	protein kinase C, zeta	-1.16	2.10E-05
19012	Ppap2a	phosphatidic acid phosphatase type 2A	-1.16	0.0041
68671	Pcyt2	phosphate cytidylyltransferase 2, ethanolamine	-1.16	0.00024
18126	Nos2	nitric oxide synthase 2, inducible	-1.16	0.042
16971	Lrp1	low density lipoprotein receptor-related protein 1	-1.16	1.90E-05
239759	Liph	lipase, member H	-1.16	0.001
18048	ILMN_199361	Mus musculus kallikrein 1-related pepidase b4 (Klk1b4), mRNA.	-1.16	8.90E-07
238377	Gpr68	G protein-coupled receptor 68	-1.16	2.70E-06
14571	Gpd2	glycerol phosphate dehydrogenase 2, mitochondrial	-1.16	0.0012
66569	Gdpd1	glycerophosphodiester phosphodiesterase domain containing 1	-1.16	9.00E-08
67064	Chmp1b	chromatin modifying protein 1B	-1.16	8.20E-05
75909	Tmem49	transmembrane protein 49	-1.17	0.0015
20648	Snta1	syntrophin, acidic 1	-1.17	4.40E-05
71699	Slc41a3	solute carrier family 41, member 3	-1.17	0.0014
228875	Slc35c2	solute carrier family 35, member C2	-1.17	6.50E-05
108705	Pttg1ip	pituitary tumor-transforming 1 interacting protein	-1.17	8.30E-06
18770	Pklr	pyruvate kinase liver and red blood cell	-1.17	3.00E-06
56421	Pfkp	phosphofructokinase, platelet	-1.17	6.20E-05
214854	Neurl3	neuralized homolog 3 homolog (Drosophila)	-1.17	3.40E-06
72748	Hdhd3	haloacid dehalogenase-like hydrolase domain containing 3	-1.17	0.00038
12363	ILMN_213344	Mus musculus caspase 4, apoptosis-related cysteine peptidase (Casp4), mRNA.	-1.17	0.017
23827	Bpnt1	bisphosphate 3'-nucleotidase 1	-1.17	0.00035
12010	B2m	beta-2 microglobulin	-1.17	0.0032
71687	Tmem25	transmembrane protein 25	-1.18	0.00011
67043	Syap1	synapse associated protein 1	-1.18	2.80E-05
20732	Spint1	serine protease inhibitor, Kunitz type 1	-1.18	0.00051
18408	Slc25a15	solute carrier family 25 (mitochondrial carrier ornithine transporter), member 15	-1.18	1.90E-06
19092	Prkg2	protein kinase, cGMP-dependent, type II	-1.18	8.70E-05
66522	Pgpep1	pyroglutamyl-peptidase I	-1.18	2.40E-06
269593	Luzp1	leucine zipper protein 1	-1.18	6.00E-07
14792	ILMN_210660	Mus musculus lysophosphatidylcholine acyltransferase 3 (Lpcat3), mRNA.	-1.18	4.90E-05
100048721	ILMN_210479	PREDICTED: Mus musculus similar to fibronectin leucine rich transmembrane protein 3, transcript variant 1 (LOC100048721), mRNA.	-1.18	1.20E-05
16619	Klk1b27	kallikrein 1-related peptidase b27	-1.18	0.00021
16362	Irf1	interferon regulatory factor 1	-1.18	0.00081
27399	Ip6k1	inositol hexaphosphate kinase 1	-1.18	7.10E-05
114664	Hsd17b11	hydroxysteroid (17-beta) dehydrogenase 11	-1.18	0.0014
78088	Ankrd56	ankyrin repeat domain 56	-1.18	2.70E-05
27403	Abca7	ATP-binding cassette, sub-family A (ABC1), member 7	-1.18	1.10E-05
69137	2200002K05Rik	RIKEN cDNA 2200002K05 gene	-1.18	1.80E-05
19331	Rab19	RAB19, member RAS oncogene family	-1.19	6.50E-06
18718	Pip4k2a	phosphatidylinositol-5-phosphate 4-kinase, type II, alpha	-1.19	7.00E-04
26424	Nr5a2	nuclear receptor subfamily 5, group A, member 2	-1.19	8.90E-05
76273	Ndfip2	Nedd4 family interacting protein 2	-1.19	3.90E-05
245049	Myrip	myosin VIIA and Rab interacting protein	-1.19	0.0017
64899	Lpin3	lipin 3	-1.19	3.60E-05
100048313	ILMN_209018	PREDICTED: Mus musculus similar to ABC transporter, transcript variant 1 (LOC100048313), mRNA.	-1.19	1.40E-05
100047214	ILMN_211513	PREDICTED: Mus musculus similar to PTEN induced putative kinase 1 (LOC100047214), mRNA.	-1.19	1.70E-05
26931	Ppp2r5c	protein phosphatase 2, regulatory subunit B (B56), gamma isoform	-1.19	4.30E-05
56055	Gtpbp2	GTP binding protein 2	-1.19	2.10E-05
93692	Glrx	glutaredoxin	-1.19	1.40E-05
13179	Dcn	decorin	-1.19	0.015
12721	Coro1a	coronin, actin binding protein 1A	-1.19	0.0095
75600	Calml4	calmodulin-like 4	-1.19	0.00052
14605	Tsc22d3	TSC22 domain family, member 3	-1.2	0.00012
21807	Tsc22d1	TSC22 domain family, member 1	-1.2	0.00028
252972	Tpcn1	two pore channel 1	-1.2	3.00E-05
108995	ILMN_216821	Mus musculus TBC1 domain family, member 10c (Tbc1d10c), mRNA.	-1.2	7.10E-05
100434	ILMN_219323	Mus musculus solute carrier family 44, member 1 (Slc44a1), mRNA.	-1.2	1.30E-05

20520	Slc22a5	solute carrier family 22 (organic cation transporter), member 5	-1.2	1.60E-06
98732	Rab3gap2	RAB3 GTPase activating protein subunit 2	-1.2	2.70E-07
18946	Pnliprp1	pancreatic lipase related protein 1	-1.2	0.03
80859	Nfkbiz	nuclear factor of kappa light polypeptide gene enhancer in B-cells inhibitor, zeta	-1.2	0.015
26390	Mapkbp1	mitogen-activated protein kinase binding protein 1	-1.2	8.10E-06
100044538	ILMN_209739	PREDICTED: Mus musculus similar to immunity-associated nucleotide 4 (LOC100044538), misc RNA.	-1.2	1.50E-05
70101	Cyp4f16	cytochrome P450, family 4, subfamily f, polypeptide 16	-1.2	0.00081
20305	Ccl6	chemokine (C-C motif) ligand 6	-1.2	6.50E-06
54608	Abhd2	abhydrolase domain containing 2	-1.2	0.00026
12780	Abcc2	ATP-binding cassette, sub-family C (CFTR/MRP), member 2	-1.2	0.00058
74131	Sash3	SAM and SH3 domain containing 3	-1.2	0.00044
106759	ILMN_193595	Mus musculus toll-like receptor adaptor molecule 1 (Ticam1), mRNA.	-1.21	1.20E-07
72065	Rap2c	RAP2C, member of RAS oncogene family	-1.21	0.00051
16913	Psmb8	proteasome (prosome, macropain) subunit, beta type 8 (large multifunctional peptidase 7)	-1.21	0.00058
226971	Plekhb2	pleckstrin homology domain containing, family B (evectins) member 2	-1.21	4.90E-06
23971	Papss1	3'-phosphoadenosine 5'-phosphosulfate synthase 1	-1.21	4.00E-07
21428	Mlx	MAX-like protein X	-1.21	2.90E-05
66447	Mgst3	microsomal glutathione S-transferase 3	-1.21	6.90E-06
16400	Itga3	integrin alpha 3	-1.21	1.50E-06
54123	Irf7	interferon regulatory factor 7	-1.21	0.023
320541	Slc35e2	solute carrier family 35, member E2	-1.21	0.00017
72046	Urgcp	upregulator of cell proliferation	-1.21	1.60E-05
58176	Rhbg	Rhesus blood group-associated B glycoprotein	-1.22	0.00045
72065	ILMN_211579	Mus musculus RAP2C, member of RAS oncogene family (Rap2c), mRNA.	-1.22	3.80E-06
16407	Itgae	integrin alpha E, epithelial-associated	-1.22	2.60E-05
14710	Gngt2	guanine nucleotide binding protein (G protein), gamma transducing activity polypeptide 2	-1.22	1.10E-05
53897	Gal3st1	galactose-3-O-sulfotransferase 1	-1.22	0.00013
98845	Eps8l2	EPS8-like 2	-1.22	1.50E-06
329540	8430427H17Rik	RIKEN cDNA 8430427H17 gene	-1.22	1.80E-06
56434	Tspan3	tetraspanin 3	-1.23	5.90E-05
56786	Tmem9b	TMEM9 domain family, member B	-1.23	1.20E-05
170459	Stard4	StAR-related lipid transfer (START) domain containing 4	-1.23	0.0041
102141	Snx25	sorting nexin 25	-1.23	4.40E-06
228765	Sdcbp2	syndecan binding protein (syntenin) 2	-1.23	6.00E-06
72333	Palld	palladin, cytoskeletal associated protein	-1.23	0.00015
66447	Mgst3	microsomal glutathione S-transferase 3	-1.23	0.00021
68180	Hyi	hydroxypyruvate isomerase homolog (E. coli)	-1.23	0.0018
12363	ILMN_213344	Mus musculus caspase 4, apoptosis-related cysteine peptidase (Casp4), mRNA.	-1.23	0.0091
171207	ILMN_216990	Mus musculus Rho GTPase activating protein 4 (Arhgap4), mRNA.	-1.23	2.00E-05
71874	2310007B03Rik	RIKEN cDNA 2310007B03 gene	-1.23	6.20E-05
272589	Tbcel	tubulin folding cofactor E-like	-1.24	0.00013
20491	Sla	src-like adaptor	-1.24	1.00E-04
19341	Rab4a	RAB4A, member RAS oncogene family	-1.24	8.40E-06
15468	Prmt2	protein arginine N-methyltransferase 2	-1.24	1.30E-05
66734	Map1lc3a	microtubule-associated protein 1 light chain 3 alpha	-1.24	4.70E-05
17160	Man2b2	mannosidase 2, alpha B2	-1.24	4.20E-06
54120	Gipc2	GIPC PDZ domain containing family, member 2	-1.24	4.30E-05
14464	Gata5	GATA binding protein 5	-1.24	1.90E-06
16800	Arhgef2	rho/rac guanine nucleotide exchange factor (GEF) 2	-1.24	2.50E-06
94284	ILMN_184466	Mus musculus UDP glucuronosyltransferase 1 family, polypeptide A6A (Ugt1a6a), mRNA.	-1.25	4.30E-05
214158	Trim38	tripartite motif-containing 38	-1.25	8.40E-06
66847	Hint3	histidine triad nucleotide binding protein 3	-1.25	3.80E-07
13835	Epha1	Eph receptor A1	-1.25	3.20E-05
13518	Dst	dystonin	-1.25	0.00043
70101	ILMN_220247	Mus musculus cytochrome P450, family 4, subfamily f, polypeptide 16 (Cyp4f16), mRNA.	-1.25	0.00038
269336	Ccdc32	coiled-coil domain containing 32	-1.25	1.30E-07
110075	Bmp3	bone morphogenetic protein 3	-1.25	0.0081
67809	Fam82a2	family with sequence similarity 82, member A2	-1.25	1.40E-06
16969	Zbtb7a	zinc finger and BTB domain containing 7a	-1.26	0.012
240832	Tor1aip2	torsin A interacting protein 2	-1.26	5.30E-06
21356	ILMN_244571	Mus musculus TAP binding protein (Tapbp), transcript variant 1, mRNA.	-1.26	0.00029
228765	Sdcbp2	syndecan binding protein (syntenin) 2	-1.26	1.30E-06
18762	Prkcz	protein kinase C, zeta	-1.26	5.10E-07
77827	ILMN_253546	Mus musculus KRAB-A domain containing 1 (Krba1), mRNA.	-1.26	1.50E-05
16428	Itk	IL2-inducible T-cell kinase	-1.26	1.70E-05
14062	F2r	coagulation factor II (thrombin) receptor	-1.26	0.00042
67460	Decr1	2,4-dienoyl CoA reductase 1, mitochondrial	-1.26	0.00013
67484	Eepd1	endonuclease/exonuclease/phosphatase family domain containing 1	-1.26	4.20E-05
21888	Tle4	transducin-like enhancer of split 4, homolog of Drosophila E(spl)	-1.27	5.10E-07
22644	Rnf103	ring finger protein 103	-1.27	8.80E-09
72065	ILMN_211579	Mus musculus RAP2C, member of RAS oncogene family (Rap2c), mRNA.	-1.27	8.40E-06
102693	Phldb1	pleckstrin homology-like domain, family B, member 1	-1.27	9.30E-05
217214	Nags	N-acetylglutamate synthase	-1.27	3.00E-07
171580	Mical1	microtubule associated monoxygenase, calponin and LIM domain containing 1	-1.27	0.00061
216136	Ilvbl	ilvB (bacterial acetolactate synthase)-like	-1.27	5.90E-08
15248	Hic1	hypermethylated in cancer 1	-1.27	3.60E-06
13197	Gadd45a	growth arrest and DNA-damage-inducible 45 alpha	-1.27	0.00034
114601	Ehbp1l1	EH domain binding protein 1-like 1	-1.27	6.50E-06
94219	ILMN_219676	Mus musculus cyclin M2 (Cnnm2), mRNA.	-1.27	2.00E-07
213056	Fam126b	family with sequence similarity 126, member B	-1.27	0.00015
11772	Ap2a2	adaptor protein complex AP-2, alpha 2 subunit	-1.27	2.70E-07
106759	Ticam1	toll-like receptor adaptor molecule 1	-1.28	0.00011
14526	Gcg	glucagon	-1.28	6.10E-05
218865	Chdh	choline dehydrogenase	-1.28	1.80E-05
234564	Ces1f	carboxylesterase 1F	-1.28	0.00075
19885	Rorc	RAR-related orphan receptor gamma	-1.29	0.0042
66901	ILMN_193507	Mus musculus protein Z, vitamin K-dependent plasma glycoprotein (Proz), mRNA.	-1.29	3.30E-08
23971	Papss1	3'-phosphoadenosine 5'-phosphosulfate synthase 1	-1.29	7.60E-07
65113	ILMN_238643	Mus musculus Nedd4 family interacting protein 1 (Ndfip1), mRNA.	-1.29	5.20E-05
211798	Mfsd9	major facilitator superfamily domain containing 9	-1.29	0.00017
26379	Esrra	estrogen related receptor, alpha	-1.29	2.00E-07
98845	Eps8l2	EPS8-like 2	-1.29	1.10E-07
110524	Dgkq	diacylglycerol kinase, theta	-1.29	7.10E-05
320661	D5Ertd579e	DNA segment, Chr 5, ERATO Doi 579, expressed	-1.29	5.10E-07
17684	Cited2	Cbp/p300-interacting transactivator, with Glu/Asp-rich carboxy-terminal domain, 2	-1.29	1.50E-05
12700	Cish	cytokine inducible SH2-containing protein	-1.29	2.30E-05
106952	Arap3	ArfGAP with RhoGAP domain, ankyrin repeat and PH domain 3	-1.29	0.00075
66548	Adamtsl5	ADAMTS-like 5	-1.29	2.20E-05
67776	ILMN_211556	Mus musculus von Willebrand factor A domain containing 5A (Vwa5a), mRNA.	-1.3	0.00013
26944	Tinag	tubulointerstitial nephritis antigen	-1.3	5.30E-06
18770	Pklr	pyruvate kinase liver and red blood cell	-1.3	1.40E-06
66904	ILMN_212103	Mus musculus propionyl Coenzyme A carboxylase, beta polypeptide (Pccb), mRNA.	-1.3	1.70E-06
18261	ILMN_212136	Mus musculus oncomodulin (Ocm), mRNA.	-1.3	7.50E-06
56217	Mpp5	membrane protein, palmitoylated 5 (MAGUK p55 subfamily member 5)	-1.3	0.00027
243197	Mfsd7a	major facilitator superfamily domain containing 7A	-1.3	3.00E-04
66905	ILMN_214383	Mus musculus mannose-6-phosphate receptor binding protein 1 (M6prbp1), mRNA.	-1.3	9.40E-05
216874	Camta2	calmodulin binding transcription activator 2	-1.3	5.30E-06
100317	ILMN_220412	Mus musculus expressed sequence AU040320 (AU040320), transcript variant 3, mRNA.	-1.3	6.20E-08
225997	Trpm6	transient receptor potential cation channel, subfamily M, member 6	-1.31	0.00017
69769	Tnfaip8l2	tumor necrosis factor, alpha-induced protein 8-like 2	-1.31	1.70E-06
52187	Rragd	Ras-related GTP binding D	-1.31	2.70E-06
16622	Klk1b5	kallikrein 1-related peptidase b5	-1.31	4.10E-06
16529	Kcnk5	potassium channel, subfamily K, member 5	-1.31	1.60E-05
108960	ILMN_217595	Mus musculus interleukin-1 receptor-associated kinase 2 (Irak2), mRNA.	-1.31	0.00016
15018	ILMN_196751	Mus musculus histocompatibility 2, Q region locus 7 (H2-Q7), mRNA.	-1.31	0.00015
12526	Cd8b1	CD8 antigen, beta chain 1	-1.31	0.0071
192653	Ttc36	tetratricopeptide repeat domain 36	-1.32	8.30E-06

215335	Slc36a1	solute carrier family 36 (proton/amino acid symporter), member 1	-1.32	0.00029
20526	Slc2a2	solute carrier family 2 (facilitated glucose transporter), member 2	-1.32	3.90E-05
20254	Scg2	secretogranin II	-1.32	6.00E-07
18703	Pigr	polymeric immunoglobulin receptor	-1.32	0.00061
66522	ILMN_209658	Mus musculus pyroglutamyl-peptidase I (Pgpep1), mRNA.	-1.32	4.20E-06
16612	Klk1	kallikrein 1	-1.32	0.0022
228550	Itpka	inositol 1,4,5-trisphosphate 3-kinase A	-1.32	2.40E-05
16432	Itm2b	integral membrane protein 2B	-1.32	0.00033
235582	ILMN_209622	Mus musculus glycerate kinase (Glyctk), transcript variant 1, mRNA.	-1.32	3.40E-07
13660	Ehd1	EH-domain containing 1	-1.32	2.80E-06
21871	Atp6v0a2	ATPase, H+ transporting, lysosomal V0 subunit A2	-1.32	3.90E-07
11541	ILMN_222330	Mus musculus adenosine A2b receptor (Adora2b), mRNA.	-1.32	5.30E-05
319613	Sybu	syntabulin (syntaxin-interacting)	-1.32	2.80E-05
22337	Vdr	vitamin D receptor	-1.33	3.90E-06
66824	ILMN_223398	Mus musculus PYD and CARD domain containing (Pycard), mRNA.	-1.33	2.50E-05
213171	ILMN_209963	Mus musculus protease, serine 27 (Prss27), mRNA.	-1.33	0.00017
54683	Prdx5	peroxiredoxin 5	-1.33	5.30E-05
72175	Mfsd8	major facilitator superfamily domain containing 8	-1.33	9.30E-06
56743	Lat2	linker for activation of T cells family, member 2	-1.33	3.20E-05
54720	Rcan1	regulator of calcineurin 1	-1.33	4.40E-05
23827	ILMN_210031	Mus musculus bisphosphate 3'-nucleotidase 1 (Bpnt1), mRNA.	-1.33	1.10E-05
215723	Mfsd6l	major facilitator superfamily domain containing 6-like	-1.33	3.20E-05
20345	Selplg	selectin, platelet (p-selectin) ligand	-1.34	4.00E-04
19116	Prlr	prolactin receptor	-1.34	3.40E-07
66612	Ormdl3	ORM1-like 3 (S. cerevisiae)	-1.34	1.90E-06
16529	Kcnk5	potassium channel, subfamily K, member 5	-1.34	3.80E-06
16362	Irf1	interferon regulatory factor 1	-1.34	8.90E-05
239853	Gpr128	G protein-coupled receptor 128	-1.34	1.30E-05
78816	ILMN_220865	Mus musculus Gem-interacting protein (Gmip), mRNA.	-1.34	5.50E-06
231931	Gimap6	GTPase, IMAP family member 6	-1.34	0.001
74147	Ehhadh	enoyl-Coenzyme A, hydratase/3-hydroxyacyl Coenzyme A dehydrogenase	-1.34	6.50E-06
13195	Ddc	dopa decarboxylase	-1.34	5.40E-05
192976	ILMN_211693	Mus musculus cDNA sequence BC046404 (BC046404), mRNA.	-1.34	6.70E-06
382000	AY761184	cDNA sequence AY761184	-1.34	0.0053
171282	Acot4	acyl-CoA thioesterase 4	-1.34	9.60E-06
30962	Slc7a9	solute carrier family 7 (cationic amino acid transporter, y+ system), member 9	-1.35	2.50E-05
54683	Prdx5	peroxiredoxin 5	-1.35	8.20E-06
16439	Itpr2	inositol 1,4,5-triphosphate receptor 2	-1.35	2.70E-05
52710	Gpr172b	G protein-coupled receptor 172B	-1.35	8.30E-06
68177	Ebpl	emopamil binding protein-like	-1.35	4.10E-06
12153	Bmp1	bone morphogenetic protein 1	-1.35	1.10E-05
20334	Sec23a	SEC23A (S. cerevisiae)	-1.36	0.00023
171580	Mical1	microtubule associated monoxygenase, calponin and LIM domain containing 1	-1.36	5.20E-06
77827	ILMN_192115	Mus musculus KRAB-A domain containing 1 (Krba1), mRNA.	-1.36	0.00041
27356	Insl6	insulin-like 6	-1.36	4.70E-06
110168	Gpr18	G protein-coupled receptor 18	-1.36	2.70E-05
12721	Coro1a	coronin, actin binding protein 1A	-1.36	1.00E-04
433256	Acsl5	acyl-CoA synthetase long-chain family member 5	-1.36	1.80E-06
80911	Acox3	acyl-Coenzyme A oxidase 3, pristanoyl	-1.36	2.40E-05
106861	Abhd3	abhydrolase domain containing 3	-1.36	0.00017
394430	ILMN_233870	Mus musculus UDP glycosyltransferase 1 family, polypeptide A10 (Ugt1a10), mRNA.	-1.37	4.60E-05
243771	ILMN_188937	Mus musculus poly (ADP-ribose) polymerase family, member 12 (Parp12), mRNA.	-1.37	0.00014
226144	Erlin1	ER lipid raft associated 1	-1.37	5.50E-06
71934	Car13	carbonic anhydrase 13	-1.37	4.20E-06
11832	Aqp7	aquaporin 7	-1.37	0.0013
60455	Tmem8	transmembrane protein 8 (five membrane-spanning domains)	-1.38	2.20E-06
16792	Laptm5	lysosomal-associated protein transmembrane 5	-1.38	0.0023
16164	Il13ra1	interleukin 13 receptor, alpha 1	-1.38	7.30E-06
14302	Frk	fyn-related kinase	-1.38	0.0031
71898	Apol9b	apolipoprotein L 9b	-1.38	0.0095
19299	Abcd3	ATP-binding cassette, sub-family D (ALD), member 3	-1.38	1.50E-06
50778	Rgs1	regulator of G-protein signaling 1	-1.39	2.00E-06
15468	Prmt2	protein arginine N-methyltransferase 2	-1.39	4.40E-06
18753	Prkcd	protein kinase C, delta	-1.39	4.30E-08
211666	Mgst2	microsomal glutathione S-transferase 2	-1.39	5.10E-07
23849	Klf6	Kruppel-like factor 6	-1.39	0.01
30942	Hnf4g	hepatocyte nuclear factor 4, gamma	-1.39	2.50E-06
15267	Hist2h2aa1	histone cluster 2, H2aa1	-1.39	4.60E-05
13198	Ddit3	DNA-damage inducible transcript 3	-1.39	2.20E-07
13191	Dctn1	dynactin 1	-1.39	2.40E-05
22349	Vil1	villin 1	-1.4	3.90E-07
100727	Ugt2b34	UDP glucuronosyltransferase 2 family, polypeptide B34	-1.4	0.00086
380712	Tlcd2	TLC domain containing 2	-1.4	1.50E-05
11853	Rhoc	ras homolog gene family, member C	-1.4	2.50E-05
329416	Nostrin	nitric oxide synthase trafficker	-1.4	5.90E-05
21386	Tbx3	T-box 3	-1.4	0.00017
74424	Tmc5	transmembrane channel-like gene family 5	-1.41	2.40E-05
20482	Skil	SKI-like	-1.41	0.00036
20719	Serpinb6a	serine (or cysteine) peptidase inhibitor, clade B, member 6a	-1.41	1.70E-07
20351	Sema4a	sema domain, immunoglobulin domain (Ig), transmembrane domain (TM) and short cytoplasmic domain, (semaphorin) 4A	-1.41	1.10E-06
545902	Ptprh	protein tyrosine phosphatase, receptor type, H	-1.41	5.10E-05
29817	Igfbp7	insulin-like growth factor binding protein 7	-1.41	0.0078
66610	Abi3	ABI gene family, member 3	-1.41	0.00032
217203	Tmem106a	transmembrane protein 106A	-1.42	9.80E-07
213171	ILMN_209963	Mus musculus protease, serine 27 (Prss27), mRNA.	-1.42	0.0015
18293	Ogdh	oxoglutarate dehydrogenase (lipoamide)	-1.42	8.50E-07
71207	ILMN_212961	Mus musculus nudix (nucleoside diphosphate linked moiety X)-type motif 4 (Nudt4), mRNA.	-1.42	0.00015
104943	Fam110c	family with sequence similarity 110, member C	-1.42	9.20E-06
109791	Clps	colipase, pancreatic	-1.42	0.0024
11828	Aqp3	aquaporin 3	-1.42	1.40E-07
223920	Soat2	sterol O-acyltransferase 2	-1.43	2.80E-06
20520	Slc22a5	solute carrier family 22 (organic cation transporter), member 5	-1.43	4.90E-07
23954	Nek3	NIMA (never in mitosis gene a)-related expressed kinase 3	-1.43	4.70E-06
56541	Habp4	hyaluronic acid binding protein 4	-1.43	1.90E-05
69068	1810011O10Rik	RIKEN cDNA 1810011O10 gene	-1.43	5.00E-04
69769	Tnfaip8l2	tumor necrosis factor, alpha-induced protein 8-like 2	-1.44	8.20E-09
229731	Slc25a24	solute carrier family 25 (mitochondrial carrier, phosphate carrier), member 24	-1.44	1.20E-05
213019	Pdlim2	PDZ and LIM domain 2	-1.44	0.0012
68671	Pcyt2	phosphate cytidylyltransferase 2, ethanolamine	-1.44	0.00013
269423	ILMN_213311	Mus musculus RIKEN cDNA 3110057O12 gene (3110057O12Rik), mRNA.	-1.44	1.60E-06
67473	Slc47a1	solute carrier family 47, member 1	-1.44	2.40E-06
215113	Slc43a2	solute carrier family 43, member 2	-1.45	1.60E-06
72040	Cdhr5	cadherin-related family member 5	-1.45	2.20E-06
382045	ILMN_236509	Mus musculus G protein-coupled receptor 114 (Gpr114), mRNA.	-1.45	0.00022
238011	Enpp7	ectonucleotide pyrophosphatase/phosphodiesterase 7	-1.45	9.20E-07
12557	Cdh17	cadherin 17	-1.45	8.50E-05
231821	Adap1	ArfGAP with dual PH domains 1	-1.45	1.10E-05
170750	Xpnpep1	X-prolyl aminopeptidase (aminopeptidase P) 1, soluble	-1.46	1.00E-04
18408	Slc25a15	solute carrier family 25 (mitochondrial carrier ornithine transporter), member 15	-1.46	5.50E-06
76608	ILMN_213485	Mus musculus HECT domain containing 3 (Hectd3), mRNA.	-1.46	2.30E-07
22637	Zap70	zeta-chain (TCR) associated protein kinase	-1.47	0.00028
21838	Thy1	thymus cell antigen 1, theta	-1.47	3.60E-05
230612	Slc5a9	solute carrier family 5 (sodium/glucose cotransporter), member 9	-1.47	9.10E-06
70129	Slc44a4	solute carrier family 44, member 4	-1.47	4.70E-06
19419	Rasgrp1	RAS guanyl releasing protein 1	-1.47	0.00013
69693	Pof1b	premature ovarian failure 1B	-1.47	5.40E-07
114664	Hsd17b11	hydroxysteroid (17-beta) dehydrogenase 11	-1.47	2.30E-05

104158	Ces1d	carboxylesterase 1D	-1.47	0.0016
74646	Spsb1	splA/ryanodine receptor domain and SOCS box containing 1	-1.48	4.00E-05
19329	ILMN_215232	Mus musculus RAB17, member RAS oncogene family (Rab17), mRNA.	-1.48	1.70E-08
106581	Itfg3	integrin alpha FG-GAP repeat containing 3	-1.48	1.60E-07
246694	ILMN_246729	Mus musculus Hermansky-Pudlak syndrome 5 homolog (human) (Hps5), transcript variant 2, mRNA.	-1.48	3.10E-05
63986	ILMN_217353	Mus musculus glia maturation factor, gamma (Gmfg), transcript variant 1, mRNA.	-1.48	6.00E-06
112407	Egln3	EGL nine homolog 3 (C. elegans)	-1.48	0.00015
12721	Coro1a	coronin, actin binding protein 1A	-1.48	0.0024
12716	Ckmt1	creatine kinase, mitochondrial 1, ubiquitous	-1.48	1.20E-07
74340	Ahcyl2	S-adenosylhomocysteine hydrolase-like 2	-1.48	2.20E-05
22234	Ugcg	UDP-glucose ceramide glucosyltransferase	-1.49	5.70E-06
71660	Rarres2	retinoic acid receptor responder (tazarotene induced) 2	-1.49	0.0095
18810	Plec	plectin	-1.49	3.00E-06
12355	Nr1i3	nuclear receptor subfamily 1, group I, member 3	-1.49	1.70E-05
52585	Dhrs1	dehydrogenase/reductase (SDR family) member 1	-1.49	4.00E-07
69274	Ctdspl	CTD (carboxy-terminal domain, RNA polymerase II, polypeptide A) small phosphatase-like	-1.49	5.40E-07
12908	Crat	carnitine acetyltransferase	-1.49	3.50E-08
71670	Acy3	aspartoacylase (aminoacylase) 3	-1.49	2.00E-06
72747	Ttc39c	tetratricopeptide repeat domain 39C	-1.49	3.20E-06
21401	Tcea3	transcription elongation factor A (SII), 3	-1.5	5.50E-06
69159	Rhebl1	Ras homolog enriched in brain like 1	-1.5	4.40E-06
17309	Mgat3	mannoside acetylglucosaminyltransferase 3	-1.5	1.10E-06
68338	Golt1a	golgi transport 1 homolog A (S. cerevisiae)	-1.5	5.40E-05
14199	Fhl1	four and a half LIM domains 1	-1.5	0.012
68778	1110038D17Rik	RIKEN cDNA 1110038D17 gene	-1.5	8.80E-09
22268	Upk1b	uroplakin 1B	-1.51	2.10E-05
235442	Rab8b	RAB8B, member RAS oncogene family	-1.51	9.50E-06
74137	Nuak2	NUAK family, SNF1-like kinase, 2	-1.51	9.20E-07
23900	Hcst	hematopoietic cell signal transducer	-1.51	3.90E-09
16985	Lsp1	lymphocyte specific 1	-1.51	4.50E-05
117591	Slc2a9	solute carrier family 2 (facilitated glucose transporter), member 9	-1.52	6.00E-07
104601	Mycbpap	MYCBP associated protein	-1.52	5.20E-06
17454	Mov10	Moloney leukemia virus 10	-1.52	0.00037
14367	Fzd5	frizzled homolog 5 (Drosophila)	-1.52	1.50E-06
72056	1810055G02Rik	RIKEN cDNA 1810055G02 gene	-1.52	4.60E-07
13430	Dnm2	dynamin 2	-1.53	9.80E-07
71904	Paqr7	progestin and adipoQ receptor family member VII	-1.53	2.70E-05
17961	Nat2	N-acetyltransferase 2 (arylamine N-acetyltransferase)	-1.53	5.70E-06
100044204	ILMN_221943	PREDICTED: Mus musculus hypothetical protein LOC100044204 (LOC100044204), mRNA.	-1.54	0.00026
109791	Clps	colipase, pancreatic	-1.54	0.0038
12500	Cd3d	CD3 antigen, delta polypeptide	-1.54	0.00096
12452	Ccng2	cyclin G2	-1.54	0.00034
73833	ILMN_214799	Mus musculus RIKEN cDNA 1110006G06 gene (1110006G06Rik), mRNA.	-1.54	1.10E-06
384071	Slc25a34	solute carrier family 25, member 34	-1.55	2.00E-06
67815	Sec14l2	SEC14-like 2 (S. cerevisiae)	-1.55	4.20E-07
19645	Rb1	retinoblastoma 1	-1.55	7.30E-05
20186	Nr1h4	nuclear receptor subfamily 1, group H, member 4	-1.55	1.90E-06
338403	Cndp1	carnosine dipeptidase 1 (metallopeptidase M20 family)	-1.55	0.00016
11883	Arsa	arylsulfatase A	-1.55	3.80E-06
73910	Arhgap18	Rho GTPase activating protein 18	-1.55	1.60E-05
11810	Apobec1	apolipoprotein B mRNA editing enzyme, catalytic polypeptide 1	-1.55	2.40E-05
384783	Irs2	insulin receptor substrate 2	-1.56	0.006
108960	Irak2	interleukin-1 receptor-associated kinase 2	-1.56	1.50E-06
16176	Il1b	interleukin 1 beta	-1.56	0.039
14990	ILMN_226026	Mus musculus histocompatibility 2, M region locus 2 (H2-M2), mRNA.	-1.56	9.10E-06
18301	Fxyd5	FXYD domain-containing ion transport regulator 5	-1.56	0.00032
13821	Epb4.1l1	erythrocyte protein band 4.1-like 1	-1.56	6.90E-07
13216	ILMN_196581	Mus musculus defensin, alpha 1 (Defa1), mRNA.	-1.56	2.30E-05
72088	Ush1c	Usher syndrome 1C homolog (human)	-1.57	3.20E-08
27219	Sgk2	serum/glucocorticoid regulated kinase 2	-1.57	0.00075
217837	ILMN_220572	Mus musculus inositol 1,3,4-triphosphate 5/6 kinase (Itpk1), mRNA.	-1.57	3.00E-06
16147	Ihh	Indian hedgehog	-1.57	8.10E-05
69824	Glod5	glyoxalase domain containing 5	-1.57	5.10E-07
66298	Defa21	defensin, alpha, 21	-1.57	0.0019
12652	Chga	chromogranin A	-1.57	1.10E-05
74018	ILMN_211844	Mus musculus amyotrophic lateral sclerosis 2 (juvenile) homolog (human) (Als2), mRNA.	-1.57	7.60E-06
67689	Aldh3b1	aldehyde dehydrogenase 3 family, member B1	-1.57	8.90E-07
14972	ILMN_251122	Mus musculus histocompatibility 2, K1, K region (H2-K1), mRNA.	-1.58	2.10E-05
63986	ILMN_217353	Mus musculus glia maturation factor, gamma (Gmfg), transcript variant 1, mRNA.	-1.58	1.10E-06
74596	Cds1	CDP-diacylglycerol synthase 1	-1.58	1.10E-08
12585	Cdr2	cerebellar degeneration-related 2	-1.58	3.90E-09
66395	Ahnak	AHNAK nucleoprotein (desmoyokin)	-1.58	5.70E-06
77577	Spns3	spinster homolog 3 (Drosophila)	-1.58	1.60E-06
328365	Zmiz1	zinc finger, MIZ-type containing 1	-1.59	0.00013
76650	Srxn1	sulfiredoxin 1 homolog (S. cerevisiae)	-1.59	3.40E-05
13131	Dab1	disabled homolog 1 (Drosophila)	-1.59	1.80E-06
11857	Arhgdib	Rho, GDP dissociation inhibitor (GDI) beta	-1.59	0.00026
215445	Rab11fip3	RAB11 family interacting protein 3 (class II)	-1.6	3.20E-06
216136	Ilvbl	ilvB (bacterial acetolactate synthase)-like	-1.6	1.00E-07
78908	Igsf3	immunoglobulin superfamily, member 3	-1.6	2.40E-06
74018	Als2	amyotrophic lateral sclerosis 2 (juvenile) homolog (human)	-1.6	2.60E-05
17921	Myo7a	myosin VIIA	-1.61	8.20E-07
11832	ILMN_210666	Mus musculus aquaporin 7 (Aqp7), mRNA.	-1.61	6.60E-08
107747	ILMN_219033	Mus musculus aldehyde dehydrogenase 1 family, member L1 (Aldh1l1), mRNA.	-1.61	9.80E-07
225997	Trpm6	transient receptor potential cation channel, subfamily M, member 6	-1.62	2.50E-05
103142	ILMN_220451	Mus musculus retinol dehydrogenase 9 (Rdh9), mRNA.	-1.62	7.00E-04
23954	Nek3	NIMA (never in mitosis gene a)-related expressed kinase 3	-1.62	4.60E-08
16995	Ltb4r1	leukotriene B4 receptor 1	-1.62	8.90E-07
113868	Acaa1a	acetyl-Coenzyme A acyltransferase 1A	-1.62	3.30E-08
53906	Phgr1	proline/histidine/glycine-rich 1	-1.62	8.90E-07
23912	Rhof	ras homolog gene family, member f	-1.63	1.40E-06
17119	Mxd1	MAX dimerization protein 1	-1.63	0.00017
17434	Mocs2	molybdenum cofactor synthesis 2	-1.63	6.60E-10
53608	Map3k6	mitogen-activated protein kinase kinase kinase 6	-1.63	6.60E-06
100044177	ILMN_185055	PREDICTED: Mus musculus hypothetical protein LOC100044177 (LOC100044177), mRNA.	-1.63	7.50E-06
16407	Itgae	integrin alpha E, epithelial-associated	-1.63	6.80E-05
207819	ILMN_212940	Mus musculus RIKEN cDNA 4930539E08 gene (4930539E08Rik), mRNA.	-1.63	2.20E-05
19645	Rb1	retinoblastoma 1	-1.64	1.80E-06
107029	Me2	malic enzyme 2, NAD(+)-dependent, mitochondrial	-1.64	6.20E-08
14538	Gcnt2	glucosaminyl (N-acetyl) transferase 2, I-branching enzyme	-1.64	5.20E-06
13823	Epb4.1l3	erythrocyte protein band 4.1-like 3	-1.64	6.90E-08
67512	Agpat2	1-acylglycerol-3-phosphate O-acyltransferase 2 (lysophosphatidic acid acyltransferase, beta)	-1.64	1.10E-06
170750	Xpnpep1	X-prolyl aminopeptidase (aminopeptidase P) 1, soluble	-1.65	8.70E-05
66601	Tmigd1	transmembrane and immunoglobulin domain containing 1	-1.65	2.00E-04
71436	ILMN_210479	Mus musculus fibronectin leucine rich transmembrane protein 3 (Flrt3), mRNA.	-1.65	1.10E-06
12962	Crybb3	crystallin, beta B3	-1.65	1.50E-06
12808	Cobl	cordon-bleu	-1.65	1.70E-05
12163	Bmp8a	bone morphogenetic protein 8a	-1.65	1.10E-05
74153	Uba7	ubiquitin-like modifier activating enzyme 7	-1.66	0.00018
72002	Slc39a5	solute carrier family 39 (metal ion transporter), member 5	-1.66	7.10E-05
64381	Ms4a8a	membrane-spanning 4-domains, subfamily A, member 8A	-1.66	7.80E-08
16421	Itgb7	integrin beta 7	-1.66	2.40E-05
69718	Ipmk	inositol polyphosphate multikinase	-1.66	1.40E-08
171095	Il17rc	interleukin 17 receptor C	-1.66	1.30E-05
12652	Chga	chromogranin A	-1.67	2.90E-07
12369	ILMN_214491	Mus musculus caspase 7 (Casp7), mRNA.	-1.67	3.00E-07
76650	Srxn1	sulfiredoxin 1 homolog (S. cerevisiae)	-1.68	1.10E-07

72472	Slc16a10	solute carrier family 16 (monocarboxylic acid transporters), member 10	-1.68	3.70E-07
16600	Klf4	Kruppel-like factor 4 (gut)	-1.68	8.00E-05
69718	Ipmk	inositol polyphosphate multikinase	-1.68	6.90E-08
107684	Coro2a	coronin, actin binding protein 2A	-1.68	2.10E-09
12014	Bach2	BTB and CNC homology 2	-1.68	4.90E-05
66333	Aqp11	aquaporin 11	-1.68	3.50E-07
53376	Usp2	ubiquitin specific peptidase 2	-1.69	3.00E-06
67405	Nts	neurotensin	-1.69	1.00E-04
15203	Heph	hephaestin	-1.69	6.20E-06
14651	ILMN_221369	Mus musculus hydroxyacyl glutathione hydrolase (Hagh), mRNA.	-1.69	7.50E-09
58991	Ghrl	ghrelin	-1.69	1.20E-06
85308	Fam158a	family with sequence similarity 158, member A	-1.69	1.10E-05
13113	Cyp3a13	cytochrome P450, family 3, subfamily a, polypeptide 13	-1.69	0.00065
12654	Chi3l1	chitinase 3-like 1	-1.69	0.0098
228775	Trib3	tribbles homolog 3 (Drosophila)	-1.7	5.00E-06
106766	Stap2	signal transducing adaptor family member 2	-1.7	2.10E-08
72002	Slc39a5	solute carrier family 39 (metal ion transporter), member 5	-1.7	1.60E-05
76787	Ppfia3	protein tyrosine phosphatase, receptor type, f polypeptide (PTPRF), interacting protein (liprin), alpha 3	-1.7	3.20E-05
50708	Hist1h1c	histone cluster 1, H1c	-1.7	4.90E-06
14916	Guca2b	guanylate cyclase activator 2b (retina)	-1.7	6.50E-06
66168	Grina	glutamate receptor, ionotropic, N-methyl D-aspartate-associated protein 1 (glutamate binding)	-1.7	1.50E-05
170716	Cyp4f13	cytochrome P450, family 4, subfamily f, polypeptide 13	-1.7	1.60E-06
20238	Atxn1	ataxin 1	-1.7	1.50E-06
72002	Slc39a5	solute carrier family 39 (metal ion transporter), member 5	-1.71	4.60E-07
18400	Slc22a18	solute carrier family 22 (organic cation transporter), member 18	-1.71	3.10E-08
20276	Scnn1a	sodium channel, nonvoltage-gated 1 alpha	-1.71	2.10E-07
18810	Plec	plectin	-1.71	3.00E-06
13136	Cd55	CD55 antigen	-1.71	2.20E-05
54215	Cd160	CD160 antigen	-1.71	0.00018
22169	Cmpk2	cytidine monophosphate (UMP-CMP) kinase 2, mitochondrial	-1.72	0.00056
434246	ILMN_252980	Mus musculus tripartite motif-containing 72 (Trim72), mRNA.	-1.72	3.40E-05
74764	Klc4	kinesin light chain 4	-1.72	8.20E-09
14102	Fas	Fas (TNF receptor superfamily member 6)	-1.72	1.70E-05
208659	Fam20a	family with sequence similarity 20, member A	-1.72	1.70E-07
11668	Aldh1a1	aldehyde dehydrogenase family 1, subfamily A1	-1.72	1.80E-05
66395	Ahnak	AHNAK nucleoprotein (desmoyokin)	-1.72	1.30E-05
69864	1810065E05Rik	RIKEN cDNA 1810065E05 gene	-1.72	0.00042
66273	1810020D17Rik	RIKEN cDNA 1810020D17 gene	-1.72	1.00E-06
22695	Zfp36	zinc finger protein 36	-1.73	3.00E-07
22057	Tob1	transducer of ErbB-2.1	-1.73	1.30E-06
380712	Tlcd2	TLC domain containing 2	-1.73	0.00018
72310	Nkg7	natural killer cell group 7 sequence	-1.73	0.0014
171095	Il17rc	interleukin 17 receptor C	-1.73	2.30E-08
67133	Gp2	glycoprotein 2 (zymogen granule membrane)	-1.73	0.0091
170750	Xpnpep1	X-prolyl aminopeptidase (aminopeptidase P) 1, soluble	-1.74	5.10E-07
217214	Nags	N-acetylglutamate synthase	-1.74	1.30E-06
100048721	ILMN_210479	PREDICTED: Mus musculus similar to fibronectin leucine rich transmembrane protein 3, transcript variant 1 (LOC100048721), mRNA.	-1.74	5.50E-06
16407	Itgae	integrin alpha E, epithelial-associated	-1.74	7.80E-05
15212	Hexb	hexosaminidase B	-1.74	8.30E-06
19252	Dusp1	dual specificity phosphatase 1	-1.74	0.00012
74481	Batf2	basic leucine zipper transcription factor, ATF-like 2	-1.74	1.00E-06
215113	Slc43a2	solute carrier family 43, member 2	-1.75	1.40E-06
338365	Slc41a2	solute carrier family 41, member 2	-1.75	2.50E-06
20526	Slc2a2	solute carrier family 2 (facilitated glucose transporter), member 2	-1.75	2.40E-05
280408	Rilp	Rab interacting lysosomal protein	-1.75	1.10E-06
17380	Mme	membrane metallo endopeptidase	-1.75	0.00015
100047937	ILMN_219033	PREDICTED: Mus musculus similar to Aldehyde dehydrogenase 1 family, member L1 (LOC100047937), mRNA.	-1.75	1.70E-07
80909	Gatsl2	GATS protein-like 2	-1.75	1.50E-07
19692	Reg1	regenerating islet-derived 1	-1.76	3.70E-05
14544	Gda	guanine deaminase	-1.76	3.60E-05
99663	Clca6	chloride channel calcium activated 6	-1.76	0.00062
109934	Abr	active BCR-related gene	-1.76	1.80E-06
20303	Ccl4	chemokine (C-C motif) ligand 4	-1.77	0.0013
69698	2310046K01Rik	RIKEN cDNA 2310046K01 gene	-1.77	1.90E-07
29813	Zfp385a	zinc finger protein 385A	-1.78	1.20E-05
27219	Sgk2	serum/glucocorticoid regulated kinase 2	-1.78	0.00035
19267	Ptpre	protein tyrosine phosphatase, receptor type, E	-1.78	1.10E-06
15486	Hsd17b2	hydroxysteroid (17-beta) dehydrogenase 2	-1.78	8.20E-06
14127	Fcer1g	Fc receptor, IgE, high affinity I, gamma polypeptide	-1.78	0.00091
14311	Cidec	cell death-inducing DFFA-like effector c	-1.78	0.00034
11472	Actn2	actinin alpha 2	-1.78	1.50E-06
19193	Pipox	pipecolic acid oxidase	-1.79	2.70E-07
16168	Il15	interleukin 15	-1.79	1.50E-07
235674	Acaa1b	acetyl-Coenzyme A acyltransferase 1B	-1.79	9.00E-08
239393	Lrp12	low density lipoprotein-related protein 12	-1.8	1.00E-06
14469	Gbp2	guanylate binding protein 2	-1.8	0.0078
11732	Ank	progressive ankylosis	-1.8	1.80E-06
319848	Slc17a4	solute carrier family 17 (sodium phosphate), member 4	-1.81	9.60E-06
18604	Pdk2	pyruvate dehydrogenase kinase, isoenzyme 2	-1.81	1.80E-07
23954	Nek3	NIMA (never in mitosis gene a)-related expressed kinase 3	-1.81	7.00E-08
223646	Naprt1	nicotinate phosphoribosyltransferase domain containing 1	-1.81	4.40E-05
171095	Il17rc	interleukin 17 receptor C	-1.81	5.10E-07
104086	Cyp27a1	cytochrome P450, family 27, subfamily a, polypeptide 1	-1.81	1.50E-06
26358	Aldh1a7	aldehyde dehydrogenase family 1, subfamily A7	-1.81	2.30E-05
20280	Scp2	sterol carrier protein 2, liver	-1.82	6.90E-07
217166	Nr1d1	nuclear receptor subfamily 1, group D, member 1	-1.82	6.50E-06
16643	Klrd1	killer cell lectin-like receptor, subfamily D, member 1	-1.82	5.00E-04
231932	Gimap7	GTPase, IMAP family member 7	-1.82	2.00E-05
14199	Fhl1	four and a half LIM domains 1	-1.82	0.0014
13142	Dao	D-amino acid oxidase	-1.82	7.00E-08
28105	Trim36	tripartite motif-containing 36	-1.83	3.90E-07
18489	Reg3b	regenerating islet-derived 3 beta	-1.83	0.016
14672	Gna11	guanine nucleotide binding protein, alpha 11	-1.83	2.20E-07
76051	ILMN_221992	Mus musculus glucosidase, alpha; neutral C (Ganc), mRNA.	-1.83	1.20E-05
13179	Dcn	decorin	-1.83	0.016
13101	Cyp2d10	cytochrome P450, family 2, subfamily d, polypeptide 10	-1.83	1.80E-05
268860	Abat	4-aminobutyrate aminotransferase	-1.83	2.10E-07
13730	Emp1	epithelial membrane protein 1	-1.84	0.00025
12945	Dmbt1	deleted in malignant brain tumors 1	-1.84	0.00022
212070	Clrn3	clarin 3	-1.84	1.60E-06
12724	Clcn2	chloride channel 2	-1.84	1.80E-08
195359	ILMN_235792	Mus musculus tripartite motif-containing 40 (Trim40), mRNA.	-1.85	2.90E-07
68024	Hist1h2bc	histone cluster 1, H2bc	-1.85	4.20E-06
15040	ILMN_196750	Mus musculus histocompatibility 2, T region locus 23 (H2-T23), mRNA.	-1.85	0.00048
13821	Epb4.1l1	erythrocyte protein band 4.1-like 1	-1.85	8.20E-07
12501	Cd3e	CD3 antigen, epsilon polypeptide	-1.85	0.00027
236576	Spry3	sprouty homolog 3 (Drosophila)	-1.86	2.50E-07
209086	Samd9l	sterile alpha motif domain containing 9-like	-1.86	9.00E-05
18799	Plcd1	phospholipase C, delta 1	-1.86	1.00E-06
213019	Pdlim2	PDZ and LIM domain 2	-1.86	0.0015
22259	Nr1h3	nuclear receptor subfamily 1, group H, member 3	-1.86	4.00E-09
13041	Ctsw	cathepsin W	-1.86	6.20E-05
20304	Ccl5	chemokine (C-C motif) ligand 5	-1.86	0.0074
20540	Slc7a7	solute carrier family 7 (cationic amino acid transporter, y+ system), member 7	-1.87	3.80E-05
231147	Sh3tc1	SH3 domain and tetratricopeptide repeats 1	-1.87	2.10E-06
20393	Sgk1	serum/glucocorticoid regulated kinase 1	-1.87	0.00065
19260	Ptpn22	protein tyrosine phosphatase, non-receptor type 22 (lymphoid)	-1.87	6.90E-07

13113	Cyp3a13	cytochrome P450, family 3, subfamily a, polypeptide 13	-1.87	0.00013
65256	Asb2	ankyrin repeat and SOCS box-containing 2	-1.87	2.00E-04
268482	ILMN_217740	Mus musculus keratin 12 (Krt12), mRNA.	-1.88	2.50E-06
100047937	ILMN_219033	PREDICTED: Mus musculus similar to Aldehyde dehydrogenase 1 family, member L1 (LOC100047937), mRNA.	-1.89	1.10E-08
232889	Pla2g4c	phospholipase A2, group IVC (cytosolic, calcium-independent)	-1.9	0.034
227929	Cytip	cytohesin 1 interacting protein	-1.9	1.50E-07
12521	Cd82	CD82 antigen	-1.9	4.70E-09
12424	Cck	cholecystokinin	-1.9	6.70E-06
215210	Tmem120a	transmembrane protein 120A	-1.91	2.10E-09
15446	Hpgd	hydroxyprostaglandin dehydrogenase 15 (NAD)	-1.91	1.40E-05
23833	Cd52	CD52 antigen	-1.91	0.00038
213019	Pdlim2	PDZ and LIM domain 2	-1.92	3.10E-05
99031	Osbpl6	oxysterol binding protein-like 6	-1.92	2.80E-08
13112	Cyp3a11	cytochrome P450, family 3, subfamily a, polypeptide 11	-1.92	0.00028
11803	Aplp1	amyloid beta (A4) precursor-like protein 1	-1.92	8.10E-06
69083	Sult1c2	sulfotransferase family, cytosolic, 1C, member 2	-1.93	8.20E-07
100045343	ILMN_211693	PREDICTED: Mus musculus similar to CDNA sequence BC046404 (LOC100045343), misc RNA.	-1.93	5.10E-07
16168	Il15	interleukin 15	-1.93	1.50E-06
26384	Gnpda1	glucosamine-6-phosphate deaminase 1	-1.93	2.40E-06
11690	Alox5ap	arachidonate 5-lipoxygenase activating protein	-1.93	0.00027
217845	Ifi27l2b	interferon, alpha-inducible protein 27 like 2B	-1.93	0.00081
20397	Sgpl1	sphingosine phosphate lyase 1	-1.94	5.40E-07
20280	Scp2	sterol carrier protein 2, liver	-1.94	1.70E-07
258458	Olfr165	olfactory receptor 165	-1.94	8.20E-07
19279	Ptprr	protein tyrosine phosphatase, receptor type, R	-1.95	1.60E-05
432516	Myo1a	myosin IA	-1.95	1.70E-07
14132	Fcgrt	Fc receptor, IgG, alpha chain transporter	-1.95	1.00E-06
208647	Creb3l2	cAMP responsive element binding protein 3-like 2	-1.95	1.00E-07
15203	Heph	hephaestin	-1.96	4.90E-06
619301	G630016D24Rik	RIKEN cDNA G630016D24 gene	-1.96	1.30E-07
102371	Gcom1	GRINL1A complex locus	-1.96	4.20E-06
69036	Zg16	zymogen granule protein 16	-1.96	1.80E-05
217166	Nr1d1	nuclear receptor subfamily 1, group D, member 1	-1.97	7.00E-05
192976	ILMN_211693	Mus musculus cDNA sequence BC046404 (BC046404), mRNA.	-1.97	1.70E-06
12013	Bach1	BTB and CNC homology 1	-1.97	2.00E-04
83672	ILMN_220695	Mus musculus synaptotagmin-like 3 (Sytl3), transcript variant 1, mRNA.	-1.98	2.20E-08
68659	Fam198b	family with sequence similarity 198, member B	-1.98	2.30E-05
21929	Tnfaip3	tumor necrosis factor, alpha-induced protein 3	-1.99	1.40E-05
11854	Rhod	ras homolog gene family, member D	-1.99	2.30E-06
13809	Enpep	glutamyl aminopeptidase	-1.99	3.20E-06
224796	Clic5	chloride intracellular channel 5	-1.99	5.90E-06
16600	Klf4	Kruppel-like factor 4 (gut)	-2	9.80E-07
14598	Ggt1	gamma-glutamyltransferase 1	-2	9.70E-06
13216	ILMN_196581	Mus musculus defensin, alpha 1 (Defa1), mRNA.	-2	1.60E-05
245038	Dclk3	doublecortin-like kinase 3	-2	1.20E-07
230576	Ttc22	tetratricopeptide repeat domain 22	-2.01	3.10E-07
16407	Itgae	integrin alpha E, epithelial-associated	-2.01	2.00E-04
381175	Ccdc68	coiled-coil domain containing 68	-2.01	1.80E-05
233836	Slc5a11	solute carrier family 5 (sodium/glucose cotransporter), member 11	-2.02	3.60E-06
226781	Slc30a10	solute carrier family 30, member 10	-2.03	0.00086
19012	Ppap2a	phosphatidic acid phosphatase type 2A	-2.03	1.00E-06
225913	Dak	dihydroxyacetone kinase 2 homolog (yeast)	-2.03	3.00E-05
230088	B230312A22Rik	RIKEN cDNA B230312A22 gene	-2.03	2.90E-07
67689	Aldh3b1	aldehyde dehydrogenase 3 family, member B1	-2.03	1.60E-06
19329	ILMN_215232	Mus musculus RAB17, member RAS oncogene family (Rab17), mRNA.	-2.04	2.50E-05
217212	Pyy	peptide YY	-2.04	3.20E-07
547253	Parp14	poly (ADP-ribose) polymerase family, member 14	-2.04	3.40E-05
69146	Gsdmd	gasdermin D	-2.04	8.20E-06
319625	Galm	galactose mutarotase	-2.04	8.80E-08
13216	ILMN_196581	Mus musculus defensin, alpha 1 (Defa1), mRNA.	-2.04	2.30E-05
170752	Bco2	beta-carotene oxygenase 2	-2.05	6.60E-06
16173	Il18	interleukin 18	-2.06	5.10E-05
69146	Gsdmd	gasdermin D	-2.06	2.80E-06
66822	Fbxo25	F-box protein 25	-2.06	4.40E-07
104086	Cyp27a1	cytochrome P450, family 27, subfamily a, polypeptide 1	-2.06	5.30E-06
104086	Cyp27a1	cytochrome P450, family 27, subfamily a, polypeptide 1	-2.06	4.30E-08
74190	1200009I06Rik	RIKEN cDNA 1200009I06 gene	-2.06	2.20E-06
13026	Pcyt1a	phosphate cytidylyltransferase 1, choline, alpha isoform	-2.07	6.60E-07
231510	ILMN_189297	Mus musculus 1-acylglycerol-3-phosphate O-acyltransferase 9 (Agpat9), transcript variant 1, mRNA.	-2.07	1.90E-06
13170	Dbp	D site albumin promoter binding protein	-2.08	0.044
93694	Clec2d	C-type lectin domain family 2, member d	-2.08	8.20E-07
11421	Ace	angiotensin I converting enzyme (peptidyl-dipeptidase A) 1	-2.08	8.80E-07
75604	Tm4sf5	transmembrane 4 superfamily member 5	-2.09	0.00015
240055	Neurl1b	neuralized homolog 1b (Drosophila)	-2.09	3.20E-08
69146	Gsdmd	gasdermin D	-2.1	1.90E-06
231821	Adap1	ArfGAP with dual PH domains 1	-2.1	5.60E-08
53880	ILMN_230036	Mus musculus NLR family, apoptosis inhibitory protein 7 (Naip7), mRNA.	-2.11	9.70E-08
71664	Mettl7b	methyltransferase like 7B	-2.11	5.70E-06
243813	Leng9	leukocyte receptor cluster (LRC) member 9	-2.11	6.60E-06
381246	Xkr9	X Kell blood group precursor related family member 9 homolog	-2.12	1.50E-07
228980	Taf4a	TAF4A RNA polymerase II, TATA box binding protein (TBP)-associated factor	-2.12	2.10E-06
20540	Slc7a7	solute carrier family 7 (cationic amino acid transporter, y+ system), member 7	-2.12	1.80E-06
330064	Slc5a6	solute carrier family 5 (sodium-dependent vitamin transporter), member 6	-2.12	5.60E-08
217721	Mfsd7c	major facilitator superfamily domain containing 7C	-2.12	1.60E-06
16419	Itgb5	integrin beta 5	-2.12	1.20E-05
20568	Slpi	secretory leukocyte peptidase inhibitor	-2.13	0.004
19116	Prlr	prolactin receptor	-2.13	3.70E-07
68591	Mocos	molybdenum cofactor sulfurase	-2.13	1.70E-06
76279	Cyp2d26	cytochrome P450, family 2, subfamily d, polypeptide 26	-2.13	0.00012
70261	2010110P09Rik	RIKEN cDNA 2010110P09 gene	-2.13	6.60E-06
20312	Cx3cl1	chemokine (C-X3-C motif) ligand 1	-2.14	2.40E-06
320709	Tmem117	transmembrane protein 117	-2.15	4.90E-05
99586	Dpyd	dihydropyrimidine dehydrogenase	-2.15	8.90E-07
67800	Dgat2	diacylglycerol O-acyltransferase 2	-2.15	3.80E-06
239393	Lrp12	low density lipoprotein-related protein 12	-2.16	6.00E-07
57319	Smpdl3a	sphingomyelin phosphodiesterase, acid-like 3A	-2.17	4.20E-06
230163	Aldob	aldolase B, fructose-bisphosphate	-2.18	1.10E-05
68846	Rnf208	ring finger protein 208	-2.19	1.10E-05
71648	ILMN_216896	Mus musculus optineurin (Optn), mRNA.	-2.19	2.90E-07
12502	Cd3g	CD3 antigen, gamma polypeptide	-2.19	5.00E-05
66895	1300014I06Rik	RIKEN cDNA 1300014I06 gene	-2.19	4.90E-07
268480	Rapgefl1	Rap guanine nucleotide exchange factor (GEF)-like 1	-2.2	6.90E-08
270198	Pfkfb4	6-phosphofructo-2-kinase/fructose-2,6-biphosphatase 4	-2.2	5.10E-08
66054	Cndp2	CNDP dipeptidase 2 (metallopeptidase M20 family)	-2.2	6.90E-08
20510	Slc1a1	solute carrier family 1 (neuronal/epithelial high affinity glutamate transporter, system Xag), member 1	-2.21	6.30E-08
26456	Sema4g	sema domain, immunoglobulin domain (Ig), transmembrane domain (TM) and short cytoplasmic domain, (semaphorin) 4G	-2.21	6.00E-07
58210	Sectm1b	secreted and transmembrane 1B	-2.21	5.30E-05
19017	Ppargc1a	peroxisome proliferative activated receptor, gamma, coactivator 1 alpha	-2.21	1.90E-06
223646	Naprt1	nicotinate phosphoribosyltransferase domain containing 1	-2.21	9.00E-08
320415	Gchfr	GTP cyclohydrolase I feedback regulator	-2.21	2.20E-07
13113	Cyp3a13	cytochrome P450, family 3, subfamily a, polypeptide 13	-2.21	4.20E-05
76279	Cyp2d26	cytochrome P450, family 2, subfamily d, polypeptide 26	-2.21	1.00E-04
66469	2810405K02Rik	RIKEN cDNA 2810405K02 gene	-2.21	2.90E-07
209387	Trim30d	tripartite motif-containing 30D	-2.22	7.30E-07
50934	Slc7a8	solute carrier family 7 (cationic amino acid transporter, y+ system), member 8	-2.23	3.30E-07
545156	Kalrn	kalirin, RhoGEF kinase	-2.23	2.70E-06
76279	Cyp2d26	cytochrome P450, family 2, subfamily d, polypeptide 26	-2.24	0.00012

54200	Sult2b1	sulfotransferase family, cytosolic, 2B, member 1	-2.25	6.90E-08
66222	Serpinb1a	serine (or cysteine) peptidase inhibitor, clade B, member 1a	-2.25	2.20E-07
13120	Cyp4b1	cytochrome P450, family 4, subfamily b, polypeptide 1	-2.25	2.10E-07
213019	Pdlim2	PDZ and LIM domain 2	-2.26	2.80E-06
56209	Gde1	glycerophosphodiester phosphodiesterase 1	-2.26	4.10E-08
11832	Aqp7	aquaporin 7	-2.26	5.90E-06
74190	1200009I06Rik	RIKEN cDNA 1200009I06 gene	-2.26	5.20E-08
71648	ILMN_216896	Mus musculus optineurin (Optn), mRNA.	-2.27	1.10E-07
16600	Klf4	Kruppel-like factor 4 (gut)	-2.27	1.00E-06
11522	Adh1	alcohol dehydrogenase 1 (class I)	-2.28	1.10E-05
76263	Gstk1	glutathione S-transferase kappa 1	-2.29	3.90E-09
235283	Gramd1b	GRAM domain containing 1B	-2.29	3.10E-08
70113	Odf3b	outer dense fiber of sperm tails 3B	-2.29	3.90E-09
20698	Sphk1	sphingosine kinase 1	-2.3	3.20E-07
20397	Sgpl1	sphingosine phosphate lyase 1	-2.3	5.00E-08
381122	Capn13	calpain 13	-2.3	1.70E-05
547431	Btnl2	butyrophilin-like 2	-2.3	3.70E-07
93835	Amn	amnionless	-2.31	1.40E-05
99709	AI747448	expressed sequence AI747448	-2.31	0.0039
109218	Tmem139	transmembrane protein 139	-2.32	1.30E-07
30805	Slc22a4	solute carrier family 22 (organic cation transporter), member 4	-2.32	1.00E-07
68636	Fahd1	fumarylacetoacetate hydrolase domain containing 1	-2.32	7.00E-08
56209	Gde1	glycerophosphodiester phosphodiesterase 1	-2.33	1.10E-07
54722	Dfna5	deafness, autosomal dominant 5 (human)	-2.33	1.80E-06
328059	Slc7a15	solute carrier family 7 (cationic amino acid transporter, y+ system), member 15	-2.34	4.60E-05
268663	Cdhr2	cadherin-related family member 2	-2.34	1.00E-06
224762	Trim31	tripartite motif-containing 31	-2.35	1.60E-07
64452	Slc5a4a	solute carrier family 5, member 4a	-2.36	3.40E-07
50778	Rgs1	regulator of G-protein signaling 1	-2.38	6.60E-06
270328	ILMN_196360	Mus musculus gasdermin C3 (Gsdmc3), mRNA.	-2.38	3.60E-05
21452	Tcn2	transcobalamin 2	-2.39	5.10E-07
268663	Cdhr2	cadherin-related family member 2	-2.39	4.90E-07
11421	Ace	angiotensin I converting enzyme (peptidyl-dipeptidase A) 1	-2.39	1.60E-06
16204	Fabp6	fatty acid binding protein 6, ileal (gastrotropin)	-2.4	0.002
102857	Slc6a8	solute carrier family 6 (neurotransmitter transporter, creatine), member 8	-2.41	1.80E-06
67131	Acbd4	acyl-Coenzyme A binding domain containing 4	-2.41	1.30E-08
69826	Ms4a10	membrane-spanning 4-domains, subfamily A, member 10	-2.42	3.00E-06
75541	1700019G17Rik	RIKEN cDNA 1700019G17 gene	-2.42	2.50E-07
20604	Sst	somatostatin	-2.43	6.20E-06
19012	Ppap2a	phosphatidic acid phosphatase type 2A	-2.43	1.30E-08
12263	C2	complement component 2 (within H-2S)	-2.43	9.40E-05
52793	Fam3b	family with sequence similarity 3, member B	-2.44	2.40E-07
15013	H2-Q2	histocompatibility 2, Q region locus 2	-2.44	4.60E-06
12632	Cfl2	cofilin 2, muscle	-2.44	1.50E-05
56209	Gde1	glycerophosphodiester phosphodiesterase 1	-2.45	9.00E-08
218454	Lhfpl2	lipoma HMGIC fusion partner-like 2	-2.45	1.30E-06
544963	Iqgap2	IQ motif containing GTPase activating protein 2	-2.45	3.60E-07
67131	Acbd4	acyl-Coenzyme A binding domain containing 4	-2.45	4.50E-07
11666	Abcd1	ATP-binding cassette, sub-family D (ALD), member 1	-2.45	1.80E-07
26941	Slc9a3r1	solute carrier family 9 (sodium/hydrogen exchanger), member 3 regulator 1	-2.46	1.50E-07
13350	Dgat1	diacylglycerol O-acyltransferase 1	-2.46	7.10E-08
64385	Cyp4f14	cytochrome P450, family 4, subfamily f, polypeptide 14	-2.46	5.00E-06
67038	2010109I03Rik	RIKEN cDNA 2010109I03 gene	-2.48	2.00E-04
170761	Pdzd3	PDZ domain containing 3	-2.49	6.00E-07
217214	Nags	N-acetylglutamate synthase	-2.49	1.90E-08
13139	Dgka	diacylglycerol kinase, alpha	-2.49	1.50E-08
13139	Dgka	diacylglycerol kinase, alpha	-2.49	3.90E-09
545288	Cyp2c67	cytochrome P450, family 2, subfamily c, polypeptide 67	-2.49	5.70E-07
17288	Mep1b	meprin 1 beta	-2.51	4.90E-05
268663	Cdhr2	cadherin-related family member 2	-2.51	5.30E-06
20510	Slc1a1	solute carrier family 1 (neuronal/epithelial high affinity glutamate transporter, system Xag), member 1	-2.52	8.10E-09
17524	Mpp1	membrane protein, palmitoylated	-2.52	8.00E-07
69574	Cmbl	carboxymethylenebutenolidase-like (Pseudomonas)	-2.52	2.40E-07
104252	Cdc42ep2	CDC42 effector protein (Rho GTPase binding) 2	-2.52	3.60E-07
434203	ILMN_244381	Mus musculus solute carrier family 28 (sodium-coupled nucleoside transporter), member 1 (Slc28a1), mRNA.	-2.55	8.50E-07
18624	Pepd	peptidase D	-2.56	5.60E-08
17777	Mttp	microsomal triglyceride transfer protein	-2.57	3.90E-07
70113	Odf3b	outer dense fiber of sperm tails 3B	-2.57	5.10E-08
231510	ILMN_189297	Mus musculus 1-acylglycerol-3-phosphate O-acyltransferase 9 (Agpat9), transcript variant 1, mRNA.	-2.59	4.40E-07
140742	ILMN_253855	Mus musculus sestrin 1 (Sesn1), mRNA.	-2.6	8.90E-07
19660	Rbp2	retinol binding protein 2, cellular	-2.6	7.00E-06
632778	Gm7092	predicted gene 7092	-2.6	1.00E-06
18947	Pnliprp2	pancreatic lipase-related protein 2	-2.61	0.00017
223646	Naprt1	nicotinate phosphoribosyltransferase domain containing 1	-2.61	2.80E-08
14377	G6pc	glucose-6-phosphatase, catalytic	-2.61	7.50E-06
71839	Osgin1	oxidative stress induced growth inhibitor 1	-2.62	3.90E-08
52793	Fam3b	family with sequence similarity 3, member B	-2.62	4.20E-08
232409	Clec2e	C-type lectin domain family 2, member e	-2.62	1.40E-07
233549	Mogat2	monoacylglycerol O-acyltransferase 2	-2.63	6.30E-07
69787	Anxa13	annexin A13	-2.63	2.50E-06
22436	Xdh	xanthine dehydrogenase	-2.64	4.40E-07
18947	Pnliprp2	pancreatic lipase-related protein 2	-2.64	0.00014
268480	Rapgefl1	Rap guanine nucleotide exchange factor (GEF)-like 1	-2.65	8.50E-07
381334	ILMN_196607	Mus musculus galactose-3-O-sulfotransferase 2 (Gal3st2), mRNA.	-2.65	5.70E-07
24108	Ubd	ubiquitin D	-2.66	0.0045
26569	Slc27a4	solute carrier family 27 (fatty acid transporter), member 4	-2.66	2.10E-07
54200	Sult2b1	sulfotransferase family, cytosolic, 2B, member 1	-2.67	3.90E-09
20459	Ptk6	PTK6 protein tyrosine kinase 6	-2.67	4.40E-07
93842	Igsf9	immunoglobulin superfamily, member 9	-2.67	4.20E-08
67389	Fam132a	family with sequence similarity 132, member A	-2.67	3.00E-09
213522	Plekhg6	pleckstrin homology domain containing, family G (with RhoGef domain) member 6	-2.68	7.30E-08
17951	Naip5	NLR family, apoptosis inhibitory protein 5	-2.68	7.50E-09
226564	Fmo4	flavin containing monooxygenase 4	-2.68	8.50E-08
14102	Fas	Fas (TNF receptor superfamily member 6)	-2.69	4.30E-08
241303	ILMN_210026	Mus musculus RIKEN cDNA A130092J06 gene (A130092J06Rik), mRNA.	-2.69	2.90E-07
107589	Mylk	myosin, light polypeptide kinase	-2.7	1.30E-06
13370	Dio1	deiodinase, iodothyronine, type I	-2.7	1.20E-07
67470	ILMN_213808	Mus musculus ATP-binding cassette, sub-family G (WHITE), member 8 (Abcg8), mRNA.	-2.7	2.30E-07
223631	ILMN_209571	Mus musculus cDNA sequence BC025446 (BC025446), mRNA.	-2.71	4.90E-07
68947	Chst8	carbohydrate (N-acetylgalactosamine 4-0) sulfotransferase 8	-2.72	4.90E-07
223920	Soat2	sterol O-acyltransferase 2	-2.73	0.00014
26384	ILMN_226523	Mus musculus glucosamine-6-phosphate deaminase 1 (Gnpda1), mRNA.	-2.73	3.80E-08
12684	Cideb	cell death-inducing DNA fragmentation factor, alpha subunit-like effector B	-2.73	2.00E-08
13482	Dpp4	dipeptidylpeptidase 4	-2.74	6.30E-08
228576	Mall	mal, T-cell differentiation protein-like	-2.75	1.30E-07
76282	Gpt	glutamic pyruvic transaminase, soluble	-2.75	2.50E-06
22264	Prap1	proline-rich acidic protein 1	-2.76	0.0027
11806	Apoa1	apolipoprotein A-I	-2.76	0.031
13088	Cyp2b10	cytochrome P450, family 2, subfamily b, polypeptide 10	-2.77	0.001
11522	Adh1	alcohol dehydrogenase 1 (class I)	-2.77	2.80E-06
231396	Ugt2b36	UDP glucuronosyltransferase 2 family, polypeptide B36	-2.78	9.00E-08
233549	Mogat2	monoacylglycerol O-acyltransferase 2	-2.78	1.40E-05
17288	Mep1b	meprin 1 beta	-2.78	0.00015
27409	Abcg5	ATP-binding cassette, sub-family G (WHITE), member 5	-2.78	3.00E-07
24059	Slco2a1	solute carrier organic anion transporter family, member 2a1	-2.79	4.70E-06
17965	Nbl1	neuroblastoma, suppression of tumorigenicity 1	-2.79	8.20E-09
68270	ILMN_218483	Mus musculus leucine rich repeat containing 50 (Lrrc50), mRNA.	-2.79	4.30E-08

192970	Dhrs11	dehydrogenase/reductase (SDR family) member 11	-2.8	2.10E-08
17289	Mertk	c-mer proto-oncogene tyrosine kinase	-2.81	3.30E-07
12182	ILMN_214474	Mus musculus bone marrow stromal cell antigen 1 (Bst1), mRNA.	-2.81	8.80E-09
76507	Abp1	amiloride binding protein 1 (amine oxidase, copper-containing)	-2.81	3.00E-08
330962	Ostb	organic solute transporter beta	-2.82	1.50E-06
20537	Slc5a1	solute carrier family 5 (sodium/glucose cotransporter), member 1	-2.83	3.40E-08
20730	Spink3	serine peptidase inhibitor, Kazal type 3	-2.84	1.10E-06
69836	Pla2g12b	phospholipase A2, group XIIB	-2.84	4.20E-06
107589	Mylk	myosin, light polypeptide kinase	-2.84	2.60E-07
68255	Tmem86b	transmembrane protein 86B	-2.85	2.50E-09
18858	Pmp22	peripheral myelin protein 22	-2.85	1.90E-06
13131	Dab1	disabled homolog 1 (Drosophila)	-2.85	4.90E-10
208677	Creb3l3	cAMP responsive element binding protein 3-like 3	-2.86	1.80E-06
223631	ILMN_209571	Mus musculus cDNA sequence BC025446 (BC025446), mRNA.	-2.86	4.30E-08
70008	ILMN_215632	Mus musculus angiotensin I converting enzyme (peptidyl-dipeptidase A) 2 (Ace2), mRNA.	-2.86	0.00019
20300	Ccl25	chemokine (C-C motif) ligand 25	-2.88	1.00E-06
12266	C3	complement component 3	-2.88	0.00037
66809	Krt20	keratin 20	-2.89	3.10E-07
16002	Igf2	insulin-like growth factor 2	-2.89	0.00017
56643	Slc15a1	solute carrier family 15 (oligopeptide transporter), member 1	-2.9	5.30E-06
99663	Clca6	chloride channel calcium activated 6	-2.9	0.00075
20300	ILMN_215623	Mus musculus chemokine (C-C motif) ligand 25 (Ccl25), mRNA.	-2.9	1.10E-05
170745	ILMN_256045	Mus musculus X-prolyl aminopeptidase (aminopeptidase P) 2, membrane-bound (Xpnpep2), transcript variant 1, mRNA.	-2.91	6.30E-08
107770	Tm6sf2	transmembrane 6 superfamily member 2	-2.92	1.20E-07
56226	Espn	espin	-2.92	2.50E-07
13479	Dpep1	dipeptidase 1 (renal)	-2.92	2.00E-06
106407	Osta	organic solute transporter alpha	-2.93	0.00058
17288	Mep1b	meprin 1 beta	-2.94	5.90E-06
76960	Bcas1	breast carcinoma amplified sequence 1	-2.96	5.80E-10
74338	Slc6a19	solute carrier family 6 (neurotransmitter transporter), member 19	-2.97	1.10E-05
74134	Cyp2s1	cytochrome P450, family 2, subfamily s, polypeptide 1	-2.97	1.30E-08
268860	Abat	4-aminobutyrate aminotransferase	-3.01	1.10E-07
67082	1700011H14Rik	RIKEN cDNA 1700011H14 gene	-3.01	1.30E-07
381204	Naaladl1	N-acetylated alpha-linked acidic dipeptidase-like 1	-3.03	8.80E-07
99709	AI747448	expressed sequence AI747448	-3.03	1.00E-05
18858	Pmp22	peripheral myelin protein 22	-3.04	3.60E-06
16548	Khk	ketohexokinase	-3.04	3.90E-08
72303	ILMN_229278	Mus musculus cytochrome P450, family 2, subfamily c, polypeptide 65 (Cyp2c65), mRNA.	-3.04	1.60E-06
216019	Hkdc1	hexokinase domain containing 1	-3.05	1.80E-07
63954	Rbp7	retinol binding protein 7, cellular	-3.07	6.00E-07
212647	ILMN_241583	Mus musculus aldehyde dehydrogenase 4 family, member A1 (Aldh4a1), nuclear gene encoding mitochondrial protein, mRNA.	-3.08	1.10E-05
20526	Slc2a2	solute carrier family 2 (facilitated glucose transporter), member 2	-3.09	7.30E-06
230579	Fam151a	family with sequence simliarity 151, member A	-3.09	5.10E-07
22271	Upp1	uridine phosphorylase 1	-3.1	9.30E-05
234673	Ces2e	carboxylesterase 2E	-3.1	6.60E-07
67307	Pbld2	phenazine biosynthesis-like protein domain containing 2	-3.1	8.30E-10
22271	Upp1	uridine phosphorylase 1	-3.12	1.50E-05
71706	Slc46a3	solute carrier family 46, member 3	-3.13	3.80E-08
54150	Rdh7	retinol dehydrogenase 7	-3.13	1.20E-08
12579	Cdkn2b	cyclin-dependent kinase inhibitor 2B (p15, inhibits CDK4)	-3.13	2.30E-08
54200	Sult2b1	sulfotransferase family, cytosolic, 2B, member 1	-3.14	3.30E-09
69123	ILMN_210310	Mus musculus RIKEN cDNA 1810022C23 gene (1810022C23Rik), mRNA.	-3.14	7.30E-10
23959	Nt5e	5' nucleotidase, ecto	-3.15	1.70E-05
13850	Ephx2	epoxide hydrolase 2, cytoplasmic	-3.15	4.90E-07
13615	Edn2	endothelin 2	-3.15	1.10E-05
13370	Dio1	deiodinase, iodothyronine, type I	-3.15	2.10E-08
665270	Plb1	phospholipase B1	-3.16	2.00E-09
30962	Slc7a9	solute carrier family 7 (cationic amino acid transporter, y+ system), member 9	-3.18	3.90E-06
17287	Mep1a	meprin 1 alpha	-3.2	1.70E-05
56226	Espn	espin	-3.21	1.80E-08
20500	Slc13a2	solute carrier family 13 (sodium-dependent dicarboxylate transporter), member 2	-3.22	3.20E-06
381204	Naaladl1	N-acetylated alpha-linked acidic dipeptidase-like 1	-3.22	3.90E-06
22271	Upp1	uridine phosphorylase 1	-3.23	2.20E-05
15930	Ido1	indoleamine 2,3-dioxygenase 1	-3.23	0.00096
14263	ILMN_211866	Mus musculus flavin containing monooxygenase 5 (Fmo5), mRNA.	-3.23	8.20E-09
71584	Gdpd2	glycerophosphodiester phosphodiesterase domain containing 2	-3.24	3.80E-11
21810	Tgfbi	transforming growth factor, beta induced	-3.26	6.60E-09
16548	Khk	ketohexokinase	-3.26	1.40E-08
56448	ILMN_223233	Mus musculus cytochrome P450, family 2, subfamily d, polypeptide 22 (Cyp2d22), mRNA.	-3.27	1.90E-06
381204	Naaladl1	N-acetylated alpha-linked acidic dipeptidase-like 1	-3.28	2.10E-06
12351	Car4	carbonic anhydrase 4	-3.29	4.30E-08
231396	Ugt2b36	UDP glucuronosyltransferase 2 family, polypeptide B36	-3.32	3.90E-09
16790	Anpep	alanyl (membrane) aminopeptidase	-3.33	7.00E-04
30962	Slc7a9	solute carrier family 7 (cationic amino acid transporter, y+ system), member 9	-3.35	8.80E-07
230163	Aldob	aldolase B, fructose-bisphosphate	-3.39	1.20E-06
50934	Slc7a8	solute carrier family 7 (cationic amino acid transporter, y+ system), member 8	-3.43	2.10E-09
54150	Rdh7	retinol dehydrogenase 7	-3.43	1.10E-09
12865	Cox7a1	cytochrome c oxidase, subunit VIIa 1	-3.43	4.10E-09
17380	ILMN_220122	Mus musculus membrane metallo endopeptidase (Mme), mRNA.	-3.46	2.20E-07
71601	ILMN_213888	Mus musculus CEA-related cell adhesion molecule 20 (Ceacam20), mRNA.	-3.47	2.20E-08
15567	Slc6a4	solute carrier family 6 (neurotransmitter transporter, serotonin), member 4	-3.49	8.80E-10
171429	ILMN_216189	Mus musculus solute carrier family 26, member 6 (Slc26a6), mRNA.	-3.49	7.30E-07
14170	Fgf15	fibroblast growth factor 15	-3.54	6.90E-08
67473	Slc47a1	solute carrier family 47, member 1	-3.54	6.30E-07
14963	ILMN_208890	Mus musculus histocompatibility 2, blastocyst (H2-Bl), mRNA.	-3.56	6.30E-08
67893	Tmem86a	transmembrane protein 86A	-3.58	3.50E-08
58866	Treh	trehalase (brush-border membrane glycoprotein)	-3.59	6.70E-06
71733	Susd2	sushi domain containing 2	-3.59	6.20E-08
101488	Slco2b1	solute carrier organic anion transporter family, member 2b1	-3.6	1.20E-06
56388	Cyp3a25	cytochrome P450, family 3, subfamily a, polypeptide 25	-3.66	5.90E-08
23844	Clca3	chloride channel calcium activated 3	-3.68	2.80E-06
11808	Apoa4	apolipoprotein A-IV	-3.68	0.00048
20443	St3gal4	ST3 beta-galactoside alpha-2,3-sialyltransferase 4	-3.7	1.30E-08
170745	Xpnpep2	X-prolyl aminopeptidase (aminopeptidase P) 2, membrane-bound	-3.71	5.70E-06
20531	Slc34a2	solute carrier family 34 (sodium phosphate), member 2	-3.71	5.00E-06
19694	Reg3a	regenerating islet-derived 3 alpha	-3.75	4.10E-07
109731	Maob	monoamine oxidase B	-3.76	2.30E-08
20259	Scin	scinderin	-3.77	2.50E-08
433470	AA467197	expressed sequence AA467197	-3.78	4.80E-08
216225	Slc5a8	solute carrier family 5 (iodide transporter), member 8	-3.83	9.00E-08
331063	Gsdmc2	gasdermin C2	-3.84	5.70E-07
53315	Sult1d1	sulfotransferase family 1D, member 1	-3.87	1.30E-08
11997	Akr1b7	aldo-keto reductase family 1, member B7	-3.87	1.40E-07
68396	Nat8	N-acetyltransferase 8 (GCN5-related, putative)	-3.97	3.40E-08
54447	Asah2	N-acylsphingosine amidohydrolase 2	-4.03	4.10E-08
237636	Npc1l1	NPC1-like 1	-4.12	2.60E-07
238011	Enpp7	ectonucleotide pyrophosphatase/phosphodiesterase 7	-4.14	2.50E-08
64454	Slc5a4b	solute carrier family 5 (neutral amino acid transporters, system A), member 4b	-4.18	4.20E-11
331063	ILMN_196357	Mus musculus expressed sequence AI987692 (AI987692), mRNA.	-4.22	1.40E-06
102294	Cyp4v3	cytochrome P450, family 4, subfamily v, polypeptide 3	-4.3	1.80E-06
107375	Slc25a45	solute carrier family 25, member 45	-4.36	1.00E-07
11847	Arg2	arginase type II	-4.37	7.00E-08
20363	Sepp1	selenoprotein P, plasma, 1	-4.52	1.10E-06
59020	Pdzk1	PDZ domain containing 1	-4.52	5.30E-05
20363	Sepp1	selenoprotein P, plasma, 1	-4.58	5.40E-07
238011	Enpp7	ectonucleotide pyrophosphatase/phosphodiesterase 7	-4.58	3.40E-08
21810	Tgfbi	transforming growth factor, beta induced	-4.63	2.30E-07

69983	Sis	sucrase isomaltase (alpha-glucosidase)	-4.63	8.20E-06
11814	Apoc3	apolipoprotein C-III	-5.08	4.40E-05
100045250	ILMN_196357	PREDICTED: Mus musculus hypothetical protein LOC100045250 (LOC100045250), misc RNA.	-5.14	4.60E-07
13419	Dnase1	deoxyribonuclease I	-5.97	2.30E-08

Supplementary Table S2

Differentialy expressed genes (|logFC| ≥ 1, q-value < 0.05) in the colonic epithelium 2 days upon Apc depletion compared to tissue with intact Apc

ENTREZ	SYMBOL	GENENAME	logFC	q-value
626708	Defa26	defensin, alpha, 26	2.38	5.00E-04
13218	Defa-rs1	defensin, alpha, related sequence 1	2.34	0.028
68009	ILMN_196346	Mus musculus defensin related cryptdin 20 (Defcr20), mRNA.	2.28	0.0037
13239	ILMN_196558	Mus musculus defensin related cryptdin 5 (Defcr5), mRNA.	2.11	0.026
68009	ILMN_196346	Mus musculus defensin related cryptdin 20 (Defcr20), mRNA.	2.01	0.0037
100044291	ILMN_221210	PREDICTED: Mus musculus hypothetical protein LOC100044291 (LOC100044291), mRNA.	1.99	0.016
17110	Lyz1	lysozyme 1	1.88	0.0096
13216	ILMN_196581	Mus musculus defensin, alpha 1 (Defa1), mRNA.	1.86	0.016
13240	Defa6	defensin, alpha, 6	1.82	0.031
17110	Lyz1	lysozyme 1	1.63	0.028
17748	Mt1	metallothionein 1	1.35	0.031
11551	ILMN_190996	Mus musculus adrenergic receptor, alpha 2a (Adra2a), mRNA.	1.28	0.026
23945	Mgll	monoglyceride lipase	1.25	0.031
213391	Rassf4	Ras association (RalGDS/AF-6) domain family member 4	1.02	0.031
12231	Btn1a1	butyrophilin, subfamily 1, member A1	-1.07	0.016
16987	ILMN_187484	Mus musculus lanosterol synthase (Lss), mRNA.	-1.12	0.016
64177	Trpv6	transient receptor potential cation channel, subfamily V, member 6	-1.33	0.04

Supplementary Table S2

Differentialy expressed genes (|logFC| ≥ 1, q-value < 0.05) in the colonic epithelium 4 days upon Apc depletion compared to tissue with intact Apc

ENTREZ	SYMBOL	GENENAME	logFC	q-value
12709.00	ILMN_193661	Mus musculus creatine kinase, brain (Ckb), mRNA.	3.86	2.50E-08
74186.00	Ccdc3	coiled-coil domain containing 3	3.17	6.40E-10
20568.00	Slpi	secretory leukocyte peptidase inhibitor	3.09	0.00018
73710.00	Tubb2b	tubulin, beta 2B	3.03	2.50E-09
17329.00	Cxcl9	chemokine (C-X-C motif) ligand 9	2.89	3.80E-05
66141.00	Ifitm3	interferon induced transmembrane protein 3	2.86	8.10E-08
15945.00	ILMN_253583	Mus musculus chemokine (C-X-C motif) ligand 10 (Cxcl10), mRNA.	2.7	0.0024
213948.00	Atg9b	ATG9 autophagy related 9 homolog B (S. cerevisiae)	2.58	1.90E-05
14969.00	H2-Eb1	histocompatibility 2, class II antigen E beta	2.43	0.00014
14570.00	Arhgdig	Rho GDP dissociation inhibitor (GDI) gamma	2.42	3.10E-07
15930.00	Ido1	indoleamine 2,3-dioxygenase 1	2.34	0.012
15937.00	Ier3	immediate early response 3	2.22	1.70E-07
16010.00	Igfbp4	insulin-like growth factor binding protein 4	2.21	1.30E-06
26897.00	ILMN_223756	Mus musculus acyl-CoA thioesterase 1 (Acot1), mRNA.	2.21	8.00E-06
19752.00	Rnase1	ribonuclease, RNase A family, 1 (pancreatic)	2.19	2.60E-05
66214.00	1190002H23Rik	RIKEN cDNA 1190002H23 gene	2.17	0.00017
100047619.00	ILMN_219663	PREDICTED: Mus musculus similar to solute carrier family 7 (cationic amino acid transporter, y+ system), member 5 (LOC100047619), misc RNA.	2.16	9.20E-07
14160.00	Lgr5	leucine rich repeat containing G protein coupled receptor 5	2.13	2.40E-07
270152.00	Amica1	adhesion molecule, interacts with CXADR antigen 1	2.12	1.40E-06
14609.00	Gja1	gap junction protein, alpha 1	1.99	5.50E-05
11459.00	Acta1	actin, alpha 1, skeletal muscle	1.99	4.90E-05
27280.00	Phlda3	pleckstrin homology-like domain, family A, member 3	1.98	0.00039
17218.00	Mcm5	minichromosome maintenance deficient 5, cell division cycle 46 (S. cerevisiae)	1.97	2.80E-08
16145.00	Igtp	interferon gamma induced GTPase	1.97	0.025
320685.00	Dctd	dCMP deaminase	1.95	3.10E-05
328162.00	Trmt61a	tRNA methyltransferase 61 homolog A (S. cerevisiae)	1.95	2.50E-06
100608.00	Noc4l	nucleolar complex associated 4 homolog (S. cerevisiae)	1.93	4.00E-06
17067.00	Ly6c1	lymphocyte antigen 6 complex, locus C1	1.93	0.00015
11925.00	Neurog3	neurogenin 3	1.88	9.30E-06
72462.00	Rrp1b	ribosomal RNA processing 1 homolog B (S. cerevisiae)	1.87	9.80E-07
13401.00	Dmwd	dystrophia myotonica-containing WD repeat motif	1.87	1.70E-07
30927.00	Snai3	snail homolog 3 (Drosophila)	1.86	2.00E-08
27280.00	Phlda3	pleckstrin homology-like domain, family A, member 3	1.85	0.00019
14028.00	Evx1	even skipped homeotic gene 1 homolog	1.85	2.70E-07
68915.00	Vars2	valyl-tRNA synthetase 2, mitochondrial (putative)	1.84	1.10E-07
26897.00	Acot1	acyl-CoA thioesterase 1	1.82	3.90E-06
102614.00	Rpp25	ribonuclease P 25 subunit (human)	1.8	4.80E-07
17216.00	Mcm2	minichromosome maintenance deficient 2 mitotin (S. cerevisiae)	1.8	5.10E-08
21677.00	Tead2	TEA domain family member 2	1.79	1.80E-06
16673.00	Krt36	keratin 36	1.79	6.90E-10
17173.00	Ascl2	achaete-scute complex homolog 2 (Drosophila)	1.79	4.40E-05
20361.00	Sema7a	sema domain, immunoglobulin domain (Ig), and GPI membrane anchor, (semaphorin) 7A	1.78	1.60E-05
110749.00	ILMN_215876	Mus musculus chromatin assembly factor 1, subunit B (p60) (Chaf1b), mRNA.	1.78	3.50E-07
64406.00	Sp5	trans-acting transcription factor 5	1.77	5.80E-07
18432.00	Mybbp1a	MYB binding protein (P160) 1a	1.77	7.10E-06
171212.00	Galnt10	UDP-N-acetyl-alpha-D-galactosamine:polypeptide N-acetylgalactosaminyltransferase 10	1.76	3.90E-08
18383.00	Tnfrsf11b	tumor necrosis factor receptor superfamily, member 11b (osteoprotegerin)	1.75	8.50E-06
100608.00	ILMN_209940	Mus musculus nucleolar complex associated 4 homolog (S. cerevisiae) (Noc4l), mRNA.	1.75	2.20E-06
69902.00	Mrto4	MRT4, mRNA turnover 4, homolog (S. cerevisiae)	1.75	5.60E-07
15469.00	Prmt1	protein arginine N-methyltransferase 1	1.75	4.80E-07
18780.00	Pla2g2a	phospholipase A2, group IIA (platelets, synovial fluid)	1.74	0.00015
17215.00	ILMN_213080	Mus musculus minichromosome maintenance deficient 3 (S. cerevisiae) (Mcm3), mRNA.	1.74	2.50E-08
12505.00	Cd44	CD44 antigen	1.74	3.60E-06
51797.00	Ctps	cytidine 5'-triphosphate synthase	1.73	3.20E-06
22321.00	Vars	valyl-tRNA synthetase	1.72	6.20E-08
14961.00	H2-Ab1	histocompatibility 2, class II antigen A, beta 1	1.72	0.0022
22041.00	Trf	transferrin	1.71	1.10E-06
14961.00	H2-Ab1	histocompatibility 2, class II antigen A, beta 1	1.71	0.0017
17865.00	ILMN_212854	Mus musculus myeloblastosis oncogene-like 2 (Mybl2), mRNA.	1.7	6.40E-10
71805.00	Nup93	nucleoporin 93	1.69	1.70E-06
14998.00	ILMN_224639	Mus musculus histocompatibility 2, class II, locus DMa (H2-DMa), mRNA.	1.69	0.0018
73710.00	2410129E14Rik	Mus musculus tubulin, beta 2b (Tubb2b), mRNA.	1.68	1.90E-05
20810.00	Srm	spermidine synthase	1.68	5.80E-05

208638.00	ILMN_211336	Mus musculus solute carrier family 25, member 38 (Slc25a38), mRNA.	1.67	6.20E-08
17319.00	Mif	macrophage migration inhibitory factor	1.67	2.50E-08
15894.00	Icam1	intercellular adhesion molecule 1	1.67	0.00034
20810.00	Srm	spermidine synthase	1.66	2.50E-05
100340.00	ILMN_210171	Mus musculus sphingomyelin phosphodiesterase, acid-like 3B (Smpdl3b), mRNA.	1.66	1.00E-04
107272.00	Psat1	phosphoserine aminotransferase 1	1.66	3.20E-05
67177.00	Cdt1	chromatin licensing and DNA replication factor 1	1.66	1.50E-06
16149.00	Cd74	CD74 antigen (invariant polypeptide of major histocompatibility complex, class II antigen-associated)	1.66	0.013
51800.00	Bok	BCL2-related ovarian killer protein	1.66	9.60E-06
22154.00	Tubb5	tubulin, beta 5	1.65	7.30E-07
66102.00	Cxcl16	chemokine (C-X-C motif) ligand 16	1.65	0.00027
233066.00	AI428936	expressed sequence AI428936	1.65	9.20E-07
16149.00	Cd74	CD74 antigen (invariant polypeptide of major histocompatibility complex, class II antigen-associated)	1.64	0.0081
27279.00	Tnfrsf12a	tumor necrosis factor receptor superfamily, member 12a	1.63	0.0015
20810.00	Srm	spermidine synthase	1.63	8.00E-06
68147.00	Gar1	GAR1 ribonucleoprotein homolog (yeast)	1.63	1.80E-06
29870.00	Gtse1	G two S phase expressed protein 1	1.63	8.90E-06
226419.00	Dyrk3	dual-specificity tyrosine-(Y)-phosphorylation regulated kinase 3	1.63	0.00011
11792.00	Apex1	apurinic/apyrimidinic endonuclease 1	1.62	2.10E-06
20509.00	Slc19a1	solute carrier family 19 (sodium/hydrogen exchanger), member 1	1.62	1.10E-06
18817.00	Plk1	polo-like kinase 1 (Drosophila)	1.62	3.80E-05
17220.00	Mcm7	minichromosome maintenance deficient 7 (S. cerevisiae)	1.61	9.60E-05
12505.00	Cd44	CD44 antigen	1.61	7.10E-06
64424.00	ILMN_189434	Mus musculus polymerase (RNA) I polypeptide E (Polr1e), mRNA.	1.6	8.00E-06
13836.00	Epha2	Eph receptor A2	1.6	0.00026
51797.00	Ctps	cytidine 5'-triphosphate synthase	1.6	4.80E-07
68337.00	Crip2	cysteine rich protein 2	1.6	3.40E-06
217995.00	ILMN_214317	Mus musculus HEAT repeat containing 1 (Heatr1), mRNA.	1.59	7.70E-07
20595.00	Smn1	survival motor neuron 1	1.58	6.50E-07
30877.00	Gnl3	guanine nucleotide binding protein-like 3 (nucleolar)	1.58	2.10E-05
14114.00	Fbln1	fibulin 1	1.58	1.10E-05
211949.00	Spsb4	splA/ryanodine receptor domain and SOCS box containing 4	1.57	5.70E-06
16010.00	Igfbp4	insulin-like growth factor binding protein 4	1.57	0.00058
30877.00	Gnl3	guanine nucleotide binding protein-like 3 (nucleolar)	1.57	6.10E-06
20019.00	Polr1a	polymerase (RNA) I polypeptide A	1.56	3.50E-07
12006.00	Axin2	axin2	1.56	8.50E-05
70024.00	Mcm10	minichromosome maintenance deficient 10 (S. cerevisiae)	1.55	8.10E-08
17110.00	Lyz1	lysozyme 1	1.55	0.0053
353156.00	Egfl7	EGF-like domain 7	1.55	2.90E-06
66953.00	Cdca7	cell division cycle associated 7	1.55	8.00E-06
101612.00	Grwd1	glutamate-rich WD repeat containing 1	1.54	2.20E-06
27221.00	Chaf1a	chromatin assembly factor 1, subunit A (p150)	1.54	2.00E-07
244886.00	AI118078	expressed sequence AI118078	1.54	1.10E-05
23825.00	Banf1	barrier to autointegration factor 1	1.53	4.80E-07
19385.00	Ranbp1	RAN binding protein 1	1.52	3.50E-07
100019.00	Mdn1	midasin homolog (yeast)	1.52	2.00E-05
17228.00	Cma1	chymase 1, mast cell	1.52	1.60E-05
12545.00	Cdc7	cell division cycle 7 (S. cerevisiae)	1.52	1.20E-07
66350.00	Pla2g12a	phospholipase A2, group XIIA	1.51	6.80E-06
13639.00	Efna4	ephrin A4	1.51	3.10E-05
11746.00	Anxa4	annexin A4	1.51	0.034
72462.00	Rrp1b	ribosomal RNA processing 1 homolog B (S. cerevisiae)	1.5	0.0037
22113.00	Phlda2	pleckstrin homology-like domain, family A, member 2	1.49	0.00031
100044829.00	ILMN_209238	PREDICTED: Mus musculus similar to Fibrillarin, transcript variant 1 (LOC100044829), mRNA.	1.49	6.40E-07
17217.00	Mcm4	minichromosome maintenance deficient 4 homolog (S. cerevisiae)	1.48	1.70E-06
100046741.00	ILMN_190874	PREDICTED: Mus musculus similar to red-1 (LOC100046741), mRNA.	1.47	1.20E-06
102657.00	Cd276	CD276 antigen	1.47	1.00E-05
19183.00	Psmc3ip	proteasome (prosome, macropain) 26S subunit, ATPase 3, interacting protein	1.46	1.00E-04
17219.00	Mcm6	minichromosome maintenance deficient 6 (MIS5 homolog, S. pombe) (S. cerevisiae)	1.46	3.70E-07
70024.00	Mcm10	minichromosome maintenance deficient 10 (S. cerevisiae)	1.46	1.20E-05
22321.00	Vars	valyl-tRNA synthetase	1.45	7.10E-06
103733.00	ILMN_184611	Mus musculus tubulin, gamma 1 (Tubg1), mRNA.	1.45	1.00E-04
22059.00	Trp53	transformation related protein 53	1.45	6.80E-06
56390.00	Sssca1	Sjogren's syndrome/scleroderma autoantigen 1 homolog (human)	1.45	1.20E-05
56390.00	Sssca1	Sjogren's syndrome/scleroderma autoantigen 1 homolog (human)	1.45	7.10E-07
641240.00	ILMN_210638	PREDICTED: Mus musculus similar to MHC class II antigen beta chain (LOC641240), mRNA.	1.45	0.013
14156.00	Fen1	flap structure specific endonuclease 1	1.45	2.40E-06
51797.00	Ctps	cytidine 5'-triphosphate synthase	1.45	2.50E-05

20641.00	Snrpd1	small nuclear ribonucleoprotein D1	1.44	3.00E-06
56520.00	Nme4	non-metastatic cells 4, protein expressed in	1.44	1.00E-05
11475.00	Acta2	actin, alpha 2, smooth muscle, aorta	1.44	0.00071
72821.00	Scn2b	sodium channel, voltage-gated, type II, beta	1.43	4.90E-05
19366.00	Rad54l	RAD54 like (S. cerevisiae)	1.43	3.10E-07
16918.00	Mycl1	v-myc myelocytomatosis viral oncogene homolog 1, lung carcinoma derived (avian)	1.43	9.60E-05
66902.00	Mtap	methylthioadenosine phosphorylase	1.43	2.90E-06
17089.00	Lyar	Ly1 antibody reactive clone	1.43	9.60E-06
233876.00	Hirip3	HIRA interacting protein 3	1.43	2.00E-07
14733.00	Gpc1	glypican 1	1.43	2.40E-06
56505.00	Ruvbl1	RuvB-like protein 1	1.42	4.60E-07
57028.00	Pdxp	pyridoxal (pyridoxine, vitamin B6) phosphatase	1.42	1.00E-06
56520.00	Nme4	non-metastatic cells 4, protein expressed in	1.42	1.20E-05
18432.00	Mybbp1a	MYB binding protein (P160) 1a	1.42	0.00016
67242.00	Gemin6	gem (nuclear organelle) associated protein 6	1.42	3.70E-06
11461.00	Actb	actin, beta	1.42	0.029
18140.00	Uhrf1	ubiquitin-like, containing PHD and RING finger domains, 1	1.41	3.20E-06
54563.00	Nup210	nucleoporin 210	1.41	6.50E-06
17110.00	Lyz1	lysozyme 1	1.41	0.00081
15505.00	Hsph1	heat shock 105kDa/110kDa protein 1	1.41	3.50E-05
100102.00	Pcsk9	proprotein convertase subtilisin/kexin type 9	1.4	8.10E-05
67134.00	Nop56	NOP56 ribonucleoprotein homolog (yeast)	1.4	9.40E-05
14961.00	H2-Ab1	histocompatibility 2, class II antigen A, beta 1	1.4	0.0076
227358.00	Fam132b	family with sequence similarity 132, member B	1.4	6.50E-06
110956.00	D17H6S56E-5	DNA segment, Chr 17, human D6S56E 5	1.39	3.50E-05
434341.00	Nlrc5	NLR family, CARD domain containing 5	1.39	0.013
73284.00	Ddit4l	DNA-damage-inducible transcript 4-like	1.38	0.0017
12443.00	Ccnd1	cyclin D1	1.38	3.50E-07
381101.00	BC048355	cDNA sequence BC048355	1.38	1.90E-05
67236.00	Cinp	cyclin-dependent kinase 2 interacting protein	1.38	3.20E-06
52530.00	Nhp2	NHP2 ribonucleoprotein homolog (yeast)	1.37	1.60E-05
105837.00	Mtbp	Mdm2, transformed 3T3 cell double minute p53 binding protein	1.37	6.20E-08
13555.00	E2f1	E2F transcription factor 1	1.37	3.50E-07
70333.00	Cd3eap	CD3E antigen, epsilon polypeptide associated protein	1.37	0.00012
21354.00	Tap1	transporter 1, ATP-binding cassette, sub-family B (MDR/TAP)	1.36	0.0067
	ILMN_187520		1.36	9.60E-05
67671.00	Rpl38	ribosomal protein L38	1.36	0.00025
59028.00	Rcl1	RNA terminal phosphate cyclase-like 1	1.36	4.90E-05
100044103.00	ILMN_210607	PREDICTED: Mus musculus similar to mKIAA1645 protein (LOC100044103), mRNA.	1.36	6.90E-05
12544.00	Cdc45	cell division cycle 45 homolog (S. cerevisiae)	1.36	2.50E-06
14450.00	Gart	phosphoribosylglycinamide formyltransferase	1.35	3.40E-05
22051.00	Trip6	thyroid hormone receptor interactor 6	1.34	8.30E-05
21915.00	Dtymk	deoxythymidylate kinase	1.34	2.00E-04
330671.00	B4galnt4	beta-1,4-N-acetyl-galactosaminyl transferase 4	1.34	1.90E-05
11792.00	Apex1	apurinic/apyrimidinic endonuclease 1	1.34	0.00061
66965.00	ILMN_198892	Mus musculus RIKEN cDNA 2310061F22 gene (2310061F22Rik), mRNA.	1.34	4.60E-07
98170.00	Tmem132a	transmembrane protein 132A	1.33	7.50E-05
98170.00	Tmem132a	transmembrane protein 132A	1.33	2.20E-06
245688.00	ILMN_215499	Mus musculus retinoblastoma binding protein 7 (Rbbp7), mRNA.	1.33	2.00E-04
56361.00	Pus1	pseudouridine synthase 1	1.33	2.30E-06
105837.00	Mtbp	Mdm2, transformed 3T3 cell double minute p53 binding protein	1.33	2.10E-06
14999.00	H2-DMb1	histocompatibility 2, class II, locus Mb1	1.33	0.0048
14038.00	Expi	extracellular proteinase inhibitor	1.33	1.70E-05
109857.00	Cbr3	carbonyl reductase 3	1.33	0.0076
406217.00	ILMN_231589	Mus musculus brain expressed gene 4 (Bex4), mRNA.	1.33	9.60E-06
20641.00	Snrpd1	small nuclear ribonucleoprotein D1	1.32	4.50E-06
56505.00	Ruvbl1	RuvB-like protein 1	1.32	1.50E-06
17304.00	Mfge8	milk fat globule-EGF factor 8 protein	1.32	0.00018
12028.00	Bax	BCL2-associated X protein	1.32	0.00012
56279.00	Fam69b	family with sequence similarity 69, member B	1.32	6.10E-06
21681.00	Thoc4	THO complex 4	1.31	2.10E-05
17427.00	Mns1	meiosis-specific nuclear structural protein 1	1.31	3.30E-06
15361.00	Hmga1	high mobility group AT-hook 1	1.31	0.00044
217995.00	ILMN_214317	Mus musculus HEAT repeat containing 1 (Heatr1), mRNA.	1.31	0.00012
12265.00	Ciita	class II transactivator	1.31	0.002
71449.00	ILMN_221329	Mus musculus RIKEN cDNA 5630401D24 gene (5630401D24Rik), mRNA.	1.31	0.00022
93840.00	Vangl2	vang-like 2 (van gogh, Drosophila)	1.3	4.60E-05
78294.00	Rps27a	ribosomal protein S27A	1.3	1.70E-05

13639.00	Efna4	ephrin A4	1.3	3.50E-05
21983.00	Tpbg	trophoblast glycoprotein	1.29	2.60E-06
21973.00	Top2a	topoisomerase (DNA) II alpha	1.29	0.00026
108037.00	Shmt2	serine hydroxymethyltransferase 2 (mitochondrial)	1.29	6.70E-06
67223.00	Rrp15	ribosomal RNA processing 15 homolog (S. cerevisiae)	1.29	3.40E-05
12443.00	Ccnd1	cyclin D1	1.29	5.40E-05
12153.00	Bmp1	bone morphogenetic protein 1	1.29	2.40E-05
18971.00	Pold1	polymerase (DNA directed), delta 1, catalytic subunit	1.28	4.90E-05
18969.00	Pola2	polymerase (DNA directed), alpha 2	1.28	6.40E-06
27756.00	Lsm2	LSM2 homolog, U6 small nuclear RNA associated (S. cerevisiae)	1.28	3.40E-06
116701.00	ILMN_211983	Mus musculus fibroblast growth factor receptor-like 1 (Fgfrl1), mRNA.	1.28	1.10E-05
214901.00	Chtf18	CTF18, chromosome transmission fidelity factor 18 homolog (S. cerevisiae)	1.28	7.60E-06
72640.00	Mex3a	mex3 homolog A (C. elegans)	1.27	2.40E-06
17357.00	Marcksl1	MARCKS-like 1	1.27	0.00023
110033.00	Kif22	kinesin family member 22	1.27	1.00E-05
110006.00	ILMN_221841	Mus musculus glucuronidase, beta (Gusb), mRNA.	1.27	2.20E-05
14450.00	Gart	phosphoribosylglycinamide formyltransferase	1.27	9.60E-05
104156.00	Etv5	ets variant gene 5	1.27	0.00019
12144.00	Blm	Bloom syndrome, RecQ helicase-like	1.27	1.40E-05
71242.00	Spata24	spermatogenesis associated 24	1.27	7.90E-05
214572.00	Prmt7	protein arginine N-methyltransferase 7	1.26	1.10E-05
53605.00	ILMN_214137	Mus musculus nucleosome assembly protein 1-like 1 (Nap1l1), mRNA.	1.26	1.30E-05
17869.00	Myc	myelocytomatosis oncogene	1.26	0.00011
17110.00	Lyz1	lysozyme 1	1.26	0.0024
17228.00	Cma1	chymase 1, mast cell	1.26	6.50E-05
52276.00	Cdca8	cell division cycle associated 8	1.26	9.30E-06
69928.00	Apitd1	apoptosis-inducing, TAF9-like domain 1	1.26	0.00026
80914.00	Uck2	uridine-cytidine kinase 2	1.25	1.80E-05
104367.00	Snora65	small nucleolar RNA, H/ACA box 65	1.25	4.00E-05
245688.00	ILMN_215499	Mus musculus retinoblastoma binding protein 7 (Rbbp7), mRNA.	1.25	0.0014
68294.00	Mfsd10	major facilitator superfamily domain containing 10	1.25	1.60E-05
	ILMN_208668		1.25	8.90E-06
66570.00	Cenpm	centromere protein M	1.25	3.10E-07
237038.00	Nox1	NADPH oxidase 1	1.24	0.0092
18102.00	ILMN_209549	Mus musculus non-metastatic cells 1, protein (NM23A) expressed in (Nme1), mRNA.	1.24	1.80E-06
108673.00	Ccdc86	coiled-coil domain containing 86	1.24	0.00035
57315.00	Wdr46	WD repeat domain 46	1.23	6.10E-05
72787.00	Tmem48	transmembrane protein 48	1.23	1.20E-05
236539.00	ILMN_226456	Mus musculus 3-phosphoglycerate dehydrogenase (Phgdh), mRNA.	1.23	0.00052
234865.00	Nup133	nucleoporin 133	1.23	1.80E-05
67711.00	Nsmce1	non-SMC element 1 homolog (S. cerevisiae)	1.23	2.00E-04
27993.00	Imp4	IMP4, U3 small nucleolar ribonucleoprotein, homolog (yeast)	1.23	7.10E-06
434632.00	BC085271	cDNA sequence BC085271	1.23	0.00032
56412.00	2610024G14Rik	RIKEN cDNA 2610024G14 gene	1.23	1.80E-05
68926.00	Ubap2	ubiquitin-associated protein 2	1.22	3.00E-06
20174.00	Ruvbl2	RuvB-like protein 2	1.22	3.50E-06
19891.00	Rpa2	replication protein A2	1.22	0.00017
59028.00	Rcl1	RNA terminal phosphate cyclase-like 1	1.22	0.00046
106582.00	Nrm	nurim (nuclear envelope membrane protein)	1.22	2.00E-06
17748.00	Mt1	metallothionein 1	1.22	0.00092
15114.00	Hap1	huntingtin-associated protein 1	1.22	0.00011
66570.00	Cenpm	centromere protein M	1.22	2.10E-06
59053.00	Fam203a	family with sequence similarity 203, member A	1.22	1.00E-05
17299.00	Mettl1	methyltransferase like 1	1.21	0.00049
110006.00	Gusb	glucuronidase, beta	1.21	7.80E-05
14630.00	Gclm	glutamate-cysteine ligase, modifier subunit	1.21	2.70E-06
69639.00	Exosc8	exosome component 8	1.21	1.80E-05
80914.00	Uck2	uridine-cytidine kinase 2	1.2	1.50E-05
110816.00	ILMN_212496	Mus musculus PWP2 periodic tryptophan protein homolog (yeast) (Pwp2), mRNA.	1.2	0.00013
654467.00	ILMN_234090	Mus musculus heterogeneous nuclear ribonucleoprotein A1 pseudogene (LOC654467) on chromosome 9.	1.2	0.00031
209737.00	ILMN_212864	Mus musculus kinesin family member 15 (Kif15), mRNA.	1.2	6.50E-06
272359.00	Irf2bp1	interferon regulatory factor 2 binding protein 1	1.2	1.30E-05
14916.00	Guca2b	guanylate cyclase activator 2b (retina)	1.2	0.00029
381903.00	Alg8	asparagine-linked glycosylation 8 homolog (yeast, alpha-1,3-glucosyltransferase)	1.2	5.60E-06
104732.00	4930427A07Rik	RIKEN cDNA 4930427A07 gene	1.2	0.00013
72657.00	2700094K13Rik	RIKEN cDNA 2700094K13 gene	1.2	1.60E-05
57257.00	Vav3	vav 3 oncogene	1.19	2.40E-06
20133.00	ILMN_231868	Mus musculus ribonucleotide reductase M1 (Rrm1), mRNA.	1.19	8.90E-05

245688.00	ILMN_215499	Mus musculus retinoblastoma binding protein 7 (Rbbp7), mRNA.	1.19	6.00E-04
226519.00	ILMN_232528	Mus musculus laminin, gamma 1 (Lamc1), mRNA.	1.19	0.00037
23834.00	Cdc6	cell division cycle 6 homolog (S. cerevisiae)	1.19	4.80E-06
69719.00	Cad	carbamoyl-phosphate synthetase 2, aspartate transcarbamylase, and dihydroorotase	1.19	0.00035
14025.00	Bcl11a	B-cell CLL/lymphoma 11A (zinc finger protein)	1.19	3.80E-05
74254.00	Gpn1	GPN-loop GTPase 1	1.18	5.50E-05
22256.00	Ung	uracil DNA glycosylase	1.18	1.00E-04
21849.00	Trim28	tripartite motif-containing 28	1.18	2.20E-05
75273.00	Pelp1	proline, glutamic acid and leucine rich protein 1	1.18	0.00014
70572.00	Ipo5	importin 5	1.18	2.90E-06
15312.00	Hmgn1	high mobility group nucleosomal binding domain 1	1.18	1.10E-05
20624.00	Eftud2	elongation factor Tu GTP binding domain containing 2	1.18	1.00E-05
27407.00	Abcf2	ATP-binding cassette, sub-family F (GCN20), member 2	1.18	5.70E-06
56412.00	2610024G14Rik	RIKEN cDNA 2610024G14 gene	1.18	1.30E-05
21781.00	Tfdp1	transcription factor Dp 1	1.17	1.50E-05
27966.00	Rrp9	RRP9, small subunit (SSU) processome component, homolog (yeast)	1.17	0.00013
72151.00	Rfc5	replication factor C (activator 1) 5	1.17	1.10E-06
57785.00	Rangrf	RAN guanine nucleotide release factor	1.17	6.20E-05
19679.00	Pitpnm2	phosphatidylinositol transfer protein, membrane-associated 2	1.17	6.10E-05
17975.00	Ncl	nucleolin	1.17	6.80E-06
16881.00	Lig1	ligase I, DNA, ATP-dependent	1.17	4.20E-05
23886.00	Gdf15	growth differentiation factor 15	1.17	0.042
55932.00	Gbp3	guanylate binding protein 3	1.17	0.019
434858.00	ILMN_233946	Mus musculus predicted gene, EG434858 (EG434858), non-coding RNA.	1.17	0.0046
72082.00	Cyp2c55	cytochrome P450, family 2, subfamily c, polypeptide 55	1.17	0.026
107995.00	Cdc20	cell division cycle 20 homolog (S. cerevisiae)	1.17	0.00023
230991.00	B930041F14Rik	RIKEN cDNA B930041F14 gene	1.17	1.80E-05
71242.00	Spata24	spermatogenesis associated 24	1.17	5.10E-06
28035.00	Usp39	ubiquitin specific peptidase 39	1.16	9.60E-06
63959.00	Slc29a1	solute carrier family 29 (nucleoside transporters), member 1	1.16	0.00045
67824.00	Nmral1	NmrA-like family domain containing 1	1.16	9.60E-06
60441.00	Mrpl38	mitochondrial ribosomal protein L38	1.16	6.70E-05
12462.00	Cct3	chaperonin containing Tcp1, subunit 3 (gamma)	1.16	3.50E-05
330050.00	Fam185a	family with sequence similarity 185, member A	1.16	4.50E-06
22294.00	Uxt	ubiquitously expressed transcript	1.15	8.00E-06
19245.00	Ptp4a3	protein tyrosine phosphatase 4a3	1.15	0.0011
215387.00	Ncaph	non-SMC condensin I complex, subunit H	1.15	0.00052
17938.00	Naca	nascent polypeptide-associated complex alpha polypeptide	1.15	2.10E-06
66973.00	Mrps18b	mitochondrial ribosomal protein S18B	1.15	1.00E-05
	ILMN_199160		1.15	0.0044
16201.00	Ilf3	interleukin enhancer binding factor 3	1.15	3.60E-06
13433.00	Dnmt1	DNA methyltransferase (cytosine-5) 1	1.15	0.00012
18117.00	Cox4nb	COX4 neighbor	1.15	7.60E-06
12740.00	Cldn4	claudin 4	1.15	0.0016
102920.00	Cenpi	centromere protein I	1.15	0.00071
107995.00	Cdc20	cell division cycle 20 homolog (S. cerevisiae)	1.15	0.00046
12181.00	Bop1	block of proliferation 1	1.15	0.00014
23825.00	Banf1	barrier to autointegration factor 1	1.15	9.80E-05
56412.00	2610024G14Rik	RIKEN cDNA 2610024G14 gene	1.15	8.00E-06
21844.00	Tiam1	T-cell lymphoma invasion and metastasis 1	1.14	1.90E-05
67390.00	Rnmtl1	RNA methyltransferase like 1	1.14	6.90E-05
78929.00	ILMN_209245	Mus musculus polymerase (RNA) III (DNA directed) polypeptide H (Polr3h), mRNA.	1.14	9.60E-06
66667.00	Hspbap1	Hspb associated protein 1	1.14	4.00E-05
55927.00	Hes6	hairy and enhancer of split 6 (Drosophila)	1.14	7.60E-06
14776.00	Gpx2	glutathione peroxidase 2	1.14	1.10E-05
14114.00	Fbln1	fibulin 1	1.14	0.00044
76267.00	Fads1	fatty acid desaturase 1	1.14	4.20E-05
71919.00	Rpap3	RNA polymerase II associated protein 3	1.14	0.00011
66912.00	Bzw2	basic leucine zipper and W2 domains 2	1.14	0.00012
11799.00	Birc5	baculoviral IAP repeat-containing 5	1.14	2.00E-05
66131.00	Tipin	timeless interacting protein	1.13	6.10E-05
225608.00	Sh3tc2	SH3 domain and tetratricopeptide repeats 2	1.13	0.00034
20135.00	ILMN_225236	Mus musculus ribonucleotide reductase M2 (Rrm2), mRNA.	1.13	0.00016
26564.00	Ror2	receptor tyrosine kinase-like orphan receptor 2	1.13	0.00058
14208.00	Ppm1g	protein phosphatase 1G (formerly 2C), magnesium-dependent, gamma isoform	1.13	4.00E-05
18972.00	Pold2	polymerase (DNA directed), delta 2, regulatory subunit	1.13	6.60E-06
18221.00	Nudc	nuclear distribution gene C homolog (Aspergillus)	1.13	3.50E-06
18221.00	Nudc	nuclear distribution gene C homolog (Aspergillus)	1.13	0.00019

50927.00	ILMN_211691	Mus musculus nuclear autoantigenic sperm protein (histone-binding) (Nasp), transcript variant 2, mRNA.	1.13	5.50E-05
70082.00	Lysmd2	LysM, putative peptidoglycan-binding, domain containing 2	1.13	0.0014
100047155.00	ILMN_221233	PREDICTED: Mus musculus similar to Small nuclear ribonucleoprotein polypeptide A (LOC100047155), mRNA.	1.13	7.00E-06
100042777.00	ILMN_212781	PREDICTED: Mus musculus similar to human protein homologous to DROER protein (LOC100042777), mRNA.	1.13	2.80E-06
69550.00	Bst2	bone marrow stromal cell antigen 2	1.13	6.20E-05
12144.00	Blm	Bloom syndrome, RecQ helicase-like	1.13	2.10E-05
11799.00	Birc5	baculoviral IAP repeat-containing 5	1.13	0.00037
406217.00	Bex4	brain expressed gene 4	1.13	0.0076
66422.00	Dctpp1	dCTP pyrophosphatase 1	1.13	0.00014
107071.00	Wdr74	WD repeat domain 74	1.12	6.10E-05
66409.00	Rsl1d1	ribosomal L1 domain containing 1	1.12	1.90E-05
28000.00	Prpf19	PRP19/PSO4 pre-mRNA processing factor 19 homolog (S. cerevisiae)	1.12	1.70E-05
11545.00	Parp1	poly (ADP-ribose) polymerase family, member 1	1.12	5.40E-05
677205.00	ILMN_212422	PREDICTED: Mus musculus similar to DEAD (Asp-Glu-Ala-Asp) box polypeptide 18 (LOC677205), misc RNA.	1.12	7.60E-06
105988.00	Espl1	extra spindle poles-like 1 (S. cerevisiae)	1.12	2.20E-05
103551.00	E130012A19Rik	RIKEN cDNA E130012A19 gene	1.12	0.0033
108888.00	Atad3a	ATPase family, AAA domain containing 3A	1.12	4.90E-05
27407.00	Abcf2	ATP-binding cassette, sub-family F (GCN20), member 2	1.12	2.00E-06
22294.00	Uxt	ubiquitously expressed transcript	1.11	3.80E-06
22143.00	Tuba1b	tubulin, alpha 1B	1.11	4.00E-05
268396.00	Sh3pxd2b	SH3 and PX domains 2B	1.11	6.90E-05
100088.00	Rcc1	regulator of chromosome condensation 1	1.11	3.50E-05
71974.00	Prmt3	protein arginine N-methyltransferase 3	1.11	2.10E-05
100043257.00	ILMN_215552	PREDICTED: Mus musculus similar to RNA binding motif protein 3 (LOC100043257), mRNA.	1.11	4.50E-06
57905.00	Isy1	ISY1 splicing factor homolog (S. cerevisiae)	1.11	8.50E-06
93695.00	Gpnmb	glycoprotein (transmembrane) nmb	1.11	0.027
227715.00	Exosc2	exosome component 2	1.11	0.00012
71963.00	Cdca4	cell division cycle associated 4	1.11	1.40E-05
11799.00	Birc5	baculoviral IAP repeat-containing 5	1.11	8.90E-05
170759.00	Atp13a1	ATPase type 13A1	1.11	0.00094
21817.00	Tgm2	transglutaminase 2, C polypeptide	1.1	0.0012
214572.00	Prmt7	protein arginine N-methyltransferase 7	1.1	3.50E-05
27374.00	ILMN_257867	Mus musculus protein arginine N-methyltransferase 5 (Prmt5), mRNA.	1.1	0.00032
56031.00	Ppie	peptidylprolyl isomerase E (cyclophilin E)	1.1	4.40E-05
211548.00	Nomo1	nodal modulator 1	1.1	1.40E-05
97961.00	Nol12	nucleolar protein 12	1.1	2.60E-05
110109.00	Nop2	NOP2 nucleolar protein homolog (yeast)	1.1	0.00034
66978.00	Luc7l	Luc7 homolog (S. cerevisiae)-like	1.1	6.50E-05
55944.00	Eif3d	eukaryotic translation initiation factor 3, subunit D	1.1	0.00014
107995.00	Cdc20	cell division cycle 20 homolog (S. cerevisiae)	1.1	0.00011
71449.00	ILMN_221329	Mus musculus RIKEN cDNA 5630401D24 gene (5630401D24Rik), mRNA.	1.1	8.10E-05
68026.00	2810417H13Rik	RIKEN cDNA 2810417H13 gene	1.1	0.002
232679.00	Zc3hc1	zinc finger, C3HC type 1	1.09	6.70E-05
230734.00	Yrdc	yrdC domain containing (E.coli)	1.09	0.00022
72171.00	Shq1	SHQ1 homolog (S. cerevisiae)	1.09	6.50E-06
107094.00	Rrp12	ribosomal RNA processing 12 homolog (S. cerevisiae)	1.09	7.80E-05
20085.00	Rps19	ribosomal protein S19	1.09	5.40E-05
27374.00	Prmt5	protein arginine N-methyltransferase 5	1.09	4.90E-05
27374.00	Prmt5	protein arginine N-methyltransferase 5	1.09	1.10E-05
56452.00	Orc6	origin recognition complex, subunit 6	1.09	3.90E-06
68294.00	Mfsd10	major facilitator superfamily domain containing 10	1.09	1.10E-05
17083.00	Tmed1	transmembrane emp24 domain containing 1	1.09	5.80E-05
22379.00	Fmnl3	formin-like 3	1.09	0.00023
13877.00	ILMN_212781	Mus musculus enhancer of rudimentary homolog (Drosophila) (Erh), mRNA.	1.09	1.40E-05
68087.00	ILMN_218673	Mus musculus dephospho-CoA kinase domain containing (Dcakd), mRNA.	1.09	1.90E-06
27407.00	Abcf2	ATP-binding cassette, sub-family F (GCN20), member 2	1.09	0.00017
74111.00	Rbm19	RNA binding motif protein 19	1.08	0.00083
240334.00	Pcyox1l	prenylcysteine oxidase 1 like	1.08	5.00E-04
66164.00	Nip7	nuclear import 7 homolog (S. cerevisiae)	1.08	2.30E-05
13849.00	Ephx1	epoxide hydrolase 1, microsomal	1.08	0.0055
13401.00	Dmwd	dystrophia myotonica-containing WD repeat motif	1.08	1.40E-05
27407.00	Abcf2	ATP-binding cassette, sub-family F (GCN20), member 2	1.08	0.00018
72155.00	Cenpn	centromere protein N	1.08	1.10E-05
66976.00	ILMN_212730	Mus musculus RIKEN cDNA 2410002F23 gene (2410002F23Rik), mRNA.	1.08	0.00025
68971.00	1500001M20Rik	RIKEN cDNA 1500001M20 gene	1.08	0.00014
22146.00	Tuba1c	tubulin, alpha 1C	1.07	0.00034
22021.00	Tpst1	protein-tyrosine sulfotransferase 1	1.07	7.90E-05
52033.00	Pbk	PDZ binding kinase	1.07	6.10E-05

26425.00	Nubp1	nucleotide binding protein 1	1.07	9.30E-06
17219.00	Mcm6	minichromosome maintenance deficient 6 (MIS5 homolog, S. pombe) (S. cerevisiae)	1.07	2.30E-06
100047856.00	ILMN_208813	PREDICTED: Mus musculus similar to calponin 3, acidic (LOC100047856), mRNA.	1.07	0.00036
15510.00	ILMN_213620	Mus musculus heat shock protein 1 (chaperonin) (Hspd1), mRNA.	1.07	0.016
14859.00	Gsta3	glutathione S-transferase, alpha 3	1.07	0.0029
20624.00	Eftud2	elongation factor Tu GTP binding domain containing 2	1.07	7.60E-06
50496.00	ILMN_220573	Mus musculus E2F transcription factor 6 (E2f6), mRNA.	1.07	2.50E-05
12387.00	ILMN_211511	Mus musculus catenin (cadherin associated protein), beta 1 (Ctnnb1), mRNA.	1.07	0.0018
107373.00	Fam111a	family with sequence similarity 111, member A	1.07	4.50E-06
27366.00	Txnl4a	thioredoxin-like 4A	1.06	2.80E-05
30056.00	Timm9	translocase of inner mitochondrial membrane 9 homolog (yeast)	1.06	7.10E-06
66628.00	Thg1l	tRNA-histidine guanylyltransferase 1-like (S. cerevisiae)	1.06	0.00015
11545.00	Parp1	poly (ADP-ribose) polymerase family, member 1	1.06	9.60E-06
56488.00	Nxt1	NTF2-related export protein 1	1.06	1.10E-05
234865.00	Nup133	nucleoporin 133	1.06	0.002
52683.00	Ncaph2	non-SMC condensin II complex, subunit H2	1.06	6.50E-06
16206.00	Lrig1	leucine-rich repeats and immunoglobulin-like domains 1	1.06	4.20E-06
76113.00	Lpo	lactoperoxidase	1.06	1.10E-05
16906.00	ILMN_220293	Mus musculus lamin B1 (Lmnb1), mRNA.	1.06	4.00E-05
80876.00	Ifitm2	interferon induced transmembrane protein 2	1.06	0.00076
66583.00	Exosc1	exosome component 1	1.06	4.60E-05
67102.00	D16Ertd472e	DNA segment, Chr 16, ERATO Doi 472, expressed	1.06	0.0011
21771.00	Cirh1a	cirrhosis, autosomal recessive 1A (human)	1.06	0.00013
67849.00	Cdca5	cell division cycle associated 5	1.06	0.00037
12462.00	Cct3	chaperonin containing Tcp1, subunit 3 (gamma)	1.06	8.30E-05
27081.00	Zfp275	zinc finger protein 275	1.05	0.00027
71354.00	Wdr31	WD repeat domain 31	1.05	4.00E-04
54141.00	Spag5	sperm associated antigen 5	1.05	6.50E-06
107686.00	Snrpd2	small nuclear ribonucleoprotein D2	1.05	3.50E-05
20587.00	Smarcb1	SWI/SNF related, matrix associated, actin dependent regulator of chromatin, subfamily b, member 1	1.05	2.00E-04
55963.00	Slc1a4	solute carrier family 1 (glutamate/neutral amino acid transporter), member 4	1.05	0.0015
28000.00	Prpf19	PRP19/PSO4 pre-mRNA processing factor 19 homolog (S. cerevisiae)	1.05	0.0012
28000.00	Prpf19	PRP19/PSO4 pre-mRNA processing factor 19 homolog (S. cerevisiae)	1.05	0.00015
67037.00	Pmf1	polyamine-modulated factor 1	1.05	4.20E-05
110109.00	Nop2	NOP2 nucleolar protein homolog (yeast)	1.05	9.40E-05
17975.00	Ncl	nucleolin	1.05	6.00E-05
108156.00	Mthfd1	methylenetetrahydrofolate dehydrogenase (NADP+ dependent), methenyltetrahydrofolate cyclohydrolase, formyltetrahydrofolate synthase	1.05	0.00037
17535.00	Mre11a	meiotic recombination 11 homolog A (S. cerevisiae)	1.05	1.80E-05
15191.00	Hdgf	hepatoma-derived growth factor	1.05	4.00E-05
232680.00	Cpa2	carboxypeptidase A2, pancreatic	1.05	6.00E-05
71963.00	Cdca4	cell division cycle associated 4	1.05	1.10E-05
71963.00	Cdca4	cell division cycle associated 4	1.05	5.80E-06
12443.00	Ccnd1	cyclin D1	1.05	0.00023
71735.00	Lrwd1	leucine-rich repeats and WD repeat domain containing 1	1.05	1.10E-05
15547.00	Trmt2a	TRM2 tRNA methyltransferase 2 homolog A (S. cerevisiae)	1.04	6.20E-05
21681.00	Thoc4	THO complex 4	1.04	2.30E-05
20425.00	Shmt1	serine hydroxymethyltransferase 1 (soluble)	1.04	0.00085
19361.00	Rad51	RAD51 homolog (S. cerevisiae)	1.04	0.0061
18637.00	Pfdn2	prefoldin 2	1.04	1.60E-05
52683.00	Ncaph2	non-SMC condensin II complex, subunit H2	1.04	1.30E-05
66902.00	Mtap	methylthioadenosine phosphorylase	1.04	3.00E-04
224092.00	Lsg1	large subunit GTPase 1 homolog (S. cerevisiae)	1.04	8.90E-06
192170.00	Eif4a3	eukaryotic translation initiation factor 4A3	1.04	0.00039
74747.00	Ddit4	DNA-damage-inducible transcript 4	1.04	0.0011
12236.00	Bub1b	budding uninhibited by benzimidazoles 1 homolog, beta (S. cerevisiae)	1.04	0.00036
27078.00	B9d1	B9 protein domain 1	1.04	5.10E-05
17025.00	ILMN_215056	Mus musculus aminolevulinate, delta-, dehydratase (Alad), mRNA.	1.04	7.50E-05
217737.00	Ahsa1	AHA1, activator of heat shock protein ATPase homolog 1 (yeast)	1.04	6.80E-06
66976.00	ILMN_212730	Mus musculus RIKEN cDNA 2410002F23 gene (2410002F23Rik), mRNA.	1.04	0.00045
68964.00	1500010J02Rik	RIKEN cDNA 1500010J02 gene	1.04	1.10E-06
57315.00	Wdr46	WD repeat domain 46	1.03	0.00013
21926.00	Tnf	tumor necrosis factor	1.03	0.0064
30056.00	Timm9	translocase of inner mitochondrial membrane 9 homolog (yeast)	1.03	1.60E-05
116914.00	Slc19a2	solute carrier family 19 (thiamine transporter), member 2	1.03	0.0034
214791.00	Sertad4	SERTA domain containing 4	1.03	1.00E-04
68275.00	Rpa1	replication protein A1	1.03	3.00E-06
19227.00	Pthlh	parathyroid hormone-like peptide	1.03	6.60E-06
93737.00	Pard6g	par-6 partitioning defective 6 homolog gamma (C. elegans)	1.03	2.10E-05

100609.00	Nsun5	NOL1/NOP2/Sun domain family, member 5	1.03	2.90E-05
56150.00	Mad2l1	MAD2 mitotic arrest deficient-like 1 (yeast)	1.03	1.70E-06
72500.00	ILMN_225633	Mus musculus immediate early response 5-like (Ier5l), mRNA.	1.03	9.30E-06
319170.00	Hist1h2an	histone cluster 1, H2an	1.03	0.012
276770.00	Eif5a	eukaryotic translation initiation factor 5A	1.03	4.00E-05
230917.00	Tmem201	transmembrane protein 201	1.02	2.80E-05
66525.00	Timm50	translocase of inner mitochondrial membrane 50 homolog (yeast)	1.02	2.00E-04
56403.00	Syncrip	synaptotagmin binding, cytoplasmic RNA interacting protein	1.02	4.40E-05
66506.00	Psmg3	proteasome (prosome, macropain) assembly chaperone 3	1.02	1.30E-05
20019.00	Polr1a	polymerase (RNA) I polypeptide A	1.02	4.90E-05
69912.00	Nup43	nucleoporin 43	1.02	4.50E-06
216443.00	Mars	methionine-tRNA synthetase	1.02	0.00092
319169.00	Hist1h2ak	histone cluster 1, H2ak	1.02	0.0081
107435.00	Hat1	histone aminotransferase 1	1.02	2.60E-05
14297.00	Fxn	frataxin	1.02	2.80E-05
20624.00	Eftud2	elongation factor Tu GTP binding domain containing 2	1.02	9.60E-06
228889.00	Ddx27	DEAD (Asp-Glu-Ala-Asp) box polypeptide 27	1.02	2.80E-05
108912.00	Cdca2	cell division cycle associated 2	1.02	7.90E-05
76813.00	ILMN_230074	Mus musculus armadillo repeat containing 6 (Armc6), mRNA.	1.02	0.00014
11564.00	Adsl	adenylosuccinate lyase	1.02	0.00012
72061.00	2010111I01Rik	RIKEN cDNA 2010111I01 gene	1.02	1.20E-05
21877.00	Tk1	thymidine kinase 1	1.01	0.0012
20621.00	Snn	stannin	1.01	0.0026
435684.00	ILMN_245068	Mus musculus Src homology 2 domain containing F (Shf), mRNA.	1.01	0.0033
16912.00	Psmb9	proteasome (prosome, macropain) subunit, beta type 9 (large multifunctional peptidase 2)	1.01	0.04
445007.00	Nup85	nucleoporin 85	1.01	0.0057
217011.00	ILMN_239279	Mus musculus notchless homolog 1 (Drosophila) (Nle1), mRNA.	1.01	0.0017
66973.00	ILMN_252778	Mus musculus mitochondrial ribosomal protein S18B (Mrps18b), nuclear gene encoding mitochondrial protein, mRNA.	1.01	0.00035
14793.00	Cdca3	cell division cycle associated 3	1.01	0.00055
215193.00	Diexf	digestive organ expansion factor homolog (zebrafish)	1.01	5.50E-05
319278.00	A230050P20Rik	RIKEN cDNA A230050P20 gene	1.01	0.0013
66356.00	2310008H09Rik	RIKEN cDNA 2310008H09 gene	1.01	6.60E-06
22793.00	Zyx	zyxin	1	0.0011
232187.00	Smyd5	SET and MYND domain containing 5	1	0.00095
19355.00	Rad1	RAD1 homolog (S. pombe)	1	1.00E-04
214572.00	Prmt7	protein arginine N-methyltransferase 7	1	1.90E-05
68106.00	Nt5c3l	5'-nucleotidase, cytosolic III-like	1	0.00013
16319.00	Incenp	inner centromere protein	1	0.00032
55927.00	Hes6	hairy and enhancer of split 6 (Drosophila)	1	0.00015
29870.00	Gtse1	G two S phase expressed protein 1	1	0.002
67112.00	Fgf22	fibroblast growth factor 22	1	0.00015
69524.00	Esam	endothelial cell-specific adhesion molecule	1	0.00018
27979.00	Eif3b	eukaryotic translation initiation factor 3, subunit B	1	0.00026
381903.00	Alg8	asparagine-linked glycosylation 8 homolog (yeast, alpha-1,3-glucosyltransferase)	1	0.00079
223921.00	Aaas	achalasia, adrenocortical insufficiency, alacrimia	1	0.00036
277414.00	Trp53i11	transformation related protein 53 inducible protein 11	-1	0.00048
229731.00	Slc25a24	solute carrier family 25 (mitochondrial carrier, phosphate carrier), member 24	-1	0.00054
67874.00	Rprm	reprimo, TP53 dependent G2 arrest mediator candidate	-1	0.00025
100047353.00	ILMN_212740	PREDICTED: Mus musculus similar to myocardial vascular inhibition factor (LOC100047353), mRNA.	-1	0.00029
100047173.00	ILMN_214714	PREDICTED: Mus musculus similar to synaptotagmin-like 1 (LOC100047173), misc RNA.	-1	1.60E-05
16196.00	Il7	interleukin 7	-1	0.002
93842.00	Igsf9	immunoglobulin superfamily, member 9	-1	0.0012
15484.00	Hsd11b2	hydroxysteroid 11-beta dehydrogenase 2	-1	0.013
14674.00	Gna13	guanine nucleotide binding protein, alpha 13	-1	0.0047
14367.00	Fzd5	frizzled homolog 5 (Drosophila)	-1	0.00015
105387.00	Akr1c14	aldo-keto reductase family 1, member C14	-1	5.40E-05
11522.00	Adh1	alcohol dehydrogenase 1 (class I)	-1	0.016
53330.00	Vamp4	vesicle-associated membrane protein 4	-1.01	2.90E-05
223697.00	Sun2	Sad1 and UNC84 domain containing 2	-1.01	0.0037
20482.00	Skil	SKI-like	-1.01	0.0063
224860.00	Plcl2	phospholipase C-like 2	-1.01	1.90E-05
224938.00	Pja2	praja 2, RING-H2 motif containing	-1.01	0.00017
102103.00	Mtus1	mitochondrial tumor suppressor 1	-1.01	0.00014
64095.00	Gpr35	G protein-coupled receptor 35	-1.01	0.003
71946.00	Endod1	endonuclease domain containing 1	-1.01	1.90E-05
171168.00	Acer1	alkaline ceramidase 1	-1.01	0.00046
208117.00	Aph1b	anterior pharynx defective 1b homolog (C. elegans)	-1.01	0.0053
66395.00	Ahnak	AHNAK nucleoprotein (desmoyokin)	-1.01	0.00061

18400.00	Slc22a18	solute carrier family 22 (organic cation transporter), member 18	-1.02	1.10E-05
24057.00	Sh3yl1	Sh3 domain YSC-like 1	-1.02	1.50E-05
71601.00	ILMN_213888	Mus musculus CEA-related cell adhesion molecule 20 (Ceacam20), mRNA.	-1.02	0.0049
11364.00	Acadm	acyl-Coenzyme A dehydrogenase, medium chain	-1.02	1.30E-06
98267.00	Stk17b	serine/threonine kinase 17b (apoptosis-inducing)	-1.03	0.0011
216233.00	Socs2	suppressor of cytokine signaling 2	-1.03	0.0015
69693.00	Pof1b	premature ovarian failure 1B	-1.03	3.80E-05
217166.00	Nr1d1	nuclear receptor subfamily 1, group D, member 1	-1.03	0.0019
83965.00	Enpp5	ectonucleotide pyrophosphatase/phosphodiesterase 5	-1.03	3.80E-05
170752.00	Bco2	beta-carotene oxygenase 2	-1.03	0.0047
73910.00	Arhgap18	Rho GTPase activating protein 18	-1.03	0.0011
11735.00	Ank3	ankyrin 3, epithelial	-1.03	0.0033
104776.00	Aldh6a1	aldehyde dehydrogenase family 6, subfamily A1	-1.03	0.0018
239559.00	A4galt	alpha 1,4-galactosyltransferase	-1.03	2.40E-07
66753.00	Erlec1	endoplasmic reticulum lectin 1	-1.03	3.00E-05
67171.00	Dram2	VDNA-damage regulated autophagy modulator 2	-1.04	0.00019
56374.00	Tmem59	transmembrane protein 59	-1.04	0.00014
233724.00	Tmem41b	transmembrane protein 41B	-1.04	0.0011
20356.00	Sema5a	sema domain, seven thrombospondin repeats (type 1 and type 1-like), transmembrane domain (TM) and short cytoplasmic domain, (semaphorin) 5A	-1.04	0.0015
65970.00	Lima1	LIM domain and actin binding 1	-1.04	0.0053
66809.00	Krt20	keratin 20	-1.04	0.007
319190.00	ILMN_213471	Mus musculus histone cluster 2, H2be (Hist2h2be), mRNA.	-1.04	8.00E-04
74155.00	Errfi1	ERBB receptor feedback inhibitor 1	-1.04	0.00035
66273.00	1810020D17Rik	RIKEN cDNA 1810020D17 gene	-1.04	0.00023
100727.00	Ugt2b34	UDP glucuronosyltransferase 2 family, polypeptide B34	-1.05	0.0087
67043.00	Syap1	synapse associated protein 1	-1.05	0.00011
217463.00	Snx13	sorting nexin 13	-1.05	0.0016
19885.00	Rorc	RAR-related orphan receptor gamma	-1.05	0.018
245867.00	Pcmtd2	protein-L-isoaspartate (D-aspartate) O-methyltransferase domain containing 2	-1.05	0.00071
26424.00	Nr5a2	nuclear receptor subfamily 5, group A, member 2	-1.05	0.00036
223646.00	Naprt1	nicotinate phosphoribosyltransferase domain containing 1	-1.05	0.0062
100046056.00	ILMN_192265	PREDICTED: Mus musculus similar to Pre-B-cell leukemia transcription factor interacting protein 1 (LOC100046056), mRNA.	-1.05	0.005
75686.00	Nudt16	nudix (nucleoside diphosphate linked moiety X)-type motif 16	-1.05	0.00011
319186.00	Hist1h2bm	histone cluster 1, H2bm	-1.05	0.00016
13360.00	Dhcr7	7-dehydrocholesterol reductase	-1.05	0.0017
13063.00	Cycs	cytochrome c, somatic	-1.05	9.00E-05
66813.00	ILMN_244180	Mus musculus Bcl2-like 14 (apoptosis facilitator) (Bcl2l14), mRNA.	-1.05	0.0016
223631.00	ILMN_209571	Mus musculus cDNA sequence BC025446 (BC025446), mRNA.	-1.05	0.0056
17940.00	Naip1	NLR family, apoptosis inhibitory protein 1	-1.06	0.0013
100047937.00	ILMN_219033	PREDICTED: Mus musculus similar to Aldehyde dehydrogenase 1 family, member L1 (LOC100047937), mRNA.	-1.06	4.60E-05
13105.00	Cyp2d9	cytochrome P450, family 2, subfamily d, polypeptide 9	-1.06	0.0092
104086.00	Cyp27a1	cytochrome P450, family 27, subfamily a, polypeptide 1	-1.06	0.0034
83429.00	Ctns	cystinosis, nephropathic	-1.06	0.00056
71908.00	Cldn23	claudin 23	-1.06	4.20E-05
12724.00	Clcn2	chloride channel 2	-1.06	1.00E-05
107747.00	ILMN_219033	Mus musculus aldehyde dehydrogenase 1 family, member L1 (Aldh1l1), mRNA.	-1.06	0.00011
66753.00	Erlec1	endoplasmic reticulum lectin 1	-1.06	9.60E-06
94224.00	Srd5a2	steroid 5 alpha-reductase 2	-1.07	1.40E-05
329416.00	Nostrin	nitric oxide synthase trafficker	-1.07	0.00092
17961.00	Nat2	N-acetyltransferase 2 (arylamine N-acetyltransferase)	-1.07	0.00029
99663.00	Clca6	chloride channel calcium activated 6	-1.07	0.025
26365.00	Ceacam1	carcinoembryonic antigen-related cell adhesion molecule 1	-1.07	0.02
353170.00	ILMN_247686	Mus musculus RIKEN cDNA 4932441K18 gene (4932441K18Rik), mRNA.	-1.07	4.20E-05
22248.00	Unc119	unc-119 homolog (C. elegans)	-1.08	8.90E-06
54683.00	Prdx5	peroxiredoxin 5	-1.08	0.00049
67801.00	Pllp	plasma membrane proteolipid	-1.08	0.0035
226971.00	Plekhb2	pleckstrin homology domain containing, family B (evectins) member 2	-1.08	2.30E-05
54484.00	Mkrn1	makorin, ring finger protein, 1	-1.08	0.00095
100046781.00	ILMN_220554	PREDICTED: Mus musculus similar to carboxypeptidase D (LOC100046781), mRNA.	-1.08	0.014
85308.00	Fam158a	family with sequence similarity 158, member A	-1.08	0.0011
13511.00	Dsg2	desmoglein 2	-1.08	0.00029
68778.00	1110038D17Rik	RIKEN cDNA 1110038D17 gene	-1.08	6.30E-07
225997.00	Trpm6	transient receptor potential cation channel, subfamily M, member 6	-1.09	0.00014
72948.00	Tppp	tubulin polymerization promoting protein	-1.09	3.50E-05
19241.00	ILMN_196070	Mus musculus thymosin, beta 4, X chromosome (Tmsb4x), mRNA.	-1.09	1.70E-05
20866.00	ILMN_221367	Mus musculus stromal interaction molecule 1 (Stim1), mRNA.	-1.09	2.50E-06
102693.00	Phldb1	pleckstrin homology-like domain, family B, member 1	-1.09	0.0019
18627.00	Per2	period homolog 2 (Drosophila)	-1.09	0.0027

54405.00	Ndufa1	NADH dehydrogenase (ubiquinone) 1 alpha subcomplex, 1	-1.09	0.00016
105559.00	Mbnl2	muscleblind-like 2	-1.09	4.40E-05
100048721.00	ILMN_210479	PREDICTED: Mus musculus similar to fibronectin leucine rich transmembrane protein 3, transcript variant 1 (LOC100048721), mRNA.	-1.09	0.00074
57890.00	Il17re	interleukin 17 receptor E	-1.09	0.0014
212070.00	Clrn3	clarin 3	-1.09	4.00E-04
76960.00	Bcas1	breast carcinoma amplified sequence 1	-1.09	2.80E-05
21934.00	Tnfrsf11a	tumor necrosis factor receptor superfamily, member 11a	-1.1	1.70E-05
21416.00	Tcf7l2	transcription factor 7-like 2, T-cell specific, HMG-box	-1.1	1.50E-05
54381.00	Pgcp	plasma glutamate carboxypeptidase	-1.1	0.0011
170761.00	Pdzd3	PDZ domain containing 3	-1.1	0.0026
18003.00	Nedd9	neural precursor cell expressed, developmentally down-regulated gene 9	-1.1	0.00059
26931.00	Ppp2r5c	protein phosphatase 2, regulatory subunit B (B56), gamma isoform	-1.1	0.00012
83379.00	Klb	klotho beta	-1.1	2.60E-06
68024.00	Hist1h2bc	histone cluster 1, H2bc	-1.1	0.00092
23882.00	Gadd45g	growth arrest and DNA-damage-inducible 45 gamma	-1.1	0.0017
11732.00	Ank	progressive ankylosis	-1.1	0.00037
107652.00	Uap1	UDP-N-acetylglucosamine pyrophosphorylase 1	-1.11	0.002
11891.00	Rab27a	RAB27A, member RAS oncogene family	-1.11	6.60E-06
213522.00	Plekhg6	pleckstrin homology domain containing, family G (with RhoGef domain) member 6	-1.11	0.00082
53880.00	ILMN_230036	Mus musculus NLR family, apoptosis inhibitory protein 7 (Naip7), mRNA.	-1.11	0.00011
16664.00	Krt14	keratin 14	-1.11	0.013
102871.00	D330045A20Rik	RIKEN cDNA D330045A20 gene	-1.11	0.00035
75415.00	Arhgap12	Rho GTPase activating protein 12	-1.11	5.10E-05
71874.00	2310007B03Rik	RIKEN cDNA 2310007B03 gene	-1.11	2.00E-04
20394.00	Scg5	secretogranin V	-1.12	6.10E-05
11852.00	Rhob	ras homolog gene family, member B	-1.12	0.00047
18858.00	Pmp22	peripheral myelin protein 22	-1.12	0.012
71801.00	Plekhf2	pleckstrin homology domain containing, family F (with FYVE domain) member 2	-1.12	0.0097
13139.00	Dgka	diacylglycerol kinase, alpha	-1.12	8.70E-05
23971.00	Papss1	3'-phosphoadenosine 5'-phosphosulfate synthase 1	-1.13	1.70E-06
107589.00	Mylk	myosin, light polypeptide kinase	-1.13	0.0029
100044862.00	ILMN_221289	PREDICTED: Mus musculus similar to Fbxl3 protein (LOC100044862), mRNA.	-1.13	0.00035
16478.00	Jund	Jun proto-oncogene related gene d	-1.13	0.0026
57890.00	Il17re	interleukin 17 receptor E	-1.13	3.80E-05
66822.00	Fbxo25	F-box protein 25	-1.13	3.00E-04
13139.00	Dgka	diacylglycerol kinase, alpha	-1.13	2.30E-05
67095.00	Trak1	trafficking protein, kinesin binding 1	-1.14	1.10E-05
22134.00	Tgoln1	trans-golgi network protein	-1.14	3.10E-05
50776.00	Polg2	polymerase (DNA directed), gamma 2, accessory subunit	-1.14	0.00019
223646.00	Naprt1	nicotinate phosphoribosyltransferase domain containing 1	-1.14	0.00012
14063.00	F2rl1	coagulation factor II (thrombin) receptor-like 1	-1.14	0.0016
212483.00	Fam193b	family with sequence similarity 193, member B	-1.14	0.00019
56643.00	Slc15a1	solute carrier family 15 (oligopeptide transporter), member 1	-1.15	0.02
66824.00	ILMN_223398	Mus musculus PYD and CARD domain containing (Pycard), mRNA.	-1.15	0.00014
59030.00	Mkks	McKusick-Kaufman syndrome protein	-1.15	1.80E-06
16168.00	Il15	interleukin 15	-1.15	2.30E-05
384009.00	Glipr2	GLI pathogenesis-related 2	-1.15	0.0033
380711.00	Rap1gap2	RAP1 GTPase activating protein 2	-1.15	3.00E-06
74754.00	Dhcr24	24-dehydrocholesterol reductase	-1.15	0.0017
66298.00	Defa21	defensin, alpha, 21	-1.15	0.019
12457.00	ILMN_208982	Mus musculus CCR4 carbon catabolite repression 4-like (S. cerevisiae) (Ccrn4l), mRNA.	-1.15	0.0029
66264.00	Ccdc28b	coiled coil domain containing 28B	-1.15	0.00041
76527.00	Il34	interleukin 34	-1.15	7.90E-07
109637.00	Upk1a	uroplakin 1A	-1.16	7.30E-05
209760.00	Tmc7	transmembrane channel-like gene family 7	-1.16	0.00082
20755.00	ILMN_215269	Mus musculus small proline-rich protein 2A (Sprr2a), mRNA.	-1.16	5.30E-06
72002.00	Slc39a5	solute carrier family 39 (metal ion transporter), member 5	-1.16	4.00E-05
108079.00	Prkaa2	protein kinase, AMP-activated, alpha 2 catalytic subunit	-1.16	6.20E-05
23954.00	Nek3	NIMA (never in mitosis gene a)-related expressed kinase 3	-1.16	1.30E-05
228576.00	Mall	mal, T-cell differentiation protein-like	-1.16	0.0011
20216.00	Acsm3	acyl-CoA synthetase medium-chain family member 3	-1.16	2.60E-05
53376.00	Usp2	ubiquitin specific peptidase 2	-1.17	0.00018
14605.00	Tsc22d3	TSC22 domain family, member 3	-1.17	0.00019
64177.00	Trpv6	transient receptor potential cation channel, subfamily V, member 6	-1.17	0.0016
170756.00	ILMN_214023	Mus musculus solute carrier family 24 (sodium/potassium/calcium exchanger), member 6 (Slc24a6), mRNA.	-1.17	0.00078
76108.00	Rap2a	RAS related protein 2a	-1.17	0.00029
76787.00	Ppfia3	protein tyrosine phosphatase, receptor type, f polypeptide (PTPRF), interacting protein (liprin), alpha 3	-1.17	0.00095
17190.00	Mbd1	methyl-CpG binding domain protein 1	-1.17	1.60E-05

212307.00	Mapre2	microtubule-associated protein, RP/EB family, member 2	-1.17	1.60E-05
56486.00	Gabarap	gamma-aminobutyric acid receptor associated protein	-1.17	4.90E-06
245038.00	Dclk3	doublecortin-like kinase 3	-1.17	4.60E-05
12653.00	Chgb	chromogranin B	-1.17	2.30E-05
110595.00	Timp4	tissue inhibitor of metalloproteinase 4	-1.18	3.50E-05
72002.00	Slc39a5	solute carrier family 39 (metal ion transporter), member 5	-1.18	0.002
67709.00	Reg4	regenerating islet-derived family, member 4	-1.18	0.002
71664.00	Mettl7b	methyltransferase like 7B	-1.18	0.002
16529.00	Kcnk5	potassium channel, subfamily K, member 5	-1.18	6.20E-05
77996.00	D730039F16Rik	RIKEN cDNA D730039F16 gene	-1.18	5.00E-04
269336.00	Ccdc32	coiled-coil domain containing 32	-1.18	5.60E-07
20730.00	Spink3	serine peptidase inhibitor, Kazal type 3	-1.19	0.0053
18799.00	Plcd1	phospholipase C, delta 1	-1.19	0.00014
16601.00	Klf9	Kruppel-like factor 9	-1.19	0.0037
16598.00	Klf2	Kruppel-like factor 2 (lung)	-1.19	0.0076
69718.00	Ipmk	inositol polyphosphate multikinase	-1.19	4.90E-06
380921.00	Dgkh	diacylglycerol kinase, eta	-1.19	3.60E-07
12684.00	Cideb	cell death-inducing DNA fragmentation factor, alpha subunit-like effector B	-1.19	0.00016
67064.00	Chmp1b	chromatin modifying protein 1B	-1.19	8.30E-05
381175.00	Ccdc68	coiled-coil domain containing 68	-1.19	0.0029
12226.00	ILMN_212740	Mus musculus B-cell translocation gene 1, anti-proliferative (Btg1), mRNA.	-1.19	1.00E-05
72361.00	ILMN_219691	Mus musculus RIKEN cDNA 2210023G05 gene (2210023G05Rik), mRNA.	-1.19	6.70E-05
21807.00	Tsc22d1	TSC22 domain family, member 1	-1.2	0.00034
18753.00	Prkcd	protein kinase C, delta	-1.2	4.40E-07
228983.00	Osbpl2	oxysterol binding protein-like 2	-1.2	9.20E-08
668837.00	ILMN_207496	PREDICTED: Mus musculus similar to ATP synthase, H+ transporting, mitochondrial F0 complex, subunit G (LOC668837), misc RNA.	-1.2	0.00047
15212.00	Hexb	hexosaminidase B	-1.2	0.00041
223706.00	Cyp2d34	cytochrome P450, family 2, subfamily d, polypeptide 34	-1.2	0.0013
12452.00	Ccng2	cyclin G2	-1.2	0.0033
223631.00	ILMN_209571	Mus musculus cDNA sequence BC025446 (BC025446), mRNA.	-1.2	0.00046
19123.00	Proc	protein C	-1.21	0.00017
260409.00	Cdc42ep3	CDC42 effector protein (Rho GTPase binding) 3	-1.21	0.0025
69787.00	Anxa13	annexin A13	-1.21	0.0049
27360.00	Add3	adducin 3 (gamma)	-1.21	4.20E-05
107723.00	Slc12a6	solute carrier family 12, member 6	-1.22	7.60E-06
223646.00	Naprt1	nicotinate phosphoribosyltransferase domain containing 1	-1.22	0.00011
239217.00	Kctd12	potassium channel tetramerisation domain containing 12	-1.22	1.90E-05
16168.00	Il15	interleukin 15	-1.22	0.00022
53897.00	Gal3st1	galactose-3-O-sulfotransferase 1	-1.22	0.00015
78252.00	Fam55b	family with sequence similarity 55, member B	-1.22	0.0047
20443.00	St3gal4	ST3 beta-galactoside alpha-2,3-sialyltransferase 4	-1.23	0.0012
18574.00	Pde1b	phosphodiesterase 1B, Ca2+-calmodulin dependent	-1.23	0.00012
105559.00	Mbnl2	muscleblind-like 2	-1.23	1.80E-05
12409.00	Cbr2	carbonyl reductase 2	-1.23	0.00047
12351.00	Car4	carbonic anhydrase 4	-1.23	0.0012
230163.00	Aldob	aldolase B, fructose-bisphosphate	-1.23	0.003
238330.00	ILMN_230817	Mus musculus RIKEN cDNA 6430527G18 gene (6430527G18Rik), mRNA.	-1.23	0.00018
67198.00	Spats2l	spermatogenesis associated, serine-rich 2-like	-1.23	0.00068
52357.00	Wwc2	WW, C2 and coiled-coil domain containing 2	-1.24	0.00049
11853.00	Rhoc	ras homolog gene family, member C	-1.24	0.00011
109731.00	Maob	monoamine oxidase B	-1.24	0.002
20238.00	Atxn1	ataxin 1	-1.24	5.80E-05
70113.00	Odf3b	outer dense fiber of sperm tails 3B	-1.24	4.70E-06
16581.00	Kifc2	kinesin family member C2	-1.25	1.40E-05
382571.00	ILMN_188365	Mus musculus potassium voltage-gated channel, subfamily F, member 1 (Kcnf1), mRNA.	-1.25	0.00073
93692.00	Glrx	glutaredoxin	-1.25	1.30E-05
13819.00	Epas1	endothelial PAS domain protein 1	-1.25	8.00E-06
13040.00	Ctss	cathepsin S	-1.25	0.0017
53422.00	ILMN_196190	Mus musculus Y box protein 2 (Ybx2), mRNA.	-1.26	9.60E-05
76273.00	Ndfip2	Nedd4 family interacting protein 2	-1.26	3.10E-05
212307.00	Mapre2	microtubule-associated protein, RP/EB family, member 2	-1.26	8.80E-07
16529.00	Kcnk5	potassium channel, subfamily K, member 5	-1.26	1.10E-05
14915.00	Guca2a	guanylate cyclase activator 2a (guanylin)	-1.26	3.80E-06
14281.00	Fos	FBJ osteosarcoma oncogene	-1.26	0.00018
68636.00	Fahd1	fumarylacetoacetate hydrolase domain containing 1	-1.26	6.10E-05
212862.00	ILMN_218521	Mus musculus choline phosphotransferase 1 (Chpt1), mRNA.	-1.26	4.20E-05
20276.00	Scnn1a	sodium channel, nonvoltage-gated 1 alpha	-1.27	8.50E-06
11758.00	ILMN_210596	Mus musculus peroxiredoxin 6 (Prdx6), mRNA.	-1.27	9.30E-06

23954.00	Nek3	NIMA (never in mitosis gene a)-related expressed kinase 3	-1.27	1.50E-06
16432.00	Itm2b	integral membrane protein 2B	-1.27	0.00054
27360.00	Add3	adducin 3 (gamma)	-1.27	6.10E-05
67038.00	2010109I03Rik	RIKEN cDNA 2010109I03 gene	-1.27	0.032
70113.00	Odf3b	outer dense fiber of sperm tails 3B	-1.27	0.00011
69864.00	1810065E05Rik	RIKEN cDNA 1810065E05 gene	-1.27	0.0056
232078.00	Thnsl2	threonine synthase-like 2 (bacterial)	-1.28	6.50E-07
21415.00	Tcf7l1	transcription factor 7-like 1 (T-cell specific, HMG box)	-1.28	0.0027
14102.00	Fas	Fas (TNF receptor superfamily member 6)	-1.28	0.00014
241041.00	Gm4956	predicted gene 4956	-1.28	1.10E-05
70031.00	Cmtm8	CKLF-like MARVEL transmembrane domain containing 8	-1.28	4.50E-06
381122.00	Capn13	calpain 13	-1.28	0.0045
12945.00	Dmbt1	deleted in malignant brain tumors 1	-1.29	0.0051
75568.00	Capsl	calcyphosine-like	-1.29	1.50E-06
74340.00	Ahcyl2	S-adenosylhomocysteine hydrolase-like 2	-1.29	0.00011
17965.00	Nbl1	neuroblastoma, suppression of tumorigenicity 1	-1.3	3.50E-05
14132.00	Fcgrt	Fc receptor, IgG, alpha chain transporter	-1.3	1.00E-04
13821.00	Epb4.1l1	erythrocyte protein band 4.1-like 1	-1.3	4.90E-05
20515.00	Slc20a1	solute carrier family 20, member 1	-1.31	1.00E-04
18703.00	Pigr	polymeric immunoglobulin receptor	-1.31	0.00073
554292.00	ILMN_238123	Mus musculus UbiE-YGHL1 fusion protein (LOC554292), mRNA.	-1.31	1.60E-05
209378.00	Itih5	inter-alpha (globulin) inhibitor H5	-1.31	1.20E-05
66270.00	Fam134b	family with sequence similarity 134, member B	-1.31	0.00018
73102.00	Slc22a23	solute carrier family 22, member 23	-1.31	4.30E-07
23971.00	Papss1	3'-phosphoadenosine 5'-phosphosulfate synthase 1	-1.32	1.50E-06
99031.00	Osbpl6	oxysterol binding protein-like 6	-1.32	2.70E-06
384061.00	ILMN_244553	Mus musculus fibronectin type III domain containing 5 (Fndc5), mRNA.	-1.32	1.60E-06
109901.00	Cela1	chymotrypsin-like elastase family, member 1	-1.32	0.0022
12864.00	Cox6c	cytochrome c oxidase, subunit VIc	-1.32	0.0055
76959.00	Chmp5	chromatin modifying protein 5	-1.32	6.50E-06
67171.00	ILMN_257311	Mus musculus transmembrane protein 77 (Tmem77), transcript variant 2, mRNA.	-1.33	4.70E-06
108682.00	Gpt2	glutamic pyruvate transaminase (alanine aminotransferase) 2	-1.33	0.00044
338521.00	Fa2h	fatty acid 2-hydroxylase	-1.33	5.00E-04
626708.00	Defa26	defensin, alpha, 26	-1.33	0.00045
71900.00	Tmem106b	transmembrane protein 106B	-1.33	5.50E-05
66938.00	1700029G01Rik	RIKEN cDNA 1700029G01 gene	-1.33	4.60E-05
19775.00	ILMN_195778	Mus musculus xenotropic and polytropic retrovirus receptor 1 (Xpr1), mRNA.	-1.34	8.00E-06
17289.00	Mertk	c-mer proto-oncogene tyrosine kinase	-1.34	0.00081
12457.00	ILMN_208982	Mus musculus CCR4 carbon catabolite repression 4-like (S. cerevisiae) (Ccrn4l), mRNA.	-1.34	0.00036
52466.00	ILMN_213036	Mus musculus solute carrier family 46, member 1 (Slc46a1), mRNA.	-1.35	0.0015
94249.00	Slc24a3	solute carrier family 24 (sodium/potassium/calcium exchanger), member 3	-1.35	1.50E-05
18569.00	Pdcd4	programmed cell death 4	-1.35	0.00011
26384.00	ILMN_226523	Mus musculus glucosamine-6-phosphate deaminase 1 (Gnpda1), mRNA.	-1.35	8.30E-05
67731.00	Fbxo32	F-box protein 32	-1.35	4.40E-05
13240.00	Defa6	defensin, alpha, 6	-1.35	0.0042
244853.00	Fam55d	family with sequence similarity 55, member D	-1.35	0.0056
227929.00	Cytip	cytohesin 1 interacting protein	-1.35	8.90E-06
232078.00	Thnsl2	threonine synthase-like 2 (bacterial)	-1.36	1.50E-06
15567.00	Slc6a4	solute carrier family 6 (neurotransmitter transporter, serotonin), member 4	-1.36	2.70E-05
18175.00	Nrap	nebulin-related anchoring protein	-1.36	7.90E-05
77996.00	D730039F16Rik	RIKEN cDNA D730039F16 gene	-1.36	1.80E-05
13101.00	Cyp2d10	cytochrome P450, family 2, subfamily d, polypeptide 10	-1.36	0.00045
13479.00	Dpep1	dipeptidase 1 (renal)	-1.37	0.0035
12700.00	Cish	cytokine inducible SH2-containing protein	-1.37	1.80E-05
212862.00	ILMN_218521	Mus musculus choline phosphotransferase 1 (Chpt1), mRNA.	-1.37	8.70E-05
58210.00	Sectm1b	secreted and transmembrane 1B	-1.38	0.0041
20196.00	S100a13	S100 calcium binding protein A13	-1.38	6.00E-05
243725.00	Ppp1r9a	protein phosphatase 1, regulatory (inhibitor) subunit 9A	-1.38	4.50E-06
68009.00	ILMN_196346	Mus musculus defensin related cryptdin 20 (Defcr20), mRNA.	-1.38	0.00065
66610.00	Abi3	ABI gene family, member 3	-1.38	0.00047
381204.00	Naaladl1	N-acetylated alpha-linked acidic dipeptidase-like 1	-1.39	0.0076
109218.00	Tmem139	transmembrane protein 139	-1.4	4.00E-05
50708.00	Hist1h1c	histone cluster 1, H1c	-1.4	5.50E-05
108112.00	ILMN_218862	Mus musculus eukaryotic translation initiation factor 4E binding protein 3 (Eif4ebp3), mRNA.	-1.4	9.40E-05
56318.00	ILMN_218213	Mus musculus acid phosphatase, prostate (Acpp), transcript variant 1, mRNA.	-1.4	1.70E-06
76507.00	Abp1	amiloride binding protein 1 (amine oxidase, copper-containing)	-1.4	6.10E-05
234797.00	6430548M08Rik	RIKEN cDNA 6430548M08 gene	-1.4	0.00084
20208.00	Saa1	serum amyloid A 1	-1.41	0.0069

76051.00	ILMN_221992	Mus musculus glucosidase, alpha; neutral C (Ganc), mRNA.	-1.41	0.00022
11676.00	Aldoc	aldolase C, fructose-bisphosphate	-1.41	9.90E-07
207819.00	ILMN_212940	Mus musculus RIKEN cDNA 4930539E08 gene (4930539E08Rik), mRNA.	-1.41	0.00013
66222.00	Serpinb1a	serine (or cysteine) peptidase inhibitor, clade B, member 1a	-1.42	4.20E-05
26395.00	Map2k1	mitogen-activated protein kinase kinase 1	-1.42	0.00086
70261.00	2010110P09Rik	RIKEN cDNA 2010110P09 gene	-1.42	0.00049
102857.00	Slc6a8	solute carrier family 6 (neurotransmitter transporter, creatine), member 8	-1.43	0.00048
71207.00	ILMN_212961	Mus musculus nudix (nucleoside diphosphate linked moiety X)-type motif 4 (Nudt4), mRNA.	-1.43	0.00018
13216.00	ILMN_196581	Mus musculus defensin, alpha 1 (Defa1), mRNA.	-1.43	0.0012
240916.00	Vsig8	V-set and immunoglobulin domain containing 8	-1.44	1.40E-05
69123.00	ILMN_210310	Mus musculus RIKEN cDNA 1810022C23 gene (1810022C23Rik), mRNA.	-1.45	4.40E-06
15430.00	Hoxd10	homeobox D10	-1.46	0.0076
71436.00	ILMN_210479	Mus musculus fibronectin leucine rich transmembrane protein 3 (Flrt3), mRNA.	-1.47	6.50E-06
67198.00	Spats2l	spermatogenesis associated, serine-rich 2-like	-1.48	9.60E-05
80890.00	ILMN_187599	Mus musculus tripartite motif protein 2 (Trim2), mRNA. XM_984114 XM_984144 XM_984172 XM_984200 XM_984238 XM_984275 XM_984313	-1.49	0.00022
245049.00	Myrip	myosin VIIA and Rab interacting protein	-1.49	0.00029
67731.00	Fbxo32	F-box protein 32	-1.49	5.80E-05
229933.00	Clca5	chloride channel calcium activated 5	-1.5	8.00E-06
76722.00	Ckmt2	creatine kinase, mitochondrial 2	-1.5	1.80E-06
16600.00	Klf4	Kruppel-like factor 4 (gut)	-1.51	2.80E-05
545156.00	Kalrn	kalirin, RhoGEF kinase	-1.51	0.00019
13239.00	ILMN_196558	Mus musculus defensin related cryptdin 5 (Defcr5), mRNA.	-1.52	0.0036
101772.00	Ano1	anoctamin 1, calcium activated chloride channel	-1.52	9.30E-06
53906.00	Phgr1	proline/histidine/glycine-rich 1	-1.52	3.40E-06
108079.00	Prkaa2	protein kinase, AMP-activated, alpha 2 catalytic subunit	-1.53	0.0011
212862.00	ILMN_218521	Mus musculus choline phosphotransferase 1 (Chpt1), mRNA.	-1.53	1.80E-05
234673.00	Ces2e	carboxylesterase 2E	-1.53	0.0011
67731.00	Fbxo32	F-box protein 32	-1.54	3.00E-04
13821.00	Epb4.1l1	erythrocyte protein band 4.1-like 1	-1.54	1.80E-06
19017.00	Ppargc1a	peroxisome proliferative activated receptor, gamma, coactivator 1 alpha	-1.55	1.00E-04
13218.00	Defa-rs1	defensin, alpha, related sequence 1	-1.55	0.0076
56448.00	ILMN_223233	Mus musculus cytochrome P450, family 2, subfamily d, polypeptide 22 (Cyp2d22), mRNA.	-1.55	0.0032
69574.00	Cmbl	carboxymethylenebutenolidase-like (Pseudomonas)	-1.55	5.50E-05
433470.00	AA467197	expressed sequence AA467197	-1.55	0.00059
227327.00	B3gnt7	UDP-GlcNAc:betaGal beta-1,3-N-acetylglucosaminyltransferase 7	-1.56	0.0025
11829.00	Aqp4	aquaporin 4	-1.56	0.00019
239606.00	Slc2a13	solute carrier family 2 (facilitated glucose transporter), member 13	-1.57	2.10E-05
102871.00	D330045A20Rik	RIKEN cDNA D330045A20 gene	-1.58	7.50E-05
231946.00	D330028D13Rik	RIKEN cDNA D330028D13 gene	-1.58	0.00029
23844.00	Clca3	chloride channel calcium activated 3	-1.58	0.0081
319848.00	Slc17a4	solute carrier family 17 (sodium phosphate), member 4	-1.59	4.60E-05
14199.00	Fhl1	four and a half LIM domains 1	-1.59	0.0087
20510.00	Slc1a1	solute carrier family 1 (neuronal/epithelial high affinity glutamate transporter, system Xag), member 1	-1.6	3.70E-06
170677.00	Cdhr1	cadherin-related family member 1	-1.6	1.70E-06
68009.00	ILMN_196346	Mus musculus defensin related cryptdin 20 (Defcr20), mRNA.	-1.6	0.00056
384061.00	Fndc5	fibronectin type III domain containing 5	-1.61	3.50E-07
230576.00	Ttc22	tetratricopeptide repeat domain 22	-1.62	5.80E-06
20541.00	Slc8a1	solute carrier family 8 (sodium/calcium exchanger), member 1	-1.63	0.00094
207259.00	Zbtb7c	zinc finger and BTB domain containing 7C	-1.64	1.50E-05
13240.00	Defa6	defensin, alpha, 6	-1.64	0.0011
16600.00	Klf4	Kruppel-like factor 4 (gut)	-1.65	4.00E-05
433904.00	Ociad2	OCIA domain containing 2	-1.66	4.40E-05
56209.00	Gde1	glycerophosphodiester phosphodiesterase 1	-1.66	8.90E-06
14199.00	Fhl1	four and a half LIM domains 1	-1.67	0.003
226781.00	Slc30a10	solute carrier family 30, member 10	-1.68	0.0044
76722.00	Ckmt2	creatine kinase, mitochondrial 2	-1.68	1.10E-06
258458.00	Olfr165	olfactory receptor 165	-1.7	6.00E-06
16600.00	Klf4	Kruppel-like factor 4 (gut)	-1.7	9.20E-05
56226.00	Espn	espin	-1.7	2.20E-05
20342.00	ILMN_227312	Mus musculus selenium binding protein 2 (Selenbp2), mRNA.	-1.71	0.00044
67182.00	Pdzk1ip1	PDZK1 interacting protein 1	-1.71	3.80E-05
100044204.00	ILMN_221943	PREDICTED: Mus musculus hypothetical protein LOC100044204 (LOC100044204), mRNA.	-1.71	0.00011
56209.00	Gde1	glycerophosphodiester phosphodiesterase 1	-1.71	1.70E-06
76787.00	Ppfia3	protein tyrosine phosphatase, receptor type, f polypeptide (PTPRF), interacting protein (liprin), alpha 3	-1.74	3.80E-05
56209.00	Gde1	glycerophosphodiester phosphodiesterase 1	-1.74	4.70E-06
99663.00	Clca6	chloride channel calcium activated 6	-1.74	0.03
192970.00	Dhrs11	dehydrogenase/reductase (SDR family) member 11	-1.74	5.70E-06
18858.00	Pmp22	peripheral myelin protein 22	-1.77	0.00094

74134.00	Cyp2s1	cytochrome P450, family 2, subfamily s, polypeptide 1	-1.77	5.00E-06
20510.00	Slc1a1	solute carrier family 1 (neuronal/epithelial high affinity glutamate transporter, system Xag), member 1	-1.78	6.70E-07
20910.00	Stxbp1	syntaxin binding protein 1	-1.79	6.50E-06
66438.00	Hamp2	hepcidin antimicrobial peptide 2	-1.79	0.00013
71687.00	Tmem25	transmembrane protein 25	-1.8	2.50E-06
20755.00	ILMN_215269	Mus musculus small proline-rich protein 2A (Sprr2a), mRNA.	-1.8	7.60E-06
66090.00	Ypel3	yippee-like 3 (Drosophila)	-1.85	2.00E-06
68416.00	Sycn	syncollin	-1.85	0.016
100041194.00	Ahnak2	AHNAK nucleoprotein 2	-1.85	2.90E-07
268860.00	Abat	4-aminobutyrate aminotransferase	-1.85	4.40E-07
15446.00	Hpgd	hydroxyprostaglandin dehydrogenase 15 (NAD)	-1.86	2.60E-05
12865.00	Cox7a1	cytochrome c oxidase, subunit VIIa 1	-1.87	4.50E-06
67307.00	Pbld2	phenazine biosynthesis-like protein domain containing 2	-1.87	3.70E-07
13034.00	ILMN_253611	Mus musculus cathepsin E (Ctse), mRNA.	-1.89	0.00017
20259.00	Scin	scinderin	-1.9	4.90E-05
16429.00	Itln1	intelectin 1 (galactofuranose binding)	-1.9	2.00E-04
12231.00	Btn1a1	butyrophilin, subfamily 1, member A1	-1.91	4.80E-07
230163.00	Aldob	aldolase B, fructose-bisphosphate	-1.91	0.00055
77889.00	Lbh	limb-bud and heart	-1.93	4.90E-05
20500.00	Slc13a2	solute carrier family 13 (sodium-dependent dicarboxylate transporter), member 2	-1.95	0.00062
26456.00	Sema4g	sema domain, immunoglobulin domain (Ig), transmembrane domain (TM) and short cytoplasmic domain, (semaphorin) 4G	-1.96	4.20E-06
268860.00	Abat	4-aminobutyrate aminotransferase	-1.96	1.60E-05
72948.00	Tppp	tubulin polymerization promoting protein	-1.98	6.50E-06
56226.00	Espn	espin	-1.98	2.30E-05
20363.00	Sepp1	selenoprotein P, plasma, 1	-2.02	0.0034
100044291.00	ILMN_221210	PREDICTED: Mus musculus hypothetical protein LOC100044291 (LOC100044291), mRNA.	-2.02	9.40E-05
20531.00	Slc34a2	solute carrier family 34 (sodium phosphate), member 2	-2.05	0.0019
16173.00	Il18	interleukin 18	-2.05	6.70E-05
23959.00	Nt5e	5' nucleotidase, ecto	-2.06	0.0012
77889.00	Lbh	limb-bud and heart	-2.08	0.00014
268480.00	Rapgefl1	Rap guanine nucleotide exchange factor (GEF)-like 1	-2.1	3.10E-07
23959.00	Nt5e	5' nucleotidase, ecto	-2.11	2.10E-05
13370.00	Dio1	deiodinase, iodothyronine, type I	-2.11	3.10E-06
57319.00	Smpdl3a	sphingomyelin phosphodiesterase, acid-like 3A	-2.12	8.50E-06
493583.00	ILMN_256959	Mus musculus intelectin b (Itlnb), mRNA.	-2.2	4.00E-05
13370.00	Dio1	deiodinase, iodothyronine, type I	-2.23	1.70E-06
12579.00	Cdkn2b	cyclin-dependent kinase inhibitor 2B (p15, inhibits CDK4)	-2.26	1.70E-06
20363.00	Sepp1	selenoprotein P, plasma, 1	-2.3	0.00079
268480.00	Rapgefl1	Rap guanine nucleotide exchange factor (GEF)-like 1	-2.33	6.10E-06
109791.00	Clps	colipase, pancreatic	-2.37	2.50E-05
17287.00	Mep1a	meprin 1 alpha	-2.41	0.00036
101488.00	Slco2b1	solute carrier organic anion transporter family, member 2b1	-2.52	7.30E-05
109791.00	Clps	colipase, pancreatic	-2.57	4.60E-05
21818.00	Tgm3	transglutaminase 3, E polypeptide	-2.58	4.20E-05
69083.00	Sult1c2	sulfotransferase family, cytosolic, 1C, member 2	-2.58	9.90E-08
67971.00	Tppp3	tubulin polymerization-promoting protein family member 3	-2.59	4.80E-07
53315.00	Sult1d1	sulfotransferase family 1D, member 1	-2.63	1.60E-06
56185.00	Hao2	hydroxyacid oxidase 2	-2.67	7.10E-06
20887.00	Sult1a1	sulfotransferase family 1A, phenol-preferring, member 1	-2.7	2.00E-05
393082.00	ILMN_243966	Mus musculus methyltransferase like 7A2 (Mettl7a2), mRNA.	-2.73	1.30E-09
545288.00	Cyp2c67	cytochrome P450, family 2, subfamily c, polypeptide 67	-2.77	4.40E-07
13615.00	Edn2	endothelin 2	-2.98	2.80E-05
22635.00	Zan	zonadhesin	-2.99	3.60E-08
233038.00	Nccrp1	non-specific cytotoxic cell receptor protein 1 homolog (zebrafish)	-3.11	2.10E-06
216225.00	Slc5a8	solute carrier family 5 (iodide transporter), member 8	-3.13	1.60E-06
219033.00	Ang4	angiogenin, ribonuclease A family, member 4	-3.25	6.70E-05
13107.00	Cyp2f2	cytochrome P450, family 2, subfamily f, polypeptide 2	-3.49	3.60E-08
18947.00	Pnliprp2	pancreatic lipase-related protein 2	-3.52	1.10E-05
232889.00	Pla2g4c	phospholipase A2, group IVC (cytosolic, calcium-independent)	-3.72	3.00E-04
331063.00	ILMN_196357	Mus musculus expressed sequence AI987692 (AI987692), mRNA.	-4.02	4.50E-06
18947.00	Pnliprp2	pancreatic lipase-related protein 2	-4.18	2.40E-06
270328.00	ILMN_196360	Mus musculus gasdermin C3 (Gsdmc3), mRNA.	-4.29	1.90E-07
331063.00	Gsdmc2	gasdermin C2	-4.4	3.50E-07
100045250.00	ILMN_196357	PREDICTED: Mus musculus hypothetical protein LOC100045250 (LOC100045250), misc RNA.	-4.5	3.70E-06

Supplementary Table S3

Ct values of genes analyzed by qRT-PCR in HEK293 cells treated with DMSO (control) or BIO; obtained Ct values were normalized to β-actin gene expression; SD, standard deviation.

	DMSO		BIO	
gene name	Ct values	SD	Ct values	SD
MSX1	28.47	0.15	27.96	0.08
MSX2	25.74	0.04	25.09	0.11
NKD1	26.23	0.18	22.42	0.15
TROY	26.21	0.05	24.43	0.11
UBB	18.62	0.06	18.74	0.01

Supplementary Table S3

Ct values of genes analyzed by qRT-PCR in STF cells with the intact (control) or truncated *APC* gene in exon 10 or exon 15; obtained Ct values were normalized to β-actin gene expression; SD, standard deviation.

	control		exon 10		exon 15	
gene name	Ct values	SD	Ct values	SD	Ct values	SD
AXIN2	31.17	0.40	29.54	0.01	28.75	0.01
MSX1	28.28	0.43	27.04	0.02	25.58	0.11
MSX2	27.20	0.16	25.78	0.01	24.14	0.03
NKD1	28.36	0.06	24.14	0.13	25.22	0.07
UBB	19.40	0.04	18.97	0.01	19.80	0.02

Supplementary Table S3

Ct values of genes analyzed by qRT-PCR in SW480 cells upon transfection with non-silencing (nsc) siRNAs or siRNAs targeting *β-catenin* mRNA; obtained Ct values were normalized to *β-actin* gene expression; SD, standard deviation.

	nsc siRNAs		β-catenin siRNAs	
gene name	Ct values	SD	Ct values	SD
β-CATENIN	21.17	0.08	26.48	0.30
MSX1	26.10	0.01	28.54	0.42
MSX2	25.22	0.24	27.65	0.17
NKD1	22.93	0.12	27.39	0.16
TROY	26.72	0.00	29.23	0.08
UBB	18.86	0.16	19.56	0.04

Supplementary Table S3

Ct values of genes analyzed by qRT-PCR in SW620 cells upon transfection with non-silencing (nsc) siRNAs or siRNAs targeting *β-catenin* mRNA; obtained Ct values were normalized to *β-actin* gene expression; SD, standard deviation.

	nsc siRNAs		β-catenin siRNAs	
gene name	Ct values	SD	Ct values	SD
β-CATENIN	20.59	1.15	23.66	0.10
MSX1	20.64	0.04	24.39	0.06
MSX2	24.28	0.06	25.99	0.11
NKD1	20.90	0.16	25.12	0.01
TROY	27.24	0.25	29.14	0.42
UBB	17.47	0.39	17.81	0.25

Supplementary Table S3

Ct values of genes analyzed by qRT-PCR in STF cells with the *APC* gene truncated in exon 10 upon transfection with non-silencing (nsc) siRNAs or siRNAs targeting *β-catenin* mRNA; obtained Ct values were normalized to *β-actin* gene expression; SD, standard deviation.

	nsc siRNAs		β-catenin siRNAs	
gene name	Ct values	SD	Ct values	SD
AXIN2	23.68	0.02	25.10	0.02
β-CATENIN	22.48	0.08	25.75	0.11
MSX1	28.99	0.14	29.83	0.10
MSX2	26.31	0.09	27.15	0.09
UBB	19.56	0.05	19.38	0.03

Supplementary Table S4

Differentialy expressed genes (with |logFC|≥ 1; p ≤ 0.05) in the small intestinal Msx1 wt and Msx1-deficient hyperplastic epithelium

ENTREZ	SYMBOL	GENENAME	logFC	p-value
170942	Erdr1	erythroid differentiation regulator 1	3.52	2.00E-04
57742	Abhd1	abhydrolase domain containing 1	2.93	4.10E-05
57742	Abhd1	abhydrolase domain containing 1	2.84	5.60E-05
434794		Mus musculus X-linked lymphocyte-regulated 4A (Xlr4a), mRNA	2.81	0.0083
11746	Anxa4	annexin A4	2.77	0.066
57742	Abhd1	abhydrolase domain containing 1	2.26	0.00068
68337	Crip2	cysteine rich protein 2	2.19	6.90E-05
223227	Sox21	SRY (sex determining region Y)-box 21	2.1	0.0098
20249	Scd1	stearoyl-Coenzyme A desaturase 1	2.03	0.001
15122		Mus musculus hemoglobin alpha, adult chain 1 (Hba-a1), mRNA.	1.92	0.19
19652	Rbm3	RNA binding motif protein 3	1.88	0.0014
14733	Gpc1	glypican 1	1.88	0.018
406217	Bex4	brain expressed X-linked 4	1.79	0.014
14472	Gbx2	gastrulation brain homeobox 2	1.78	0.019
223227	Sox21	SRY (sex determining region Y)-box 21	1.77	0.014
20350	Sema3f	sema domain, immunoglobulin domain (Ig), short basic domain, secreted, (semaphorin) 3F	1.67	0.014
192212	Prom2	prominin 2	1.65	0.048
69195	Tmem121	transmembrane protein 121	1.64	0.14
64293	Stk32b	serine/threonine kinase 32B	1.64	0.00092
23962	Oasl2	2'-5' oligoadenylate synthetase-like 2	1.57	0.0045
16145	Igtp	interferon gamma induced GTPase	1.57	0.1
329064	Pkd2l1	polycystic kidney disease 2-like 1	1.52	0.0026
15360	Hmgcs2	3-hydroxy-3-methylglutaryl-Coenzyme A synthase 2	1.52	0.006
14469	Gbp2	guanylate binding protein 2	1.5	0.22
21380	Tbx1	T-box 1	1.49	0.0081
20350	Sema3f	sema domain, immunoglobulin domain (Ig), short basic domain, secreted, (semaphorin) 3F	1.42	0.013
100038882	Isg15	ISG15 ubiquitin-like modifier	1.41	0.067
17329	Cxcl9	chemokine (C-X-C motif) ligand 9	1.41	0.12
68713	Ifitm1	interferon induced transmembrane protein 1	1.34	0.11
76943	Psapl1	prosaposin-like 1	1.32	0.0097
13644	Efs	embryonal Fyn-associated substrate	1.32	0.02
72434	Lypd3	Ly6/Plaur domain containing 3	1.31	0.011
67855	Asprv1	aspartic peptidase, retroviral-like 1	1.31	0.047
22269	Upk2	uroplakin 2	1.3	0.0084
24110	Usp18	ubiquitin specific peptidase 18	1.29	0.018
52250	Reep1	receptor accessory protein 1	1.29	0.015
11749	Anxa6	annexin A6	1.28	0.049
226245	Plekhs1	pleckstrin homology domain containing, family S member 1	1.28	0.028
20671	Sox17	SRY (sex determining region Y)-box 17	1.27	0.05
108052	Slc14a1	solute carrier family 14 (urea transporter), member 1	1.27	0.002
11567	Avil	advillin	1.27	0.14
67967	Pold3	polymerase (DNA-directed), delta 3, accessory subunit	1.26	0.0033
58185	Rsad2	radical S-adenosyl methionine domain containing 2	1.25	0.024
17242	Mdk	midkine	1.25	4.10E-05
20129	Rptn	repetin	1.24	0.0021
20671	Sox17	SRY (sex determining region Y)-box 17	1.23	0.0072
67951	Tubb6	tubulin, beta 6 class V	1.21	0.04
20910	Stxbp1	syntaxin binding protein 1	1.21	0.0026
268396	Sh3pxd2b	SH3 and PX domains 2B	1.2	0.0029
13034	Ctse	cathepsin E	1.2	0.11
22153	Tubb4a	tubulin, beta 4A class IVA	1.19	0.053
15959	Ifit3	interferon-induced protein with tetratricopeptide repeats 3	1.19	0.041
231050	Galnt11	UDP-N-acetyl-alpha-D-galactosamine:polypeptide N-acetylgalactosaminyltransferase 11	1.19	0.036
69065	Chac1	ChaC, cation transport regulator 1	1.18	0.045
108052	Slc14a1	solute carrier family 14 (urea transporter), member 1	1.17	0.012
654824	Ankrd37	ankyrin repeat domain 37	1.17	0.0047
225027	Srsf7	serine/arginine-rich splicing factor 7	1.16	0.039
240334	Pcyox1l	prenylcysteine oxidase 1 like	1.16	0.001
246728	Oas2	2'-5' oligoadenylate synthetase 2	1.15	0.12
27261	Dok3	docking protein 3	1.15	0.0033
74007	Btbd11	BTB (POZ) domain containing 11	1.15	0.042
11752	Anxa8	annexin A8	1.12	0.1
22228	Ucp2	uncoupling protein 2 (mitochondrial, proton carrier)	1.1	9.60E-05
226123	Morn4	MORN repeat containing 4	1.1	0.0011
406217		Mus musculus brain expressed gene 4 (Bex4), mRNA.	1.09	0.027
244886	Tmem266	transmembrane protein 266	1.09	0.0042
240675	Vwa2	von Willebrand factor A domain containing 2	1.08	0.0048
58194	Sh3kbp1	SH3-domain kinase binding protein 1	1.08	0.058
331063	Gsdmc2	gasdermin C2	1.08	0.0099
18812	Prl2c3	prolactin family 2, subfamily c, member 3	1.07	0.0057

27395	Mrpl15	mitochondrial ribosomal protein L15	1.07	0.0046
24110	Usp18	ubiquitin specific peptidase 18	1.07	0.0093
15958	Ifit2	interferon-induced protein with tetratricopeptide repeats 2	1.07	0.02
54396	Irgm2	immunity-related GTPase family M member 2	1.06	0.17
15505	Hsph1	heat shock 105kDa/110kDa protein 1	1.06	0.003
24110	Usp18	ubiquitin specific peptidase 18	1.05	0.023
331063	Gsdmc2	gasdermin C2	1.05	0.021
54352	Irx5	Iroquois related homeobox 5 (Drosophila)	1.03	0.011
11639	Ak4	adenylate kinase 4	1.02	0.061
74777	Sepn1	selenoprotein N, 1	1.01	0.02
213171	Prss27	protease, serine 27	1.01	0.06
21939	Cd40	CD40 antigen	1.01	0.002
21858	Timp2	tissue inhibitor of metalloproteinase 2	1	0.016
22379	Fmnl3	formin-like 3	1	0.18
11684	Alox12	arachidonate 12-lipoxygenase	1	0.0048
381524	AI427809	expressed sequence AI427809	1	0.0057
67800	Dgat2	diacylglycerol O-acyltransferase 2	-1	0.0065
21685	Tef	thyrotroph embryonic factor	-1.01	0.012
217166	Nr1d1	nuclear receptor subfamily 1, group D, member 1	-1.01	0.034
56615	Mgst1	microsomal glutathione S-transferase 1	-1.01	0.021
216136	Ilvbl	ilvB (bacterial acetolactate synthase)-like	-1.01	0.0079
66412	Arrdc4	arrestin domain containing 4	-1.02	0.0092
74525	Fam234b	family with sequence similarity 234, member B	-1.02	0.0084
330962	Slc51b	solute carrier family 51, beta subunit	-1.03	0.17
14345	Fut4	fucosyltransferase 4	-1.03	0.0057
74519	Cyp2j9	cytochrome P450, family 2, subfamily j, polypeptide 9	-1.03	0.18
11459	Acta1	actin, alpha 1, skeletal muscle	-1.03	0.06
50934	Slc7a8	solute carrier family 7 (cationic amino acid transporter, y+ system), member 8	-1.04	0.022
71584	Gdpd2	glycerophosphodiester phosphodiesterase domain containing 2	-1.04	0.0091
226040	Tmem252	transmembrane protein 252	-1.04	0.00038
101488	Slco2b1	solute carrier organic anion transporter family, member 2b1	-1.05	0.015
74338	Slc6a19	solute carrier family 6 (neurotransmitter transporter), member 19	-1.05	0.11
18150		Mus musculus nucleoplasmin 3 (Npm3), mRNA.	-1.05	0.043
18096	Nkx6-1	NK6 homeobox 1	-1.05	0.04
13088	Cyp2b10	cytochrome P450, family 2, subfamily b, polypeptide 10	-1.05	0.11
100434	Slc44a1	solute carrier family 44, member 1	-1.06	0.0037
19341	Rab4a	RAB4A, member RAS oncogene family	-1.06	0.0069
18036	Nfkbib	nuclear factor of kappa light polypeptide gene enhancer in B cells inhibitor, beta	-1.06	0.004
433278	Khdc1c	KH domain containing 1C	-1.06	0.00052
14219	Ctgf	connective tissue growth factor	-1.06	0.00012
68396	Nat8	N-acetyltransferase 8 (GCN5-related)	-1.06	0.16
223631	BC025446	cDNA sequence BC025446	-1.06	0.056
66273	Aamdc	adipogenesis associated Mth938 domain containing	-1.06	0.0027
225997	Trpm6	transient receptor potential cation channel, subfamily M, member 6	-1.07	0.00025
242864	Napepld	N-acyl phosphatidylethanolamine phospholipase D	-1.07	0.038
68943	Pink1	PTEN induced putative kinase 1	-1.07	0.0039
15186	Hdc	histidine decarboxylase	-1.07	0.012
97998	Deptor	DEP domain containing MTOR-interacting protein	-1.07	0.018
11829	Aqp4	aquaporin 4	-1.07	0.0059
23849	Klf6	Kruppel-like factor 6	-1.08	0.00018
13120	Cyp4b1	cytochrome P450, family 4, subfamily b, polypeptide 1	-1.08	0.11
381903	Alg8	asparagine-linked glycosylation 8 (alpha-1,3-glucosyltransferase)	-1.08	0.017
230163	Aldob	aldolase B, fructose-bisphosphate	-1.08	0.052
11539	Adora1	adenosine A1 receptor	-1.08	0.0032
217166	Nr1d1	nuclear receptor subfamily 1, group D, member 1	-1.09	0.061
72303	Cyp2c65	cytochrome P450, family 2, subfamily c, polypeptide 65	-1.09	0.11
102294	Cyp4v3	cytochrome P450, family 4, subfamily v, polypeptide 3	-1.1	0.14
22064		Mus musculus transient receptor potential cation channel, subfamily C, member 2 (Trpc2), transcript variant 1, mRNA.	-1.11	0.019
208659	Fam20a	family with sequence similarity 20, member A	-1.11	0.0055
71972	Dnmbp	dynamin binding protein	-1.11	0.034
104681	Slc16a6	solute carrier family 16 (monocarboxylic acid transporters), member 6	-1.14	0.0023
13106	Cyp2e1	cytochrome P450, family 2, subfamily e, polypeptide 1	-1.14	0.22
19329	Rab17	RAB17, member RAS oncogene family	-1.15	0.0031
104009	Qsox1	quiescin Q6 sulfhydryl oxidase 1	-1.15	0.0048
17988	Ndrg1	N-myc downstream regulated gene 1	-1.15	0.061
18604	Pdk2	pyruvate dehydrogenase kinase, isoenzyme 2	-1.16	0.063
57257	Vav3	vav 3 oncogene	-1.17	0.017
104158	Ces1d	carboxylesterase 1D	-1.17	0.3
14164	Fgf1	fibroblast growth factor 1	-1.18	0.035
225913	Tkfc	triokinase, FMN cyclase	-1.19	0.0058
230163	Aldob	aldolase B, fructose-bisphosphate	-1.19	0.028
72514	Fgfbp3	fibroblast growth factor binding protein 3	-1.2	0.0013
67470	Abcg8	ATP-binding cassette, sub-family G (WHITE), member 8	-1.2	0.0086
20526	Slc2a2	solute carrier family 2 (facilitated glucose transporter), member 2	-1.21	0.037
68404	Nrn1	neuritin 1	-1.21	0.0021
109731	Maob	monoamine oxidase B	-1.21	0.065

71584	Gdpd2	glycerophosphodiester phosphodiesterase domain containing 2	-1.22	0.019
56643	Slc15a1	solute carrier family 15 (oligopeptide transporter), member 1	-1.23	0.076
15199	Hebp1	heme binding protein 1	-1.23	0.052
114664	Hsd17b11	hydroxysteroid (17-beta) dehydrogenase 11	-1.24	0.02
14456	Gas6	growth arrest specific 6	-1.24	0.014
11826	Aqp1	aquaporin 1	-1.24	0.036
117591	Slc2a9	solute carrier family 2 (facilitated glucose transporter), member 9	-1.25	0.0094
328059	Slc7a15	solute carrier family 7 (cationic amino acid transporter, y+ system), member 15	-1.26	0.12
72373	Psca	prostate stem cell antigen	-1.26	0.0058
15567	Slc6a4	solute carrier family 6 (neurotransmitter transporter, serotonin), member 4	-1.29	0.006
68180	Hyi	hydroxypyruvate isomerase (putative)	-1.29	0.0043
192653	Ttc36	tetratricopeptide repeat domain 36	-1.31	0.0019
76279	Cyp2d26	cytochrome P450, family 2, subfamily d, polypeptide 26	-1.31	0.18
67473	Slc47a1	solute carrier family 47, member 1	-1.31	0.17
15446	Hpgd	hydroxyprostaglandin dehydrogenase 15 (NAD)	-1.34	0.06
12696	Cirbp	cold inducible RNA binding protein	-1.34	0.0014
26458	Slc27a2	solute carrier family 27 (fatty acid transporter), member 2	-1.35	0.0039
104681	Slc16a6	solute carrier family 16 (monocarboxylic acid transporters), member 6	-1.37	0.0067
14164	Fgf1	fibroblast growth factor 1	-1.37	0.021
69983	Sis	sucrase isomaltase (alpha-glucosidase)	-1.37	0.0039
93695	Gpnmb	glycoprotein (transmembrane) nmb	-1.38	0.11
56350	Arl3	ADP-ribosylation factor-like 3	-1.39	4.00E-04
108099	Prkag2	protein kinase, AMP-activated, gamma 2 non-catalytic subunit	-1.4	0.0035
232889	Pla2g4c	phospholipase A2, group IVC (cytosolic, calcium-independent)	-1.4	0.22
209387	Trim30d	tripartite motif-containing 30D	-1.4	0.03
384783	Irs2	insulin receptor substrate 2	-1.43	0.0096
20526	Slc2a2	solute carrier family 2 (facilitated glucose transporter), member 2	-1.44	0.033
78748	Rassf10	Ras association (RalGDS/AF-6) domain family (N-terminal) member 10	-1.45	0.0065
27276	Plekhb1	pleckstrin homology domain containing, family B (evectins) member 1	-1.46	0.0011
11826	Aqp1	aquaporin 1	-1.46	0.021
235674	Acaa1b	acetyl-Coenzyme A acyltransferase 1B	-1.47	0.19
66116	Nat8f1	N-acetyltransferase 8 (GCN5-related) family member 1	-1.48	4.00E-04
69710	Arap1	ArfGAP with RhoGAP domain, ankyrin repeat and PH domain 1	-1.48	0.00034
20526	Slc2a2	solute carrier family 2 (facilitated glucose transporter), member 2	-1.54	0.067
66298	Defa21	defensin, alpha, 21	-1.56	0.0033
76279	Cyp2d26	cytochrome P450, family 2, subfamily d, polypeptide 26	-1.57	0.18
170442	Bbox1	butyrobetaine (gamma), 2-oxoglutarate dioxygenase 1 (gamma-butyrobetaine hydroxylase)	-1.58	0.026
76279	Cyp2d26	cytochrome P450, family 2, subfamily d, polypeptide 26	-1.59	0.14
20887	Sult1a1	sulfotransferase family 1A, phenol-preferring, member 1	-1.6	0.049
11826	Aqp1	aquaporin 1	-1.6	0.011
20019	Polr1a	polymerase (RNA) I polypeptide A	-1.61	0.00012
16204	Fabp6	fatty acid binding protein 6, ileal (gastrotropin)	-1.62	0.2
67432	Hoga1	4-hydroxy-2-oxoglutarate aldolase 1	-1.64	0.0047
381058	Unc93a	unc-93 homolog A (C. elegans)	-1.65	0.019
545902	Ptprh	protein tyrosine phosphatase, receptor type, H	-1.67	0.0083
20170	Hps6	Hermansky-Pudlak syndrome 6	-1.72	0.00016
233575	Pgap2	post-GPI attachment to proteins 2	-1.73	0.0023
53315	Sult1d1	sulfotransferase family 1D, member 1	-1.76	0.0043
11997	Akr1b7	aldo-keto reductase family 1, member B7	-1.78	0.026
14121	Fbp1	fructose bisphosphatase 1	-1.79	0.016
16548	Khk	ketohexokinase	-1.86	0.0085
16548	Khk	ketohexokinase	-1.9	0.0022
67432	Hoga1	4-hydroxy-2-oxoglutarate aldolase 1	-1.9	0.013
14377	G6pc	glucose-6-phosphatase, catalytic	-1.95	0.021
192236	Hps1	Hermansky-Pudlak syndrome 1	-2.05	0.00082
54150	Rdh7	retinol dehydrogenase 7	-2.08	0.033
233549	Mogat2	monoacylglycerol O-acyltransferase 2	-2.12	0.0023
69710	Arap1	ArfGAP with RhoGAP domain, ankyrin repeat and PH domain 1	-2.12	0.0015
11522	Adh1	alcohol dehydrogenase 1 (class I)	-2.13	0.012
56018	Stard10	START domain containing 10	-2.14	0.0017
12780	Abcc2	ATP-binding cassette, sub-family C (CFTR/MRP), member 2	-2.18	0.0059
54150	Rdh7	retinol dehydrogenase 7	-2.23	0.032
56388	Cyp3a25	cytochrome P450, family 3, subfamily a, polypeptide 25	-2.4	0.034
11522	Adh1	alcohol dehydrogenase 1 (class I)	-2.44	0.0073
17921	Myo7a	myosin VIIA	-2.54	0.0032
13112	Cyp3a11	cytochrome P450, family 3, subfamily a, polypeptide 11	-2.82	0.065
17701	Msx1	msh homeobox 1	-3.05	0.064
233549	Mogat2	monoacylglycerol O-acyltransferase 2	-3.14	0.0037
233571	P2ry6	pyrimidinergic receptor P2Y, G-protein coupled, 6	-3.31	0.00024
18479	Pak1	p21 protein (Cdc42/Rac)-activated kinase 1	-3.4	0.003
68185	Coa4	cytochrome c oxidase assembly factor 4	-3.44	8.00E-04
52443	Mrpl48	mitochondrial ribosomal protein L48	-3.56	0.0018
52443	Mrpl48	mitochondrial ribosomal protein L48	-3.77	0.0016
27050	Rps3	ribosomal protein S3	-6.14	0.0025

Supplementary Table S5

Ct values of genes analyzed in the small intestinal epithelium of the indicated mice 7 days after tamoxifen administration; the values were normalized to *β-actin* gene expression; SD, standard deviation.

	Control		*Apc* $^{KO/KO}$ *Msx1* $^{+/+}$		*Apc* $^{KO/KO}$ *Msx1* $^{KO/KO}$	
gene name	Ct values	SD	Ct values	SD	Ct values	SD
Ascl2	34.25	0.80	27.76	0.51	27.26	0.15
Axin2	30.52	0.74	25.49	0.58	25.27	0.11
Chromogranin A	28.48	0.49	28.21	0.33	28.97	0.35
GAPDH	21.43	0.31	20.84	0.59	20.31	0.14
Lgr5	34.10	0.62	28.98	0.49	28.60	0.03
Msx1	42.54	1.51	28.95	0.95	32.64	0.72
Msx2	45.11	0.69	34.54	0.16	34.31	1.04
SI	25.27	0.73	27.27	0.01	28.73	0.46
SP5	30.74	0.46	27.34	1.19	26.73	0.15

Supplementary Table S6

Differentialy expressed genes (|logFC| ≥ 0.8) in in the Apc/Msx1 double-deficient colonic mucosa when compared to the Apc-deficient tissue with intact Msx1

PROBE ID	ENSEMBL ID	SYMBOL	GENENAME	logFC	p-value
ENSMUST00000094836	ENSMUSG00000029123	Stk32b	serine/threonine kinase 32B	2.06e+00	3.70e-09
ENSMUST00000103399	ENSMUSG00000076598	Igkv3-7	immunoglobulin kappa variable 3-7	1.94e+00	8.93e-04
ENSMUST00000197560	ENSMUSG00000076598	Igkv3-7	immunoglobulin kappa variable 3-7	1.94e+00	8.93e-04
ENSMUST00000177591	ENSMUSG00000096768	Erdr1	erythroid differentiation regulator 1	1.48e+00	2.43e-02
ENSMUST00000177671	ENSMUSG00000096768	Erdr1	erythroid differentiation regulator 1	1.44e+00	2.60e-02
ENSMUST00000178789	ENSMUSG00000095562	Gm21887	predicted gene, 21887	1.37e+00	2.73e-02
ENSMUST00000179483	ENSMUSG00000096768	Erdr1	erythroid differentiation regulator 1	1.37e+00	2.73e-02
ENSMUST00000180251	ENSMUSG00000095562	Gm21887	predicted gene, 21887	1.37e+00	2.73e-02
ENSMUST00000044159	ENSMUSG00000060807	Serpina6	serine (or cysteine) peptidase inhibitor, clade A, member 6	1.36e+00	1.54e-04
ENSMUST00000179077	ENSMUSG00000096768	Erdr1	erythroid differentiation regulator 1	1.34e+00	2.64e-02
ENSMUST00000100692	ENSMUSG00000095528	Gm10375	predicted gene 10375	1.16e+00	3.12e-02
ENSMUST00000163970	ENSMUSG00000095528	Gm10375	predicted gene 10375	1.16e+00	3.12e-02
ENSMUST00000196706	ENSMUSG00000027869	Hsd3b6	hydroxy-delta-5-steroid dehydrogenase, 3 beta- and steroid delta-isomerase 6	1.15e+00	4.68e-04
ENSMUST00000211636	ENSMUSG00000040640	Erc2	ELKS/RAB6-interacting/CAST family member 2	1.15e+00	8.61e-04
ENSMUST00000144418	ENSMUSG00000028469	Npr2	natriuretic peptide receptor 2	1.15e+00	1.48e-02
ENSMUST00000172766	ENSMUSG00000050423	Ppp1r3g	protein phosphatase 1, regulatory (inhibitor) subunit 3G	1.13e+00	5.97e-03
ENSMUST00000113512	ENSMUSG00000073643	Wdfy1	WD repeat and FYVE domain containing 1	1.12e+00	3.83e-03
ENSMUST00000113513	ENSMUSG00000073643	Wdfy1	WD repeat and FYVE domain containing 1	1.12e+00	3.83e-03
ENSMUST00000113514	ENSMUSG00000073643	Wdfy1	WD repeat and FYVE domain containing 1	1.12e+00	3.83e-03
ENSMUST00000113515	ENSMUSG00000073643	Wdfy1	WD repeat and FYVE domain containing 1	1.12e+00	3.83e-03
ENSMUST00000187005	ENSMUSG00000073643	Wdfy1	WD repeat and FYVE domain containing 1	1.08e+00	3.73e-03
ENSMUST00000203150	ENSMUSG00000030361	Klrb1a	killer cell lectin-like receptor subfamily B member 1A	1.06e+00	4.21e-02
ENSMUST00000172486	ENSMUSG00000015222	Map2	microtubule-associated protein 2	1.05e+00	8.34e-03
ENSMUST00000135885	ENSMUSG00000029095	Ablim2	actin-binding LIM protein 2	1.04e+00	1.43e-04
ENSMUST00000186702	ENSMUSG00000041460	Cacna2d4	calcium channel, voltage-dependent, alpha 2/delta subunit 4	1.03e+00	5.87e-03
ENSMUST00000131920	ENSMUSG00000023267	Gabrr2	gamma-aminobutyric acid (GABA) C receptor, subunit rho 2	1.03e+00	3.48e-02
ENSMUST00000171262	ENSMUSG00000006711	D130043K22Rik	RIKEN cDNA D130043K22 gene	1.01e+00	1.44e-02
ENSMUST00000186394	ENSMUSG00000074109	Mrgprx2	MAS-related GPR, member X2	1.00e+00	8.34e-03
ENSMUST00000103483	ENSMUSG00000076674	Ighv3-8	immunoglobulin heavy variable V3-8	9.86e-01	3.05e-01
ENSMUST00000185329	ENSMUSG00000025932	Eya1	EYA transcriptional coactivator and phosphatase 1	9.84e-01	4.82e-02
ENSMUST00000040361	ENSMUSG00000039347	Atp6v0e2	ATPase, H+ transporting, lysosomal V0 subunit E2	9.79e-01	5.14e-03
ENSMUST00000136987	ENSMUSG00000043587	Pxylp1	2-phosphoxylose phosphatase 1	9.68e-01	9.28e-04
ENSMUST00000144697	ENSMUSG00000026999	Nup35	nucleoporin 35	9.67e-01	1.95e-02
ENSMUST00000153129	ENSMUSG00000028047	Thbs3	thrombospondin 3	9.65e-01	4.39e-02
ENSMUST00000103350	ENSMUSG00000076549	Igkv4-68	immunoglobulin kappa variable 4-68	9.63e-01	1.02e-02
ENSMUST00000137290	ENSMUSG00000031698	Mylk3	myosin light chain kinase 3	9.62e-01	1.37e-02
ENSMUST00000169797	ENSMUSG00000037849	Ifi206	interferon activated gene 206	9.49e-01	7.66e-02
ENSMUST00000155275	ENSMUSG00000021596	Mctp1	multiple C2 domains, transmembrane 1	9.39e-01	6.10e-03
ENSMUST00000162154	ENSMUSG00000022148	Fyb	FYN binding protein	9.37e-01	1.19e-02
ENSMUST00000161947	ENSMUSG00000022148	Fyb	FYN binding protein	9.29e-01	7.54e-03
ENSMUST00000190151	ENSMUSG00000021209	Ppp4r4	protein phosphatase 4, regulatory subunit 4	9.08e-01	7.42e-03
ENSMUST00000172478	ENSMUSG00000074369	Obox2	oocyte specific homeobox 2	8.92e-01	1.01e-02
ENSMUST00000174076	ENSMUSG00000074369	Obox2	oocyte specific homeobox 2	8.92e-01	1.01e-02
ENSMUST00000174305	ENSMUSG00000074369	Obox2	oocyte specific homeobox 2	8.92e-01	1.01e-02
ENSMUST00000149336	ENSMUSG00000029651	Mtus2	microtubule associated tumor suppressor candidate 2	8.84e-01	5.44e-03
ENSMUST00000194041	ENSMUSG00000026587	Astn1	astrotactin 1	8.62e-01	1.16e-04
ENSMUST00000213557	ENSMUSG00000071317	Bves	blood vessel epicardial substance	8.61e-01	1.04e-02
ENSMUST00000015576	ENSMUSG00000022226	Mcpt2	mast cell protease 2	8.59e-01	7.10e-04
ENSMUST00000207685	ENSMUSG00000035177	Nlrp2	NLR family, pyrin domain containing 2	8.50e-01	6.82e-04
ENSMUST00000204277	ENSMUSG00000039347	Atp6v0e2	ATPase, H+ transporting, lysosomal V0 subunit E2	8.50e-01	3.72e-03
ENSMUST00000201736	ENSMUSG00000094719	Gm5108	predicted gene 5108	8.48e-01	1.46e-03
ENSMUST00000135355	ENSMUSG00000021645	Smn1	survival motor neuron 1	8.46e-01	4.36e-02
ENSMUST00000142251	ENSMUSG00000051747	Ttn	titin	8.43e-01	8.03e-02
ENSMUST00000195849	ENSMUSG00000034837	Gnat1	guanine nucleotide binding protein, alpha transducing 1	8.41e-01	2.01e-02
ENSMUST00000202984	ENSMUSG00000006641	Slc5a6	solute carrier family 5 (sodium-dependent vitamin transporter), member 6	8.29e-01	1.50e-02
ENSMUST00000022836	ENSMUSG00000022227	Mcpt1	mast cell protease 1	8.28e-01	1.36e-02
ENSMUST00000176196	ENSMUSG00000032595	Cdhr4	cadherin-related family member 4	8.25e-01	1.70e-02
ENSMUST00000177093	ENSMUSG00000032595	Cdhr4	cadherin-related family member 4	8.25e-01	1.70e-02
ENSMUST00000141085	ENSMUSG00000041216	Clvs1	clavesin 1	8.23e-01	3.49e-02
ENSMUST00000095450	ENSMUSG00000071178	Serpina1b	serine (or cysteine) preptidase inhibitor, clade A, member 1B	8.20e-01	2.66e-02
ENSMUST00000164454	ENSMUSG00000071178	Serpina1b	serine (or cysteine) preptidase inhibitor, clade A, member 1B	8.20e-01	2.66e-02
ENSMUST00000186166	ENSMUSG00000071178	Serpina1b	serine (or cysteine) preptidase inhibitor, clade A, member 1B	8.20e-01	2.66e-02
ENSMUST00000195095	ENSMUSG00000104098	AA619741	expressed sequence AA619741	8.18e-01	3.66e-02
ENSMUST00000168044	ENSMUSG00000043557	Mdga1	MAM domain containing glycosylphosphatidylinositol anchor 1	8.15e-01	2.99e-04
ENSMUST00000189541	ENSMUSG00000016918	Sulf1	sulfatase 1	8.13e-01	1.79e-02
ENSMUST00000172308	ENSMUSG00000072731	Gm3715	predicted gene 3715	8.08e-01	3.60e-03
ENSMUST00000190082	ENSMUSG00000026246	Alppl2	alkaline phosphatase, placental-like 2	8.08e-01	1.82e-02
ENSMUST00000148715	ENSMUSG00000009246	Trpm5	transient receptor potential cation channel, subfamily M, member 5	8.06e-01	4.03e-02
ENSMUST00000191403	ENSMUSG00000099826	Scgb2b10	secretoglobin, family 2B, member 10	8.04e-01	1.00e-01
ENSMUST00000103323	ENSMUSG00000076522	Igkv16-104	immunoglobulin kappa variable 16-104	8.00e-01	1.14e-01

Supplementary Table S7

Ct values of genes analyzed in the proximal colonic epithelium of the indicated mice 7 days after tamoxifen administration; the values were normalized to β-actin gene expression; SD, standard deviation.

	Control		*Apc* $^{KO/KO}$ *Msx1* $^{+/+}$		*Apc* $^{KO/KO}$ *Msx1* $^{KO/KO}$	
gene name	Ct values	SD	Ct values	SD	Ct values	SD
Bves	37.99	3.99	40.51	1.40	40.44	2.59
Mdga1	31.68	1.22	33.49	0.55	32.96	0.91
Mtus2	32.68	1.15	35.65	0.84	35.19	0.23
Mylk3	37.07	1.51	39.09	0.69	38.71	1.37
Msx1	36.40	1.51	26.64	0.50	30.14	0.86
Msx2	36.94	1.06	27.23	0.62	26.55	0.33
Slc5a6	26.08	1.11	28.40	0.31	26.72	1.07
Stk32b	34.16	0.87	34.69	0.41	27.41	0.49
Trpm	39.73	3.53	44.12	3.19	42.30	2.30
Ttn	40.55	1.27	40.29	0.61	40.81	2.14
Ubb	18.27	0.94	19.63	0.34	19.30	0.29

Supplementary Table S2

Differentialy expressed genes (|logFC| ≥ 1, q-value < 0.05) in the colonic epithelium 2 days upon Apc depletion compared to tissue with intact Apc

ENTREZ	SYMBOL	GENENAME	logFC	q-value
626708	Defa26	defensin, alpha, 26	2.38	5.00E-04
13218	Defa-rs1	defensin, alpha, related sequence 1	2.34	0.028
68009	ILMN_196346	Mus musculus defensin related cryptdin 20 (Defcr20), mRNA.	2.28	0.0037
13239	ILMN_196558	Mus musculus defensin related cryptdin 5 (Defcr5), mRNA.	2.11	0.026
68009	ILMN_196346	Mus musculus defensin related cryptdin 20 (Defcr20), mRNA.	2.01	0.0037
100044291	ILMN_221210	PREDICTED: Mus musculus hypothetical protein LOC100044291 (LOC100044291), mRNA.	1.99	0.016
17110	Lyz1	lysozyme 1	1.88	0.0096
13216	ILMN_196581	Mus musculus defensin, alpha 1 (Defa1), mRNA.	1.86	0.016
13240	Defa6	defensin, alpha, 6	1.82	0.031
17110	Lyz1	lysozyme 1	1.63	0.028
17748	Mt1	metallothionein 1	1.35	0.031
11551	ILMN_190996	Mus musculus adrenergic receptor, alpha 2a (Adra2a), mRNA.	1.28	0.026
23945	Mgll	monoglyceride lipase	1.25	0.031
213391	Rassf4	Ras association (RalGDS/AF-6) domain family member 4	1.02	0.031
12231	Btn1a1	butyrophilin, subfamily 1, member A1	-1.07	0.016
16987	ILMN_187484	Mus musculus lanosterol synthase (Lss), mRNA.	-1.12	0.016
64177	Trpv6	transient receptor potential cation channel, subfamily V, member 6	-1.33	0.04

Supplementary Table S2

Differentialy expressed genes (|logFC| ≥ 1, q-value < 0.05) in the colonic epithelium 4 days upon Apc depletion compared to tissue with intact Apc

ENTREZ	SYMBOL	GENENAME	logFC	q-value
12709.00	ILMN_193661	Mus musculus creatine kinase, brain (Ckb), mRNA.	3.86	2.50E-08
74186.00	Ccdc3	coiled-coil domain containing 3	3.17	6.40E-10
20568.00	Slpi	secretory leukocyte peptidase inhibitor	3.09	0.00018
73710.00	Tubb2b	tubulin, beta 2B	3.03	2.50E-09
17329.00	Cxcl9	chemokine (C-X-C motif) ligand 9	2.89	3.80E-05
66141.00	Ifitm3	interferon induced transmembrane protein 3	2.86	8.10E-08
15945.00	ILMN_253583	Mus musculus chemokine (C-X-C motif) ligand 10 (Cxcl10), mRNA.	2.7	0.0024
213948.00	Atg9b	ATG9 autophagy related 9 homolog B (S. cerevisiae)	2.58	1.90E-05
14969.00	H2-Eb1	histocompatibility 2, class II antigen E beta	2.43	0.00014
14570.00	Arhgdig	Rho GDP dissociation inhibitor (GDI) gamma	2.42	3.10E-07
15930.00	Ido1	indoleamine 2,3-dioxygenase 1	2.34	0.012
15937.00	Ier3	immediate early response 3	2.22	1.70E-07
16010.00	Igfbp4	insulin-like growth factor binding protein 4	2.21	1.30E-06
26897.00	ILMN_223756	Mus musculus acyl-CoA thioesterase 1 (Acot1), mRNA.	2.21	8.00E-06
19752.00	Rnase1	ribonuclease, RNase A family, 1 (pancreatic)	2.19	2.60E-05
66214.00	1190002H23Rik	RIKEN cDNA 1190002H23 gene	2.17	0.00017
100047619.00	ILMN_219663	PREDICTED: Mus musculus similar to solute carrier family 7 (cationic amino acid transporter, y+ system), member 5 (LOC100047619), misc RNA.	2.16	9.20E-07
14160.00	Lgr5	leucine rich repeat containing G protein coupled receptor 5	2.13	2.40E-07
270152.00	Amica1	adhesion molecule, interacts with CXADR antigen 1	2.12	1.40E-06
14609.00	Gja1	gap junction protein, alpha 1	1.99	5.50E-05
11459.00	Acta1	actin, alpha 1, skeletal muscle	1.99	4.90E-05
27280.00	Phlda3	pleckstrin homology-like domain, family A, member 3	1.98	0.00039
17218.00	Mcm5	minichromosome maintenance deficient 5, cell division cycle 46 (S. cerevisiae)	1.97	2.80E-08
16145.00	Igtp	interferon gamma induced GTPase	1.97	0.025
320685.00	Dctd	dCMP deaminase	1.95	3.10E-05
328162.00	Trmt61a	tRNA methyltransferase 61 homolog A (S. cerevisiae)	1.95	2.50E-06
100608.00	Noc4l	nucleolar complex associated 4 homolog (S. cerevisiae)	1.93	4.00E-06
17067.00	Ly6c1	lymphocyte antigen 6 complex, locus C1	1.93	0.00015
11925.00	Neurog3	neurogenin 3	1.88	9.30E-06
72462.00	Rrp1b	ribosomal RNA processing 1 homolog B (S. cerevisiae)	1.87	9.80E-07
13401.00	Dmwd	dystrophia myotonica-containing WD repeat motif	1.87	1.70E-07
30927.00	Snai3	snail homolog 3 (Drosophila)	1.86	2.00E-08
27280.00	Phlda3	pleckstrin homology-like domain, family A, member 3	1.85	0.00019
14028.00	Evx1	even skipped homeotic gene 1 homolog	1.85	2.70E-07
68915.00	Vars2	valyl-tRNA synthetase 2, mitochondrial (putative)	1.84	1.10E-07
26897.00	Acot1	acyl-CoA thioesterase 1	1.82	3.90E-06
102614.00	Rpp25	ribonuclease P 25 subunit (human)	1.8	4.80E-07
17216.00	Mcm2	minichromosome maintenance deficient 2 mitotin (S. cerevisiae)	1.8	5.10E-08
21677.00	Tead2	TEA domain family member 2	1.79	1.80E-06
16673.00	Krt36	keratin 36	1.79	6.90E-10
17173.00	Ascl2	achaete-scute complex homolog 2 (Drosophila)	1.79	4.40E-05
20361.00	Sema7a	sema domain, immunoglobulin domain (Ig), and GPI membrane anchor, (semaphorin) 7A	1.78	1.60E-05
110749.00	ILMN_215876	Mus musculus chromatin assembly factor 1, subunit B (p60) (Chaf1b), mRNA.	1.78	3.50E-07
64406.00	Sp5	trans-acting transcription factor 5	1.77	5.80E-07
18432.00	Mybbp1a	MYB binding protein (P160) 1a	1.77	7.10E-06
171212.00	Galnt10	UDP-N-acetyl-alpha-D-galactosamine:polypeptide N-acetylgalactosaminyltransferase 10	1.76	3.90E-08
18383.00	Tnfrsf11b	tumor necrosis factor receptor superfamily, member 11b (osteoprotegerin)	1.75	8.50E-06
100608.00	ILMN_209940	Mus musculus nucleolar complex associated 4 homolog (S. cerevisiae) (Noc4l), mRNA.	1.75	2.20E-06
69902.00	Mrto4	MRT4, mRNA turnover 4, homolog (S. cerevisiae)	1.75	5.60E-07
15469.00	Prmt1	protein arginine N-methyltransferase 1	1.75	4.80E-07
18780.00	Pla2g2a	phospholipase A2, group IIA (platelets, synovial fluid)	1.74	0.00015
17215.00	ILMN_213080	Mus musculus minichromosome maintenance deficient 3 (S. cerevisiae) (Mcm3), mRNA.	1.74	2.50E-08
12505.00	Cd44	CD44 antigen	1.74	3.60E-06
51797.00	Ctps	cytidine 5'-triphosphate synthase	1.73	3.20E-06
22321.00	Vars	valyl-tRNA synthetase	1.72	6.20E-08
14961.00	H2-Ab1	histocompatibility 2, class II antigen A, beta 1	1.72	0.0022
22041.00	Trf	transferrin	1.71	1.10E-06
14961.00	H2-Ab1	histocompatibility 2, class II antigen A, beta 1	1.71	0.0017
17865.00	ILMN_212854	Mus musculus myeloblastosis oncogene-like 2 (Mybl2), mRNA.	1.7	6.40E-10
71805.00	Nup93	nucleoporin 93	1.69	1.70E-06
14998.00	ILMN_224639	Mus musculus histocompatibility 2, class II, locus DMa (H2-DMa), mRNA.	1.69	0.0018
73710.00	2410129E14Rik	Mus musculus tubulin, beta 2b (Tubb2b), mRNA.	1.68	1.90E-05
20810.00	Srm	spermidine synthase	1.68	5.80E-05

208638.00	ILMN_211336	Mus musculus solute carrier family 25, member 38 (Slc25a38), mRNA.	1.67	6.20E-08
17319.00	Mif	macrophage migration inhibitory factor	1.67	2.50E-08
15894.00	Icam1	intercellular adhesion molecule 1	1.67	0.00034
20810.00	Srm	spermidine synthase	1.66	2.50E-05
100340.00	ILMN_210171	Mus musculus sphingomyelin phosphodiesterase, acid-like 3B (Smpdl3b), mRNA.	1.66	1.00E-04
107272.00	Psat1	phosphoserine aminotransferase 1	1.66	3.20E-05
67177.00	Cdt1	chromatin licensing and DNA replication factor 1	1.66	1.50E-06
16149.00	Cd74	CD74 antigen (invariant polypeptide of major histocompatibility complex, class II antigen-associated)	1.66	0.013
51800.00	Bok	BCL2-related ovarian killer protein	1.66	9.60E-06
22154.00	Tubb5	tubulin, beta 5	1.65	7.30E-07
66102.00	Cxcl16	chemokine (C-X-C motif) ligand 16	1.65	0.00027
233066.00	AI428936	expressed sequence AI428936	1.65	9.20E-07
16149.00	Cd74	CD74 antigen (invariant polypeptide of major histocompatibility complex, class II antigen-associated)	1.64	0.0081
27279.00	Tnfrsf12a	tumor necrosis factor receptor superfamily, member 12a	1.63	0.0015
20810.00	Srm	spermidine synthase	1.63	8.00E-06
68147.00	Gar1	GAR1 ribonucleoprotein homolog (yeast)	1.63	1.80E-06
29870.00	Gtse1	G two S phase expressed protein 1	1.63	8.90E-06
226419.00	Dyrk3	dual-specificity tyrosine-(Y)-phosphorylation regulated kinase 3	1.63	0.00011
11792.00	Apex1	apurinic/apyrimidinic endonuclease 1	1.62	2.10E-06
20509.00	Slc19a1	solute carrier family 19 (sodium/hydrogen exchanger), member 1	1.62	1.10E-06
18817.00	Plk1	polo-like kinase 1 (Drosophila)	1.62	3.80E-05
17220.00	Mcm7	minichromosome maintenance deficient 7 (S. cerevisiae)	1.61	9.60E-05
12505.00	Cd44	CD44 antigen	1.61	7.10E-06
64424.00	ILMN_189434	Mus musculus polymerase (RNA) I polypeptide E (Polr1e), mRNA.	1.6	8.00E-06
13836.00	Epha2	Eph receptor A2	1.6	0.00026
51797.00	Ctps	cytidine 5'-triphosphate synthase	1.6	4.80E-07
68337.00	Crip2	cysteine rich protein 2	1.6	3.40E-06
217995.00	ILMN_214317	Mus musculus HEAT repeat containing 1 (Heatr1), mRNA.	1.59	7.70E-07
20595.00	Smn1	survival motor neuron 1	1.58	6.50E-07
30877.00	Gnl3	guanine nucleotide binding protein-like 3 (nucleolar)	1.58	2.10E-05
14114.00	Fbln1	fibulin 1	1.58	1.10E-05
211949.00	Spsb4	splA/ryanodine receptor domain and SOCS box containing 4	1.57	5.70E-06
16010.00	Igfbp4	insulin-like growth factor binding protein 4	1.57	0.00058
30877.00	Gnl3	guanine nucleotide binding protein-like 3 (nucleolar)	1.57	6.10E-06
20019.00	Polr1a	polymerase (RNA) I polypeptide A	1.56	3.50E-07
12006.00	Axin2	axin2	1.56	8.50E-05
70024.00	Mcm10	minichromosome maintenance deficient 10 (S. cerevisiae)	1.55	8.10E-08
17110.00	Lyz1	lysozyme 1	1.55	0.0053
353156.00	Egfl7	EGF-like domain 7	1.55	2.90E-06
66953.00	Cdca7	cell division cycle associated 7	1.55	8.00E-06
101612.00	Grwd1	glutamate-rich WD repeat containing 1	1.54	2.20E-06
27221.00	Chaf1a	chromatin assembly factor 1, subunit A (p150)	1.54	2.00E-07
244886.00	AI118078	expressed sequence AI118078	1.54	1.10E-05
23825.00	Banf1	barrier to autointegration factor 1	1.53	4.80E-07
19385.00	Ranbp1	RAN binding protein 1	1.52	3.50E-07
100019.00	Mdn1	midasin homolog (yeast)	1.52	2.00E-05
17228.00	Cma1	chymase 1, mast cell	1.52	1.60E-05
12545.00	Cdc7	cell division cycle 7 (S. cerevisiae)	1.52	1.20E-07
66350.00	Pla2g12a	phospholipase A2, group XIIA	1.51	6.80E-06
13639.00	Efna4	ephrin A4	1.51	3.10E-05
11746.00	Anxa4	annexin A4	1.51	0.034
72462.00	Rrp1b	ribosomal RNA processing 1 homolog B (S. cerevisiae)	1.5	0.0037
22113.00	Phlda2	pleckstrin homology-like domain, family A, member 2	1.49	0.00031
100044829.00	ILMN_209238	PREDICTED: Mus musculus similar to Fibrillarin, transcript variant 1 (LOC100044829), mRNA.	1.49	6.40E-07
17217.00	Mcm4	minichromosome maintenance deficient 4 homolog (S. cerevisiae)	1.48	1.70E-06
100046741.00	ILMN_190874	PREDICTED: Mus musculus similar to red-1 (LOC100046741), mRNA.	1.47	1.20E-06
102657.00	Cd276	CD276 antigen	1.47	1.00E-05
19183.00	Psmc3ip	proteasome (prosome, macropain) 26S subunit, ATPase 3, interacting protein	1.46	1.00E-04
17219.00	Mcm6	minichromosome maintenance deficient 6 (MIS5 homolog, S. pombe) (S. cerevisiae)	1.46	3.70E-07
70024.00	Mcm10	minichromosome maintenance deficient 10 (S. cerevisiae)	1.46	1.20E-05
22321.00	Vars	valyl-tRNA synthetase	1.45	7.10E-06
103733.00	ILMN_184611	Mus musculus tubulin, gamma 1 (Tubg1), mRNA.	1.45	1.00E-04
22059.00	Trp53	transformation related protein 53	1.45	6.80E-06
56390.00	Sssca1	Sjogren's syndrome/scleroderma autoantigen 1 homolog (human)	1.45	1.20E-05
56390.00	Sssca1	Sjogren's syndrome/scleroderma autoantigen 1 homolog (human)	1.45	7.10E-07
641240.00	ILMN_210638	PREDICTED: Mus musculus similar to MHC class II antigen beta chain (LOC641240), mRNA.	1.45	0.013
14156.00	Fen1	flap structure specific endonuclease 1	1.45	2.40E-06
51797.00	Ctps	cytidine 5'-triphosphate synthase	1.45	2.50E-05

20641.00	Snrpd1	small nuclear ribonucleoprotein D1	1.44	3.00E-06
56520.00	Nme4	non-metastatic cells 4, protein expressed in	1.44	1.00E-05
11475.00	Acta2	actin, alpha 2, smooth muscle, aorta	1.44	0.00071
72821.00	Scn2b	sodium channel, voltage-gated, type II, beta	1.43	4.90E-05
19366.00	Rad54l	RAD54 like (S. cerevisiae)	1.43	3.10E-07
16918.00	Mycl1	v-myc myelocytomatosis viral oncogene homolog 1, lung carcinoma derived (avian)	1.43	9.60E-05
66902.00	Mtap	methylthioadenosine phosphorylase	1.43	2.90E-06
17089.00	Lyar	Ly1 antibody reactive clone	1.43	9.60E-06
233876.00	Hirip3	HIRA interacting protein 3	1.43	2.00E-07
14733.00	Gpc1	glypican 1	1.43	2.40E-06
56505.00	Ruvbl1	RuvB-like protein 1	1.42	4.60E-07
57028.00	Pdxp	pyridoxal (pyridoxine, vitamin B6) phosphatase	1.42	1.00E-06
56520.00	Nme4	non-metastatic cells 4, protein expressed in	1.42	1.20E-05
18432.00	Mybbp1a	MYB binding protein (P160) 1a	1.42	0.00016
67242.00	Gemin6	gem (nuclear organelle) associated protein 6	1.42	3.70E-06
11461.00	Actb	actin, beta	1.42	0.029
18140.00	Uhrf1	ubiquitin-like, containing PHD and RING finger domains, 1	1.41	3.20E-06
54563.00	Nup210	nucleoporin 210	1.41	6.50E-06
17110.00	Lyz1	lysozyme 1	1.41	0.00081
15505.00	Hsph1	heat shock 105kDa/110kDa protein 1	1.41	3.50E-05
100102.00	Pcsk9	proprotein convertase subtilisin/kexin type 9	1.4	8.10E-05
67134.00	Nop56	NOP56 ribonucleoprotein homolog (yeast)	1.4	9.40E-05
14961.00	H2-Ab1	histocompatibility 2, class II antigen A, beta 1	1.4	0.0076
227358.00	Fam132b	family with sequence similarity 132, member B	1.4	6.50E-06
110956.00	D17H6S56E-5	DNA segment, Chr 17, human D6S56E 5	1.39	3.50E-05
434341.00	Nlrc5	NLR family, CARD domain containing 5	1.39	0.013
73284.00	Ddit4l	DNA-damage-inducible transcript 4-like	1.38	0.0017
12443.00	Ccnd1	cyclin D1	1.38	3.50E-07
381101.00	BC048355	cDNA sequence BC048355	1.38	1.90E-05
67236.00	Cinp	cyclin-dependent kinase 2 interacting protein	1.38	3.20E-06
52530.00	Nhp2	NHP2 ribonucleoprotein homolog (yeast)	1.37	1.60E-05
105837.00	Mtbp	Mdm2, transformed 3T3 cell double minute p53 binding protein	1.37	6.20E-08
13555.00	E2f1	E2F transcription factor 1	1.37	3.50E-07
70333.00	Cd3eap	CD3E antigen, epsilon polypeptide associated protein	1.37	0.00012
21354.00	Tap1	transporter 1, ATP-binding cassette, sub-family B (MDR/TAP)	1.36	0.0067
	ILMN_187520		1.36	9.60E-05
67671.00	Rpl38	ribosomal protein L38	1.36	0.00025
59028.00	Rcl1	RNA terminal phosphate cyclase-like 1	1.36	4.90E-05
100044103.00	ILMN_210607	PREDICTED: Mus musculus similar to mKIAA1645 protein (LOC100044103), mRNA.	1.36	6.90E-05
12544.00	Cdc45	cell division cycle 45 homolog (S. cerevisiae)	1.36	2.50E-06
14450.00	Gart	phosphoribosylglycinamide formyltransferase	1.35	3.40E-05
22051.00	Trip6	thyroid hormone receptor interactor 6	1.34	8.30E-05
21915.00	Dtymk	deoxythymidylate kinase	1.34	2.00E-04
330671.00	B4galnt4	beta-1,4-N-acetyl-galactosaminyl transferase 4	1.34	1.90E-05
11792.00	Apex1	apurinic/apyrimidinic endonuclease 1	1.34	0.00061
66965.00	ILMN_198892	Mus musculus RIKEN cDNA 2310061F22 gene (2310061F22Rik), mRNA.	1.34	4.60E-07
98170.00	Tmem132a	transmembrane protein 132A	1.33	7.50E-05
98170.00	Tmem132a	transmembrane protein 132A	1.33	2.20E-06
245688.00	ILMN_215499	Mus musculus retinoblastoma binding protein 7 (Rbbp7), mRNA.	1.33	2.00E-04
56361.00	Pus1	pseudouridine synthase 1	1.33	2.30E-06
105837.00	Mtbp	Mdm2, transformed 3T3 cell double minute p53 binding protein	1.33	2.10E-06
14999.00	H2-DMb1	histocompatibility 2, class II, locus Mb1	1.33	0.0048
14038.00	Expi	extracellular proteinase inhibitor	1.33	1.70E-05
109857.00	Cbr3	carbonyl reductase 3	1.33	0.0076
406217.00	ILMN_231589	Mus musculus brain expressed gene 4 (Bex4), mRNA.	1.33	9.60E-06
20641.00	Snrpd1	small nuclear ribonucleoprotein D1	1.32	4.50E-06
56505.00	Ruvbl1	RuvB-like protein 1	1.32	1.50E-06
17304.00	Mfge8	milk fat globule-EGF factor 8 protein	1.32	0.00018
12028.00	Bax	BCL2-associated X protein	1.32	0.00012
56279.00	Fam69b	family with sequence similarity 69, member B	1.32	6.10E-06
21681.00	Thoc4	THO complex 4	1.31	2.10E-05
17427.00	Mns1	meiosis-specific nuclear structural protein 1	1.31	3.30E-06
15361.00	Hmga1	high mobility group AT-hook 1	1.31	0.00044
217995.00	ILMN_214317	Mus musculus HEAT repeat containing 1 (Heatr1), mRNA.	1.31	0.00012
12265.00	Ciita	class II transactivator	1.31	0.002
71449.00	ILMN_221329	Mus musculus RIKEN cDNA 5630401D24 gene (5630401D24Rik), mRNA.	1.31	0.00022
93840.00	Vangl2	vang-like 2 (van gogh, Drosophila)	1.3	4.60E-05
78294.00	Rps27a	ribosomal protein S27A	1.3	1.70E-05

13639.00	Efna4	ephrin A4	1.3	3.50E-05
21983.00	Tpbg	trophoblast glycoprotein	1.29	2.60E-06
21973.00	Top2a	topoisomerase (DNA) II alpha	1.29	0.00026
108037.00	Shmt2	serine hydroxymethyltransferase 2 (mitochondrial)	1.29	6.70E-06
67223.00	Rrp15	ribosomal RNA processing 15 homolog (S. cerevisiae)	1.29	3.40E-05
12443.00	Ccnd1	cyclin D1	1.29	5.40E-05
12153.00	Bmp1	bone morphogenetic protein 1	1.29	2.40E-05
18971.00	Pold1	polymerase (DNA directed), delta 1, catalytic subunit	1.28	4.90E-05
18969.00	Pola2	polymerase (DNA directed), alpha 2	1.28	6.40E-06
27756.00	Lsm2	LSM2 homolog, U6 small nuclear RNA associated (S. cerevisiae)	1.28	3.40E-06
116701.00	ILMN_211983	Mus musculus fibroblast growth factor receptor-like 1 (Fgfrl1), mRNA.	1.28	1.10E-05
214901.00	Chtf18	CTF18, chromosome transmission fidelity factor 18 homolog (S. cerevisiae)	1.28	7.60E-06
72640.00	Mex3a	mex3 homolog A (C. elegans)	1.27	2.40E-06
17357.00	Marcksl1	MARCKS-like 1	1.27	0.00023
110033.00	Kif22	kinesin family member 22	1.27	1.00E-05
110006.00	ILMN_221841	Mus musculus glucuronidase, beta (Gusb), mRNA.	1.27	2.20E-05
14450.00	Gart	phosphoribosylglycinamide formyltransferase	1.27	9.60E-05
104156.00	Etv5	ets variant gene 5	1.27	0.00019
12144.00	Blm	Bloom syndrome, RecQ helicase-like	1.27	1.40E-05
71242.00	Spata24	spermatogenesis associated 24	1.27	7.90E-05
214572.00	Prmt7	protein arginine N-methyltransferase 7	1.26	1.10E-05
53605.00	ILMN_214137	Mus musculus nucleosome assembly protein 1-like 1 (Nap1l1), mRNA.	1.26	1.30E-05
17869.00	Myc	myelocytomatosis oncogene	1.26	0.00011
17110.00	Lyz1	lysozyme 1	1.26	0.0024
17228.00	Cma1	chymase 1, mast cell	1.26	6.50E-05
52276.00	Cdca8	cell division cycle associated 8	1.26	9.30E-06
69928.00	Apitd1	apoptosis-inducing, TAF9-like domain 1	1.26	0.00026
80914.00	Uck2	uridine-cytidine kinase 2	1.25	1.80E-05
104367.00	Snora65	small nucleolar RNA, H/ACA box 65	1.25	4.00E-05
245688.00	ILMN_215499	Mus musculus retinoblastoma binding protein 7 (Rbbp7), mRNA.	1.25	0.0014
68294.00	Mfsd10	major facilitator superfamily domain containing 10	1.25	1.60E-05
	ILMN_208668		1.25	8.90E-06
66570.00	Cenpm	centromere protein M	1.25	3.10E-07
237038.00	Nox1	NADPH oxidase 1	1.24	0.0092
18102.00	ILMN_209549	Mus musculus non-metastatic cells 1, protein (NM23A) expressed in (Nme1), mRNA.	1.24	1.80E-06
108673.00	Ccdc86	coiled-coil domain containing 86	1.24	0.00035
57315.00	Wdr46	WD repeat domain 46	1.23	6.10E-05
72787.00	Tmem48	transmembrane protein 48	1.23	1.20E-05
236539.00	ILMN_226456	Mus musculus 3-phosphoglycerate dehydrogenase (Phgdh), mRNA.	1.23	0.00052
234865.00	Nup133	nucleoporin 133	1.23	1.80E-05
67711.00	Nsmce1	non-SMC element 1 homolog (S. cerevisiae)	1.23	2.00E-04
27993.00	Imp4	IMP4, U3 small nucleolar ribonucleoprotein, homolog (yeast)	1.23	7.10E-06
434632.00	BC085271	cDNA sequence BC085271	1.23	0.00032
56412.00	2610024G14Rik	RIKEN cDNA 2610024G14 gene	1.23	1.80E-05
68926.00	Ubap2	ubiquitin-associated protein 2	1.22	3.00E-06
20174.00	Ruvbl2	RuvB-like protein 2	1.22	3.50E-06
19891.00	Rpa2	replication protein A2	1.22	0.00017
59028.00	Rcl1	RNA terminal phosphate cyclase-like 1	1.22	0.00046
106582.00	Nrm	nurim (nuclear envelope membrane protein)	1.22	2.00E-06
17748.00	Mt1	metallothionein 1	1.22	0.00092
15114.00	Hap1	huntingtin-associated protein 1	1.22	0.00011
66570.00	Cenpm	centromere protein M	1.22	2.10E-06
59053.00	Fam203a	family with sequence similarity 203, member A	1.22	1.00E-05
17299.00	Mettl1	methyltransferase like 1	1.21	0.00049
110006.00	Gusb	glucuronidase, beta	1.21	7.80E-05
14630.00	Gclm	glutamate-cysteine ligase, modifier subunit	1.21	2.70E-06
69639.00	Exosc8	exosome component 8	1.21	1.80E-05
80914.00	Uck2	uridine-cytidine kinase 2	1.2	1.50E-05
110816.00	ILMN_212496	Mus musculus PWP2 periodic tryptophan protein homolog (yeast) (Pwp2), mRNA.	1.2	0.00013
654467.00	ILMN_234090	Mus musculus heterogeneous nuclear ribonucleoprotein A1 pseudogene (LOC654467) on chromosome 9.	1.2	0.00031
209737.00	ILMN_212864	Mus musculus kinesin family member 15 (Kif15), mRNA.	1.2	6.50E-06
272359.00	Irf2bp1	interferon regulatory factor 2 binding protein 1	1.2	1.30E-05
14916.00	Guca2b	guanylate cyclase activator 2b (retina)	1.2	0.00029
381903.00	Alg8	asparagine-linked glycosylation 8 homolog (yeast, alpha-1,3-glucosyltransferase)	1.2	5.60E-06
104732.00	4930427A07Rik	RIKEN cDNA 4930427A07 gene	1.2	0.00013
72657.00	2700094K13Rik	RIKEN cDNA 2700094K13 gene	1.2	1.60E-05
57257.00	Vav3	vav 3 oncogene	1.19	2.40E-06
20133.00	ILMN_231868	Mus musculus ribonucleotide reductase M1 (Rrm1), mRNA.	1.19	8.90E-05

245688.00	ILMN_215499	Mus musculus retinoblastoma binding protein 7 (Rbbp7), mRNA.	1.19	6.00E-04
226519.00	ILMN_232528	Mus musculus laminin, gamma 1 (Lamc1), mRNA.	1.19	0.00037
23834.00	Cdc6	cell division cycle 6 homolog (S. cerevisiae)	1.19	4.80E-06
69719.00	Cad	carbamoyl-phosphate synthetase 2, aspartate transcarbamylase, and dihydroorotase	1.19	0.00035
14025.00	Bcl11a	B-cell CLL/lymphoma 11A (zinc finger protein)	1.19	3.80E-05
74254.00	Gpn1	GPN-loop GTPase 1	1.18	5.50E-05
22256.00	Ung	uracil DNA glycosylase	1.18	1.00E-04
21849.00	Trim28	tripartite motif-containing 28	1.18	2.20E-05
75273.00	Pelp1	proline, glutamic acid and leucine rich protein 1	1.18	0.00014
70572.00	Ipo5	importin 5	1.18	2.90E-06
15312.00	Hmgn1	high mobility group nucleosomal binding domain 1	1.18	1.10E-05
20624.00	Eftud2	elongation factor Tu GTP binding domain containing 2	1.18	1.00E-05
27407.00	Abcf2	ATP-binding cassette, sub-family F (GCN20), member 2	1.18	5.70E-06
56412.00	2610024G14Rik	RIKEN cDNA 2610024G14 gene	1.18	1.30E-05
21781.00	Tfdp1	transcription factor Dp 1	1.17	1.50E-05
27966.00	Rrp9	RRP9, small subunit (SSU) processome component, homolog (yeast)	1.17	0.00013
72151.00	Rfc5	replication factor C (activator 1) 5	1.17	1.10E-06
57785.00	Rangrf	RAN guanine nucleotide release factor	1.17	6.20E-05
19679.00	Pitpnm2	phosphatidylinositol transfer protein, membrane-associated 2	1.17	6.10E-05
17975.00	Ncl	nucleolin	1.17	6.80E-06
16881.00	Lig1	ligase I, DNA, ATP-dependent	1.17	4.20E-05
23886.00	Gdf15	growth differentiation factor 15	1.17	0.042
55932.00	Gbp3	guanylate binding protein 3	1.17	0.019
434858.00	ILMN_233946	Mus musculus predicted gene, EG434858 (EG434858), non-coding RNA.	1.17	0.0046
72082.00	Cyp2c55	cytochrome P450, family 2, subfamily c, polypeptide 55	1.17	0.026
107995.00	Cdc20	cell division cycle 20 homolog (S. cerevisiae)	1.17	0.00023
230991.00	B930041F14Rik	RIKEN cDNA B930041F14 gene	1.17	1.80E-05
71242.00	Spata24	spermatogenesis associated 24	1.17	5.10E-06
28035.00	Usp39	ubiquitin specific peptidase 39	1.16	9.60E-06
63959.00	Slc29a1	solute carrier family 29 (nucleoside transporters), member 1	1.16	0.00045
67824.00	Nmral1	NmrA-like family domain containing 1	1.16	9.60E-06
60441.00	Mrpl38	mitochondrial ribosomal protein L38	1.16	6.70E-05
12462.00	Cct3	chaperonin containing Tcp1, subunit 3 (gamma)	1.16	3.50E-05
330050.00	Fam185a	family with sequence similarity 185, member A	1.16	4.50E-06
22294.00	Uxt	ubiquitously expressed transcript	1.15	8.00E-06
19245.00	Ptp4a3	protein tyrosine phosphatase 4a3	1.15	0.0011
215387.00	Ncaph	non-SMC condensin I complex, subunit H	1.15	0.00052
17938.00	Naca	nascent polypeptide-associated complex alpha polypeptide	1.15	2.10E-06
66973.00	Mrps18b	mitochondrial ribosomal protein S18B	1.15	1.00E-05
	ILMN_199160		1.15	0.0044
16201.00	Ilf3	interleukin enhancer binding factor 3	1.15	3.60E-06
13433.00	Dnmt1	DNA methyltransferase (cytosine-5) 1	1.15	0.00012
18117.00	Cox4nb	COX4 neighbor	1.15	7.60E-06
12740.00	Cldn4	claudin 4	1.15	0.0016
102920.00	Cenpi	centromere protein I	1.15	0.00071
107995.00	Cdc20	cell division cycle 20 homolog (S. cerevisiae)	1.15	0.00046
12181.00	Bop1	block of proliferation 1	1.15	0.00014
23825.00	Banf1	barrier to autointegration factor 1	1.15	9.80E-05
56412.00	2610024G14Rik	RIKEN cDNA 2610024G14 gene	1.15	8.00E-06
21844.00	Tiam1	T-cell lymphoma invasion and metastasis 1	1.14	1.90E-05
67390.00	Rnmtl1	RNA methyltransferase like 1	1.14	6.90E-05
78929.00	ILMN_209245	Mus musculus polymerase (RNA) III (DNA directed) polypeptide H (Polr3h), mRNA.	1.14	9.60E-06
66667.00	Hspbap1	Hspb associated protein 1	1.14	4.00E-05
55927.00	Hes6	hairy and enhancer of split 6 (Drosophila)	1.14	7.60E-06
14776.00	Gpx2	glutathione peroxidase 2	1.14	1.10E-05
14114.00	Fbln1	fibulin 1	1.14	0.00044
76267.00	Fads1	fatty acid desaturase 1	1.14	4.20E-05
71919.00	Rpap3	RNA polymerase II associated protein 3	1.14	0.00011
66912.00	Bzw2	basic leucine zipper and W2 domains 2	1.14	0.00012
11799.00	Birc5	baculoviral IAP repeat-containing 5	1.14	2.00E-05
66131.00	Tipin	timeless interacting protein	1.13	6.10E-05
225608.00	Sh3tc2	SH3 domain and tetratricopeptide repeats 2	1.13	0.00034
20135.00	ILMN_225236	Mus musculus ribonucleotide reductase M2 (Rrm2), mRNA.	1.13	0.00016
26564.00	Ror2	receptor tyrosine kinase-like orphan receptor 2	1.13	0.00058
14208.00	Ppm1g	protein phosphatase 1G (formerly 2C), magnesium-dependent, gamma isoform	1.13	4.00E-05
18972.00	Pold2	polymerase (DNA directed), delta 2, regulatory subunit	1.13	6.60E-06
18221.00	Nudc	nuclear distribution gene C homolog (Aspergillus)	1.13	3.50E-06
18221.00	Nudc	nuclear distribution gene C homolog (Aspergillus)	1.13	0.00019

50927.00	ILMN_211691	Mus musculus nuclear autoantigenic sperm protein (histone-binding) (Nasp), transcript variant 2, mRNA.	1.13	5.50E-05
70082.00	Lysmd2	LysM, putative peptidoglycan-binding, domain containing 2	1.13	0.0014
100047155.00	ILMN_221233	PREDICTED: Mus musculus similar to Small nuclear ribonucleoprotein polypeptide A (LOC100047155), mRNA.	1.13	7.00E-06
100042777.00	ILMN_212781	PREDICTED: Mus musculus similar to human protein homologous to DROER protein (LOC100042777), mRNA.	1.13	2.80E-06
69550.00	Bst2	bone marrow stromal cell antigen 2	1.13	6.20E-05
12144.00	Blm	Bloom syndrome, RecQ helicase-like	1.13	2.10E-05
11799.00	Birc5	baculoviral IAP repeat-containing 5	1.13	0.00037
406217.00	Bex4	brain expressed gene 4	1.13	0.0076
66422.00	Dctpp1	dCTP pyrophosphatase 1	1.13	0.00014
107071.00	Wdr74	WD repeat domain 74	1.12	6.10E-05
66409.00	Rsl1d1	ribosomal L1 domain containing 1	1.12	1.90E-05
28000.00	Prpf19	PRP19/PSO4 pre-mRNA processing factor 19 homolog (S. cerevisiae)	1.12	1.70E-05
11545.00	Parp1	poly (ADP-ribose) polymerase family, member 1	1.12	5.40E-05
677205.00	ILMN_212422	PREDICTED: Mus musculus similar to DEAD (Asp-Glu-Ala-Asp) box polypeptide 18 (LOC677205), misc RNA.	1.12	7.60E-06
105988.00	Espl1	extra spindle poles-like 1 (S. cerevisiae)	1.12	2.20E-05
103551.00	E130012A19Rik	RIKEN cDNA E130012A19 gene	1.12	0.0033
108888.00	Atad3a	ATPase family, AAA domain containing 3A	1.12	4.90E-05
27407.00	Abcf2	ATP-binding cassette, sub-family F (GCN20), member 2	1.12	2.00E-06
22294.00	Uxt	ubiquitously expressed transcript	1.11	3.80E-06
22143.00	Tuba1b	tubulin, alpha 1B	1.11	4.00E-05
268396.00	Sh3pxd2b	SH3 and PX domains 2B	1.11	6.90E-05
100088.00	Rcc1	regulator of chromosome condensation 1	1.11	3.50E-05
71974.00	Prmt3	protein arginine N-methyltransferase 3	1.11	2.10E-05
100043257.00	ILMN_215552	PREDICTED: Mus musculus similar to RNA binding motif protein 3 (LOC100043257), mRNA.	1.11	4.50E-06
57905.00	Isy1	ISY1 splicing factor homolog (S. cerevisiae)	1.11	8.50E-06
93695.00	Gpnmb	glycoprotein (transmembrane) nmb	1.11	0.027
227715.00	Exosc2	exosome component 2	1.11	0.00012
71963.00	Cdca4	cell division cycle associated 4	1.11	1.40E-05
11799.00	Birc5	baculoviral IAP repeat-containing 5	1.11	8.90E-05
170759.00	Atp13a1	ATPase type 13A1	1.11	0.00094
21817.00	Tgm2	transglutaminase 2, C polypeptide	1.1	0.0012
214572.00	Prmt7	protein arginine N-methyltransferase 7	1.1	3.50E-05
27374.00	ILMN_257867	Mus musculus protein arginine N-methyltransferase 5 (Prmt5), mRNA.	1.1	0.00032
56031.00	Ppie	peptidylprolyl isomerase E (cyclophilin E)	1.1	4.40E-05
211548.00	Nomo1	nodal modulator 1	1.1	1.40E-05
97961.00	Nol12	nucleolar protein 12	1.1	2.60E-05
110109.00	Nop2	NOP2 nucleolar protein homolog (yeast)	1.1	0.00034
66978.00	Luc7l	Luc7 homolog (S. cerevisiae)-like	1.1	6.50E-05
55944.00	Eif3d	eukaryotic translation initiation factor 3, subunit D	1.1	0.00014
107995.00	Cdc20	cell division cycle 20 homolog (S. cerevisiae)	1.1	0.00011
71449.00	ILMN_221329	Mus musculus RIKEN cDNA 5630401D24 gene (5630401D24Rik), mRNA.	1.1	8.10E-05
68026.00	2810417H13Rik	RIKEN cDNA 2810417H13 gene	1.1	0.002
232679.00	Zc3hc1	zinc finger, C3HC type 1	1.09	6.70E-05
230734.00	Yrdc	yrdC domain containing (E.coli)	1.09	0.00022
72171.00	Shq1	SHQ1 homolog (S. cerevisiae)	1.09	6.50E-06
107094.00	Rrp12	ribosomal RNA processing 12 homolog (S. cerevisiae)	1.09	7.80E-05
20085.00	Rps19	ribosomal protein S19	1.09	5.40E-05
27374.00	Prmt5	protein arginine N-methyltransferase 5	1.09	4.90E-05
27374.00	Prmt5	protein arginine N-methyltransferase 5	1.09	1.10E-05
56452.00	Orc6	origin recognition complex, subunit 6	1.09	3.90E-06
68294.00	Mfsd10	major facilitator superfamily domain containing 10	1.09	1.10E-05
17083.00	Tmed1	transmembrane emp24 domain containing 1	1.09	5.80E-05
22379.00	Fmnl3	formin-like 3	1.09	0.00023
13877.00	ILMN_212781	Mus musculus enhancer of rudimentary homolog (Drosophila) (Erh), mRNA.	1.09	1.40E-05
68087.00	ILMN_218673	Mus musculus dephospho-CoA kinase domain containing (Dcakd), mRNA.	1.09	1.90E-06
27407.00	Abcf2	ATP-binding cassette, sub-family F (GCN20), member 2	1.09	0.00017
74111.00	Rbm19	RNA binding motif protein 19	1.08	0.00083
240334.00	Pcyox1l	prenylcysteine oxidase 1 like	1.08	5.00E-04
66164.00	Nip7	nuclear import 7 homolog (S. cerevisiae)	1.08	2.30E-05
13849.00	Ephx1	epoxide hydrolase 1, microsomal	1.08	0.0055
13401.00	Dmwd	dystrophia myotonica-containing WD repeat motif	1.08	1.40E-05
27407.00	Abcf2	ATP-binding cassette, sub-family F (GCN20), member 2	1.08	0.00018
72155.00	Cenpn	centromere protein N	1.08	1.10E-05
66976.00	ILMN_212730	Mus musculus RIKEN cDNA 2410002F23 gene (2410002F23Rik), mRNA.	1.08	0.00025
68971.00	1500001M20Rik	RIKEN cDNA 1500001M20 gene	1.08	0.00014
22146.00	Tuba1c	tubulin, alpha 1C	1.07	0.00034
22021.00	Tpst1	protein-tyrosine sulfotransferase 1	1.07	7.90E-05
52033.00	Pbk	PDZ binding kinase	1.07	6.10E-05

26425.00	Nubp1	nucleotide binding protein 1	1.07	9.30E-06
17219.00	Mcm6	minichromosome maintenance deficient 6 (MIS5 homolog, S. pombe) (S. cerevisiae)	1.07	2.30E-06
100047856.00	ILMN_208813	PREDICTED: Mus musculus similar to calponin 3, acidic (LOC100047856), mRNA.	1.07	0.00036
15510.00	ILMN_213620	Mus musculus heat shock protein 1 (chaperonin) (Hspd1), mRNA.	1.07	0.016
14859.00	Gsta3	glutathione S-transferase, alpha 3	1.07	0.0029
20624.00	Eftud2	elongation factor Tu GTP binding domain containing 2	1.07	7.60E-06
50496.00	ILMN_220573	Mus musculus E2F transcription factor 6 (E2f6), mRNA.	1.07	2.50E-05
12387.00	ILMN_211511	Mus musculus catenin (cadherin associated protein), beta 1 (Ctnnb1), mRNA.	1.07	0.0018
107373.00	Fam111a	family with sequence similarity 111, member A	1.07	4.50E-06
27366.00	Txnl4a	thioredoxin-like 4A	1.06	2.80E-05
30056.00	Timm9	translocase of inner mitochondrial membrane 9 homolog (yeast)	1.06	7.10E-06
66628.00	Thg1l	tRNA-histidine guanylyltransferase 1-like (S. cerevisiae)	1.06	0.00015
11545.00	Parp1	poly (ADP-ribose) polymerase family, member 1	1.06	9.60E-06
56488.00	Nxt1	NTF2-related export protein 1	1.06	1.10E-05
234865.00	Nup133	nucleoporin 133	1.06	0.002
52683.00	Ncaph2	non-SMC condensin II complex, subunit H2	1.06	6.50E-06
16206.00	Lrig1	leucine-rich repeats and immunoglobulin-like domains 1	1.06	4.20E-06
76113.00	Lpo	lactoperoxidase	1.06	1.10E-05
16906.00	ILMN_220293	Mus musculus lamin B1 (Lmnb1), mRNA.	1.06	4.00E-05
80876.00	Ifitm2	interferon induced transmembrane protein 2	1.06	0.00076
66583.00	Exosc1	exosome component 1	1.06	4.60E-05
67102.00	D16Ertd472e	DNA segment, Chr 16, ERATO Doi 472, expressed	1.06	0.0011
21771.00	Cirh1a	cirrhosis, autosomal recessive 1A (human)	1.06	0.00013
67849.00	Cdca5	cell division cycle associated 5	1.06	0.00037
12462.00	Cct3	chaperonin containing Tcp1, subunit 3 (gamma)	1.06	8.30E-05
27081.00	Zfp275	zinc finger protein 275	1.05	0.00027
71354.00	Wdr31	WD repeat domain 31	1.05	4.00E-04
54141.00	Spag5	sperm associated antigen 5	1.05	6.50E-06
107686.00	Snrpd2	small nuclear ribonucleoprotein D2	1.05	3.50E-05
20587.00	Smarcb1	SWI/SNF related, matrix associated, actin dependent regulator of chromatin, subfamily b, member 1	1.05	2.00E-04
55963.00	Slc1a4	solute carrier family 1 (glutamate/neutral amino acid transporter), member 4	1.05	0.0015
28000.00	Prpf19	PRP19/PSO4 pre-mRNA processing factor 19 homolog (S. cerevisiae)	1.05	0.0012
28000.00	Prpf19	PRP19/PSO4 pre-mRNA processing factor 19 homolog (S. cerevisiae)	1.05	0.00015
67037.00	Pmf1	polyamine-modulated factor 1	1.05	4.20E-05
110109.00	Nop2	NOP2 nucleolar protein homolog (yeast)	1.05	9.40E-05
17975.00	Ncl	nucleolin	1.05	6.00E-05
108156.00	Mthfd1	methylenetetrahydrofolate dehydrogenase (NADP+ dependent), methenyltetrahydrofolate cyclohydrolase, formyltetrahydrofolate synthase	1.05	0.00037
17535.00	Mre11a	meiotic recombination 11 homolog A (S. cerevisiae)	1.05	1.80E-05
15191.00	Hdgf	hepatoma-derived growth factor	1.05	4.00E-05
232680.00	Cpa2	carboxypeptidase A2, pancreatic	1.05	6.00E-05
71963.00	Cdca4	cell division cycle associated 4	1.05	1.10E-05
71963.00	Cdca4	cell division cycle associated 4	1.05	5.80E-06
12443.00	Ccnd1	cyclin D1	1.05	0.00023
71735.00	Lrwd1	leucine-rich repeats and WD repeat domain containing 1	1.05	1.10E-05
15547.00	Trmt2a	TRM2 tRNA methyltransferase 2 homolog A (S. cerevisiae)	1.04	6.20E-05
21681.00	Thoc4	THO complex 4	1.04	2.30E-05
20425.00	Shmt1	serine hydroxymethyltransferase 1 (soluble)	1.04	0.00085
19361.00	Rad51	RAD51 homolog (S. cerevisiae)	1.04	0.0061
18637.00	Pfdn2	prefoldin 2	1.04	1.60E-05
52683.00	Ncaph2	non-SMC condensin II complex, subunit H2	1.04	1.30E-05
66902.00	Mtap	methylthioadenosine phosphorylase	1.04	3.00E-04
224092.00	Lsg1	large subunit GTPase 1 homolog (S. cerevisiae)	1.04	8.90E-06
192170.00	Eif4a3	eukaryotic translation initiation factor 4A3	1.04	0.00039
74747.00	Ddit4	DNA-damage-inducible transcript 4	1.04	0.0011
12236.00	Bub1b	budding uninhibited by benzimidazoles 1 homolog, beta (S. cerevisiae)	1.04	0.00036
27078.00	B9d1	B9 protein domain 1	1.04	5.10E-05
17025.00	ILMN_215056	Mus musculus aminolevulinate, delta-, dehydratase (Alad), mRNA.	1.04	7.50E-05
217737.00	Ahsa1	AHA1, activator of heat shock protein ATPase homolog 1 (yeast)	1.04	6.80E-06
66976.00	ILMN_212730	Mus musculus RIKEN cDNA 2410002F23 gene (2410002F23Rik), mRNA.	1.04	0.00045
68964.00	1500010J02Rik	RIKEN cDNA 1500010J02 gene	1.04	1.10E-06
57315.00	Wdr46	WD repeat domain 46	1.03	0.00013
21926.00	Tnf	tumor necrosis factor	1.03	0.0064
30056.00	Timm9	translocase of inner mitochondrial membrane 9 homolog (yeast)	1.03	1.60E-05
116914.00	Slc19a2	solute carrier family 19 (thiamine transporter), member 2	1.03	0.0034
214791.00	Sertad4	SERTA domain containing 4	1.03	1.00E-04
68275.00	Rpa1	replication protein A1	1.03	3.00E-06
19227.00	Pthlh	parathyroid hormone-like peptide	1.03	6.60E-06
93737.00	Pard6g	par-6 partitioning defective 6 homolog gamma (C. elegans)	1.03	2.10E-05

100609.00	Nsun5	NOL1/NOP2/Sun domain family, member 5	1.03	2.90E-05
56150.00	Mad2l1	MAD2 mitotic arrest deficient-like 1 (yeast)	1.03	1.70E-06
72500.00	ILMN_225633	Mus musculus immediate early response 5-like (Ier5l), mRNA.	1.03	9.30E-06
319170.00	Hist1h2an	histone cluster 1, H2an	1.03	0.012
276770.00	Eif5a	eukaryotic translation initiation factor 5A	1.03	4.00E-05
230917.00	Tmem201	transmembrane protein 201	1.02	2.80E-05
66525.00	Timm50	translocase of inner mitochondrial membrane 50 homolog (yeast)	1.02	2.00E-04
56403.00	Syncrip	synaptotagmin binding, cytoplasmic RNA interacting protein	1.02	4.40E-05
66506.00	Psmg3	proteasome (prosome, macropain) assembly chaperone 3	1.02	1.30E-05
20019.00	Polr1a	polymerase (RNA) I polypeptide A	1.02	4.90E-05
69912.00	Nup43	nucleoporin 43	1.02	4.50E-06
216443.00	Mars	methionine-tRNA synthetase	1.02	0.00092
319169.00	Hist1h2ak	histone cluster 1, H2ak	1.02	0.0081
107435.00	Hat1	histone aminotransferase 1	1.02	2.60E-05
14297.00	Fxn	frataxin	1.02	2.80E-05
20624.00	Eftud2	elongation factor Tu GTP binding domain containing 2	1.02	9.60E-06
228889.00	Ddx27	DEAD (Asp-Glu-Ala-Asp) box polypeptide 27	1.02	2.80E-05
108912.00	Cdca2	cell division cycle associated 2	1.02	7.90E-05
76813.00	ILMN_230074	Mus musculus armadillo repeat containing 6 (Armc6), mRNA.	1.02	0.00014
11564.00	Adsl	adenylosuccinate lyase	1.02	0.00012
72061.00	2010111I01Rik	RIKEN cDNA 2010111I01 gene	1.02	1.20E-05
21877.00	Tk1	thymidine kinase 1	1.01	0.0012
20621.00	Snn	stannin	1.01	0.0026
435684.00	ILMN_245068	Mus musculus Src homology 2 domain containing F (Shf), mRNA.	1.01	0.0033
16912.00	Psmb9	proteasome (prosome, macropain) subunit, beta type 9 (large multifunctional peptidase 2)	1.01	0.04
445007.00	Nup85	nucleoporin 85	1.01	0.0057
217011.00	ILMN_239279	Mus musculus notchless homolog 1 (Drosophila) (Nle1), mRNA.	1.01	0.0017
66973.00	ILMN_252778	Mus musculus mitochondrial ribosomal protein S18B (Mrps18b), nuclear gene encoding mitochondrial protein, mRNA.	1.01	0.00035
14793.00	Cdca3	cell division cycle associated 3	1.01	0.00055
215193.00	Diexf	digestive organ expansion factor homolog (zebrafish)	1.01	5.50E-05
319278.00	A230050P20Rik	RIKEN cDNA A230050P20 gene	1.01	0.0013
66356.00	2310008H09Rik	RIKEN cDNA 2310008H09 gene	1.01	6.60E-06
22793.00	Zyx	zyxin	1	0.0011
232187.00	Smyd5	SET and MYND domain containing 5	1	0.00095
19355.00	Rad1	RAD1 homolog (S. pombe)	1	1.00E-04
214572.00	Prmt7	protein arginine N-methyltransferase 7	1	1.90E-05
68106.00	Nt5c3l	5'-nucleotidase, cytosolic III-like	1	0.00013
16319.00	Incenp	inner centromere protein	1	0.00032
55927.00	Hes6	hairy and enhancer of split 6 (Drosophila)	1	0.00015
29870.00	Gtse1	G two S phase expressed protein 1	1	0.002
67112.00	Fgf22	fibroblast growth factor 22	1	0.00015
69524.00	Esam	endothelial cell-specific adhesion molecule	1	0.00018
27979.00	Eif3b	eukaryotic translation initiation factor 3, subunit B	1	0.00026
381903.00	Alg8	asparagine-linked glycosylation 8 homolog (yeast, alpha-1,3-glucosyltransferase)	1	0.00079
223921.00	Aaas	achalasia, adrenocortical insufficiency, alacrimia	1	0.00036
277414.00	Trp53i11	transformation related protein 53 inducible protein 11	-1	0.00048
229731.00	Slc25a24	solute carrier family 25 (mitochondrial carrier, phosphate carrier), member 24	-1	0.00054
67874.00	Rprm	reprimo, TP53 dependent G2 arrest mediator candidate	-1	0.00025
100047353.00	ILMN_212740	PREDICTED: Mus musculus similar to myocardial vascular inhibition factor (LOC100047353), mRNA.	-1	0.00029
100047173.00	ILMN_214714	PREDICTED: Mus musculus similar to synaptotagmin-like 1 (LOC100047173), misc RNA.	-1	1.60E-05
16196.00	Il7	interleukin 7	-1	0.002
93842.00	Igsf9	immunoglobulin superfamily, member 9	-1	0.0012
15484.00	Hsd11b2	hydroxysteroid 11-beta dehydrogenase 2	-1	0.013
14674.00	Gna13	guanine nucleotide binding protein, alpha 13	-1	0.0047
14367.00	Fzd5	frizzled homolog 5 (Drosophila)	-1	0.00015
105387.00	Akr1c14	aldo-keto reductase family 1, member C14	-1	5.40E-05
11522.00	Adh1	alcohol dehydrogenase 1 (class I)	-1	0.016
53330.00	Vamp4	vesicle-associated membrane protein 4	-1.01	2.90E-05
223697.00	Sun2	Sad1 and UNC84 domain containing 2	-1.01	0.0037
20482.00	Skil	SKI-like	-1.01	0.0063
224860.00	Plcl2	phospholipase C-like 2	-1.01	1.90E-05
224938.00	Pja2	praja 2, RING-H2 motif containing	-1.01	0.00017
102103.00	Mtus1	mitochondrial tumor suppressor 1	-1.01	0.00014
64095.00	Gpr35	G protein-coupled receptor 35	-1.01	0.003
71946.00	Endod1	endonuclease domain containing 1	-1.01	1.90E-05
171168.00	Acer1	alkaline ceramidase 1	-1.01	0.00046
208117.00	Aph1b	anterior pharynx defective 1b homolog (C. elegans)	-1.01	0.0053
66395.00	Ahnak	AHNAK nucleoprotein (desmoyokin)	-1.01	0.00061

18400.00	Slc22a18	solute carrier family 22 (organic cation transporter), member 18	-1.02	1.10E-05
24057.00	Sh3yl1	Sh3 domain YSC-like 1	-1.02	1.50E-05
71601.00	ILMN_213888	Mus musculus CEA-related cell adhesion molecule 20 (Ceacam20), mRNA.	-1.02	0.0049
11364.00	Acadm	acyl-Coenzyme A dehydrogenase, medium chain	-1.02	1.30E-06
98267.00	Stk17b	serine/threonine kinase 17b (apoptosis-inducing)	-1.03	0.0011
216233.00	Socs2	suppressor of cytokine signaling 2	-1.03	0.0015
69693.00	Pof1b	premature ovarian failure 1B	-1.03	3.80E-05
217166.00	Nr1d1	nuclear receptor subfamily 1, group D, member 1	-1.03	0.0019
83965.00	Enpp5	ectonucleotide pyrophosphatase/phosphodiesterase 5	-1.03	3.80E-05
170752.00	Bco2	beta-carotene oxygenase 2	-1.03	0.0047
73910.00	Arhgap18	Rho GTPase activating protein 18	-1.03	0.0011
11735.00	Ank3	ankyrin 3, epithelial	-1.03	0.0033
104776.00	Aldh6a1	aldehyde dehydrogenase family 6, subfamily A1	-1.03	0.0018
239559.00	A4galt	alpha 1,4-galactosyltransferase	-1.03	2.40E-07
66753.00	Erlec1	endoplasmic reticulum lectin 1	-1.03	3.00E-05
67171.00	Dram2	VDNA-damage regulated autophagy modulator 2	-1.04	0.00019
56374.00	Tmem59	transmembrane protein 59	-1.04	0.00014
233724.00	Tmem41b	transmembrane protein 41B	-1.04	0.0011
20356.00	Sema5a	sema domain, seven thrombospondin repeats (type 1 and type 1-like), transmembrane domain (TM) and short cytoplasmic domain, (semaphorin) 5A	-1.04	0.0015
65970.00	Lima1	LIM domain and actin binding 1	-1.04	0.0053
66809.00	Krt20	keratin 20	-1.04	0.007
319190.00	ILMN_213471	Mus musculus histone cluster 2, H2be (Hist2h2be), mRNA.	-1.04	8.00E-04
74155.00	Errfi1	ERBB receptor feedback inhibitor 1	-1.04	0.00035
66273.00	1810020D17Rik	RIKEN cDNA 1810020D17 gene	-1.04	0.00023
100727.00	Ugt2b34	UDP glucuronosyltransferase 2 family, polypeptide B34	-1.05	0.0087
67043.00	Syap1	synapse associated protein 1	-1.05	0.00011
217463.00	Snx13	sorting nexin 13	-1.05	0.0016
19885.00	Rorc	RAR-related orphan receptor gamma	-1.05	0.018
245867.00	Pcmtd2	protein-L-isoaspartate (D-aspartate) O-methyltransferase domain containing 2	-1.05	0.00071
26424.00	Nr5a2	nuclear receptor subfamily 5, group A, member 2	-1.05	0.00036
223646.00	Naprt1	nicotinate phosphoribosyltransferase domain containing 1	-1.05	0.0062
100046056.00	ILMN_192265	PREDICTED: Mus musculus similar to Pre-B-cell leukemia transcription factor interacting protein 1 (LOC100046056), mRNA.	-1.05	0.005
75686.00	Nudt16	nudix (nucleoside diphosphate linked moiety X)-type motif 16	-1.05	0.00011
319186.00	Hist1h2bm	histone cluster 1, H2bm	-1.05	0.00016
13360.00	Dhcr7	7-dehydrocholesterol reductase	-1.05	0.0017
13063.00	Cycs	cytochrome c, somatic	-1.05	9.00E-05
66813.00	ILMN_244180	Mus musculus Bcl2-like 14 (apoptosis facilitator) (Bcl2l14), mRNA.	-1.05	0.0016
223631.00	ILMN_209571	Mus musculus cDNA sequence BC025446 (BC025446), mRNA.	-1.05	0.0056
17940.00	Naip1	NLR family, apoptosis inhibitory protein 1	-1.06	0.0013
100047937.00	ILMN_219033	PREDICTED: Mus musculus similar to Aldehyde dehydrogenase 1 family, member L1 (LOC100047937), mRNA.	-1.06	4.60E-05
13105.00	Cyp2d9	cytochrome P450, family 2, subfamily d, polypeptide 9	-1.06	0.0092
104086.00	Cyp27a1	cytochrome P450, family 27, subfamily a, polypeptide 1	-1.06	0.0034
83429.00	Ctns	cystinosis, nephropathic	-1.06	0.00056
71908.00	Cldn23	claudin 23	-1.06	4.20E-05
12724.00	Clcn2	chloride channel 2	-1.06	1.00E-05
107747.00	ILMN_219033	Mus musculus aldehyde dehydrogenase 1 family, member L1 (Aldh1l1), mRNA.	-1.06	0.00011
66753.00	Erlec1	endoplasmic reticulum lectin 1	-1.06	9.60E-06
94224.00	Srd5a2	steroid 5 alpha-reductase 2	-1.07	1.40E-05
329416.00	Nostrin	nitric oxide synthase trafficker	-1.07	0.00092
17961.00	Nat2	N-acetyltransferase 2 (arylamine N-acetyltransferase)	-1.07	0.00029
99663.00	Clca6	chloride channel calcium activated 6	-1.07	0.025
26365.00	Ceacam1	carcinoembryonic antigen-related cell adhesion molecule 1	-1.07	0.02
353170.00	ILMN_247686	Mus musculus RIKEN cDNA 4932441K18 gene (4932441K18Rik), mRNA.	-1.07	4.20E-05
22248.00	Unc119	unc-119 homolog (C. elegans)	-1.08	8.90E-06
54683.00	Prdx5	peroxiredoxin 5	-1.08	0.00049
67801.00	Pllp	plasma membrane proteolipid	-1.08	0.0035
226971.00	Plekhb2	pleckstrin homology domain containing, family B (evectins) member 2	-1.08	2.30E-05
54484.00	Mkrn1	makorin, ring finger protein, 1	-1.08	0.00095
100046781.00	ILMN_220554	PREDICTED: Mus musculus similar to carboxypeptidase D (LOC100046781), mRNA.	-1.08	0.014
85308.00	Fam158a	family with sequence similarity 158, member A	-1.08	0.0011
13511.00	Dsg2	desmoglein 2	-1.08	0.00029
68778.00	1110038D17Rik	RIKEN cDNA 1110038D17 gene	-1.08	6.30E-07
225997.00	Trpm6	transient receptor potential cation channel, subfamily M, member 6	-1.09	0.00014
72948.00	Tppp	tubulin polymerization promoting protein	-1.09	3.50E-05
19241.00	ILMN_196070	Mus musculus thymosin, beta 4, X chromosome (Tmsb4x), mRNA.	-1.09	1.70E-05
20866.00	ILMN_221367	Mus musculus stromal interaction molecule 1 (Stim1), mRNA.	-1.09	2.50E-06
102693.00	Phldb1	pleckstrin homology-like domain, family B, member 1	-1.09	0.0019
18627.00	Per2	period homolog 2 (Drosophila)	-1.09	0.0027

54405.00	Ndufa1	NADH dehydrogenase (ubiquinone) 1 alpha subcomplex, 1	-1.09	0.00016
105559.00	Mbnl2	muscleblind-like 2	-1.09	4.40E-05
100048721.00	ILMN_210479	PREDICTED: Mus musculus similar to fibronectin leucine rich transmembrane protein 3, transcript variant 1 (LOC100048721), mRNA.	-1.09	0.00074
57890.00	Il17re	interleukin 17 receptor E	-1.09	0.0014
212070.00	Clrn3	clarin 3	-1.09	4.00E-04
76960.00	Bcas1	breast carcinoma amplified sequence 1	-1.09	2.80E-05
21934.00	Tnfrsf11a	tumor necrosis factor receptor superfamily, member 11a	-1.1	1.70E-05
21416.00	Tcf7l2	transcription factor 7-like 2, T-cell specific, HMG-box	-1.1	1.50E-05
54381.00	Pgcp	plasma glutamate carboxypeptidase	-1.1	0.0011
170761.00	Pdzd3	PDZ domain containing 3	-1.1	0.0026
18003.00	Nedd9	neural precursor cell expressed, developmentally down-regulated gene 9	-1.1	0.00059
26931.00	Ppp2r5c	protein phosphatase 2, regulatory subunit B (B56), gamma isoform	-1.1	0.00012
83379.00	Klb	klotho beta	-1.1	2.60E-06
68024.00	Hist1h2bc	histone cluster 1, H2bc	-1.1	0.00092
23882.00	Gadd45g	growth arrest and DNA-damage-inducible 45 gamma	-1.1	0.0017
11732.00	Ank	progressive ankylosis	-1.1	0.00037
107652.00	Uap1	UDP-N-acetylglucosamine pyrophosphorylase 1	-1.11	0.002
11891.00	Rab27a	RAB27A, member RAS oncogene family	-1.11	6.60E-06
213522.00	Plekhg6	pleckstrin homology domain containing, family G (with RhoGef domain) member 6	-1.11	0.00082
53880.00	ILMN_230036	Mus musculus NLR family, apoptosis inhibitory protein 7 (Naip7), mRNA.	-1.11	0.00011
16664.00	Krt14	keratin 14	-1.11	0.013
102871.00	D330045A20Rik	RIKEN cDNA D330045A20 gene	-1.11	0.00035
75415.00	Arhgap12	Rho GTPase activating protein 12	-1.11	5.10E-05
71874.00	2310007B03Rik	RIKEN cDNA 2310007B03 gene	-1.11	2.00E-04
20394.00	Scg5	secretogranin V	-1.12	6.10E-05
11852.00	Rhob	ras homolog gene family, member B	-1.12	0.00047
18858.00	Pmp22	peripheral myelin protein 22	-1.12	0.012
71801.00	Plekhf2	pleckstrin homology domain containing, family F (with FYVE domain) member 2	-1.12	0.0097
13139.00	Dgka	diacylglycerol kinase, alpha	-1.12	8.70E-05
23971.00	Papss1	3'-phosphoadenosine 5'-phosphosulfate synthase 1	-1.13	1.70E-06
107589.00	Mylk	myosin, light polypeptide kinase	-1.13	0.0029
100044862.00	ILMN_221289	PREDICTED: Mus musculus similar to Fbxl3 protein (LOC100044862), mRNA.	-1.13	0.00035
16478.00	Jund	Jun proto-oncogene related gene d	-1.13	0.0026
57890.00	Il17re	interleukin 17 receptor E	-1.13	3.80E-05
66822.00	Fbxo25	F-box protein 25	-1.13	3.00E-04
13139.00	Dgka	diacylglycerol kinase, alpha	-1.13	2.30E-05
67095.00	Trak1	trafficking protein, kinesin binding 1	-1.14	1.10E-05
22134.00	Tgoln1	trans-golgi network protein	-1.14	3.10E-05
50776.00	Polg2	polymerase (DNA directed), gamma 2, accessory subunit	-1.14	0.00019
223646.00	Naprt1	nicotinate phosphoribosyltransferase domain containing 1	-1.14	0.00012
14063.00	F2rl1	coagulation factor II (thrombin) receptor-like 1	-1.14	0.0016
212483.00	Fam193b	family with sequence similarity 193, member B	-1.14	0.00019
56643.00	Slc15a1	solute carrier family 15 (oligopeptide transporter), member 1	-1.15	0.02
66824.00	ILMN_223398	Mus musculus PYD and CARD domain containing (Pycard), mRNA.	-1.15	0.00014
59030.00	Mkks	McKusick-Kaufman syndrome protein	-1.15	1.80E-06
16168.00	Il15	interleukin 15	-1.15	2.30E-05
384009.00	Glipr2	GLI pathogenesis-related 2	-1.15	0.0033
380711.00	Rap1gap2	RAP1 GTPase activating protein 2	-1.15	3.00E-06
74754.00	Dhcr24	24-dehydrocholesterol reductase	-1.15	0.0017
66298.00	Defa21	defensin, alpha, 21	-1.15	0.019
12457.00	ILMN_208982	Mus musculus CCR4 carbon catabolite repression 4-like (S. cerevisiae) (Ccrn4l), mRNA.	-1.15	0.0029
66264.00	Ccdc28b	coiled coil domain containing 28B	-1.15	0.00041
76527.00	Il34	interleukin 34	-1.15	7.90E-07
109637.00	Upk1a	uroplakin 1A	-1.16	7.30E-05
209760.00	Tmc7	transmembrane channel-like gene family 7	-1.16	0.00082
20755.00	ILMN_215269	Mus musculus small proline-rich protein 2A (Sprr2a), mRNA.	-1.16	5.30E-06
72002.00	Slc39a5	solute carrier family 39 (metal ion transporter), member 5	-1.16	4.00E-05
108079.00	Prkaa2	protein kinase, AMP-activated, alpha 2 catalytic subunit	-1.16	6.20E-05
23954.00	Nek3	NIMA (never in mitosis gene a)-related expressed kinase 3	-1.16	1.30E-05
228576.00	Mall	mal, T-cell differentiation protein-like	-1.16	0.0011
20216.00	Acsm3	acyl-CoA synthetase medium-chain family member 3	-1.16	2.60E-05
53376.00	Usp2	ubiquitin specific peptidase 2	-1.17	0.00018
14605.00	Tsc22d3	TSC22 domain family, member 3	-1.17	0.00019
64177.00	Trpv6	transient receptor potential cation channel, subfamily V, member 6	-1.17	0.0016
170756.00	ILMN_214023	Mus musculus solute carrier family 24 (sodium/potassium/calcium exchanger), member 6 (Slc24a6), mRNA.	-1.17	0.00078
76108.00	Rap2a	RAS related protein 2a	-1.17	0.00029
76787.00	Ppfia3	protein tyrosine phosphatase, receptor type, f polypeptide (PTPRF), interacting protein (liprin), alpha 3	-1.17	0.00095
17190.00	Mbd1	methyl-CpG binding domain protein 1	-1.17	1.60E-05

212307.00	Mapre2	microtubule-associated protein, RP/EB family, member 2	-1.17	1.60E-05
56486.00	Gabarap	gamma-aminobutyric acid receptor associated protein	-1.17	4.90E-06
245038.00	Dclk3	doublecortin-like kinase 3	-1.17	4.60E-05
12653.00	Chgb	chromogranin B	-1.17	2.30E-05
110595.00	Timp4	tissue inhibitor of metalloproteinase 4	-1.18	3.50E-05
72002.00	Slc39a5	solute carrier family 39 (metal ion transporter), member 5	-1.18	0.002
67709.00	Reg4	regenerating islet-derived family, member 4	-1.18	0.002
71664.00	Mettl7b	methyltransferase like 7B	-1.18	0.002
16529.00	Kcnk5	potassium channel, subfamily K, member 5	-1.18	6.20E-05
77996.00	D730039F16Rik	RIKEN cDNA D730039F16 gene	-1.18	5.00E-04
269336.00	Ccdc32	coiled-coil domain containing 32	-1.18	5.60E-07
20730.00	Spink3	serine peptidase inhibitor, Kazal type 3	-1.19	0.0053
18799.00	Plcd1	phospholipase C, delta 1	-1.19	0.00014
16601.00	Klf9	Kruppel-like factor 9	-1.19	0.0037
16598.00	Klf2	Kruppel-like factor 2 (lung)	-1.19	0.0076
69718.00	Ipmk	inositol polyphosphate multikinase	-1.19	4.90E-06
380921.00	Dgkh	diacylglycerol kinase, eta	-1.19	3.60E-07
12684.00	Cideb	cell death-inducing DNA fragmentation factor, alpha subunit-like effector B	-1.19	0.00016
67064.00	Chmp1b	chromatin modifying protein 1B	-1.19	8.30E-05
381175.00	Ccdc68	coiled-coil domain containing 68	-1.19	0.0029
12226.00	ILMN_212740	Mus musculus B-cell translocation gene 1, anti-proliferative (Btg1), mRNA.	-1.19	1.00E-05
72361.00	ILMN_219691	Mus musculus RIKEN cDNA 2210023G05 gene (2210023G05Rik), mRNA.	-1.19	6.70E-05
21807.00	Tsc22d1	TSC22 domain family, member 1	-1.2	0.00034
18753.00	Prkcd	protein kinase C, delta	-1.2	4.40E-07
228983.00	Osbpl2	oxysterol binding protein-like 2	-1.2	9.20E-08
668837.00	ILMN_207496	PREDICTED: Mus musculus similar to ATP synthase, H+ transporting, mitochondrial F0 complex, subunit G (LOC668837), misc RNA.	-1.2	0.00047
15212.00	Hexb	hexosaminidase B	-1.2	0.00041
223706.00	Cyp2d34	cytochrome P450, family 2, subfamily d, polypeptide 34	-1.2	0.0013
12452.00	Ccng2	cyclin G2	-1.2	0.0033
223631.00	ILMN_209571	Mus musculus cDNA sequence BC025446 (BC025446), mRNA.	-1.2	0.00046
19123.00	Proc	protein C	-1.21	0.00017
260409.00	Cdc42ep3	CDC42 effector protein (Rho GTPase binding) 3	-1.21	0.0025
69787.00	Anxa13	annexin A13	-1.21	0.0049
27360.00	Add3	adducin 3 (gamma)	-1.21	4.20E-05
107723.00	Slc12a6	solute carrier family 12, member 6	-1.22	7.60E-06
223646.00	Naprt1	nicotinate phosphoribosyltransferase domain containing 1	-1.22	0.00011
239217.00	Kctd12	potassium channel tetramerisation domain containing 12	-1.22	1.90E-05
16168.00	Il15	interleukin 15	-1.22	0.00022
53897.00	Gal3st1	galactose-3-O-sulfotransferase 1	-1.22	0.00015
78252.00	Fam55b	family with sequence similarity 55, member B	-1.22	0.0047
20443.00	St3gal4	ST3 beta-galactoside alpha-2,3-sialyltransferase 4	-1.23	0.0012
18574.00	Pde1b	phosphodiesterase 1B, Ca2+-calmodulin dependent	-1.23	0.00012
105559.00	Mbnl2	muscleblind-like 2	-1.23	1.80E-05
12409.00	Cbr2	carbonyl reductase 2	-1.23	0.00047
12351.00	Car4	carbonic anhydrase 4	-1.23	0.0012
230163.00	Aldob	aldolase B, fructose-bisphosphate	-1.23	0.003
238330.00	ILMN_230817	Mus musculus RIKEN cDNA 6430527G18 gene (6430527G18Rik), mRNA.	-1.23	0.00018
67198.00	Spats2l	spermatogenesis associated, serine-rich 2-like	-1.23	0.00068
52357.00	Wwc2	WW, C2 and coiled-coil domain containing 2	-1.24	0.00049
11853.00	Rhoc	ras homolog gene family, member C	-1.24	0.00011
109731.00	Maob	monoamine oxidase B	-1.24	0.002
20238.00	Atxn1	ataxin 1	-1.24	5.80E-05
70113.00	Odf3b	outer dense fiber of sperm tails 3B	-1.24	4.70E-06
16581.00	Kifc2	kinesin family member C2	-1.25	1.40E-05
382571.00	ILMN_188365	Mus musculus potassium voltage-gated channel, subfamily F, member 1 (Kcnf1), mRNA.	-1.25	0.00073
93692.00	Glrx	glutaredoxin	-1.25	1.30E-05
13819.00	Epas1	endothelial PAS domain protein 1	-1.25	8.00E-06
13040.00	Ctss	cathepsin S	-1.25	0.0017
53422.00	ILMN_196190	Mus musculus Y box protein 2 (Ybx2), mRNA.	-1.26	9.60E-05
76273.00	Ndfip2	Nedd4 family interacting protein 2	-1.26	3.10E-05
212307.00	Mapre2	microtubule-associated protein, RP/EB family, member 2	-1.26	8.80E-07
16529.00	Kcnk5	potassium channel, subfamily K, member 5	-1.26	1.10E-05
14915.00	Guca2a	guanylate cyclase activator 2a (guanylin)	-1.26	3.80E-06
14281.00	Fos	FBJ osteosarcoma oncogene	-1.26	0.00018
68636.00	Fahd1	fumarylacetoacetate hydrolase domain containing 1	-1.26	6.10E-05
212862.00	ILMN_218521	Mus musculus choline phosphotransferase 1 (Chpt1), mRNA.	-1.26	4.20E-05
20276.00	Scnn1a	sodium channel, nonvoltage-gated 1 alpha	-1.27	8.50E-06
11758.00	ILMN_210596	Mus musculus peroxiredoxin 6 (Prdx6), mRNA.	-1.27	9.30E-06

23954.00	Nek3	NIMA (never in mitosis gene a)-related expressed kinase 3	-1.27	1.50E-06
16432.00	Itm2b	integral membrane protein 2B	-1.27	0.00054
27360.00	Add3	adducin 3 (gamma)	-1.27	6.10E-05
67038.00	2010109I03Rik	RIKEN cDNA 2010109I03 gene	-1.27	0.032
70113.00	Odf3b	outer dense fiber of sperm tails 3B	-1.27	0.00011
69864.00	1810065E05Rik	RIKEN cDNA 1810065E05 gene	-1.27	0.0056
232078.00	Thnsl2	threonine synthase-like 2 (bacterial)	-1.28	6.50E-07
21415.00	Tcf7l1	transcription factor 7-like 1 (T-cell specific, HMG box)	-1.28	0.0027
14102.00	Fas	Fas (TNF receptor superfamily member 6)	-1.28	0.00014
241041.00	Gm4956	predicted gene 4956	-1.28	1.10E-05
70031.00	Cmtm8	CKLF-like MARVEL transmembrane domain containing 8	-1.28	4.50E-06
381122.00	Capn13	calpain 13	-1.28	0.0045
12945.00	Dmbt1	deleted in malignant brain tumors 1	-1.29	0.0051
75568.00	Capsl	calcyphosine-like	-1.29	1.50E-06
74340.00	Ahcyl2	S-adenosylhomocysteine hydrolase-like 2	-1.29	0.00011
17965.00	Nbl1	neuroblastoma, suppression of tumorigenicity 1	-1.3	3.50E-05
14132.00	Fcgrt	Fc receptor, IgG, alpha chain transporter	-1.3	1.00E-04
13821.00	Epb4.1l1	erythrocyte protein band 4.1-like 1	-1.3	4.90E-05
20515.00	Slc20a1	solute carrier family 20, member 1	-1.31	1.00E-04
18703.00	Pigr	polymeric immunoglobulin receptor	-1.31	0.00073
554292.00	ILMN_238123	Mus musculus UbiE-YGHL1 fusion protein (LOC554292), mRNA.	-1.31	1.60E-05
209378.00	Itih5	inter-alpha (globulin) inhibitor H5	-1.31	1.20E-05
66270.00	Fam134b	family with sequence similarity 134, member B	-1.31	0.00018
73102.00	Slc22a23	solute carrier family 22, member 23	-1.31	4.30E-07
23971.00	Papss1	3'-phosphoadenosine 5'-phosphosulfate synthase 1	-1.32	1.50E-06
99031.00	Osbpl6	oxysterol binding protein-like 6	-1.32	2.70E-06
384061.00	ILMN_244553	Mus musculus fibronectin type III domain containing 5 (Fndc5), mRNA.	-1.32	1.60E-06
109901.00	Cela1	chymotrypsin-like elastase family, member 1	-1.32	0.0022
12864.00	Cox6c	cytochrome c oxidase, subunit VIc	-1.32	0.0055
76959.00	Chmp5	chromatin modifying protein 5	-1.32	6.50E-06
67171.00	ILMN_257311	Mus musculus transmembrane protein 77 (Tmem77), transcript variant 2, mRNA.	-1.33	4.70E-06
108682.00	Gpt2	glutamic pyruvate transaminase (alanine aminotransferase) 2	-1.33	0.00044
338521.00	Fa2h	fatty acid 2-hydroxylase	-1.33	5.00E-04
626708.00	Defa26	defensin, alpha, 26	-1.33	0.00045
71900.00	Tmem106b	transmembrane protein 106B	-1.33	5.50E-05
66938.00	1700029G01Rik	RIKEN cDNA 1700029G01 gene	-1.33	4.60E-05
19775.00	ILMN_195778	Mus musculus xenotropic and polytropic retrovirus receptor 1 (Xpr1), mRNA.	-1.34	8.00E-06
17289.00	Mertk	c-mer proto-oncogene tyrosine kinase	-1.34	0.00081
12457.00	ILMN_208982	Mus musculus CCR4 carbon catabolite repression 4-like (S. cerevisiae) (Ccrn4l), mRNA.	-1.34	0.00036
52466.00	ILMN_213036	Mus musculus solute carrier family 46, member 1 (Slc46a1), mRNA.	-1.35	0.0015
94249.00	Slc24a3	solute carrier family 24 (sodium/potassium/calcium exchanger), member 3	-1.35	1.50E-05
18569.00	Pdcd4	programmed cell death 4	-1.35	0.00011
26384.00	ILMN_226523	Mus musculus glucosamine-6-phosphate deaminase 1 (Gnpda1), mRNA.	-1.35	8.30E-05
67731.00	Fbxo32	F-box protein 32	-1.35	4.40E-05
13240.00	Defa6	defensin, alpha, 6	-1.35	0.0042
244853.00	Fam55d	family with sequence similarity 55, member D	-1.35	0.0056
227929.00	Cytip	cytohesin 1 interacting protein	-1.35	8.90E-06
232078.00	Thnsl2	threonine synthase-like 2 (bacterial)	-1.36	1.50E-06
15567.00	Slc6a4	solute carrier family 6 (neurotransmitter transporter, serotonin), member 4	-1.36	2.70E-05
18175.00	Nrap	nebulin-related anchoring protein	-1.36	7.90E-05
77996.00	D730039F16Rik	RIKEN cDNA D730039F16 gene	-1.36	1.80E-05
13101.00	Cyp2d10	cytochrome P450, family 2, subfamily d, polypeptide 10	-1.36	0.00045
13479.00	Dpep1	dipeptidase 1 (renal)	-1.37	0.0035
12700.00	Cish	cytokine inducible SH2-containing protein	-1.37	1.80E-05
212862.00	ILMN_218521	Mus musculus choline phosphotransferase 1 (Chpt1), mRNA.	-1.37	8.70E-05
58210.00	Sectm1b	secreted and transmembrane 1B	-1.38	0.0041
20196.00	S100a13	S100 calcium binding protein A13	-1.38	6.00E-05
243725.00	Ppp1r9a	protein phosphatase 1, regulatory (inhibitor) subunit 9A	-1.38	4.50E-06
68009.00	ILMN_196346	Mus musculus defensin related cryptdin 20 (Defcr20), mRNA.	-1.38	0.00065
66610.00	Abi3	ABI gene family, member 3	-1.38	0.00047
381204.00	Naaladl1	N-acetylated alpha-linked acidic dipeptidase-like 1	-1.39	0.0076
109218.00	Tmem139	transmembrane protein 139	-1.4	4.00E-05
50708.00	Hist1h1c	histone cluster 1, H1c	-1.4	5.50E-05
108112.00	ILMN_218862	Mus musculus eukaryotic translation initiation factor 4E binding protein 3 (Eif4ebp3), mRNA.	-1.4	9.40E-05
56318.00	ILMN_218213	Mus musculus acid phosphatase, prostate (Acpp), transcript variant 1, mRNA.	-1.4	1.70E-06
76507.00	Abp1	amiloride binding protein 1 (amine oxidase, copper-containing)	-1.4	6.10E-05
234797.00	6430548M08Rik	RIKEN cDNA 6430548M08 gene	-1.4	0.00084
20208.00	Saa1	serum amyloid A 1	-1.41	0.0069

76051.00	ILMN_221992	Mus musculus glucosidase, alpha; neutral C (Ganc), mRNA.	-1.41	0.00022
11676.00	Aldoc	aldolase C, fructose-bisphosphate	-1.41	9.90E-07
207819.00	ILMN_212940	Mus musculus RIKEN cDNA 4930539E08 gene (4930539E08Rik), mRNA.	-1.41	0.00013
66222.00	Serpinb1a	serine (or cysteine) peptidase inhibitor, clade B, member 1a	-1.42	4.20E-05
26395.00	Map2k1	mitogen-activated protein kinase kinase 1	-1.42	0.00086
70261.00	2010110P09Rik	RIKEN cDNA 2010110P09 gene	-1.42	0.00049
102857.00	Slc6a8	solute carrier family 6 (neurotransmitter transporter, creatine), member 8	-1.43	0.00048
71207.00	ILMN_212961	Mus musculus nudix (nucleoside diphosphate linked moiety X)-type motif 4 (Nudt4), mRNA.	-1.43	0.00018
13216.00	ILMN_196581	Mus musculus defensin, alpha 1 (Defa1), mRNA.	-1.43	0.0012
240916.00	Vsig8	V-set and immunoglobulin domain containing 8	-1.44	1.40E-05
69123.00	ILMN_210310	Mus musculus RIKEN cDNA 1810022C23 gene (1810022C23Rik), mRNA.	-1.45	4.40E-06
15430.00	Hoxd10	homeobox D10	-1.46	0.0076
71436.00	ILMN_210479	Mus musculus fibronectin leucine rich transmembrane protein 3 (Flrt3), mRNA.	-1.47	6.50E-06
67198.00	Spats2l	spermatogenesis associated, serine-rich 2-like	-1.48	9.60E-05
80890.00	ILMN_187599	Mus musculus tripartite motif protein 2 (Trim2), mRNA. XM_984114 XM_984144 XM_984172 XM_984200 XM_984238 XM_984275 XM_984313	-1.49	0.00022
245049.00	Myrip	myosin VIIA and Rab interacting protein	-1.49	0.00029
67731.00	Fbxo32	F-box protein 32	-1.49	5.80E-05
229933.00	Clca5	chloride channel calcium activated 5	-1.5	8.00E-06
76722.00	Ckmt2	creatine kinase, mitochondrial 2	-1.5	1.80E-06
16600.00	Klf4	Kruppel-like factor 4 (gut)	-1.51	2.80E-05
545156.00	Kalrn	kalirin, RhoGEF kinase	-1.51	0.00019
13239.00	ILMN_196558	Mus musculus defensin related cryptdin 5 (Defcr5), mRNA.	-1.52	0.0036
101772.00	Ano1	anoctamin 1, calcium activated chloride channel	-1.52	9.30E-06
53906.00	Phgr1	proline/histidine/glycine-rich 1	-1.52	3.40E-06
108079.00	Prkaa2	protein kinase, AMP-activated, alpha 2 catalytic subunit	-1.53	0.0011
212862.00	ILMN_218521	Mus musculus choline phosphotransferase 1 (Chpt1), mRNA.	-1.53	1.80E-05
234673.00	Ces2e	carboxylesterase 2E	-1.53	0.0011
67731.00	Fbxo32	F-box protein 32	-1.54	3.00E-04
13821.00	Epb4.1l1	erythrocyte protein band 4.1-like 1	-1.54	1.80E-06
19017.00	Ppargc1a	peroxisome proliferative activated receptor, gamma, coactivator 1 alpha	-1.55	1.00E-04
13218.00	Defa-rs1	defensin, alpha, related sequence 1	-1.55	0.0076
56448.00	ILMN_223233	Mus musculus cytochrome P450, family 2, subfamily d, polypeptide 22 (Cyp2d22), mRNA.	-1.55	0.0032
69574.00	Cmbl	carboxymethylenebutenolidase-like (Pseudomonas)	-1.55	5.50E-05
433470.00	AA467197	expressed sequence AA467197	-1.55	0.00059
227327.00	B3gnt7	UDP-GlcNAc:betaGal beta-1,3-N-acetylglucosaminyltransferase 7	-1.56	0.0025
11829.00	Aqp4	aquaporin 4	-1.56	0.00019
239606.00	Slc2a13	solute carrier family 2 (facilitated glucose transporter), member 13	-1.57	2.10E-05
102871.00	D330045A20Rik	RIKEN cDNA D330045A20 gene	-1.58	7.50E-05
231946.00	D330028D13Rik	RIKEN cDNA D330028D13 gene	-1.58	0.00029
23844.00	Clca3	chloride channel calcium activated 3	-1.58	0.0081
319848.00	Slc17a4	solute carrier family 17 (sodium phosphate), member 4	-1.59	4.60E-05
14199.00	Fhl1	four and a half LIM domains 1	-1.59	0.0087
20510.00	Slc1a1	solute carrier family 1 (neuronal/epithelial high affinity glutamate transporter, system Xag), member 1	-1.6	3.70E-06
170677.00	Cdhr1	cadherin-related family member 1	-1.6	1.70E-06
68009.00	ILMN_196346	Mus musculus defensin related cryptdin 20 (Defcr20), mRNA.	-1.6	0.00056
384061.00	Fndc5	fibronectin type III domain containing 5	-1.61	3.50E-07
230576.00	Ttc22	tetratricopeptide repeat domain 22	-1.62	5.80E-06
20541.00	Slc8a1	solute carrier family 8 (sodium/calcium exchanger), member 1	-1.63	0.00094
207259.00	Zbtb7c	zinc finger and BTB domain containing 7C	-1.64	1.50E-05
13240.00	Defa6	defensin, alpha, 6	-1.64	0.0011
16600.00	Klf4	Kruppel-like factor 4 (gut)	-1.65	4.00E-05
433904.00	Ociad2	OCIA domain containing 2	-1.66	4.40E-05
56209.00	Gde1	glycerophosphodiester phosphodiesterase 1	-1.66	8.90E-06
14199.00	Fhl1	four and a half LIM domains 1	-1.67	0.003
226781.00	Slc30a10	solute carrier family 30, member 10	-1.68	0.0044
76722.00	Ckmt2	creatine kinase, mitochondrial 2	-1.68	1.10E-06
258458.00	Olfr165	olfactory receptor 165	-1.7	6.00E-06
16600.00	Klf4	Kruppel-like factor 4 (gut)	-1.7	9.20E-05
56226.00	Espn	espin	-1.7	2.20E-05
20342.00	ILMN_227312	Mus musculus selenium binding protein 2 (Selenbp2), mRNA.	-1.71	0.00044
67182.00	Pdzk1ip1	PDZK1 interacting protein 1	-1.71	3.80E-05
100044204.00	ILMN_221943	PREDICTED: Mus musculus hypothetical protein LOC100044204 (LOC100044204), mRNA.	-1.71	0.00011
56209.00	Gde1	glycerophosphodiester phosphodiesterase 1	-1.71	1.70E-06
76787.00	Ppfia3	protein tyrosine phosphatase, receptor type, f polypeptide (PTPRF), interacting protein (liprin), alpha 3	-1.74	3.80E-05
56209.00	Gde1	glycerophosphodiester phosphodiesterase 1	-1.74	4.70E-06
99663.00	Clca6	chloride channel calcium activated 6	-1.74	0.03
192970.00	Dhrs11	dehydrogenase/reductase (SDR family) member 11	-1.74	5.70E-06
18858.00	Pmp22	peripheral myelin protein 22	-1.77	0.00094

74134.00	Cyp2s1	cytochrome P450, family 2, subfamily s, polypeptide 1	-1.77	5.00E-06
20510.00	Slc1a1	solute carrier family 1 (neuronal/epithelial high affinity glutamate transporter, system Xag), member 1	-1.78	6.70E-07
20910.00	Stxbp1	syntaxin binding protein 1	-1.79	6.50E-06
66438.00	Hamp2	hepcidin antimicrobial peptide 2	-1.79	0.00013
71687.00	Tmem25	transmembrane protein 25	-1.8	2.50E-06
20755.00	ILMN_215269	Mus musculus small proline-rich protein 2A (Sprr2a), mRNA.	-1.8	7.60E-06
66090.00	Ypel3	yippee-like 3 (Drosophila)	-1.85	2.00E-06
68416.00	Sycn	syncollin	-1.85	0.016
100041194.00	Ahnak2	AHNAK nucleoprotein 2	-1.85	2.90E-07
268860.00	Abat	4-aminobutyrate aminotransferase	-1.85	4.40E-07
15446.00	Hpgd	hydroxyprostaglandin dehydrogenase 15 (NAD)	-1.86	2.60E-05
12865.00	Cox7a1	cytochrome c oxidase, subunit VIIa 1	-1.87	4.50E-06
67307.00	Pbld2	phenazine biosynthesis-like protein domain containing 2	-1.87	3.70E-07
13034.00	ILMN_253611	Mus musculus cathepsin E (Ctse), mRNA.	-1.89	0.00017
20259.00	Scin	scinderin	-1.9	4.90E-05
16429.00	Itln1	intelectin 1 (galactofuranose binding)	-1.9	2.00E-04
12231.00	Btn1a1	butyrophilin, subfamily 1, member A1	-1.91	4.80E-07
230163.00	Aldob	aldolase B, fructose-bisphosphate	-1.91	0.00055
77889.00	Lbh	limb-bud and heart	-1.93	4.90E-05
20500.00	Slc13a2	solute carrier family 13 (sodium-dependent dicarboxylate transporter), member 2	-1.95	0.00062
26456.00	Sema4g	sema domain, immunoglobulin domain (Ig), transmembrane domain (TM) and short cytoplasmic domain, (semaphorin) 4G	-1.96	4.20E-06
268860.00	Abat	4-aminobutyrate aminotransferase	-1.96	1.60E-05
72948.00	Tppp	tubulin polymerization promoting protein	-1.98	6.50E-06
56226.00	Espn	espin	-1.98	2.30E-05
20363.00	Sepp1	selenoprotein P, plasma, 1	-2.02	0.0034
100044291.00	ILMN_221210	PREDICTED: Mus musculus hypothetical protein LOC100044291 (LOC100044291), mRNA.	-2.02	9.40E-05
20531.00	Slc34a2	solute carrier family 34 (sodium phosphate), member 2	-2.05	0.0019
16173.00	Il18	interleukin 18	-2.05	6.70E-05
23959.00	Nt5e	5' nucleotidase, ecto	-2.06	0.0012
77889.00	Lbh	limb-bud and heart	-2.08	0.00014
268480.00	Rapgefl1	Rap guanine nucleotide exchange factor (GEF)-like 1	-2.1	3.10E-07
23959.00	Nt5e	5' nucleotidase, ecto	-2.11	2.10E-05
13370.00	Dio1	deiodinase, iodothyronine, type I	-2.11	3.10E-06
57319.00	Smpdl3a	sphingomyelin phosphodiesterase, acid-like 3A	-2.12	8.50E-06
493583.00	ILMN_256959	Mus musculus intelectin b (Itlnb), mRNA.	-2.2	4.00E-05
13370.00	Dio1	deiodinase, iodothyronine, type I	-2.23	1.70E-06
12579.00	Cdkn2b	cyclin-dependent kinase inhibitor 2B (p15, inhibits CDK4)	-2.26	1.70E-06
20363.00	Sepp1	selenoprotein P, plasma, 1	-2.3	0.00079
268480.00	Rapgefl1	Rap guanine nucleotide exchange factor (GEF)-like 1	-2.33	6.10E-06
109791.00	Clps	colipase, pancreatic	-2.37	2.50E-05
17287.00	Mep1a	meprin 1 alpha	-2.41	0.00036
101488.00	Slco2b1	solute carrier organic anion transporter family, member 2b1	-2.52	7.30E-05
109791.00	Clps	colipase, pancreatic	-2.57	4.60E-05
21818.00	Tgm3	transglutaminase 3, E polypeptide	-2.58	4.20E-05
69083.00	Sult1c2	sulfotransferase family, cytosolic, 1C, member 2	-2.58	9.90E-08
67971.00	Tppp3	tubulin polymerization-promoting protein family member 3	-2.59	4.80E-07
53315.00	Sult1d1	sulfotransferase family 1D, member 1	-2.63	1.60E-06
56185.00	Hao2	hydroxyacid oxidase 2	-2.67	7.10E-06
20887.00	Sult1a1	sulfotransferase family 1A, phenol-preferring, member 1	-2.7	2.00E-05
393082.00	ILMN_243966	Mus musculus methyltransferase like 7A2 (Mettl7a2), mRNA.	-2.73	1.30E-09
545288.00	Cyp2c67	cytochrome P450, family 2, subfamily c, polypeptide 67	-2.77	4.40E-07
13615.00	Edn2	endothelin 2	-2.98	2.80E-05
22635.00	Zan	zonadhesin	-2.99	3.60E-08
233038.00	Nccrp1	non-specific cytotoxic cell receptor protein 1 homolog (zebrafish)	-3.11	2.10E-06
216225.00	Slc5a8	solute carrier family 5 (iodide transporter), member 8	-3.13	1.60E-06
219033.00	Ang4	angiogenin, ribonuclease A family, member 4	-3.25	6.70E-05
13107.00	Cyp2f2	cytochrome P450, family 2, subfamily f, polypeptide 2	-3.49	3.60E-08
18947.00	Pnliprp2	pancreatic lipase-related protein 2	-3.52	1.10E-05
232889.00	Pla2g4c	phospholipase A2, group IVC (cytosolic, calcium-independent)	-3.72	3.00E-04
331063.00	ILMN_196357	Mus musculus expressed sequence AI987692 (AI987692), mRNA.	-4.02	4.50E-06
18947.00	Pnliprp2	pancreatic lipase-related protein 2	-4.18	2.40E-06
270328.00	ILMN_196360	Mus musculus gasdermin C3 (Gsdmc3), mRNA.	-4.29	1.90E-07
331063.00	Gsdmc2	gasdermin C2	-4.4	3.50E-07
100045250.00	ILMN_196357	PREDICTED: Mus musculus hypothetical protein LOC100045250 (LOC100045250), misc RNA.	-4.5	3.70E-06

Supplementary Table S3

Ct values of genes analyzed by qRT-PCR in HEK293 cells treated with DMSO (control) or BIO; obtained Ct values were normalized to β-actin gene expression; SD, standard deviation.

	DMSO		BIO	
gene name	Ct values	SD	Ct values	SD
MSX1	28.47	0.15	27.96	0.08
MSX2	25.74	0.04	25.09	0.11
NKD1	26.23	0.18	22.42	0.15
TROY	26.21	0.05	24.43	0.11
UBB	18.62	0.06	18.74	0.01

Supplementary Table S3

Ct values of genes analyzed by qRT-PCR in STF cells with the intact (control) or truncated *APC* gene in exon 10 or exon 15; obtained Ct values were normalized to β-actin gene expression; SD, standard deviation.

	control		exon 10		exon 15	
gene name	Ct values	SD	Ct values	SD	Ct values	SD
AXIN2	31.17	0.40	29.54	0.01	28.75	0.01
MSX1	28.28	0.43	27.04	0.02	25.58	0.11
MSX2	27.20	0.16	25.78	0.01	24.14	0.03
NKD1	28.36	0.06	24.14	0.13	25.22	0.07
UBB	19.40	0.04	18.97	0.01	19.80	0.02

Supplementary Table S3

Ct values of genes analyzed by qRT-PCR in SW480 cells upon transfection with non-silencing (nsc) siRNAs or siRNAs targeting *β-catenin* mRNA; obtained Ct values were normalized to *β-actin* gene expression; SD, standard deviation.

	nsc siRNAs		β-catenin siRNAs	
gene name	Ct values	SD	Ct values	SD
β-CATENIN	21.17	0.08	26.48	0.30
MSX1	26.10	0.01	28.54	0.42
MSX2	25.22	0.24	27.65	0.17
NKD1	22.93	0.12	27.39	0.16
TROY	26.72	0.00	29.23	0.08
UBB	18.86	0.16	19.56	0.04

Supplementary Table S3

Ct values of genes analyzed by qRT-PCR in SW620 cells upon transfection with non-silencing (nsc) siRNAs or siRNAs targeting *β-catenin* mRNA; obtained Ct values were normalized to *β-actin* gene expression; SD, standard deviation.

	nsc siRNAs		β-catenin siRNAs	
gene name	Ct values	SD	Ct values	SD
β-CATENIN	20.59	1.15	23.66	0.10
MSX1	20.64	0.04	24.39	0.06
MSX2	24.28	0.06	25.99	0.11
NKD1	20.90	0.16	25.12	0.01
TROY	27.24	0.25	29.14	0.42
UBB	17.47	0.39	17.81	0.25

Supplementary Table S3

Ct values of genes analyzed by qRT-PCR in STF cells with the *APC* gene truncated in exon 10 upon transfection with non-silencing (nsc) siRNAs or siRNAs targeting *β-catenin* mRNA; obtained Ct values were normalized to *β-actin* gene expression; SD, standard deviation.

	nsc siRNAs		β-catenin siRNAs	
gene name	Ct values	SD	Ct values	SD
AXIN2	23.68	0.02	25.10	0.02
β-CATENIN	22.48	0.08	25.75	0.11
MSX1	28.99	0.14	29.83	0.10
MSX2	26.31	0.09	27.15	0.09
UBB	19.56	0.05	19.38	0.03

Supplementary Table S4

Differentialy expressed genes (with |logFC|≥ 1; p ≤ 0.05) in the small intestinal Msx1 wt and Msx1-deficient hyperplastic epithelium

ENTREZ	SYMBOL	GENENAME	logFC	p-value
170942	Erdr1	erythroid differentiation regulator 1	3.52	2.00E-04
57742	Abhd1	abhydrolase domain containing 1	2.93	4.10E-05
57742	Abhd1	abhydrolase domain containing 1	2.84	5.60E-05
434794		Mus musculus X-linked lymphocyte-regulated 4A (Xlr4a), mRNA	2.81	0.0083
11746	Anxa4	annexin A4	2.77	0.066
57742	Abhd1	abhydrolase domain containing 1	2.26	0.00068
68337	Crip2	cysteine rich protein 2	2.19	6.90E-05
223227	Sox21	SRY (sex determining region Y)-box 21	2.1	0.0098
20249	Scd1	stearoyl-Coenzyme A desaturase 1	2.03	0.001
15122		Mus musculus hemoglobin alpha, adult chain 1 (Hba-a1), mRNA.	1.92	0.19
19652	Rbm3	RNA binding motif protein 3	1.88	0.0014
14733	Gpc1	glypican 1	1.88	0.018
406217	Bex4	brain expressed X-linked 4	1.79	0.014
14472	Gbx2	gastrulation brain homeobox 2	1.78	0.019
223227	Sox21	SRY (sex determining region Y)-box 21	1.77	0.014
20350	Sema3f	sema domain, immunoglobulin domain (Ig), short basic domain, secreted, (semaphorin) 3F	1.67	0.014
192212	Prom2	prominin 2	1.65	0.048
69195	Tmem121	transmembrane protein 121	1.64	0.14
64293	Stk32b	serine/threonine kinase 32B	1.64	0.00092
23962	Oasl2	2'-5' oligoadenylate synthetase-like 2	1.57	0.0045
16145	Igtp	interferon gamma induced GTPase	1.57	0.1
329064	Pkd2l1	polycystic kidney disease 2-like 1	1.52	0.0026
15360	Hmgcs2	3-hydroxy-3-methylglutaryl-Coenzyme A synthase 2	1.52	0.006
14469	Gbp2	guanylate binding protein 2	1.5	0.22
21380	Tbx1	T-box 1	1.49	0.0081
20350	Sema3f	sema domain, immunoglobulin domain (Ig), short basic domain, secreted, (semaphorin) 3F	1.42	0.013
100038882	Isg15	ISG15 ubiquitin-like modifier	1.41	0.067
17329	Cxcl9	chemokine (C-X-C motif) ligand 9	1.41	0.12
68713	Ifitm1	interferon induced transmembrane protein 1	1.34	0.11
76943	Psapl1	prosaposin-like 1	1.32	0.0097
13644	Efs	embryonal Fyn-associated substrate	1.32	0.02
72434	Lypd3	Ly6/Plaur domain containing 3	1.31	0.011
67855	Asprv1	aspartic peptidase, retroviral-like 1	1.31	0.047
22269	Upk2	uroplakin 2	1.3	0.0084
24110	Usp18	ubiquitin specific peptidase 18	1.29	0.018
52250	Reep1	receptor accessory protein 1	1.29	0.015
11749	Anxa6	annexin A6	1.28	0.049
226245	Plekhs1	pleckstrin homology domain containing, family S member 1	1.28	0.028
20671	Sox17	SRY (sex determining region Y)-box 17	1.27	0.05
108052	Slc14a1	solute carrier family 14 (urea transporter), member 1	1.27	0.002
11567	Avil	advillin	1.27	0.14
67967	Pold3	polymerase (DNA-directed), delta 3, accessory subunit	1.26	0.0033
58185	Rsad2	radical S-adenosyl methionine domain containing 2	1.25	0.024
17242	Mdk	midkine	1.25	4.10E-05
20129	Rptn	repetin	1.24	0.0021
20671	Sox17	SRY (sex determining region Y)-box 17	1.23	0.0072
67951	Tubb6	tubulin, beta 6 class V	1.21	0.04
20910	Stxbp1	syntaxin binding protein 1	1.21	0.0026
268396	Sh3pxd2b	SH3 and PX domains 2B	1.2	0.0029
13034	Ctse	cathepsin E	1.2	0.11
22153	Tubb4a	tubulin, beta 4A class IVA	1.19	0.053
15959	Ifit3	interferon-induced protein with tetratricopeptide repeats 3	1.19	0.041
231050	Galnt11	UDP-N-acetyl-alpha-D-galactosamine:polypeptide N-acetylgalactosaminyltransferase 11	1.19	0.036
69065	Chac1	ChaC, cation transport regulator 1	1.18	0.045
108052	Slc14a1	solute carrier family 14 (urea transporter), member 1	1.17	0.012
654824	Ankrd37	ankyrin repeat domain 37	1.17	0.0047
225027	Srsf7	serine/arginine-rich splicing factor 7	1.16	0.039
240334	Pcyox1l	prenylcysteine oxidase 1 like	1.16	0.001
246728	Oas2	2'-5' oligoadenylate synthetase 2	1.15	0.12
27261	Dok3	docking protein 3	1.15	0.0033
74007	Btbd11	BTB (POZ) domain containing 11	1.15	0.042
11752	Anxa8	annexin A8	1.12	0.1
22228	Ucp2	uncoupling protein 2 (mitochondrial, proton carrier)	1.1	9.60E-05
226123	Morn4	MORN repeat containing 4	1.1	0.0011
406217		Mus musculus brain expressed gene 4 (Bex4), mRNA.	1.09	0.027
244886	Tmem266	transmembrane protein 266	1.09	0.0042
240675	Vwa2	von Willebrand factor A domain containing 2	1.08	0.0048
58194	Sh3kbp1	SH3-domain kinase binding protein 1	1.08	0.058
331063	Gsdmc2	gasdermin C2	1.08	0.0099
18812	Prl2c3	prolactin family 2, subfamily c, member 3	1.07	0.0057

27395	Mrpl15	mitochondrial ribosomal protein L15	1.07	0.0046
24110	Usp18	ubiquitin specific peptidase 18	1.07	0.0093
15958	Ifit2	interferon-induced protein with tetratricopeptide repeats 2	1.07	0.02
54396	Irgm2	immunity-related GTPase family M member 2	1.06	0.17
15505	Hsph1	heat shock 105kDa/110kDa protein 1	1.06	0.003
24110	Usp18	ubiquitin specific peptidase 18	1.05	0.023
331063	Gsdmc2	gasdermin C2	1.05	0.021
54352	Irx5	Iroquois related homeobox 5 (Drosophila)	1.03	0.011
11639	Ak4	adenylate kinase 4	1.02	0.061
74777	Sepn1	selenoprotein N, 1	1.01	0.02
213171	Prss27	protease, serine 27	1.01	0.06
21939	Cd40	CD40 antigen	1.01	0.002
21858	Timp2	tissue inhibitor of metalloproteinase 2	1	0.016
22379	Fmnl3	formin-like 3	1	0.18
11684	Alox12	arachidonate 12-lipoxygenase	1	0.0048
381524	AI427809	expressed sequence AI427809	1	0.0057
67800	Dgat2	diacylglycerol O-acyltransferase 2	-1	0.0065
21685	Tef	thyrotroph embryonic factor	-1.01	0.012
217166	Nr1d1	nuclear receptor subfamily 1, group D, member 1	-1.01	0.034
56615	Mgst1	microsomal glutathione S-transferase 1	-1.01	0.021
216136	Ilvbl	ilvB (bacterial acetolactate synthase)-like	-1.01	0.0079
66412	Arrdc4	arrestin domain containing 4	-1.02	0.0092
74525	Fam234b	family with sequence similarity 234, member B	-1.02	0.0084
330962	Slc51b	solute carrier family 51, beta subunit	-1.03	0.17
14345	Fut4	fucosyltransferase 4	-1.03	0.0057
74519	Cyp2j9	cytochrome P450, family 2, subfamily j, polypeptide 9	-1.03	0.18
11459	Acta1	actin, alpha 1, skeletal muscle	-1.03	0.06
50934	Slc7a8	solute carrier family 7 (cationic amino acid transporter, y+ system), member 8	-1.04	0.022
71584	Gdpd2	glycerophosphodiester phosphodiesterase domain containing 2	-1.04	0.0091
226040	Tmem252	transmembrane protein 252	-1.04	0.00038
101488	Slco2b1	solute carrier organic anion transporter family, member 2b1	-1.05	0.015
74338	Slc6a19	solute carrier family 6 (neurotransmitter transporter), member 19	-1.05	0.11
18150		Mus musculus nucleoplasmin 3 (Npm3), mRNA.	-1.05	0.043
18096	Nkx6-1	NK6 homeobox 1	-1.05	0.04
13088	Cyp2b10	cytochrome P450, family 2, subfamily b, polypeptide 10	-1.05	0.11
100434	Slc44a1	solute carrier family 44, member 1	-1.06	0.0037
19341	Rab4a	RAB4A, member RAS oncogene family	-1.06	0.0069
18036	Nfkbib	nuclear factor of kappa light polypeptide gene enhancer in B cells inhibitor, beta	-1.06	0.004
433278	Khdc1c	KH domain containing 1C	-1.06	0.00052
14219	Ctgf	connective tissue growth factor	-1.06	0.00012
68396	Nat8	N-acetyltransferase 8 (GCN5-related)	-1.06	0.16
223631	BC025446	cDNA sequence BC025446	-1.06	0.056
66273	Aamdc	adipogenesis associated Mth938 domain containing	-1.06	0.0027
225997	Trpm6	transient receptor potential cation channel, subfamily M, member 6	-1.07	0.00025
242864	Napepld	N-acyl phosphatidylethanolamine phospholipase D	-1.07	0.038
68943	Pink1	PTEN induced putative kinase 1	-1.07	0.0039
15186	Hdc	histidine decarboxylase	-1.07	0.012
97998	Deptor	DEP domain containing MTOR-interacting protein	-1.07	0.018
11829	Aqp4	aquaporin 4	-1.07	0.0059
23849	Klf6	Kruppel-like factor 6	-1.08	0.00018
13120	Cyp4b1	cytochrome P450, family 4, subfamily b, polypeptide 1	-1.08	0.11
381903	Alg8	asparagine-linked glycosylation 8 (alpha-1,3-glucosyltransferase)	-1.08	0.017
230163	Aldob	aldolase B, fructose-bisphosphate	-1.08	0.052
11539	Adora1	adenosine A1 receptor	-1.08	0.0032
217166	Nr1d1	nuclear receptor subfamily 1, group D, member 1	-1.09	0.061
72303	Cyp2c65	cytochrome P450, family 2, subfamily c, polypeptide 65	-1.09	0.11
102294	Cyp4v3	cytochrome P450, family 4, subfamily v, polypeptide 3	-1.1	0.14
22064		Mus musculus transient receptor potential cation channel, subfamily C, member 2 (Trpc2), transcript variant 1, mRNA.	-1.11	0.019
208659	Fam20a	family with sequence similarity 20, member A	-1.11	0.0055
71972	Dnmbp	dynamin binding protein	-1.11	0.034
104681	Slc16a6	solute carrier family 16 (monocarboxylic acid transporters), member 6	-1.14	0.0023
13106	Cyp2e1	cytochrome P450, family 2, subfamily e, polypeptide 1	-1.14	0.22
19329	Rab17	RAB17, member RAS oncogene family	-1.15	0.0031
104009	Qsox1	quiescin Q6 sulfhydryl oxidase 1	-1.15	0.0048
17988	Ndrg1	N-myc downstream regulated gene 1	-1.15	0.061
18604	Pdk2	pyruvate dehydrogenase kinase, isoenzyme 2	-1.16	0.063
57257	Vav3	vav 3 oncogene	-1.17	0.017
104158	Ces1d	carboxylesterase 1D	-1.17	0.3
14164	Fgf1	fibroblast growth factor 1	-1.18	0.035
225913	Tkfc	triokinase, FMN cyclase	-1.19	0.0058
230163	Aldob	aldolase B, fructose-bisphosphate	-1.19	0.028
72514	Fgfbp3	fibroblast growth factor binding protein 3	-1.2	0.0013
67470	Abcg8	ATP-binding cassette, sub-family G (WHITE), member 8	-1.2	0.0086
20526	Slc2a2	solute carrier family 2 (facilitated glucose transporter), member 2	-1.21	0.037
68404	Nrn1	neuritin 1	-1.21	0.0021
109731	Maob	monoamine oxidase B	-1.21	0.065

71584	Gdpd2	glycerophosphodiester phosphodiesterase domain containing 2	-1.22	0.019
56643	Slc15a1	solute carrier family 15 (oligopeptide transporter), member 1	-1.23	0.076
15199	Hebp1	heme binding protein 1	-1.23	0.052
114664	Hsd17b11	hydroxysteroid (17-beta) dehydrogenase 11	-1.24	0.02
14456	Gas6	growth arrest specific 6	-1.24	0.014
11826	Aqp1	aquaporin 1	-1.24	0.036
117591	Slc2a9	solute carrier family 2 (facilitated glucose transporter), member 9	-1.25	0.0094
328059	Slc7a15	solute carrier family 7 (cationic amino acid transporter, y+ system), member 15	-1.26	0.12
72373	Psca	prostate stem cell antigen	-1.26	0.0058
15567	Slc6a4	solute carrier family 6 (neurotransmitter transporter, serotonin), member 4	-1.29	0.006
68180	Hyi	hydroxypyruvate isomerase (putative)	-1.29	0.0043
192653	Ttc36	tetratricopeptide repeat domain 36	-1.31	0.0019
76279	Cyp2d26	cytochrome P450, family 2, subfamily d, polypeptide 26	-1.31	0.18
67473	Slc47a1	solute carrier family 47, member 1	-1.31	0.17
15446	Hpgd	hydroxyprostaglandin dehydrogenase 15 (NAD)	-1.34	0.06
12696	Cirbp	cold inducible RNA binding protein	-1.34	0.0014
26458	Slc27a2	solute carrier family 27 (fatty acid transporter), member 2	-1.35	0.0039
104681	Slc16a6	solute carrier family 16 (monocarboxylic acid transporters), member 6	-1.37	0.0067
14164	Fgf1	fibroblast growth factor 1	-1.37	0.021
69983	Sis	sucrase isomaltase (alpha-glucosidase)	-1.37	0.0039
93695	Gpnmb	glycoprotein (transmembrane) nmb	-1.38	0.11
56350	Arl3	ADP-ribosylation factor-like 3	-1.39	4.00E-04
108099	Prkag2	protein kinase, AMP-activated, gamma 2 non-catalytic subunit	-1.4	0.0035
232889	Pla2g4c	phospholipase A2, group IVC (cytosolic, calcium-independent)	-1.4	0.22
209387	Trim30d	tripartite motif-containing 30D	-1.4	0.03
384783	Irs2	insulin receptor substrate 2	-1.43	0.0096
20526	Slc2a2	solute carrier family 2 (facilitated glucose transporter), member 2	-1.44	0.033
78748	Rassf10	Ras association (RalGDS/AF-6) domain family (N-terminal) member 10	-1.45	0.0065
27276	Plekhb1	pleckstrin homology domain containing, family B (evectins) member 1	-1.46	0.0011
11826	Aqp1	aquaporin 1	-1.46	0.021
235674	Acaa1b	acetyl-Coenzyme A acyltransferase 1B	-1.47	0.19
66116	Nat8f1	N-acetyltransferase 8 (GCN5-related) family member 1	-1.48	4.00E-04
69710	Arap1	ArfGAP with RhoGAP domain, ankyrin repeat and PH domain 1	-1.48	0.00034
20526	Slc2a2	solute carrier family 2 (facilitated glucose transporter), member 2	-1.54	0.067
66298	Defa21	defensin, alpha, 21	-1.56	0.0033
76279	Cyp2d26	cytochrome P450, family 2, subfamily d, polypeptide 26	-1.57	0.18
170442	Bbox1	butyrobetaine (gamma), 2-oxoglutarate dioxygenase 1 (gamma-butyrobetaine hydroxylase)	-1.58	0.026
76279	Cyp2d26	cytochrome P450, family 2, subfamily d, polypeptide 26	-1.59	0.14
20887	Sult1a1	sulfotransferase family 1A, phenol-preferring, member 1	-1.6	0.049
11826	Aqp1	aquaporin 1	-1.6	0.011
20019	Polr1a	polymerase (RNA) I polypeptide A	-1.61	0.00012
16204	Fabp6	fatty acid binding protein 6, ileal (gastrotropin)	-1.62	0.2
67432	Hoga1	4-hydroxy-2-oxoglutarate aldolase 1	-1.64	0.0047
381058	Unc93a	unc-93 homolog A (C. elegans)	-1.65	0.019
545902	Ptprh	protein tyrosine phosphatase, receptor type, H	-1.67	0.0083
20170	Hps6	Hermansky-Pudlak syndrome 6	-1.72	0.00016
233575	Pgap2	post-GPI attachment to proteins 2	-1.73	0.0023
53315	Sult1d1	sulfotransferase family 1D, member 1	-1.76	0.0043
11997	Akr1b7	aldo-keto reductase family 1, member B7	-1.78	0.026
14121	Fbp1	fructose bisphosphatase 1	-1.79	0.016
16548	Khk	ketohexokinase	-1.86	0.0085
16548	Khk	ketohexokinase	-1.9	0.0022
67432	Hoga1	4-hydroxy-2-oxoglutarate aldolase 1	-1.9	0.013
14377	G6pc	glucose-6-phosphatase, catalytic	-1.95	0.021
192236	Hps1	Hermansky-Pudlak syndrome 1	-2.05	0.00082
54150	Rdh7	retinol dehydrogenase 7	-2.08	0.033
233549	Mogat2	monoacylglycerol O-acyltransferase 2	-2.12	0.0023
69710	Arap1	ArfGAP with RhoGAP domain, ankyrin repeat and PH domain 1	-2.12	0.0015
11522	Adh1	alcohol dehydrogenase 1 (class I)	-2.13	0.012
56018	Stard10	START domain containing 10	-2.14	0.0017
12780	Abcc2	ATP-binding cassette, sub-family C (CFTR/MRP), member 2	-2.18	0.0059
54150	Rdh7	retinol dehydrogenase 7	-2.23	0.032
56388	Cyp3a25	cytochrome P450, family 3, subfamily a, polypeptide 25	-2.4	0.034
11522	Adh1	alcohol dehydrogenase 1 (class I)	-2.44	0.0073
17921	Myo7a	myosin VIIA	-2.54	0.0032
13112	Cyp3a11	cytochrome P450, family 3, subfamily a, polypeptide 11	-2.82	0.065
17701	Msx1	msh homeobox 1	-3.05	0.064
233549	Mogat2	monoacylglycerol O-acyltransferase 2	-3.14	0.0037
233571	P2ry6	pyrimidinergic receptor P2Y, G-protein coupled, 6	-3.31	0.00024
18479	Pak1	p21 protein (Cdc42/Rac)-activated kinase 1	-3.4	0.003
68185	Coa4	cytochrome c oxidase assembly factor 4	-3.44	8.00E-04
52443	Mrpl48	mitochondrial ribosomal protein L48	-3.56	0.0018
52443	Mrpl48	mitochondrial ribosomal protein L48	-3.77	0.0016
27050	Rps3	ribosomal protein S3	-6.14	0.0025

Supplementary Table S5

Ct values of genes analyzed in the small intestinal epithelium of the indicated mice 7 days after tamoxifen administration; the values were normalized to *β-actin* gene expression; SD, standard deviation.

	Control		*Apc* $^{KO/KO}$ *Msx1* $^{+/+}$		*Apc* $^{KO/KO}$ *Msx1* $^{KO/KO}$	
gene name	Ct values	SD	Ct values	SD	Ct values	SD
Ascl2	34.25	0.80	27.76	0.51	27.26	0.15
Axin2	30.52	0.74	25.49	0.58	25.27	0.11
Chromogranin A	28.48	0.49	28.21	0.33	28.97	0.35
GAPDH	21.43	0.31	20.84	0.59	20.31	0.14
Lgr5	34.10	0.62	28.98	0.49	28.60	0.03
Msx1	42.54	1.51	28.95	0.95	32.64	0.72
Msx2	45.11	0.69	34.54	0.16	34.31	1.04
SI	25.27	0.73	27.27	0.01	28.73	0.46
SP5	30.74	0.46	27.34	1.19	26.73	0.15

Supplementary Table S6

Differentialy expressed genes (|logFC| ≥ 0.8) in in the Apc/Msx1 double-deficient colonic mucosa when compared to the Apc-deficient tissue with intact Msx1

PROBE ID	ENSEMBL ID	SYMBOL	GENENAME	logFC	p-value
ENSMUST00000094836	ENSMUSG00000029123	Stk32b	serine/threonine kinase 32B	2.06e+00	3.70e-09
ENSMUST00000103399	ENSMUSG00000076598	Igkv3-7	immunoglobulin kappa variable 3-7	1.94e+00	8.93e-04
ENSMUST00000197560	ENSMUSG00000076598	Igkv3-7	immunoglobulin kappa variable 3-7	1.94e+00	8.93e-04
ENSMUST00000177591	ENSMUSG00000096768	Erdr1	erythroid differentiation regulator 1	1.48e+00	2.43e-02
ENSMUST00000177671	ENSMUSG00000096768	Erdr1	erythroid differentiation regulator 1	1.44e+00	2.60e-02
ENSMUST00000178789	ENSMUSG00000095562	Gm21887	predicted gene, 21887	1.37e+00	2.73e-02
ENSMUST00000179483	ENSMUSG00000096768	Erdr1	erythroid differentiation regulator 1	1.37e+00	2.73e-02
ENSMUST00000180251	ENSMUSG00000095562	Gm21887	predicted gene, 21887	1.37e+00	2.73e-02
ENSMUST00000044159	ENSMUSG00000060807	Serpina6	serine (or cysteine) peptidase inhibitor, clade A, member 6	1.36e+00	1.54e-04
ENSMUST00000179077	ENSMUSG00000096768	Erdr1	erythroid differentiation regulator 1	1.34e+00	2.64e-02
ENSMUST00000100692	ENSMUSG00000095528	Gm10375	predicted gene 10375	1.16e+00	3.12e-02
ENSMUST00000163970	ENSMUSG00000095528	Gm10375	predicted gene 10375	1.16e+00	3.12e-02
ENSMUST00000196706	ENSMUSG00000027869	Hsd3b6	hydroxy-delta-5-steroid dehydrogenase, 3 beta- and steroid delta-isomerase 6	1.15e+00	4.68e-04
ENSMUST00000211636	ENSMUSG00000040640	Erc2	ELKS/RAB6-interacting/CAST family member 2	1.15e+00	8.61e-04
ENSMUST00000144418	ENSMUSG00000028469	Npr2	natriuretic peptide receptor 2	1.15e+00	1.48e-02
ENSMUST00000172766	ENSMUSG00000050423	Ppp1r3g	protein phosphatase 1, regulatory (inhibitor) subunit 3G	1.13e+00	5.97e-03
ENSMUST00000113512	ENSMUSG00000073643	Wdfy1	WD repeat and FYVE domain containing 1	1.12e+00	3.83e-03
ENSMUST00000113513	ENSMUSG00000073643	Wdfy1	WD repeat and FYVE domain containing 1	1.12e+00	3.83e-03
ENSMUST00000113514	ENSMUSG00000073643	Wdfy1	WD repeat and FYVE domain containing 1	1.12e+00	3.83e-03
ENSMUST00000113515	ENSMUSG00000073643	Wdfy1	WD repeat and FYVE domain containing 1	1.12e+00	3.83e-03
ENSMUST00000187005	ENSMUSG00000073643	Wdfy1	WD repeat and FYVE domain containing 1	1.08e+00	3.73e-03
ENSMUST00000203150	ENSMUSG00000030361	Klrb1a	killer cell lectin-like receptor subfamily B member 1A	1.06e+00	4.21e-02
ENSMUST00000172486	ENSMUSG00000015222	Map2	microtubule-associated protein 2	1.05e+00	8.34e-03
ENSMUST00000135885	ENSMUSG00000029095	Ablim2	actin-binding LIM protein 2	1.04e+00	1.43e-04
ENSMUST00000186702	ENSMUSG00000041460	Cacna2d4	calcium channel, voltage-dependent, alpha 2/delta subunit 4	1.03e+00	5.87e-03
ENSMUST00000131920	ENSMUSG00000023267	Gabrr2	gamma-aminobutyric acid (GABA) C receptor, subunit rho 2	1.03e+00	3.48e-02
ENSMUST00000171262	ENSMUSG00000006711	D130043K22Rik	RIKEN cDNA D130043K22 gene	1.01e+00	1.44e-02
ENSMUST00000186394	ENSMUSG00000074109	Mrgprx2	MAS-related GPR, member X2	1.00e+00	8.34e-03
ENSMUST00000103483	ENSMUSG00000076674	Ighv3-8	immunoglobulin heavy variable V3-8	9.86e-01	3.05e-01
ENSMUST00000185329	ENSMUSG00000025932	Eya1	EYA transcriptional coactivator and phosphatase 1	9.84e-01	4.82e-02
ENSMUST00000040361	ENSMUSG00000039347	Atp6v0e2	ATPase, H+ transporting, lysosomal V0 subunit E2	9.79e-01	5.14e-03
ENSMUST00000136987	ENSMUSG00000043587	Pxylp1	2-phosphoxylose phosphatase 1	9.68e-01	9.28e-04
ENSMUST00000144697	ENSMUSG00000026999	Nup35	nucleoporin 35	9.67e-01	1.95e-02
ENSMUST00000153129	ENSMUSG00000028047	Thbs3	thrombospondin 3	9.65e-01	4.39e-02
ENSMUST00000103350	ENSMUSG00000076549	Igkv4-68	immunoglobulin kappa variable 4-68	9.63e-01	1.02e-02
ENSMUST00000137290	ENSMUSG00000031698	Mylk3	myosin light chain kinase 3	9.62e-01	1.37e-02
ENSMUST00000169797	ENSMUSG00000037849	Ifi206	interferon activated gene 206	9.49e-01	7.66e-02
ENSMUST00000155275	ENSMUSG00000021596	Mctp1	multiple C2 domains, transmembrane 1	9.39e-01	6.10e-03
ENSMUST00000162154	ENSMUSG00000022148	Fyb	FYN binding protein	9.37e-01	1.19e-02
ENSMUST00000161947	ENSMUSG00000022148	Fyb	FYN binding protein	9.29e-01	7.54e-03
ENSMUST00000190151	ENSMUSG00000021209	Ppp4r4	protein phosphatase 4, regulatory subunit 4	9.08e-01	7.42e-03
ENSMUST00000172478	ENSMUSG00000074369	Obox2	oocyte specific homeobox 2	8.92e-01	1.01e-02
ENSMUST00000174076	ENSMUSG00000074369	Obox2	oocyte specific homeobox 2	8.92e-01	1.01e-02
ENSMUST00000174305	ENSMUSG00000074369	Obox2	oocyte specific homeobox 2	8.92e-01	1.01e-02
ENSMUST00000149336	ENSMUSG00000029651	Mtus2	microtubule associated tumor suppressor candidate 2	8.84e-01	5.44e-03
ENSMUST00000194041	ENSMUSG00000026587	Astn1	astrotactin 1	8.62e-01	1.16e-04
ENSMUST00000213557	ENSMUSG00000071317	Bves	blood vessel epicardial substance	8.61e-01	1.04e-02
ENSMUST00000015576	ENSMUSG00000022226	Mcpt2	mast cell protease 2	8.59e-01	7.10e-04
ENSMUST00000207685	ENSMUSG00000035177	Nlrp2	NLR family, pyrin domain containing 2	8.50e-01	6.82e-04
ENSMUST00000204277	ENSMUSG00000039347	Atp6v0e2	ATPase, H+ transporting, lysosomal V0 subunit E2	8.50e-01	3.72e-03
ENSMUST00000201736	ENSMUSG00000094719	Gm5108	predicted gene 5108	8.48e-01	1.46e-03
ENSMUST00000135355	ENSMUSG00000021645	Smn1	survival motor neuron 1	8.46e-01	4.36e-02
ENSMUST00000142251	ENSMUSG00000051747	Ttn	titin	8.43e-01	8.03e-02
ENSMUST00000195849	ENSMUSG00000034837	Gnat1	guanine nucleotide binding protein, alpha transducing 1	8.41e-01	2.01e-02
ENSMUST00000202984	ENSMUSG00000006641	Slc5a6	solute carrier family 5 (sodium-dependent vitamin transporter), member 6	8.29e-01	1.50e-02
ENSMUST00000022836	ENSMUSG00000022227	Mcpt1	mast cell protease 1	8.28e-01	1.36e-02
ENSMUST00000176196	ENSMUSG00000032595	Cdhr4	cadherin-related family member 4	8.25e-01	1.70e-02
ENSMUST00000177093	ENSMUSG00000032595	Cdhr4	cadherin-related family member 4	8.25e-01	1.70e-02
ENSMUST00000141085	ENSMUSG00000041216	Clvs1	clavesin 1	8.23e-01	3.49e-02
ENSMUST00000095450	ENSMUSG00000071178	Serpina1b	serine (or cysteine) preptidase inhibitor, clade A, member 1B	8.20e-01	2.66e-02
ENSMUST00000164454	ENSMUSG00000071178	Serpina1b	serine (or cysteine) preptidase inhibitor, clade A, member 1B	8.20e-01	2.66e-02
ENSMUST00000186166	ENSMUSG00000071178	Serpina1b	serine (or cysteine) preptidase inhibitor, clade A, member 1B	8.20e-01	2.66e-02
ENSMUST00000195095	ENSMUSG00000104098	AA619741	expressed sequence AA619741	8.18e-01	3.66e-02
ENSMUST00000168044	ENSMUSG00000043557	Mdga1	MAM domain containing glycosylphosphatidylinositol anchor 1	8.15e-01	2.99e-04
ENSMUST00000189541	ENSMUSG00000016918	Sulf1	sulfatase 1	8.13e-01	1.79e-02
ENSMUST00000172308	ENSMUSG00000072731	Gm3715	predicted gene 3715	8.08e-01	3.60e-03
ENSMUST00000190082	ENSMUSG00000026246	Alppl2	alkaline phosphatase, placental-like 2	8.08e-01	1.82e-02
ENSMUST00000148715	ENSMUSG00000009246	Trpm5	transient receptor potential cation channel, subfamily M, member 5	8.06e-01	4.03e-02
ENSMUST00000191403	ENSMUSG00000099826	Scgb2b10	secretoglobin, family 2B, member 10	8.04e-01	1.00e-01
ENSMUST00000103323	ENSMUSG00000076522	Igkv16-104	immunoglobulin kappa variable 16-104	8.00e-01	1.14e-01

Supplementary Table S7

Ct values of genes analyzed in the proximal colonic epithelium of the indicated mice 7 days after tamoxifen administration; the values were normalized to β-actin gene expression; SD, standard deviation.

	Control		*Apc* $^{KO/KO}$ *Msx1* $^{+/+}$		*Apc* $^{KO/KO}$ *Msx1* $^{KO/KO}$	
gene name	Ct values	SD	Ct values	SD	Ct values	SD
Bves	37.99	3.99	40.51	1.40	40.44	2.59
Mdga1	31.68	1.22	33.49	0.55	32.96	0.91
Mtus2	32.68	1.15	35.65	0.84	35.19	0.23
Mylk3	37.07	1.51	39.09	0.69	38.71	1.37
Msx1	36.40	1.51	26.64	0.50	30.14	0.86
Msx2	36.94	1.06	27.23	0.62	26.55	0.33
Slc5a6	26.08	1.11	28.40	0.31	26.72	1.07
Stk32b	34.16	0.87	34.69	0.41	27.41	0.49
Trpm	39.73	3.53	44.12	3.19	42.30	2.30
Ttn	40.55	1.27	40.29	0.61	40.81	2.14
Ubb	18.27	0.94	19.63	0.34	19.30	0.29

Supplementary Table S8

Differentialy expressed genes (|logFC| ≥ 1 ; q-value < 0.05) in SW620 cells with the disrupted *MSX1* gene compared to SW620 cells with intact *MSX1*
Genes that were identified in Watanabe et al. (ref. No. 24) are in red.

ENTREZ	SYMBOL	GENENAME	logFC	p-value
25984	KRT23	keratin 23, type I	4.99	3.4e-08
3860	KRT13	keratin 13, type I	4.12	3.6e-05
11009	IL24	interleukin 24	3.65	9.7e-07
430	ASCL2	achaete-scute family bHLH transcription factor 2	3.51	7.6e-07
2706	GJB2	gap junction protein, beta 2, 26kDa	3.27	4.4e-07
79083	MLPH	melanophilin	3.11	3.2e-05
9289	ADGRG1	adhesion G protein-coupled receptor G1	3.05	2.3e-07
54923	LIME1	Lck interacting transmembrane adaptor 1	3.01	0.00000050
54843	SYTL2	synaptotagmin-like 2	2.99	0.00000300
1473	CST5	cystatin D	2.93	5.1e-05
4923	NTSR1	neurotensin receptor 1 (high affinity)	2.87	0.00015
56937	PMEPA1	prostate transmembrane protein, androgen induced 1	2.80	3.8e-05
80206	FHOD3	formin homology 2 domain containing 3	2.77	6.8e-06
9289	ADGRG1	adhesion G protein-coupled receptor G1	2.71	3.7e-06
4843	NOS2	nitric oxide synthase 2, inducible	2.65	2.2e-08
56937	PMEPA1	prostate transmembrane protein, androgen induced 1	2.60	2.9e-05
4071	TM4SF1	transmembrane 4 L six family member 1	2.44	7.1e-05
124056	NOXO1	NADPH oxidase organizer 1	2.44	3.9e-05
10974	ADIRF	adipogenesis regulatory factor	2.37	1.6e-05
400221	FLJ22447	uncharacterized LOC400221	2.30	3.2e-06
124056	NOXO1	NADPH oxidase organizer 1	2.16	1.7e-06
2043	EPHA4	EPH receptor A4	2.15	3.6e-05
374946	DRAXIN	dorsal inhibitory axon guidance protein	2.12	5.6e-07
114794	ELFN2	extracellular leucine-rich repeat and fibronectin type III domain containing 2	2.04	2.4e-05
83715	ESPN	espin	2.00	1.5e-05
8771	TNFRSF6B	tumor necrosis factor receptor superfamily, member 6b, decoy	1.94	0.00034
54843	SYTL2	synaptotagmin-like 2	1.94	0.00018
894	CCND2	cyclin D2	1.92	0.00011
257313	UTS2B	urotensin 2B	1.91	5.4e-05
56648	EIF5A2	eukaryotic translation initiation factor 5A2	1.87	9.3e-06
4843	NOS2	nitric oxide synthase 2, inducible	1.86	7.1e-08
894	CCND2	cyclin D2	1.86	9.9e-05
926	CD8B	CD8b molecule	1.85	5.9e-05
547	KIF1A	kinesin family member 1A	1.83	7.3e-07
926	CD8B	CD8b molecule	1.83	0.00003000
10265	IRX5	iroquois homeobox 5	1.80	1.7e-07
11009	IL24	interleukin 24	1.72	1.5e-05
283869	NPW	neuropeptide W	1.71	7.3e-05
79865	TREML2	triggering receptor expressed on myeloid cells-like 2	1.69	1.1e-05
100288413	ERVMER34-1	endogenous retrovirus group MER34, member 1	1.68	0.00041
94234	FOXQ1	forkhead box Q1	1.68	1.8e-06
1045	CDX2	caudal type homeobox 2	1.67	8.4e-05
11151	CORO1A	coronin, actin binding protein, 1A	1.65	8.5e-06
375607	NAT16	N-acetyltransferase 16 (GCN5-related, putative)	1.64	1.2e-06
124056	NOXO1	NADPH oxidase organizer 1	1.63	1.1e-05
440925	LINC01124	long intergenic non-protein coding RNA 1124	1.63	4.3e-07
147	ADRA1B	adrenoceptor alpha 1B	1.63	0.00040000
201305	SPNS3	spinster homolog 3 (Drosophila)	1.61	0.00001000
8470	SORBS2	sorbin and SH3 domain containing 2	1.61	0.00030000
10265	IRX5	iroquois homeobox 5	1.61	6.6e-05
130576	LYPD6B	LY6/PLAUR domain containing 6B	1.60	3.5e-07
3651	PDX1	pancreatic and duodenal homeobox 1	1.59	2.7e-05
1525	CXADR	coxsackie virus and adenovirus receptor	1.57	4.5e-07
91614	DEPDC7	DEP domain containing 7	1.57	7.7e-05
1366	CLDN7	claudin 7	1.57	0.00043
5010	CLDN11	claudin 11	1.57	5.3e-06
7739	ZNF185	zinc finger protein 185 (LIM domain)	1.56	5.1e-05
56648	EIF5A2	eukaryotic translation initiation factor 5A2	1.56	0.00055
83604	TMEM47	transmembrane protein 47	1.55	0.00056
7054	TH	tyrosine hydroxylase	1.55	0.00064
8470	SORBS2	sorbin and SH3 domain containing 2	1.55	3.6e-05
2295	FOXF2	forkhead box F2	1.54	3.2e-06
11033	ADAP1	ArfGAP with dual PH domains 1	1.52	0.00014
50486	G0S2	G0/G1 switch 2	1.51	6.9e-05
93273	LEMD1	LEM domain containing 1	1.50	0.00033
145447	ABHD12B	abhydrolase domain containing 12B	1.50	9.6e-05
2051	EPHB6	EPH receptor B6	1.49	1.1e-06
440900	LINC01191	long intergenic non-protein coding RNA 1191	1.48	0.00013
91614	DEPDC7	DEP domain containing 7	1.47	0.00040000
440603	BCL2L15	BCL2-like 15	1.47	0.00036
127534	GJB4	gap junction protein, beta 4, 30.3kDa	1.46	0.00018
5753	PTK6	protein tyrosine kinase 6	1.45	4.4e-06
100133190	ADIRF-AS1	ADIRF antisense RNA 1	1.45	1.2e-06
11009	IL24	interleukin 24	1.44	0.00041
26281	FGF20	fibroblast growth factor 20	1.43	0.00014
11033	ADAP1	ArfGAP with dual PH domains 1	1.43	4.5e-05
115701	ALPK2	alpha-kinase 2	1.43	0.00029
55504	TNFRSF19	tumor necrosis factor receptor superfamily, member 19	1.41	3.2e-05
28514	DLL1	delta-like 1 (Drosophila)	1.41	9.6e-05
145447	ABHD12B	abhydrolase domain containing 12B	1.39	8.9e-05
8718	TNFRSF25	tumor necrosis factor receptor superfamily, member 25	1.38	9.8e-06
9022	CLIC3	chloride intracellular channel 3	1.38	6.2e-06
90271	OLMALINC	oligodendrocyte maturation-associated long intergenic non-coding RNA	1.35	3.9e-06
2274	FHL2	four and a half LIM domains 2	1.34	0.00029
26232	FBXO2	F-box protein 2	1.33	0.00000010

440603	BCL2L15	BCL2-like 15	1.32	0.00036
9048	ARTN	artemin	1.31	1.7e-05
2870	GRK6	G protein-coupled receptor kinase 6	1.29	1.7e-05
79006	METRN	meteorin, glial cell differentiation regulator	1.28	1.2e-05
4939	OAS2	2'-5'-oligoadenylate synthetase 2, 69/71kDa	1.27	0.00017
10870	HCST	hematopoietic cell signal transducer	1.27	0.00044
2852	GPER1	G protein-coupled estrogen receptor 1	1.27	1.3e-06
353322	ANKRD37	ankyrin repeat domain 37	1.27	1.7e-05
9096	TBX18	T-box 18	1.26	8.7e-06
84634	KISS1R	KISS1 receptor	1.26	0.00040000
26281	FGF20	fibroblast growth factor 20	1.26	0.00028
9590	AKAP12	A kinase (PRKA) anchor protein 12	1.26	0.00013
23504	RIMBP2	RIMS binding protein 2	1.25	0.00013
389058	SP5	Sp5 transcription factor	1.24	4.1e-06
23616	SH3BP1	SH3-domain binding protein 1	1.24	0.00012
283748	PLA2G4D	phospholipase A2, group IVD (cytosolic)	1.24	0.00024
137075	CLDN23	claudin 23	1.24	0.00051
9590	AKAP12	A kinase (PRKA) anchor protein 12	1.24	0.00035
55057	AIM1L	absent in melanoma 1-like	1.23	0.00024
1299	COL9A3	collagen, type IX, alpha 3	1.22	0.00018
8728	ADAM19	ADAM metallopeptidase domain 19	1.22	0.00002000
2707	GJB3	gap junction protein, beta 3, 31kDa	1.21	0.00022
2274	FHL2	four and a half LIM domains 2	1.21	0.00033
7474	WNT5A	wingless-type MMTV integration site family, member 5A	1.20	5.3e-05
54436	SH3TC1	SH3 domain and tetratricopeptide repeats 1	1.20	0.00023
137994	LETM2	leucine zipper-EF-hand containing transmembrane protein 2	1.20	2.7e-06
377841	ENTPD8	ectonucleoside triphosphate diphosphohydrolase 8	1.19	0.00062
90271	OLMALINC	oligodendrocyte maturation-associated long intergenic non-coding RNA	1.19	3.4e-05
285489	DOK7	docking protein 7	1.18	3.1e-05
65997	RASL11B	RAS-like, family 11, member B	1.17	1.5e-05
2870	GRK6	G protein-coupled receptor kinase 6	1.17	0.00034
1820	ARID3A	AT rich interactive domain 3A (BRIGHT-like)	1.17	0.00023
2852	GPER1	G protein-coupled estrogen receptor 1	1.16	1.8e-05
5352	PLOD2	procollagen-lysine, 2-oxoglutarate 5-dioxygenase 2	1.15	0.00011
4828	NMB	neuromedin B	1.15	1.8e-05
64757	MARC1	mitochondrial amidoxime reducing component 1	1.15	8.1e-05
130576	LYPD6B	LY6/PLAUR domain containing 6B	1.15	5.7e-05
7262	PHLDA2	pleckstrin homology-like domain, family A, member 2	1.14	9.2e-05
29903	CCDC106	coiled-coil domain containing 106	1.14	1.9e-05
2571	GAD1	glutamate decarboxylase 1 (brain, 67kDa)	1.13	2.7e-06
3638	INSIG1	insulin induced gene 1	1.12	0.00038
5002	SLC22A18	solute carrier family 22, member 18	1.11	0.00045
59307	SIGIRR	single immunoglobulin and toll-interleukin 1 receptor (TIR) domain	1.11	0.00041
3714	JAG2	jagged 2	1.10	0.00035
146330	FBXL16	F-box and leucine-rich repeat protein 16	1.10	0.00032
2161	F12	coagulation factor XII (Hageman factor)	1.09	0.00039
860	RUNX2	runt-related transcription factor 2	1.08	0.00016
55083	KIF26B	kinesin family member 26B	1.08	1.5e-05
3422	IDI1	isopentenyl-diphosphate delta isomerase 1	1.08	0.00011
340706	VWA2	von Willebrand factor A domain containing 2	1.07	0.00024
59307	SIGIRR	single immunoglobulin and toll-interleukin 1 receptor (TIR) domain	1.07	7.5e-05
3669	ISG20	interferon stimulated exonuclease gene 20kDa	1.07	0.00038
2571	GAD1	glutamate decarboxylase 1 (brain, 67kDa)	1.07	1.4e-06
2571	GAD1	glutamate decarboxylase 1 (brain, 67kDa)	1.07	4.6e-05
1525	CXADR	coxsackie virus and adenovirus receptor	1.06	6.9e-06
50650	ARHGEF3	Rho guanine nucleotide exchange factor (GEF) 3	1.06	2.1e-06
9260	PDLIM7	PDZ and LIM domain 7 (enigma)	1.05	0.00053
4752	NEK3	NIMA-related kinase 3	1.05	1.1e-05
27040	LAT	linker for activation of T cells	1.05	0.00013
10331	B3GNT3	UDP-GlcNAc:betaGal beta-1,3-N-acetylglucosaminyltransferase 3	1.05	0.00052
55074	OXR1	oxidation resistance 1	1.04	1.7e-05
140862	ISM1	isthmin 1, angiogenesis inhibitor	1.04	0.00013
432	ASGR1	asialoglycoprotein receptor 1	1.04	1.9e-05
8739	HRK	harakiri, BCL2 interacting protein	1.03	0.00009000
727957	MROH1	maestro heat-like repeat family member 1	1.03	8.6e-05
1627	DBN1	drebrin 1	1.03	0.00011
1294	COL7A1	collagen, type VII, alpha 1	1.03	0.00010000
619279	ZNF704	zinc finger protein 704	1.02	0.00032
3714	JAG2	jagged 2	1.02	3.9e-06
619279	ZNF704	zinc finger protein 704	1.02	0.00040000
11340	EXOSC8	exosome component 8	1.02	7.1e-05
27295	PDLIM3	PDZ and LIM domain 3	1.01	1.5e-05
445	ASS1	argininosuccinate synthase 1	1.01	8.9e-05
338440	ANO9	anoctamin 9	1.01	0.00030000
79056	PRRG4	proline rich Gla (G-carboxyglutamic acid) 4 (transmembrane)	1.00	0.00021
55057	AIM1L	absent in melanoma 1-like	1.00	8.9e-05
170680	PSORS1C2	psoriasis susceptibility 1 candidate 2	-1.00	0.00024
4139	MARK1	MAP/microtubule affinity-regulating kinase 1	-1.00	0.00021
7728	ZNF175	zinc finger protein 175	-1.01	0.00005000
1540	CYLD	cylindromatosis (turban tumor syndrome)	-1.01	5.8e-05
55321	TMEM74B	transmembrane protein 74B	-1.01	0.00044
84775	ZNF607	zinc finger protein 607	-1.02	0.00054
7105	TSPAN6	tetraspanin 6	-1.02	0.00000700
8320	EOMES	eomesodermin	-1.02	0.00061
80727	TTYH3	tweety family member 3	-1.03	8.5e-05
64759	TNS3	tensin 3	-1.03	4.5e-05
1021	CDK6	cyclin-dependent kinase 6	-1.03	7.9e-06
6777	STAT5B	signal transducer and activator of transcription 5B	-1.04	0.00024
7105	TSPAN6	tetraspanin 6	-1.05	0.00013
1522	CTSZ	cathepsin Z	-1.05	0.00039
53947	A4GALT	alpha 1,4-galactosyltransferase	-1.06	0.00041

6482	ST3GAL1	ST3 beta-galactoside alpha-2,3-sialyltransferase 1	-1.08	1.5e-05
3675	ITGA3	integrin, alpha 3 (antigen CD49C, alpha 3 subunit of VLA-3 receptor)	-1.08	0.00057
91608	RASL10B	RAS-like, family 10, member B	-1.10	0.00020000
55890	GPRC5C	G protein-coupled receptor, class C, group 5, member C	-1.10	0.00055
725	C4BPB	complement component 4 binding protein, beta	-1.10	0.00054
54809	SAMD9	sterile alpha motif domain containing 9	-1.11	0.00027
51438	MAGEC2	melanoma antigen family C2	-1.11	0.00009000
91461	PKDCC	protein kinase domain containing, cytoplasmic	-1.11	4.3e-05
55616	ASAP3	ArfGAP with SH3 domain, ankyrin repeat and PH domain 3	-1.11	0.00042
124912	SPACA3	sperm acrosome associated 3	-1.12	3.4e-05
8651	SOCS1	suppressor of cytokine signaling 1	-1.12	6.9e-06
1031	CDKN2C	cyclin-dependent kinase inhibitor 2C (p18, inhibits CDK4)	-1.13	9.8e-06
114038	LINC00313	long intergenic non-protein coding RNA 313	-1.13	6.1e-05
146850	PIK3R6	phosphoinositide-3-kinase, regulatory subunit 6	-1.14	0.00063
857	CAV1	caveolin 1, caveolae protein, 22kDa	-1.15	9.8e-05
9715	FAM131B	family with sequence similarity 131, member B	-1.16	9.8e-05
79822	ARHGAP28	Rho GTPase activating protein 28	-1.16	1.3e-05
8506	CNTNAP1	contactin associated protein 1	-1.17	4.6e-05
6653	SORL1	sortilin-related receptor, L(DLR class) A repeats containing	-1.19	4.4e-05
2159	F10	coagulation factor X	-1.20	0.00012
317649	EIF4E3	eukaryotic translation initiation factor 4E family member 3	-1.20	0.00020000
745	MYRF	myelin regulatory factor	-1.20	0.00055
10628	TXNIP	thioredoxin interacting protein	-1.21	0.00016
51438	MAGEC2	melanoma antigen family C2	-1.21	1.4e-05
3708	ITPR1	inositol 1,4,5-trisphosphate receptor, type 1	-1.21	8.5e-05
9056	SLC7A7	solute carrier family 7 (amino acid transporter light chain, y+L system), member 7	-1.22	0.00024
9638	FEZ1	fasciculation and elongation protein zeta 1 (zygin I)	-1.23	0.00048
716	C1S	complement component 1, s subcomponent	-1.23	0.00039
6653	SORL1	sortilin-related receptor, L(DLR class) A repeats containing	-1.24	0.00061
943	TNFRSF8	tumor necrosis factor receptor superfamily, member 8	-1.25	3.7e-06
22920	KIFAP3	kinesin-associated protein 3	-1.25	4.9e-05
55890	GPRC5C	G protein-coupled receptor, class C, group 5, member C	-1.28	0.00034
57493	HEG1	heart development protein with EGF-like domains 1	-1.30	0.00059
8857	FCGBP	Fc fragment of IgG binding protein	-1.30	0.00058
104	ADARB1	adenosine deaminase, RNA-specific, B1	-1.31	0.00003000
23228	PLCL2	phospholipase C-like 2	-1.35	0.00033
2192	FBLN1	fibulin 1	-1.35	0.00025
104	ADARB1	adenosine deaminase, RNA-specific, B1	-1.40	2.4e-06
104	ADARB1	adenosine deaminase, RNA-specific, B1	-1.41	1.2e-05
6578	SLCO2A1	solute carrier organic anion transporter family, member 2A1	-1.42	4.1e-05
3689	ITGB2	integrin, beta 2 (complement component 3 receptor 3 and 4 subunit)	-1.42	0.00049
4613	MYCN	v-myc avian myelocytomatosis viral oncogene neuroblastoma derived homolog	-1.44	0.00016
57574	MARCH4	membrane-associated ring finger (C3HC4) 4, E3 ubiquitin protein ligase	-1.46	0.00021
54549	SDK2	sidekick cell adhesion molecule 2	-1.47	0.00043
79694	MANEA	mannosidase, endo-alpha	-1.53	0.00062
3814	KISS1	KiSS-1 metastasis-suppressor	-1.59	0.00006000
25894	PLEKHG4	pleckstrin homology domain containing, family G (with RhoGef domain) member 4	-1.61	0.00053
7032	TFF2	trefoil factor 2	-1.62	0.00027
4137	MAPT	microtubule-associated protein tau	-1.63	3.1e-05
55244	SLC47A1	solute carrier family 47 (multidrug and toxin extrusion), member 1	-1.66	9.2e-05
5159	PDGFRB	platelet-derived growth factor receptor, beta polypeptide	-1.66	2.6e-05
343990	KIAA1211L	KIAA1211-like	-1.66	2.7e-06
30846	EHD2	EH-domain containing 2	-1.66	3.4e-05
343990	KIAA1211L	KIAA1211-like	-1.68	2.7e-07
2192	FBLN1	fibulin 1	-1.74	0.00016
162494	RHBDL3	rhomboid, veinlet-like 3 (Drosophila)	-1.76	5.2e-05
146850	PIK3R6	phosphoinositide-3-kinase, regulatory subunit 6	-1.85	0.00022
641700	ECSCR	endothelial cell surface expressed chemotaxis and apoptosis regulator	-1.86	0.00050000
3689	ITGB2	integrin, beta 2 (complement component 3 receptor 3 and 4 subunit)	-1.89	9.6e-05
6448	SGSH	N-sulfoglucosamine sulfohydrolase	-1.93	4.7e-06
5654	HTRA1	HtrA serine peptidase 1	-1.93	0.00019
5138	PDE2A	phosphodiesterase 2A, cGMP-stimulated	-2.09	0.00056
3851	KRT4	keratin 4, type II	-2.15	0.00043
946	SIGLEC6	sialic acid binding Ig-like lectin 6	-2.37	1.3e-06
6280	S100A9	S100 calcium binding protein A9	-2.40	0.00038
2018	EMX2	empty spiracles homeobox 2	-2.58	0.00049
2018	EMX2	empty spiracles homeobox 2	-2.76	0.00016
1277	COL1A1	collagen, type I, alpha 1	-2.87	0.00019
24141	LAMP5	lysosomal-associated membrane protein family, member 5	-2.90	2.1e-07

Supplementary Table S9

Colorectal neoplasia specimens summary
HYP, hyperplasia; LGD, low-grade dysplasia;
HGD, high-grade dysplasia, CRC, carcinoma

	**Age** (median, min.-max.)	**Gender** (M/F)
HYP	69 (59-77)	6/3
LGD	67 (53-89)	12/15
HGD	64 (36-85)	15/9
CRC	82 (63-90)	6/6

Supplementary Table S10

List of primers used for qRT-PCR

Gene	Organism	Forward primer sequence (5´ to 3´)	Reverse primer sequence (5´ to 3´)
β-actin	Mouse	GATCTGGCACCACACCTTCT	GGGGTGTTGAAGGTCTCAAA
β-actin	Human	GGCATCCTCACCCTGAAGTA	AGGTGTGGTGCCAGATTTTC
ABHD12B	Human	CGGAAGAAAAATTGCTGCTC	TCACCCCAGGTTCAACTCTC
Ascl2	Mouse	AAGCACACCTTGACTGGTACG	AAGTGGACGTTTGCACCTTCA
ASCL2	Human	GCGAGCTACTCGACTTCTCC	CTCGGCTTCCGGGGCTGAGG
Axin2	Mouse	TAGGCGGAATGAAGATGGAC	CTGGTCACCCAACAAGGAGT
AXIN2	Human	TGAGGTCCACGGAAACTGTTGACAGT	CCCTCCCGCGAATTGAGTGTGA
Bves	Mouse	GAACTGGCGAGAGATTCACC	ATCATCACATCCAAGGCACA
CDX2	Human	TGCTGCAAACGCTCAACCCCGG	CGGCTTTCCTCCGGATGGTGATG
Chromogranin A (CHGA)	Mouse	GCGCCGGGCAAGTTTTTGCC	GGGCTGGGTTTGGACAGCGAG
CTSZ	Human	GCTTCTGCTGCTCGTGCT	GTTGACACCATCCACATTGC
CTNNB1 (β-catenin)	Human	TTCCAGACACGCTATCATGC	AATCCACTGGTGAACCAAGC
DEPDC7	Human	ACCTAAGAGGCAGTCCACCA	GTCTGGTTGCTCAGGAAAGC
EGFP	Aequorea victoria	GACGTAAACGGCCACAAGTT	GAACTTCAGGGTCAGCTTGC
ENTPD8	Human	AGCGTCTAAGCACAGCTTCC	TCCACCAGGAGGAGAATGAG
GAPDH	Mouse	AACTTTGGCATTGTGGAAGG	ATCCACAGTCTTCTGGGTGG
KRT23	Human	GCCTCCGAAGGACCTTAGAC	AGATCTTCCCTGGGACCTGT
Lgr5	Mouse	CCTGTCCAGGCTTTCAGAAG	CTGTGGAGTCCATCAAAGCA
Mdga1	Mouse	CCTCACACCCTACACCACCT	GGGCCCAGTATTAGGAGAGC
Msx1	Mouse	CTCTCGGCCATTTCTCAGTC	TTGGTCTTGTGCTTGCGTAG
MSX1	Human	AGAAGATGCGCTCGTCAAAG	GGCTTACGGTTCGTCTTGTG
Msx2	Mouse	AATTCCGAAGACGGAGCAC	CGGTTGGTCTTGTGTTTCCT
MSX2	Human	CGGTCAAGTCGGAAAATTCA	GAGGAGCTGGGATGTGGTAA
Mtus2	Mouse	TCGTCCTCCTGGCTATTCAC	CCCTTGGGTGTGTCCTTAGA
Mylk3	Mouse	CCCAGGAAGAACTGAAGCTG	CGACCCCCTCCTAAGACTTC
Nkd1	Mouse	AGGACGACTTCCCCCTAGAA	TGCAGCAAGCTGGTAATGTC
NKD1	Human	GCCTCCTGAGAAGACTGACG	TTGCCGTTGTTGTCAAAGTC
RASL10B	Human	GGGGAGCCCCTACTTCTCTC	ACCGTCAGGACCAACCATTG
RASL11B	Human	CCTGGCTCTTCAGGTTCAAG	GTGGAGCTGGCTGATGAGTT
Slc5a6	Mouse	GCCCTAGGAATTGTCTGCAA	GGCAAGGGAACACTGCATAG
SORBS2	Human	AATTCACATGGTGCACAAGC	AGACCGATCTCTTGGTCGAA
SP5	Mouse	GGACAGGAAACTGGGTCGTA	AATCGGGCCTAGCAAAAACT
SP5	Human	ACTTTGCGCAGTACCAGAGC	ACGTCTTCCCGTACACCTTG
Stk32b	Mouse	GTGCAGAAGCGAGACACAAA	CTGTAGGTGGTAGCGCAGGT
Sucrose Isomaltase (SI)	Mouse	TTCAAGAAATCACAACATTCAATTTACCTAG	CTAAAACTTTCTTTGACATTTGAGCAA
TMEM47	Human	TGCCATCATTCTCATTGCAT	AACCCCAGTTGAACTCATGG
TNFRSF19 (Troy)	Mouse	GCTCAGGATGCTCAAAGGAC	CCAGACACCAAGACTGCTCA
TNFRSF19 (TROY)	Human	CTATGGGGAGGATGCACAGT	TCTCCACAAGGCACACACTC
Trpm	Mouse	GGCACACAGAGTGGACTTGA	AAGCCACGAAAATCTGATCG
Ttn	Mouse	CCTGCCTCAGTGGAAGAGAC	TTCTGGCTCTGGTTCCAGTT
Ubb	Mouse	ATGTGAAGGCCAAGATCCAG	TAATAGCCACCCCTCAGACG
UBB	Human	GCTTTGTTGGGTGAGCTTGT	TCACGAAGATCTGCATTTTGA

Supplementary Table S10

List of primers used for cDNA amplification for subsequent cloning into pBluescript KS II vector (designed for ISH probes synthesis)

Gene	Organism	Forward primer sequence (5´ to 3´)	Reverse primer sequence (5´ to 3´)
Ascl2	Mouse	AGTGGATCCATGGAAGCACACCTTGACTG	GAGGTCGACTCAGTAGCCCCCTAACCAAC

Supplementary Table S10

List of oligos used for lentiCRISPR and pARv-RFP cloning

Gene	Organism	Forward primer sequence (5´ to 3´)	Reverse primer sequence (5´ to 3´)	Recipient plasmid name
APC	Human	CACCGACTGCTGGAACTTCGCTCAC	AAACGTGAGCGAAGTTCCAGCAGTC	lentiCRISPR
APC	Human	CCTGTGAGCGAAGTTCCAGCAGTGTCCGAT	CGGACACTGCTGGAACTTCGCTCACAGG	pARv-RFP for lentiCRISPR
MSX1	Human	CACCGAGGCGCTCATGGCCGACCAC	AAACGTGGTCGGCCATGAGCGCCTC	lentiCRISPR#1
MSX1	Human	CACCGCCCACCGAGAAATGGCCGAG	AAACCTCGGCCATTTCTCGGTGGGC	lentiCRISPR#2
MSX1	Human	TGGAGGCGCTCATGGCCGACCACAGGCGAT	CGCCTGTGGTCGGCCATGAGCGCCTCCA	pARv-RFP for lentiCRISPR#1

Supplementary Table S10

List of primers used for amplification of CRISPR-targeted sites from genomic DNA

Gene	Organism	Primer sequence (5′ to 3′)	name in figure
MSX1	Human	CACTACAGGAAGCTAGCTTCTTCCCGCAAGG	P1
MSX1	Human	GGCAAAGAAGTCATGTCAGCAGCCGGGGCC	P2
MSX1	Human	GGCTGGCCAGTGCTGCGGCAGAAGGG	P3
MSX1	Human	CACGCCATTGAAATCTGGCTGCTATTATGCCGAG	P4

List of primers used for amplification of SP5 promoter element

Gene	Organism	Forward primer sequence (5′ to 3′)	Reverse primer sequence (5′ to 3′)
SP5	Human	GCGGGTACCGCGAGGGTGCAGGGTGTGCAAGTAAA	GCACGGAGTACCAGGAGAGA

Supplementary Table S10

List of primers used for analysis of gene regulatory regions occupancy by MSX1

Gene	Organism	Forward primer sequence (5´ to 3´)	Reverse primer sequence (5´ to 3´)	Primers number in Figures
ASCL2	Human	CCTTGGTAAATACTTCACGTGC	CAGTCAGAGTCTTCAACACTCC	P1 and P2
ASCL2	Human	GCCTGCTTTTGTATTGCCCA	AGTTTCAGCCTCCCGAGTAG	P3 and P4
ASCL2	Human	GACGGCTCAGATAGTGTGGA	CACCACCAACACCTCTCTCT	P5 and P6
SP5	Human	CCCCCTTTGATCAGGAAAAT	GCTTCAGGATCACCTCCAAG	
